# Data on systematic review and meta-analysis of epidemiologic evidence on the association between perineal use of talc powder and risk of ovarian cancer

**DOI:** 10.1016/j.dib.2020.105277

**Published:** 2020-02-20

**Authors:** Mohamed Kadry Taher, Nawal Farhat, Nataliya A. Karyakina, Nataliya Shilnikova, Siva Ramoju, Christopher A. Gravel, Kannan Krishnan, Donald Mattison, Shi-Wu Wen, Daniel Krewski

**Affiliations:** a Risk Sciences International, 251 Laurier Ave W, Suite 700, Ottawa, ON K1P 5J6, Canada; b McLaughlin Centre for Population Health Risk Assessment, Faculty of Medicine, University of Ottawa, 600 Peter Morand Crescent, Ottawa, ON, K1G 5Z3, Canada; c School of Epidemiology and Public Health, University of Ottawa, 600 Peter Morand Crescent, Ottawa, ON, K1G 5Z3, Canada; d Department of Epidemiology, Biostatistics and Occupational Health, McGill University, 1020 Pine Avenue West, Montreal, Qc, H3A 1A2, Canada; e OMNI Research Group, Department of Obstetrics and Gynaecology, University of Ottawa Faculty of Medicine, Ottawa, ON, Canada; f Clinical Epidemiology Program, Ottawa Hospital Research Institute, Ottawa, ON, Canada; g School of Public Health, Central South University, Changsha, China

**Keywords:** Talc, Perineal exposure, Ovarian cancer, Observational study, Systematic review, Meta-analysis

## Abstract

This paper describes data from a systematic review and meta-analysis [1] conducted to identify and evaluate published peer reviewed evidence on the association between perineal use of talc powder and risk of ovarian cancer. These data were collected from multiple electronic bibliographic databases, as well as from grey literature sources, without applying time, language or other filters. A meta-analysis was conducted to quantitatively assess the ovarian cancer risk in relation to talc use and other potential risk factors.

**Table:** Specifications Table

Subject	Medicine and Dentistry
Specific subject area	Obstetrics, Gynecology and Women's Health
Type of data	Tables and Figures
How data were acquired	Data were acquired directly from published original studies
Data format	Filtered and summarized
Parameters for data collection	Data from original, peer-reviewed observational studies on the association between perineal use of talc powder and risk of ovarian cancer.
Description of data collection	A systematic review of eight electronic databases was independently conducted by two reviewers. Additional peer reviewed articles were also identified in the grey literature. After applying relevant inclusion/exclusion criteria, 30 original studies (4 cohort studies and 26 case-control studies) were subject to detailed qualitative assessment and data extraction. After excluding studies that examined overlapping populations, a series of meta-analyses of risk estimates reported in 27 of these studies was conducted.
Data source location	Institution: Risk Sciences International (www.risksciences.com) City: Ottawa Country: Canada
Data accessibility	Data are available as supplementary material to the related research article at: https://ars.els-cdn.com/content/image/1-s2.0-S0890623818306373-mmc1.pdf
Related research article	M. Kadry Taher, N. Farhat, N.A. Karyakina, N. Shilnikova, S. Ramoju, C.A. Gravel, K. Krishnan, D. Mattison, S.W. Wen, D. Krewski, Critical Review of the Association between Perineal Use of Talc Powder and Risk of Ovarian Cancer, *Reprod Toxicol* 90 (2019) 88–101 [1]

**Table:** 

**Value of the Data** • These data describe epidemiologic evidence published between 1982 – 2016 on the risk of ovarian cancer in relation to perineal use of talc powder in diverse populations from different geographic and ethnic backgrounds. • The data were developed using a comprehensive systematic review involving 8 electronic bibliographic databases and multiple grey literature sources. • The data were sufficiently rich to support a series of detailed meta-analyses, including an overall main analysis and 16 subgroup analyses describing the impact of a number of relevant factors on ovarian cancer risk.

## 1 Data

Perineal exposure to talc powder has been repeatedly questioned as a possible cause of human ovarian cancer. A robust, multi-step search and screening of evidence was conducted to identify original, peer-reviewed evidence from human subjects. Extracted data include detailed population demographics and risk of ovarian cancer in relation to a wide range of possible risk factors. Data extracted from these studies are summarized in Table 1 in the main review, and detailed in Supplementary Material VI [1].

We conducted meta-analyses through pooling and analyzing the reported risk estimates, while ensuring the inclusion of data-rich studies and avoiding the inclusion of multiple studies reporting on the same population. Data extracted from these studies are summarized in Table 2 in the main review, and detailed in Supplementary Material XI [1]. The rationale for decisions on selecting data from overlapping studies for inclusion in the meta-analysis is shown in Supplementary Material XI: Table 8 [1].

## 2 Experimental design, materials, and methods

We conducted a robust systematic search of 8 electronic bibliographic databases and other grey literature sources for original human studies examining the association of perineal use of talc powder and the risk of ovarian cancer. The search was conducted according to the PRISMA (Preferred Reporting Items for Systematic Reviews and Meta-Analyses) guidelines, and following the specific guidance provided by the Cochrane Collaboration [2]. Searched databases include Medline (Ovid), EMBASE, Cochrane, PubMed, CINAHL, WHO Clinical Trials Registry (ICTRN), US Clinical Trials Database, UK Clinical Trials Gateway, and grey literature sources. No language, time or other filters were applied to limit the search output.

A robust search strategy was designed and conducted by two reviewers (MT and NF), followed by automated then manual removal of duplicate studies. Identified studies were independently screened and assessed by the two reviewers using Distiller SR software [3]. A total of 30 original studies were retained for further in-depth assessment after applying predetermined inclusion/exclusion criteria.

Quality of the included studies was also assessed independently using the Newcastle-Ottawa Scale [4] based on 3 domains: criteria for selection of study groups; comparability of the study groups; and ascertainment of either the exposure or outcome of interest for case-control or cohort studies respectively (see details in Supplementary Material V [1]). Any disagreement was resolved via consensus between the two reviewers at each stage of the review. Four experienced investigators were also available to provide guidance throughout the entire review process.

A summary of the systematic review strategy is provided in the Materials and Methods section of the main review [1]. Additional details on the systematic review strategy, database search terms (Supplementary Material II), study assessment and data extraction (Supplementary Material III), reasons for study exclusion (Supplementary Material IV), and quality assessment [4] (Supplementary Material V). All Supplementary Material are available online [Download Acrobat PDF file (2MB) https://ars.els-cdn.com/content/image/1-s2.0-S0890623818306373-mmc1.pdf].

In conducting the meta-analysis, we considered the reported maximally-adjusted risk estimates (relative risks, odds ratios or hazard ratios) to be practically equivalent, based on the rarity of the endpoint of interest, ovarian cancer. We pooled these estimates using random effects model with inverse variance weighting to obtain an overall risk estimate and associated 95% confidence interval [5]. We used *I*^*2*^, a Chi-Squared statistic, to assess heterogeneity among studies combined in the meta-analysis [2]. This statistic evaluates whether the differences observed among studies are due to chance or possibly due to structural differences among studies (heterogeneity). The random effects model helped to account for heterogeneity among the studies included in the meta-analysis [5].

The initial analysis involved an evaluation of ovarian cancer risk in relation to genital/perineal use of talc powder (ever vs never use). Sixteen additional subgroup analyses were performed to assess the effects of duration and frequency of talc use, tumor histology, tumor behavior, and the possible effect of menopausal state, hormone use, and pelvic surgery. Additionally, we assessed possible effects of the study design, type of study controls, Newcastle Ottawa Scale (NOS) quality score [4], and publication year, as well as the influence of individual studies on the overall pooled effect [6]. The results of the meta-analyses are summarized in Table 2 in the main review, and detailed in Supplementary Material XI [1].
