# Supplementary material for: Data on systematic review and meta-analysis of epidemiologic evidence on the association between perineal use of talc powder and risk of ovarian cancer
Source: Data Brief. 2020 Feb 20;29:105277. doi: 10.1016/j.dib.2020.105277 (PMC7049564; doi:10.1016/j.dib.2020.105277)
Supplement: Multimedia component 1 [file mmc1.pdf]

Critical Review of the Association between Perineal Use of Talc Powder and Risk of Ovarian Cancer

Mohamed Kadry Taher[A, B, C], Nawal Farhat[A, B, C], Nataliya A. Karyakina[A, B], Nataliya Shilnikova[A, B], Siva Ramoju[A], Christopher A. Gravel[B, C, D], Kannan Krishnan[A], Donald Mattison[A, B, C], Shi-Wu Wen[C, E, F, G], Daniel Krewski[A, B, C]

A. ***Risk Sciences International,*** *251 Laurier Ave W, Suite 700, Ottawa, ON K1P 5J6, Canada*
B. ***McLaughlin Centre for Population Health Risk Assessment, Faculty of Medicine, University of Ottawa,*** *600 Peter Morand Crescent, Ottawa, ON, K1G 5Z3, Canada*
C. ***School of Epidemiology and Public Health, University of Ottawa,*** *600 Peter Morand Crescent, Ottawa, ON, K1G 5Z3, Canada*
D. ***Department of Epidemiology, Biostatistics and Occupational Health, McGill University,*** *1020 Pine Avenue West, Montreal, Qc, H3A 1A2, Canada*
E. ***OMNI Research Group, Department of Obstetrics and Gynaecology, University of Ottawa Faculty of Medicine,*** *Ottawa, ON, Canada*
F. ***Clinical Epidemiology Program, Ottawa Hospital Research Institute,*** *Ottawa, ON, Canada*
G. ***Nanhai Hospital, Southern Medical University,*** *Guangzhou, China*

Corresponding Author: *Dr. Mohamed Taher (Mohamed.Taher@uOttawa.ca)*

Corresponding Address: *600 Peter Morand Crescent, Room 216, Ottawa, ON, K1G 5Z3, Canada*

Supplementary Material

Table of Contents

List of Tables vi

List of Figures vii

List of Acronyms viii

Supplementary Material I: Consideration of talc carcinogenicity by national and international agencies 11

Supplementary Material II: Literature review – human studies 25

II.1. Review strategy 25

II.2. Systematic literature search and identification of relevant studies 27

II.3. Evaluation of quality of retained human studies 29

II.4. Database search strategy 30

EMBASE 30

Medline Ovid 32

PubMed 34

Cochrane Library (Wiley) 39

CINAHL 40

WHO Clinical Trials Registry 41

US Clinical Trials Database 44

UK Clinical Trials Gateway 46

Grey Literature 48

II.5. Database search results 49

Supplementary Material III: Distiller SR forms for screening and data abstraction - human studies 51

Level 1 (title and abstract screening) 51

Level 2 (full-text screening) 52

Level 3 (data abstraction) 53

Supplementary Material IV: List of excluded human studies with reasons for exclusion60
Level 1 – Reasons for exclusion 60
Level 2 – Reasons for exclusion 68
Level 3 – Reasons for exclusion 96
Supplementary Material V: Newcastle-Ottawa Scale for quality assessment of non-randomized studies 97
V.1. Newcastle-Ottawa Scale for case-control studies 98
V.2. Newcastle-Ottawa Scale for cohort studies 99
Supplementary Material VI: Summary of studies included in the systematic review of human epidemiologic studies 101
Supplementary Material VII: Strengths and limitations of included studies identified by the original study authors and the authors of the current review 216
Supplementary Material VIII: Literature search strategy for non-human studies 232
VIII.1. Literature search strategy and identification of relevant non-human studies . 232
VIII.2. Database search strategy 234
Embase 234
Medline Ovid 237
Toxline Proquest 239
VIII.3. Database search results 241
Supplementary Material IX: Inclusion-exclusion criteria for non-human studies 242
Supplementary Material X: Summary of non-human studies included in this review .. 244
X.1. Animal studies on talc (published up to 2006) 244
X.2. Animal studies on talc (published after 2006) 270
X.3. In-Vitro studies on talc 287
X.4. Absorption, distribution and elimination of talc by different routes of exposure 301
Supplementary Material XI: Meta-analysis of retained human studies on perineal talc exposure 317

Methodology 317
Overall perineal talc use 320
Perineal talc use and ovarian cancer 320
Forest plots of the subgroup analyses of perineal talc use 321
Perineal talc use and ovarian cancer by race/ethnicity 321
Perineal talc use – assessment of reporting studies 322
Perineal talc use and ovarian cancer, by study design 322
Perineal talc use and ovarian cancer, by type of study controls 323
Perineal talc use and ovarian cancer, by score of the New Castle Ottawa Scale (NOS) 324
Perineal talc use (ever vs. never) and ovarian cancer, by the study's year of publication 325
Perineal talc use – assessment of talc exposure 326
Perineal talc use and ovarian cancer, by frequency of use 326
Perineal talc use and ovarian cancer, by duration of use 327
Perineal talc use and ovarian cancer, by type of use 328
Perineal talc use – tumor histology 329
Perineal talc use and ovarian cancer, by tumor histology 329
Perineal talc use– tumor behavior 329
Perineal talc use and ovarian cancer, by overall tumor behavior 330
Perineal talc use and ovarian cancer, for serous ovarian tumors 330
Perineal talc use and ovarian cancer, for mucinous ovarian tumors 331
Perineal talc use and ovarian cancer, for endometrioid ovarian tumors 332
Perineal talc use and ovarian cancer, for clear-cell ovarian tumors 333
Perineal talc use – modifying factors 334
Perineal talc use and ovarian cancer, by menopausal state 334

Perineal talc use and ovarian cancer, by pelvic surgery 335
Perineal talc use and ovarian cancer – influence analysis 336
References 338

List of Tables

Table 1: Assessment of perineal exposure to body powder among women 12
Table 2: IARC classification of talc carcinogenicity following perineal exposure 14
Table 3: Exposure limits and considerations on talc carcinogenicity in occupational settings 15
Table 4: Regulation of talc used in foodstuff by different public health government organizations and agencies 17
Table 5: Regulatory decisions on talc use in cosmetic products 21
Table 6: Comparison of studies included in the meta-analysis of Berge et al. (2017), Penninkilampi et al. (2018) with studies included in the current review 22
Table 7: Recent reports* on association of perineal exposure to talc and ovarian cancer 24
Table 8: Rationale for exclusion of specific original studies from this review 318
Table 9: Influence analysis of individual studies included in this meta-analysis 336

List of Figures

Figure 1. Steps involved in evaluation of the association between perineal use of talc and ovarian cancer risk 26
Figure 2: Influence analysis study. 337

List of Acronyms

**ANSES**	French Agency for Food, Environmental and Occupational Health & Safety
**ACGIH**	American Conference of Governmental Industrial Hygienists
**BMD**	Benchmark dose
**BMDL**	Benchmark dose lower confidence limit
**BMI**	Body mass index
**CAS**	Chemical Abstracts Service
**CEPA**	Canadian Environmental Protection Act
**CI**	Confidence interval
**CMP**	Chemicals Management Plan
**CRP**	C-reactive protein
**DFG**	Deutsche Forschungsgemeinschaft
**ECHA**	European Chemical Agency
**EOC**	Epithelial Ovarian Cancer
**EPA**	US Environmental Protection Agency
**ESRAB**	Existing Substances Risk Assessment Bureau
**FAO**	Food and Agriculture Organization
**FSH**	Follicle Stimulating Hormone
**GLP**	Good Laboratory Practice
**GMP**	Good Manufacturing Practice
**GRAS**	Generally Recognized as Safe
**GRF**	German Research Foundation
**HR**	Hazard Ratio
**HT**	Hormone Therapy
**IARC**	International Agency for Research on Cancer
**ICD**	International Classification of Diseases
**IOFI**	International Organization of the Flavor Industry
**JECFA**	Joint FAO/WHO Expert Committee on Food Additives
**LH**	Luteinizing Hormone
**LOAEL**	Lowest observed adverse effect level
**MAK**	Maximale Arbeitsplatz-Konzentration *(maximum workplace concentration)*

**NECC**	New England Case Control
**NHS**	Nurses' Health Study
**NOAEL**	No observed adverse effect level
**NOS**	Newcastle Ottawa Scale
**NTP**	National Toxicology Program
**OC**	Oral contraceptive
**OR**	Odds Ratio
**OSHA**	Occupational Safety and Health Administration
**PEL**	Permissible Exposure Limit
**PID**	Pelvic Inflammatory Disease
**PMN**	Polymorphonuclear Neutrophils
**PRISMA**	Preferred Reporting Items for Systematic Reviews and Meta-Analyses
**RCT**	Randomized Controlled Trial
**RDD**	Random Digit Dialing
**REACH**	Registration, Evaluation, Authorization and Restriction of Chemicals , Belgium
**ROS**	Reactive Oxygen Species
**RR**	Relative Risk
**RSI**	Risk Sciences International
**SEER**	Surveillance, Epidemiology, and End Results Program
**TLV**	Threshold Limit Value
**TWA**	Time Weighted Average
**USP**	United States Pharmacopeia

Critical Review of the Association between Perineal Use of Talc and Risk of Ovarian Cancer

Supplementary Material I – VII

Human Studies

Supplementary Material I: Consideration of talc carcinogenicity by national and international agencies

In addition to peer-reviewed publications, web-based resources were searched for talc carcinogenic hazard assessment documents produced by national and international public health regulatory agencies. The grey literature search was conducted through Google and Google Scholar engines keeping the focus of the search on the talc carcinogenic hazard and risk assessment documents developed by national and international agencies. Specifically, the following agencies were targeted: American Conference of Governmental Industrial Hygienists (ACGIH), French Agency for Food, Environmental and Occupational Health & Safety, France (ANSES), Agency for Toxic Substances and Disease Registry (ATSDR), Food and Agriculture Organization of the United Nations/World Health Organization (FAO/WHO), Health Canada, Health Council of the Netherlands (Health Council of the Netherlands), Environment Protection Agency, United States (US EPA), European Chemical Agency (ECHA), European Food Safety Authority (EFSA), German Research Foundation (GRF), International Agency for Research on Cancer (IARC), International Organization of the Flavor Industry IOFI), Food and Drug Administration, United States (US FDA), Standards for Food Additives (SFA), Joint FAO/WHO Expert Committee on Food Additives (JECFA), the National Toxicology Program, United States (US NTP), National Institute for Public Health and the Environment, the Netherlands (RIVM), New Zealand Ministry of Health, Occupational Safety and Health Administration, United States (US OSHA), Registration, Evaluation, Authorisation and Restriction of Chemicals Program (REACH), World Health Organization (WHO). Where available, results are provided in the following Tables.

Table 1: Assessment of perineal exposure to body powder among women[1]

Location	No. of Controls	Prevalence of Ever Use of Talc	Type of Perineal Use of Powder by Women	Reference
Massachusetts, USA	21	28.4%	Exposure to talc by dusting	Cramer et al. (1982) [2]
Washington DC, USA	171	1.8%	Body talc	Hartge et al. (1983) [3]
California, USA	539	45.8%	Use of talcum powder	Whittemore et al. (1988) [4]
United Kingdom	451	59.0%	Use of talc	Booth et al. (1989) [5]
Washington, USA	158	40.5%	Exposure to powder (cornstarch, baby powder, talc, deodorizing powder); detailed information on type of powder used	Harlow & Weiss (1989) [6]
China	224	2.2%	Dusting powder	Chen et al. (1992) [7]
Massachusetts, USA	239	39.3%	Exposure to baby powder, deodorizing or scented powder	Harlow et al. (1992) [8]
Maryland, USA	46	17.3%	Genital bath talc (also asked use on napkins or diaphragm)	Rosenblatt et al. (1992) [9]
Athens, Greece	193	3.6%	Local application of talc	Tzonou et al. (1993) [10]
Israel	408	5.6%	Use of talc	Shushan et al (1996). [11]
Toronto, Canada	564	35.6%	Regular application of talc	Chang & Risch (1997) [12]

[1] Source: [1] IARC/International Agency for Research on Cancer, Carbon black, titanium dioxide, and talc, IARC Monogr Eval Carcinog Risks Hum 93 (2010) 1-413.

Location	No. of Controls	Prevalence of Ever Use of Talc	Type of Perineal Use of Powder by Women	Reference
Washington, USA	422	39.3%	Dusting with cornstarch, talcum powder, baby or scented powder, and deodorizing spray	Cook et al. (1997) [13]
New York, USA	50	26%	Use of talc	Eltabbakh et al. (1998) [14]
Montreal, Canada	170	4.7%	Use of talc	Godard et al. (1998) [15]
New England, USA	523	18.2%	Use of talc, baby or deodorizing powders or cornstarch	Cramer et al. (1999) [16]
New York, USA	693	35%	Use of talc (on genital or thigh area and sanitary napkins)	Wong et al. (1999) [17]
Delaware Valley, USA	1367	40%	Use of talc (on genital/rectal area and feet, sanitary napkins, underwear, diaphragm/cervical cap, male partner user)	Ness et al. (2000) [18]
California, USA	1122	37.1%	Use of talcum powder	Mills et al. (2004) [19]
USA	78 630 cohort	40.4%	Use of talc	Gertig et al. (2000) [20]

Table 2: IARC classification of talc carcinogenicity following perineal exposure

Agency	Conclusion	Comments
IARC (2010), France [1]	Perineal use of talc-based body powder.<br>Possibly carcinogenic to humans (Group IIB).	This category is used for agents, mixtures and exposure circumstances for which there is limited evidence of carcinogenicity in humans and less than sufficient evidence of carcinogenicity in experimental animals. It may also be used when there is inadequate evidence of carcinogenicity in humans but there is sufficient evidence of carcinogenicity in experimental animals. In some instances, an agent, mixture or exposure circumstance for which there is inadequate evidence of carcinogenicity in humans but limited evidence of carcinogenicity in experimental animals together with supporting evidence from other relevant data may be placed in this group.

Table 3: Exposure limits and considerations on talc carcinogenicity in occupational settings

Agency	Conclusion	Comments
**Inhalation hazard**		
IARC (2010), France [1]	Inhaled talc not containing asbestos or asbestiform fibers.<br>Occupational exposure<br>Not classifiable as to its carcinogenicity (Group 3).	Group III: The agent (mixture or exposure circumstance) is not classifiable as to its carcinogenicity to humans.<br>This category is used most commonly for agents, mixtures and exposure circumstances for which the evidence of carcinogenicity is inadequate in humans and inadequate or limited in experimental animals.<br>Exceptionally, agents (mixtures) for which the evidence of carcinogenicity is inadequate in humans but sufficient in experimental animals may be placed in this category when there is strong evidence that the mechanism of carcinogenicity in experimental animals does not operate in humans.<br>Agents, mixtures and exposure circumstances that do not fall into any other group are also placed in this category.
ACGIH (2016), USA [21]	Not classifiable as a Human Carcinogen.<br><br>TLV – for particulate matter containing no asbestos and <1% crystalline silica (respirable fraction).	Talc, containing no asbestos fibers.<br>Occupational exposure.

Agency	Conclusion	Comments
OSHA (2013), United States [22]	Permissible Exposure Levels/PEL<br>20x 10^6 particles per cubic foot	Talc, containing no asbestos.<br>(Talc containing asbestos - use asbestos limit).<br>Occupational exposure.
GRF-German Research Foundation (2006) [23]	Carcinogen category: 3B.	Talc, containing no asbestos.<br>Occupational exposure.
Health Council of the Netherlands (2012) [24]	Data are insufficient to evaluate the carcinogenic properties of talc<br>Category 3.	Talc, containing no asbestos.<br>Occupational exposure.
ANSES (2012), France [25]	"...unable to conclude on the carcinogenicity of talc contaminated with<br>non-asbestiform amphibole fibres (including ATA fibres), or with cleavage fragments from other minerals."	Talc, containing no asbestos but non-asbestiform amphibole fibres.<br>Occupational exposure.
REACH (2017), Belgium [26]	Not classified as a carcinogen according to the Globally Harmonized System of Classification and Labelling of Chemicals.	Occupational exposure.

Table 4: Regulation of talc used in foodstuff by different public health government organizations and agencies

Agency	Food	Value	Limitations/Exception
Health Canada (2011) [27]	Dried split legumes	GMP	"The evaluation of available data supports the safety and effectiveness of this food additive in the above specified use. Since there are no safety concerns regarding the use of this food additive on dried split legumes, the Regulations are amended to permit the extended use of talc on dried split legumes at a maximum level of use consistent with good manufacturing practice. "
Health Canada, (2003) [28]	Chewing gum	GMP	"Therefore, it is the intention of Health Canada to recommend that the Food and Drug Regulations be amended to permit the use of talc as a dusting agent in the manufacture of chewing gum at a maximum level of use consistent with good manufacturing practice."
FDA (2015) USA [29]	All food	GRAS (generally recognized as safe) - 182.2437	Talc is "generally recognized as safe. Substances in this category are by definition not food additives. Most GRAS substances have no quantitative restrictions as to use although their use must conform to good manufacturing practice."
FDA (2009) USA [30]	Drugs generally	GMP 73.1550	Color additives exempt for certification and permanently listed for drug use. None of these color additives may be used in products that are for use in the area of the eye unless otherwise indicated.
FDA (2015), USA [31]	No data	ASP	"Fully up-to-date toxicology information has been sought".
Japanese Existing Food	Number 194	-	Talc is "included in the List of food additives from natural origin compiled by the Ministry of Health and Welfare."

Agency	Food	Value	Limitations/Exception
Additives (2014) [32]			
Japanese Standards for Food Additives (2014) [33]	All food	0.50% (as maximum residue limit)	Water-insoluble minerals: Talc<br>Only in case where its use is indispensable for manufacture or processing of food.
	Chewing gum (when talc is only used)	5.0% (as maximum residue limit)	Only in cases where its use is indispensable for manufacture or processing of food.
FAO/WHO 2014 [34]	Additive permitted for use in food in general	GMP	Food categories or individual food items excluded from the general conditions:<br>- Milk and buttermilk (excluding heat-treated milk)<br>- Fermented and renneted milk products (plain) excluding dairy based drinks)<br>- Pasteurized cream<br>- Sterilized, UHT, whipping or whipped, and reduced fat creams<br>- Whey cheese<br>- Whey protein cheese<br>- Dried whey and whey products, excluding whey cheese<br>- Fats and oils, essentially free from water<br>- Butter and concentrated butter<br>- Margarine<br>- Fresh fruit<br>- Fresh vegetables (including mushrooms and fungi, roots and tubers, pulses and legumes, and aloe vera), seaweeds, and nuts and seeds<br>- Frozen vegetables (including mushrooms and fungi, roots and tubers, pulses and legumes, and aloe vera), seaweeds, and nuts and seeds

Agency	Food	Value	Limitations/Exception
			- Fermented vegetables (including mushrooms and fungi, roots and tubers, pulses and legumes, and aloe vera) and seaweed products, excluding fermented soybean products<br>- Whole, broken or flaked grain, including rice<br>- Flours and starches<br>- Fresh pastas and noodles and like products<br>- Dried pastas and noodles and like products<br>- Fresh meat, poultry and game<br>- Fresh fish and fish products, including mollusks, crustaceans and echinoderms<br>- Processed fish and fish products, including mollusks, crustaceans and echinoderms<br>- Fresh eggs<br>- Liquid egg products<br>- Frozen egg products<br>- Refined and raw sugars<br>- Brown sugar, excluding soft white sugar, soft brown sugar, glucose syrup, dried glucose syrup raw cane sugar<br>- Sugar solutions and syrups, also (partially inverted, inclluding treacle and molasses, excluding soft white sugar, soft brown sugar, glucose syrup, dried glucose syrup raw cane sugar)<br>- Other sugars and syrups (e.g. xylose, maple syrup, sugar toppings)<br>- Honey<br>- Salt and salt substitutes<br>- Herbs and spices (only herbs)<br>- Infant formulae, follow-up formulae, and formulae for special medical purposes for infants<br>- Complementary food for infants and young children

Agency	Food	Value	Limitations/Exception
			- Waters<br>- Fruit and vegetable juices<br>- Fruit and vegetable nectars<br>- Coffee, coffee substitutes, tea, herbal infusions, and other hot cereal beverages, excluding cocoa<br>- Grape wines
IOFI (2012) [35]	Anticaking agent for flavourings	No data	
REACH (2017), Belgium [26]	Not classified in accord to Globally Harmonized System of Classification and Labelling of Chemicals	Oral exposure	Not classified.

Table 5: Regulatory decisions on talc use in cosmetic products

Agency	Condition of use
Health Canada (2015) [36]	Talc is included in the “List of Ingredients that are Restricted for Use in Cosmetic Products”<br>a) Products in powder form intended for infants and children<br>b) "Keep out of reach of children", "Keep powder away from child's face to avoid inhalation which can cause breathing problems."
European Union, 2009 [37]	Talc (powdery products intended to be used for children under 3 years of age) is restricted:<br>“Keep powder away from children’s nose and mouth”.

Table 6: Comparison of studies included in the meta-analysis of Berge et al. (2017), Penninkilampi et al. (2018) with studies included in the current review

Study	Berge et al 2017 [38]	Penninkilampi and Eslick (2018) [85]	Current Review
Cramer et al. (1982) [2]	✓	X	✓
Hartge et al. (1983) [3]	✓	✓	✓
Whittemore et al. (1988) [4]	✓	✓	✓
Booth et al. (1989) [5]	✓	✓	X
Harlow & Weiss (1989) [6]	✓	✓	✓
Chen et al. (1992) [7]	✓	✓	X
Harlow et al. (1992) [8]	✓	X	✓
Rosenblatt et al. (1992) [9]	✓	✓	✓
Tzonou et al. (1993) [10]	✓	✓	✓
Cramer et al. (1995) [39]	X	✓	X
Purdie et al. (1995) [40]	✓	✓	X
Shushan et al. (1996) [11]	X	✓	X
Chang & Risch (1997) [12]	✓	✓	✓
Cook et al. (1997) [13]	✓	✓	✓
Green et al. (1997) [41]	X	✓	✓
Godard et al. (1998) [15]	✓	✓	✓
Wong et al. (1999) [17]	✓	✓	✓
Ness et al. (2000) [18]	✓	✓	✓
Langseth and Kjærheim (2004)[42]	X	X	✓
Mills et al. (2004) [19]	✓	✓	✓
Gates et al. (2008) [43]	X	✓	✓
Goodman et al. (2008) [44]	✓	X	X
Merritt et al. (2008) [45]	✓	✓	✓
Moorman et al. (2009) [46]	✓	X	✓

Study	Berge et al 2017 [38]	Penninkilampi and Eslick (2018) [85]	Current Review
Wu et al. (2009) [47]	X	✓	✓
Rosenblatt et al. (2011) [48]	✓	✓	✓
Kurta et al. (2012) [49]	X	✓	✓
Lo-Ciganic et al. (2012) [50]	✓	X	X
Wu et al. (2015) [51]	✓	✓	✓
Cramer et al. (2016) [52]	✓	✓	✓
Schildkraut et al. (2016) [53]	✓	✓	✓
Gertig et al. (2000) [20]	X	✓	✓
Gates et al. (2010) [54]	✓	X	X
Houghton et al. (2014) [55]	✓	✓	✓
Gonzalez et al. (2016) [56]	✓	✓	✓

Table 7: Recent reports* on association of perineal exposure to talc and ovarian cancer

Review	No. of Studies			No. of Participants		Risk of Ovarian Cancer in talc Users (95% CI)
	Cohort	Case-Control	Total	Cases	Controls	
Langseth et al. (2008) [57]	1	20	21	NR**	NR	1.35 (1.26 to 1.46)
Terry et al. (2013) [58]	0	8	8	8,525	9,859	1.35 (1.26 to 1.46)
Penninkilampi and Eslick (2018) [59]	3	24	27	14,311	19,314	1.31(1.24 to 1.39)
Huncharek et al. (2003) [60]	1	15	16	5,260	6,733	1.33 (1.16 to 1.45)
**Current Review (2019)**	**3**	**24**	**27**	**16,352**	**19,808**	**1.28 (1.20 to 1.37)**
Berge et al. (2017) [38]	3	24	27	15,019	18,851	1.22 (1.13 to 1.30)

** Arranged in a descending order from highest to lowest reported risk*

*** NR: Not Reported*

Supplementary Material II: Literature review – human studies

II.1. Review strategy

In conducting our review, we established a comprehensive, multi-step search strategy (Figure 1) to identify relevant studies on talc from multiple bibliographic databases, relevant national and international agencies and other grey literature sources. Potentially relevant studies were then each screened independently by two reviewers (MT and NF) for inclusion in our review, based on predetermined eligibility criteria. The two reviewers then individually examined each study selected for inclusion, extracted key characteristics and detailed findings and assessed its quality based on the Newcastle-Ottawa scale (NOS) [61].

Discrepancies were resolved by consensus or via a panel comprised of three senior scientists. Qualitative findings from human studies were evaluated along with relevant findings from non-human studies in assessing the evidence for a causal association between perineal talc exposure and ovarian cancer.

Consistent with the literature and available data, a meta-analysis was conducted using standard approaches for combining data from multiple epidemiological studies. Subgroup analyses included the histologic subtypes, duration and frequency of use, method of perineal talc powder application, menopausal status of study participants, as well as other factors. In combining data from multiple studies using meta analysis, the exposure metrics (total applications, frequency of use, and years of use) used in the original studies were used. Exposure-response relationships based on data from these studies were also assessed.

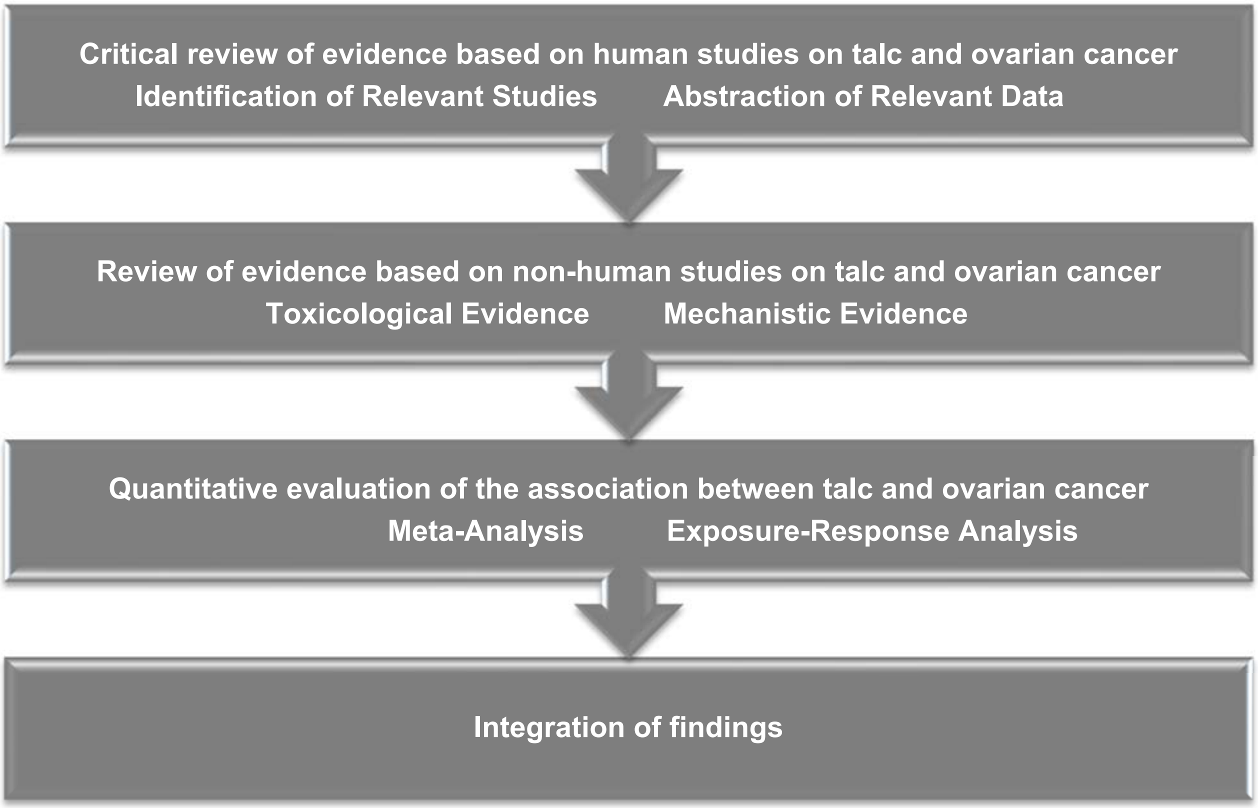

Figure 1. Steps involved in evaluation of the association between perineal use of talc and ovarian cancer risk

II.2. Systematic literature search and identification of relevant studies

We conducted a systematic search for all original studies involving human subjects that examined the association of the genital/perineal use of talc powder and the risk of ovarian cancer. Our approach to this systematic review followed the PRISMA guidelines, and more specific guidance provided by the Cochrane Collaboration [62]. This approach began with the development of the search strategy, which laid out the search terms to be used and the databases to be searched in order to identify literature relevant to the evaluation of the genital/perineal use of talc and risk of ovarian cancer.

The search strategy (Supplementary Material II) was implemented between April 8 and April 12, 2017, and updated on September 24 - 26, 2018. The search examined multiple databases for relevant studies without applying any language, time or other filters to limit the search output. The electronic databases searched included EMBASE, Medline, Cochrane, PubMed, CINAHL, WHO Clinical Trials Registry (ICTRN), US Clinical Trials Database, UK Clinical Trials Gateway, and grey literature sources.

Identified references from all sources were collated using the EndNote reference management application. EndNote was used to identify potential duplicates, with manual resolution employed to remove actual duplicates. Duplicate screening of titles and abstracts (Stage 1) and full-text (Stage 2) was performed (MT, NF, NS and NK) to identify studies eligible for inclusion in the review. The review was completed through a multi-level assessment process, using the Distiller SR software [63]. Screening forms and criteria used for assessment and data abstraction are presented in Supplementary Material III. Data extracted from the retained studies via Distiller SR were exported independently to Microsoft Excel spreadsheets, and then compared by the two

reviewers (MT and NF). Duplicate screening was done on all studies, along with duplicate quality assessment and data abstraction on a 30% sample of included studies. Conflicts identified in each step were resolved via consensus between the reviewers or via a panel of three senior scientists, prior to moving to the next step. A summary and analysis of the extracted data are presented in the results section of this review.

In conducting this review, we examined Berge et al. [38] to identify relevant epidemiological studies for evaluation. This recent systematic review identified 24 case–control studies and 3 cohort studies, including 16,005 cases of ovarian cancer and 201,881 controls. The 27 epidemiological studies represented twenty studies conducted in the US, two in Australia, two in Canada, one in Great Britain, one in Norway, one in China, and one in Greece. These studies were also identified in our systematic review, and were individually evaluated and scored by two reviewers (MT and NF) as detailed in the next section (see Table 6 & Supplementary Material XI), which identified some differences in the studies included in our review compared to Berge et al. [38].

Compared to the 27 studies included in the meta-analysis by Berge et al. [38], our meta-analysis excluded 6 of the studies analyzed by Berge et al. [38] (including 5 case-control and 1 cohort studies), and included 6 additional studies (5 case-control and 1 cohort studies) that were not captured in the meta-analysis by Berge et al. [38].

In five of these excluded studies [5, 7, 40, 44, 50], we were unable to identify a risk estimate for talc use from the original studies, or the reported risk estimate was inconsistent with the reference groups of other studies. We also excluded the study by Gates et al. [54] in favor of an earlier study by Gertig et al. [20] that targeted the same cohort, since that the newer study reports effect estimates for a reference group (< 1

use talc/week) that was inconsistent with the reference groups of other studies (never use of talc). While both studies were included in the systematic review, only Gertig et al. [20] was included in the meta-analysis.

Another recent review by Penninkilampi et al in 2018 [85] captured 24 case-control studies and 3 cohort studies, with some differences in their inclusion/exclusion criteria for selection of studies. Table 6 lists the studies included and excluded in our meta-analyses, compared to that of Berge et al. [38] and Penninkilampi et al [85].

II.3. Evaluation of quality of retained human studies

The relevance, reliability and adequacy of each included study were critically assessed using appropriate quality assessment procedures. The quality of non-randomized studies included in the review was assessed using the NOS [61], as recommended in the Cochrane Handbook [62]. Results of our NOS evaluation of study quality can be found in Supplementary Material IV. Systematic reviews using the NOS for quality assessment have set different cut-off points to classify study quality: in this review, we used a cut-off point of 7+ stars to represent studies of higher quality.

II.4. Database search strategy

EMBASE[2]

Concept	#	EMBASE Query	Retrieved
Exposure	1	talc/	4,565
	2	talc*.tw.	3,814
	3	talc* powder.tw.	260
	4	perineal powder.tw.	7
	5	genital powder.tw.	6
	6	baby powder.tw.	57
	7	sclerosol.tw.	7
	8	or/1-7	5,709
Outcome	9	exp ovary cancer/	101,640
	10	(cancer* adj3 ovar*).tw.	68,509
	11	exp ovary carcinoma/	28,881
	12	(carcino* adj3 ovar*).tw.	23,509
	13	(malignan* adj3 ovar*).tw.	7,592
	14	exp ovary tumor/	126,728
	15	(tumor* adj3 ovar*).tw.	22,107
	16	(tumour* adj3 ovar*).tw.	4,227
	17	(neoplasm* adj3 ovar*).tw.	2,833
	18	granulosa cell tumor/	3,018
	19	granulosa cell tumor.tw.	1,231
	20	luteoma/	183
	21	luteoma.tw.	195
	22	meigs syndrome/	119
	23	meigs syndrome.mp.	561
	24	meigs syndrome.tw.	527
	25	demons meigs syndrome.tw.	57
	26	sertoli-leydig cell tumor/	800

[2] Embase: Excerpta Medica Database Guide (Embase Classic + Embase 1947 to 2017 May 02)

Concept	#	EMBASE Query	Retrieved
	27	sertoli-leydig cell tumor.tw.	287
	28	thecoma/	920
	29	thecoma.tw.	319
	30	teratoma/	26,119
	31	teratoma.tw.	13,459
	32	dysgerminoma/	3,599
	33	dysgerminoma.tw.	1,368
	34	androblastoma/	983
	35	androblastoma.tw.	92
	36	or/9-35	164,670
Combined	37	8 and 36	196

Medline Ovid[3]

Concept	#	Medline Query	Retrieved
Exposure	1	Talc/	1,993
	2	talc*.tw.	2,750
	3	talc* powder.tw.	198
	4	perineal powder.tw.	6
	5	genital powder.tw.	5
	6	baby powder.tw.	54
	7	sclerosol.tw.	1
	8	or/1-7	3,303
Outcome	9	exp Ovarian Neoplasms/	76,139
	10	(neoplas* adj3 ovar*).tw.	2,883
	11	(cancer* adj3 ovar*).tw.	48,828
	12	(carcino* adj3 ovar*).tw.	18,426
	13	(malignan* adj3 ovar*).tw.	5,736
	14	(tumor* adj3 ovar*).tw.	17,398
	15	(tumour* adj3 ovar*).tw.	3,280
	16	(tumor* adj3 ovar*).tw.	17,398
	17	(malignan* adj3 ovar*).tw.	5,736
	18	(reproduct* adj3 cancer*).tw.	1,197
	19	(reproduct* adj3 carcino*).tw.	298
	20	(reproduct* adj3 tumor*).tw.	344
	21	(reproduct* adj3 tumour*).tw.	59
	22	Struma Ovarii/	518
	23	struma ovarii.tw.	509
	24	Granulosa Cell Tumor/	2,214
	25	granulosa cell tumor.tw.	1,052

[3] *MEDLINE(R) Epub Ahead of Print, In-Process & Other Non-Indexed Citations, Ovid MEDLINE(R) Daily and Ovid MEDLINE(R) 1946 to Present*

Concept	#	Medline Query	Retrieved
	26	(Hereditary Breast and Ovarian Cancer Syndrome).tw.	184
	27	Luteoma/	84
	28	luteoma.tw.	170
	29	Meigs Syndrome/	561
	30	meigs syndrome.tw.	554
	31	Sertoli-Leydig Cell Tumor/	782
	32	sertoli-leydig cell tumor.tw.	228
	33	Thecoma/	917
	34	thecoma.tw.	317
	35	or/9-34	98,708
Combined	36	8 and 35	136

PubMed

**Concept**	**#**	**PubMed Query**	Retrieved
	1	talc [tw]	2,910
	2	talcum	3,042
	3	talcum [tw]	294
	4	talcum powder	2,982
	5	talcum powder [tw]	147
	6	talc* powder [tw]	382
	7	perineal powder	28
	8	"perineal powder" [tw]	6
	9	genital powder	35
	10	"genital powder" [tw]	5
	11	baby powder	1,184
	12	"baby powder" [tw]	51
	13	((((((((((talc [tw]) OR talcum) OR talcum [tw]) OR talcum powder) OR talcum powder [tw]) OR talc* powder [tw]) OR perineal powder) OR "perineal powder" [tw]) OR genital powder) OR "genital powder" [tw]) OR baby powder) OR "baby powder" [tw]	1,550
	14	ovarian neoplasm	89,072
	15	ovarian neoplasm [tw]	555
	16	ovarian neoplasms	88,113
	17	ovarian neoplasms [tw]	70,791
	18	ovarian neoplasia	88,287
	19	ovarian neoplasia [tw]	181
	20	ovarian cancer	99,677
	21	ovarian cancer [tw]	42,576
	22	cancer of the ovary	95,226
	23	cancer of the ovary [tw]	486
	24	ovarian carcinoma	32,460
	25	ovarian carcinoma [tw]	11,837
	26	carcinoma of the ovary	9,449
	27	carcinoma of the ovary [tw]	1,293

Concept	#	PubMed Query	Retrieved
	28	ovarian carcinogenesis	3,212
	29	ovarian carcinogenesis [tw]	547
	30	ovarian malignancy	96,720
	31	ovarian malignancy [tw]	861
	32	ovarian tumor	95,286
	33	ovarian tumor [tw]	4,791
	34	ovarian tumors	91,012
	35	ovarian tumors [tw]	6,864
	36	ovarian tumour	89,099
	37	ovarian tumour [tw]	862
	38	ovarian tumours	88,604
	39	ovarian tumours [tw]	1,367
	40	reproductive cancer	91,336
	41	reproductive cancer [tw]	68
	42	carcinoma ovarii	273
	43	carcinoma ovarii [tw]	2
	44	struma ovarii	631
	45	struma ovarii [tw]	631
	46	clear cell carcinoma of the ovary	869
	47	clear cell carcinoma of the ovary [tw]	201
	48	small cell carcinoma of the ovary hypercalcemic type	73
	49	small cell carcinoma of the ovary hypercalcemic type [tw]	27
	50	mucinous carcinoma of the ovary	1,152
	51	mucinous carcinoma of the ovary [tw]	28
	52	low-grade serous carcinoma of the ovary	168
	53	low-grade serous carcinoma of the ovary [tw]	41
	54	granulosa cell tumor	3,212
	55	granulosa cell tumor [tw]	2,439
	56	granulosa cell tumors	3,353

Concept	#	PubMed Query	Retrieved
	57	granulosa cell tumors [tw]	810
	58	granulosa cell tumour	3,212
	59	granulosa cell tumour [tw]	222
	60	granulosa cell tumours	3,353
	61	granulosa cell tumours [tw]	168
	62	hereditary breast and ovarian cancer syndrome	596
	63	hereditary breast and ovarian cancer syndrome [tw]	246
	64	luteoma	193
	65	luteoma [tw]	193
	66	Meigs Syndrome	675
	67	Meigs Syndrome [tw]	607
	68	sertoli-leydig cell tumor	3,418
	69	sertoli-leydig cell tumor [tw]	892
	70	sertoli-leydig cell tumors	3,357
	71	sertoli-leydig cell tumors [tw]	181
	72	sertoli-leydig cell tumour	3,418
	73	sertoli-leydig cell tumour [tw]	51
	74	sertoli-leydig cell tumours	1,031
	75	sertoli-leydig cell tumours [tw]	27
	76	thecoma	993
	77	thecoma [tw]	993
	78	(((ovarian neoplasm) OR ovarian neoplasm [tw]) OR ovarian neoplasms) OR ovarian neoplasms [tw]) OR ovarian neoplasia) OR ovarian neoplasia [tw]) OR ovarian cancer) OR ovarian cancer [tw]) OR cancer of the ovary) OR cancer of the ovary [tw]) OR ovarian carcinoma) OR ovarian carcinoma [tw]) OR carcinoma of the ovary) OR carcinoma of the ovary [tw]) OR ovarian carcinogenesis) OR ovarian carcinogenesis [tw]) OR ovarian malignancy) OR ovarian malignancy [tw]) OR ovarian tumor) OR ovarian tumor [tw]) OR ovarian tumors) OR ovarian tumors [tw]) OR ovarian tumour) OR ovarian	199,501

Concept	#	PubMed Query	Retrieved
		tumour [tw]) OR ovarian tumours) OR ovarian tumours [tw]) OR reproductive cancer) OR reproductive cancer [tw]) OR carcinoma ovarii) OR carcinoma ovarii [tw]) OR struma ovarii) OR struma ovarii [tw]) OR clear cell carcinoma of the ovary) OR clear cell carcinoma of the ovary [tw]) OR small cell carcinoma of the ovary hypercalcemic type) OR small cell carcinoma of the ovary hypercalcemic type [tw]) OR mucinous carcinoma of the ovary) OR mucinous carcinoma of the ovary [tw]) OR low-grade serous carcinoma of the ovary) OR low-grade serous carcinoma of the ovary [tw]) OR granulosa cell tumor) OR granulosa cell tumor [tw]) OR granulosa cell tumors) OR granulosa cell tumors [tw]) OR granulosa cell tumour) OR granulosa cell tumour [tw]) OR granulosa cell tumours) OR granulosa cell tumours [tw]) OR (hereditary breast and ovarian cancer syndrome)) OR (hereditary breast and ovarian cancer syndrome [tw])) OR luteoma) OR luteoma [tw]) OR Meigs Syndrome) OR Meigs Syndrome [tw]) OR sertoli-leydig cell tumor) OR sertoli-leydig cell tumor [tw]) OR sertoli-leydig cell tumors) OR sertoli-leydig cell tumors [tw]) OR sertoli-leydig cell tumour) OR sertoli-leydig cell tumour [tw]) OR sertoli-leydig cell tumours) OR sertoli-leydig cell tumours [tw]) OR thecoma) OR thecoma [tw]	
	79	((((((((((((talc [tw]) OR talcum) OR talcum [tw]) OR talcum powder) OR talcum powder [tw]) OR talc* powder [tw]) OR perineal powder) OR "perineal powder" [tw]) OR genital powder) OR "genital powder" [tw]) OR baby powder) OR "baby powder" [tw])) AND (((ovarian neoplasm) OR ovarian neoplasm [tw]) OR ovarian neoplasms) OR ovarian neoplasms [tw]) OR ovarian neoplasia) OR ovarian neoplasia [tw]) OR ovarian cancer) OR ovarian cancer [tw]) OR cancer of the ovary) OR cancer of the ovary [tw]) OR ovarian carcinoma) OR ovarian carcinoma [tw]) OR carcinoma of the ovary) OR carcinoma of the ovary [tw]) OR ovarian carcinogenesis) OR ovarian carcinogenesis [tw]) OR ovarian malignancy) OR ovarian malignancy [tw]) OR ovarian tumor) OR	40

Concept	#	PubMed Query	Retrieved
		ovarian tumor [tw]) OR ovarian tumors) OR ovarian tumors [tw]) OR ovarian tumour) OR ovarian tumour [tw]) OR ovarian tumours) OR ovarian tumours [tw]) OR reproductive cancer) OR reproductive cancer [tw]) OR carcinoma ovarii) OR carcinoma ovarii [tw]) OR struma ovarii) OR struma ovarii [tw]) OR clear cell carcinoma of the ovary) OR clear cell carcinoma of the ovary [tw]) OR small cell carcinoma of the ovary hypercalcemic type) OR small cell carcinoma of the ovary hypercalcemic type [tw]) OR mucinous carcinoma of the ovary) OR mucinous carcinoma of the ovary [tw]) OR low-grade serous carcinoma of the ovary) OR low-grade serous carcinoma of the ovary [tw]) OR granulosa cell tumor) OR granulosa cell tumor [tw]) OR granulosa cell tumors) OR granulosa cell tumors [tw]) OR granulosa cell tumour) OR granulosa cell tumour [tw]) OR granulosa cell tumours) OR granulosa cell tumours [tw]) OR (hereditary breast and ovarian cancer syndrome)) OR (hereditary breast and ovarian cancer syndrome [tw])) OR luteoma) OR luteoma [tw]) OR Meigs Syndrome) OR Meigs Syndrome [tw]) OR sertoli-leydig cell tumor) OR sertoli-leydig cell tumor [tw]) OR sertoli-leydig cell tumors) OR sertoli-leydig cell tumors [tw]) OR sertoli-leydig cell tumour) OR sertoli-leydig cell tumour [tw]) OR sertoli-leydig cell tumours) OR sertoli-leydig cell tumours [tw]) OR thecoma) OR thecoma [tw]	

Cochrane Library (Wiley)

Concept	#	Cochrane query	Retrieved
	#1	talc and "ovar* cancer*"	5
	#2	talc and "ovar* tumour*"	1
	#3	talc and "ovar* tumor*"	1
	#4	MeSH descriptor: [Talc] explode all trees	56
	#5	MeSH descriptor: [Ovarian Neoplasms] explode all trees	1,524
	#6	#4 and #5	1
	#7	#1 or #2 or #3 or #6	6

CINAHL[4]

Concept	#	CINAHL query	
Outcome	**S1**	(MH "Ovarian Neoplasms+")	5,758
	**S2**	TX ovar* N3 neoplasm*	5,732
	**S3**	TX ovar* N3 cancer*	4,619
	**S4**	TX ovar* N3 carcino*	505
	**S5**	TX ovar* N3 tumor*	406
	**S6**	TX ovar* N3 tumour*	125
	**S7**	TX reproductive N3 cancer*	285
	**S8**	TX reproductive N3 tumor*	10
	**S9**	TX reproductive N3 tumour*	4
	**S10**	TX reproductive N3 carcino*	29
Exposure	**S11**	MH talc	122
	**S12**	TX "talc"	185
	**S13**	TX "baby powder"	8
	**S14**	TX "genital powder"	1
	**S15**	TX "perineal powder"	1
	**S16**	TX sclerosol	1
	**S17**	S11 OR S12 OR S13 OR S14 OR S15 OR S16	191
	**S18**	S1 OR S2 OR S3 OR S4 OR S5 OR S6 OR S7 OR S8 OR S9 OR S10	7,517
	**S19**	**S17 AND S18**	17

[4] Cumulative Index to Nursing and Allied Health Literature

WHO Clinical Trials Registry

Reference #	Reference	Remarks
ISRCTN12709516	Debulking Surgery and Hyperthermic Chemotherapy for pleural mesothelioma: a pilot study.	
NCT02825095	Management of Malignant Pleural Effusion - Indwelling Pleural Catheter or Talc Pleurodesis	Not recruiting
JPRN-UMIN000021408	A randomized comparative phase 3 trial of pleurodesis in malignant pleural effusions: sterile graded talc vs. OK-432 (WJOG8415L)	Recruiting
ISRCTN16441661	The efficacy of sonographic and biological pleurodesis indicators of malignant pleural effusion (SIMPLE)	Recruiting
ISRCTN15503522	A trial looking at quality of life in the treatment of patients with malignant pleural effusion	Recruiting
EUCTR2012-000599-40-GB	A randomised controlled trial to determine whether the use of indwelling pleural catheters in conjunction with talc slurry is superior to using an indwelling pleural catheter alone, in patients with malignant pleural effusion.	Authorised
ISRCTN47845793	A randomised trial to determine the best method for delivering talc for the management of malignant pleural effusions in patients with a good performance status	Recruiting
NCT01409551	Video-assisted Hyperthermic Pleural Chemoperfusion vs Talc Pleurodesis for Refractory Malignant Pleural Effusions.	Not recruiting
CTRI/2010/091/001247	A clinical trial to evaluate the quality of life with AYUSH QOL 2C in patients of Non metastatic breast cancer as an adjuvant to chemotherapy	Recruiting
ISRCTN12852177	A randomised controlled trial to evaluate whether use of intrapleural urokinase aids the drainage of multi-	Not Recruiting

Reference #	Reference	Remarks
	septated pleural effusion compared to placebo	
ISRCTN59686582	Talc Pleurodesis: A Comparative Pilot Study Comparing Doctor vs Nurse Lead Procedure	Not Recruiting
ISRCTN34321019	Prospective randomised controlled trial of video assisted cytoreductive pleurectomy compared to talc pleurodesis in patients with suspected or proven mesothelioma	Not Recruiting
ISRCTN35591640	A study to compare the efficiency of sterile talc, tetracycline and bleomycin as sclerosing agents for medical pleurodesis in the treatment of malignant pleural effusions	Not Recruiting
NCT00042770	Standard Chest Tube Compared With a Small Catheter in Treating Malignant Pleural Effusion in Patients With Cancer	Not recruiting
NCT00002872	Bleomycin, Doxycycline, or Talc in Treating Patients With Malignant Pleural Effusions	Not recruiting
NCT00002622	Talc in Treating Patients With Malignant Pleural Effusion	Not recruiting
ISRCTN12709516	Debulking Surgery and Hyperthermic Chemotherapy for pleural mesothelioma: a pilot study.	
NCT02825095	Management of Malignant Pleural Effusion - Indwelling Pleural Catheter or Talc Pleurodesis	Not recruiting
JPRN-UMIN000021408	A randomized comparative phase 3 trial of pleurodesis in malignant pleural effusions: sterile graded talc vs. OK-432 (WJOG8415L)	Recruiting
ISRCTN16441661	The efficacy of sonographic and biological pleurodesis indicators of malignant pleural effusion (SIMPLE)	Recruiting

Reference #	Reference	Remarks
ISRCTN15503522	A trial looking at quality of life in the treatment of patients with malignant pleural effusion	Recruiting
EUCTR2012-000599-40-GB	A randomised controlled trial to determine whether the use of indwelling pleural catheters in conjunction with talc slurry is superior to using an indwelling pleural catheter alone, in patients with malignant pleural effusion.	Authorised
ISRCTN47845793	A randomised trial to determine the best method for delivering talc for the management of malignant pleural effusions in patients with a good performance status	Recruiting
NCT01409551	Video-assisted Hyperthermic Pleural Chemoperfusion vs Talc Pleurodesis for Refractory Malignant Pleural Effusions.	Not recruiting
CTRI/2010/091/001247	A clinical trial to evaluate the quality of life with AYUSH QOL 2C in patients of Non metastatic breast cancer as an adjuvant to chemotherapy	Recruiting

US Clinical Trials Database

#	CT number	Title	Status	Results
1	NCT00042770	Standard Chest Tube Compared With a Small Catheter in Treating Malignant Pleural Effusion in Patients With Cancer	Completed	No Results Available
2	NCT00002872	Bleomycin, Doxycycline, or Talc in Treating Patients With Malignant Pleural Effusions	Completed	No Results Available
3	NCT00002622	Talc in Treating Patients With Malignant Pleural Effusion	Completed	No Results Available
4	NCT01409551	Video-assisted Hyperthermic Pleural Chemoperfusion vs Talc Pleurodesis for Refractory Malignant Pleural Effusions.	Completed	No Results Available
5	NCT00821860	Video-Assisted Surgery or Talc Pleurodesis in Treating Patients With Malignant Mesothelioma	Completed	No Results Available
6	NCT02825095	Management of Malignant Pleural Effusion - Indwelling Pleural Catheter or Talc Pleurodesis	Not yet recruiting	No Results Available
7	NCT02511600	Comparison of Progel Sealant to Standard of Care (SOC) for Patients Undergoing Decortication	Withdrawn	No Results Available
8	NCT02045121	Multicentre Study Comparing Indwelling Pleural Catheter With Talc Pleurodesis for Malignant Pleural Effusion Management	Unknown status	No Results Available
9	NCT00430664	A Comparative Study of the Safety and Efficacy of Face Talc Slurry and Iodopovidone for Pleurodesis	Unknown status	No Results Available
10	NCT01469728	Comparison of Thoracoscopic Talc Pleurodesis by Thoracic Epidural or General Anesthesia	Completed	No Results Available
11	NCT02674243	Efficacy of Iodopovidone Versus Talc in Palliative Malignant Pleural Effusion	Recruiting	No Results Available
12	NCT02805062	Manometry vs Clinical Assessment in the Detection of Trapped Lung in	Not yet recruiting	No Results Available

#	CT number	Title	Status	Results
		Patients With Suspected Pleural Malignancy		
13	NCT00896285	The First Therapeutic Intervention in Malignant Pleural Effusion Trial	Completed	No Results Available
14	NCT00758316	A Prospective, Randomized Controlled Trial for a Rapid Pleurodesis Protocol for the Management of Pleural Effusions	Completed	No Results Available
15	NCT02519049	11C-Methionine PET/CT Imaging in Patients Affected by Malignant Pleural Mesothelioma (MPM)	Recruiting	No Results Available
16	NCT02583282	A Study to Compare the Efficacy and Safety of Intrapleural Doxycycline Versus Iodopovidone for Performing Pleurodesis in Malignant Pleural Effusion	Recruiting	No Results Available
17	NCT00789087	Talc Pleurodesis in Patients With Recurrent Malignant Pleural Effusion	Completed	No Results Available
18	NCT00644319	Ibuprofen or Morphine in Treating Pain in Patients Undergoing Pleurodesis for Malignant Pleural Effusion	Unknown status	No Results Available
19	NCT02517749	Out Patient Talc Slurry Via Indwelling Pleural Catheter for Malignant Pleural Effusion Vs Usual Inpatient Management	Recruiting	No Results Available
20	NCT00637676	Tunneled Pleural Catheter in Partially Entrapped Lung	Completed	No Results Available
21	NCT02625675	Using Thoracic Ultrasound to Predict Pleurodesis Success in Malignant Pleural Effusions: a Pilot Study	Recruiting	No Results Available
22	NCT02127021	The Effects of High Dose Pancreatic Enzyme Replacement Therapy After Pancreatoduodenectomy	Recruiting	No Results Available

UK Clinical Trials Gateway

#	Reference
1.	Ibuprofen or Morphine in Treating Pain in Patients Undergoing Pleurodesis for Malignant Pleural Effusion
2.	The first Therapeutic Interventions in Malignant Effusion trial
3.	SSAT067 PK of Atazanavir/Cobicistat and Darunavir/Cobicistat
4.	Pharmacokinetic Effect of Evotaz/Microgynon Co-administration
5.	REPAIR: Right vEntricular Remodeling in Pulmonary ArterIal hypeRtension
6.	SSAT061: PK of DTG and EVT/COBI in Healthy Volunteers
7.	Efavirenz to Dolutegravir Switch in Patients With CNS Toxicity
8.	Bortezomib in Treating Patients With Malignant Pleural Mesothelioma
9.	Efficacy, Pharmacokinetics, and Safety of Presatovir in Hospitalized Adults With Respiratory Syncytial Virus (RSV) Infection
10.	Study to Assess the Efficacy and Safety of Raxone in LHON Patients
11.	A Multicentre Study to Evaluate the Safety and Efficacy of AD 923 in Comparison to MSIR for the Treatment of CBP in Subjects
12.	Rifaximin for Preventing Relapse of Clostridium Associated Diarrhoea
13.	Examining the effect of intravenous zoledronic acid on pleural fluid production, breathlessness and quality of life in patients with a malignant pleural effusion
14.	Using Ultrasound to Predict the Results of Draining Pleural Effusions
15.	Prospective blinded randomised placebo controlled trial investigating whether oxycodone modified release reduces parenteral opioid use following intermediate thoracic surgery
16.	A randomised controlled trial to evaluate whether use of intrapleural urokinase aids the drainage of multi-septated pleural effusion compared to placebo
17.	A trial looking at quality of life in the treatment of patients with malignant pleural effusion
18.	A randomised trial to determine the best method for delivering talc for the management of malignant pleural effusions in patients with a good performance status
19.	A study to compare the efficiency of sterile talc, tetracycline and bleomycin as sclerosing agents for medical pleurodesis in the treatment of malignant pleural effusions
20.	Prospective randomised controlled trial of video assisted cytoreductive pleurectomy compared to talc pleurodesis in patients with suspected or proven mesothelioma

21. Talc Pleurodesis: A Comparative Pilot Study Comparing Doctor vs Nurse Lead Procedure
22. Video-Assisted Surgery or Talc Pleurodesis in Treating Patients With Malignant Mesothelioma
23. Using Thoracic Ultrasound to Predict Pleurodesis Success in Malignant Pleural Effusions: a Pilot Study.
24. The efficacy of sonographic and biological pleurodesis indicators of malignant pleural effusion (SIMPLE).
25. The Efficacy of Sonographic and Biological Pleurodesis Indicators of Malignant Pleural Effusion (SIMPLE) - a randomised trial.
26. Randomised controlled trial comparing outpatient management of malignant pleural effusion via an indwelling pleural catheter and talc pleurodesis versus standard inpatient management in improving health related quality of life.
27. Out Patient Talc Slurry Via Indwelling Pleural Catheter for Malignant Pleural Effusion Vs Usual Inpatient Management.
28. The efficacy of Indwelling Pleural Catheter placement versus IPC placement PLUS sclerosant (talc) in patients with malignant pleural effusions managed exclusively as out-patients.
29. Evaluating the efficacy of thoracoscopy and talc poudrage versus pleurodesis using talc slurry: a randomised trial to determine the most effective method for the management of malignant pleural effusions in patients with a good performance status.

Grey Literature

Source	# or Retrieved References
NIOSHTIC	357
CISILO	75
CAB Direct	22
OSHLINE	17
Canadiana	5
CADTH	1
Total	477

II.5. Database search results

The search strategy yielded 196 references from EMBASE, 136 from Medline, 2 from Cochrane Library, 240 from PubMed, 17 from CINAHL, 14 from WHO-ICTRN, 22 ClinicalTrials.gov, and 29 UK Clinical Trials Gateway, for an initial total of 656 references (including duplicates). Although the grey literature search yielded another 477 references, reviewers concurred to including only the five peer-reviewed references, in addition to the 656 peer-reviewed references that were retrieved from searching the other databases. After automatic removal of duplicates, 326 references remained for further consideration. Upon manual removal of duplicates, 282 references were available for screening and review.

First level screening of titles and abstracts resulted in the exclusion of 52 studies. Full-text examination of the remaining 230 references resulted in the exclusion of 193 references with reasons, and in the inclusion of 37 studies for abstraction of relevant data (Supplementary Materials X and XI). Seven studies were subsequently excluded due to reporting on overlapping populations, leaving 30 studies for further qualitative / quantitative analyses. A detailed PRISMA flow diagram is shown in Figure 2 [64].

The thirty observational studies that met the inclusion criteria for the systematic review included 26 case-control studies and four prospective cohort studies. Although two cohort studies by Gertig et al. 2000 [20] and Gates et al. 2010 [54] analyzed overlapping populations, they were both included since they reported on complementary findings. Key characteristics of these studies are summarized in Table 8.

Twenty-one of the thirty studies were carried out in the USA, with the remaining studies conducted in Europe (n=4), Canada (n=2), Australia (n=2) and China (n=1).

Forty percent (n=12) of the studies were relatively recent, published in the last decade, with the remaining studies published between 1982 and 2006. The study populations generally included adult women. Several studies analyzed data from populations initially recruited for other purposes, such as the Nurses' Health Study (NHS) [20, 52, 54] and Women's Health Initiative (WHI) [55].

The number of ovarian cancer patients analyzed varied considerably from 46 cases [42] to 22,041 cases [52]. Twenty-seven out of the 30 included studies assessed the association between ever use of perineal talc use and ovarian cancer. Subgroup analyses examining the effect of frequency and duration of use, type of use, period of use and other factors varied among these studies (see Table 2 in the main manuscript).

Overall conclusions on the association between perineal talc exposure and ovarian cancer risk were mixed. Sixty three percent (n=19) of the studies concluded the presence of a positive association between perineal exposure to talc powder and ovarian cancer risk [2, 5, 7-9, 12, 13, 18, 19, 41, 43, 45, 47-49, 51-54]. Ten studies concluded the absence of an association [3, 6, 10, 15, 17, 20, 42, 46, 55, 56]. Only one study could not reach a solid conclusion on the presence or absence of an association [4]. Many of the 30 included studies reported variability in some of the analyzed subgroups regarding possible association between exposure to talk powder and risk of ovarian cancer. Supplementary Material X presents the findings and details of all the studies included in the analysis, while Supplementary Material XI summarizes the strengths and limitations of each of these studies as identified by the original study authors and by us.

Supplementary Material III: Distiller SR forms for screening and data abstraction - human studies

Level 1 (title and abstract screening)

1. **Is this study related to Talc?**
   (including CAS RN 14807-96-6, sclerosol, baby powder, perineal powder, genital powder, talcum powder etc)
   - ○ Yes
   - ○ No
   - ○ Not Sure

2. **Is this study related to ovarian cancer?**
   - ○ Yes
   - ○ No
   - ○ Not Sure

3. **Does this study involve human subjects?**
   - ○ Yes
   - ○ No
   - ○ Not Sure

Level 2 (full-text screening)

1. **Is this study related to Talc?**
   (including CAS RN 14807-96-6, sclerosol, baby powder, perineal powder, genital powder, talcum powder etc)
   - O Yes
   - O No

2. **What is the route of exposure?**
   - ☐ Genital/Perineal
   - ☐ Dermal
   - ☐ Inhalational
   - ☐ Oral
   - ☐ Others (eg: injections)

3. **Is this study related to ovarian cancer?**
   - O Yes
   - O No
   - O Other Types of Cancer?

4. **Does this study involve human subjects?**
   - O Yes
   - O No

5. **What is the study type?**
   - O Original Study
   - O Systematic Review
   - O Systematic Review and Meta-Analysis
   - O Meta-Analysis without Systematic Review
   - O Editorial/Commentary with no primary data
   - O Literature Review
   - O Other

6. **Is the full-article PDF available?**
   - O Yes
   - O No

7. **Comments:**

Level 3 (data abstraction)

I. REFERENCES & NOTES TO EXTRACTOR

In Text Citation Format

[ ]

II. FUNDING

Funding Source

- ☐ Not Reported
- ☐ Other [ ]
- ☐ Other [ ]
- ☐ Other [ ]

Conflict of Interest

Select an Answer
Not Reported
Authors report NO COIs
Authors report COIs

III. STUDY POPULATION AND DEMOGRAPHICS

Country

[ ]

Region

[ ]

Race/Ethnicity (as reported):

- ☐ Not Reported
- ☐ American Indian or Alaska Native
- ☐ Black or African American
- ☐ Native Hawaiian or Other Pacific Islander
- ☐ "other" [ ]
- ☐ Hispanic
- ☐ Asian
- ☐ White

Cohort Name (If applicable)

Age

☐ No age data reported
☐ Age Range
☐ Mean ± SD
☐ Median

Sex

Select an Answer
Male
Female
Both
Not reported

Inclusion Criteria:

Exclusion criteria:

IV. Methodology

Study design

- Select an Answer
- cohort, prospective
- cohort, retrospective
- cross-sectional
- case-control
- case-control, nested
- case report
- case series
- other

Sample size

☐ For cohort studies: cases/final cohort size. - TOTAL
☐ For cohort studies: cases/final cohort size. - ANALYZED
☐ For case-control studies: cases/controls - TOTAL
☐ For case-control studies: cases/controls - ANALYZED
☐ Other

Calendar Years of Sampling

Average length of follow-up (cohort studies)

Response rate (%)

V. Exposure

Type of Talc powder

- **Select an Answer**
- Talc, containing asbestos
- Talc, not containing asbestos
- Talc, other impurity
- Talc, not specified

Route of Exposure

☐ Perineal/Genital
☐ Dermal
☐ Inhalational
☐ Oral

Source

☐ Cosmetic
☐ Occupational
☐ Environmental
☐ Other

Method of Exposure Ascertainment

☐ Self-Reported Use ☐ Blood ☐ Serum ☐ Plasma ☐ Urine
☐ Hair ☐ Skin ☐ Medical Records ☐ Other

Duration of exposure (e.g. mean or range)

Categorization of exposure (e.g. dichotomous, quartiles, never vs. ever)

VI. Outcome:

Ovarian cancer subtype or histology (if reported)

Method of outcome ascertainment

Adjustment Factors:

Confounding adjustment (specify variables)(Use "+" for matched or stratified variables)

VII. Results

UNADJUSTED- Risk estimate(s) and 95% confidence interval and/or p-value
Example: [OR 1.5 (1.3,2)]

ADJUSTED- Risk estimate(s) and 95% confidence interval and/or p-value
Example: [females, OR 1.2 (1.0, 1.3); p-value 0.005]; [age, OR 1.4 (1.2, 1.6)]

Was there an apparent exposure exposure-response?

- ☐ NA, single dose or exposure study
- ☐ NA, no associations at any exposure level and no trend analysis
- ☐ Yes, statistically significant trend (list p-value)
- ☐ No, no statistically significant trend (list p-value)
- ☐ Not sure

Exposure-response summary across exposure groups

Strengths Reported in the Study:

Limitations Reported in the Study:

Strengths, You, Yourself, Have Concluded about the Study

Limitations, You, Yourself, Have Concluded about the Study

Main Finding?

Author Overall Conclusion

Select an Answer
supportive of positive association
supportive of negative (or inverse) association
no association
unclear

COMMENTS?

Supplementary Material IV: List of excluded human studies with reasons for exclusion

Level 1 – Reasons for exclusion

Serial	Reference	Exclusion Criteria
1	**Keskin, N.,Teksen, Y. A.,Ongun, E. G.,Ozay, Y.,Saygili, H.**. Does long-term talc exposure have a carcinogenic effect on the female genital system of rats? An experimental pilot study. *Archives of Gynecology & Obstetrics.* 2009. 280:925-31	Level 1, Form Scr - T & A (Human), Does this study involve human subjects? -> No
2	**Boorman, G. A.,Seely, J. C.**. The lack of an ovarian effect of lifetime talc exposure in F344/N rats and B6C3F1 mice. *Regulatory Toxicology & Pharmacology.* 1995. 21:242-3	Level 1, Form Scr - T & A (Human), Does this study involve human subjects? -> No
3	**Gordon, R. E.,Fitzgerald, S.,Millette, J.**. Asbestos in commercial cosmetic talcum powder as a cause of mesothelioma in women.[Erratum appears in Int J Occup Environ Health. 2015;21(4):347-8; PMID: 26743791]. *International Journal of Occupational & Environmental Health.* 2014. 20:318-32	Level 1, Form Scr - T & A (Human), Is this study related to ovarian cancer? -> No
4	**Ried, M.,Hofmann, H. S.**. The treatment of pleural carcinosis with malignant pleural effusion. *Deutsches Arzteblatt International.* 2013. 110:313-8	Level 1, Form Scr - T & A (Human), Is this study related to ovarian cancer? -> No
5	**Filosso, P. L.,Sandri, A.,Felletti, G.,Ruffini, E.,Lausi, P. O.,Oliaro, A.**. Preliminary results of a new small-bore percutaneous pleural catheter used for treatment of malignant pleural effusions in ECOG PS 3-4 patients. *European Journal of Surgical Oncology.* 2011. 37:1093-8	Level 1, Form Scr - T & A (Human), Is this study related to ovarian cancer? -> No

Serial	Reference	Exclusion Criteria
6	**Bielsa, S.,Hernandez, P.,Rodriguez-Panadero, F.,Taberner, T.,Salud, A.,Porcel, J. M.**. Tumor type influences the effectiveness of pleurodesis in malignant effusions. *Lung.* 2011. 189:151-5	Level 1, Form Scr - T & A (Human), Is this study related to ovarian cancer? -> No
7	**Ozyurtkan, M. O.,Balci, A. E.,Cakmak, M.**. Predictors of mortality within three months in the patients with malignant pleural effusion. *European Journal of Internal Medicine.* 2010. 21:30-4	Level 1, Form Scr - T & A (Human), Is this study related to ovarian cancer? -> No
8	**Gross, J. L.,Disanzio, T. G.,Younes, R. N.,Haddad, F. J.,da Silva, R. A.,Avertano, A. B.**. Do concomitant ascites influence the effectiveness of palliative surgical management of pleural effusion in patients with malignancies?. *World Journal of Surgery.* 2009. 33:266-71	Level 1, Form Scr - T & A (Human), Is this study related to ovarian cancer? -> No
9	**Sioris, T.,Sihvo, E.,Salo, J.,Rasanen, J.,Knuuttila, A.**. Long-term indwelling pleural catheter (PleurX) for malignant pleural effusion unsuitable for talc pleurodesis. *European Journal of Surgical Oncology.* 2009. 35:546-51	Level 1, Form Scr - T & A (Human), Is this study related to ovarian cancer? -> No
10	**Medford, A. R.,Maskell, N. A.**. A national survey of oncologist and chest physicians' attitudes towards empirical anti-oestrogen therapy, early pleurodesis and preference of sclerosing agent in malignant breast and ovarian pleural disease. *Palliative Medicine.* 2005. 19:430-1	Level 1, Form Scr - T & A (Human), Is this study related to ovarian cancer? -> No
11	**Bondoc, A. Y.,Bach, P. B.,Sklarin, N. T.,Vander Els, N. J.**. Arterial desaturation syndrome following pleurodesis with talc slurry: incidence, clinical features, and outcome. *Cancer Investigation.* 2003. 21:848-54	Level 1, Form Scr - T & A (Human), Is this study related to ovarian cancer? -> No
12	**Erickson, K. V.,Yost, M.,Bynoe, R.,Almond, C.,Nottingham, J.**. Primary treatment of malignant pleural effusions:	Level 1, Form Scr - T & A (Human), Is this study related to ovarian cancer? -> No

Serial	Reference	Exclusion Criteria
	video-assisted thoracoscopic surgery poudrage versus tube thoracostomy. *American Surgeon.* 2002. 68:955-9; discussion 959-60	
13	**Mager, H. J.,Maesen, B.,Verzijlbergen, F.,Schramel, F.**. Distribution of talc suspension during treatment of malignant pleural effusion with talc pleurodesis. *Lung Cancer.* 2002. 36:77-81	Level 1, Form Scr - T & A (Human), Is this study related to ovarian cancer? -> No
14	**Bronner, G. M.,Baas, P.,Beijnen, J. H.**. [Pleurodesis in malignant pleural effusion]. *Nederlands Tijdschrift voor Geneeskunde.* 1997. 141:1810-4	Level 1, Form Scr - T & A (Human), Is this study related to ovarian cancer? -> No
15	**Sanchez-Armengol, A.,Rodriguez-Panadero, F.**. Survival and talc pleurodesis in metastatic pleural carcinoma, revisited. Report of 125 cases. *Chest.* 1993. 104:1482-5	Level 1, Form Scr - T & A (Human), Is this study related to ovarian cancer? -> No
16	**Webb, W. R.,Ozmen, V.,Moulder, P. V.,Shabahang, B.,Breaux, J.**. Iodized talc pleurodesis for the treatment of pleural effusions. *Journal of Thoracic & Cardiovascular Surgery.* 1992. 103:881-5; discussion 885-6	Level 1, Form Scr - T & A (Human), Is this study related to ovarian cancer? -> No
17	**Ladjimi, S.,M'Raihi, L.,Djemel, A.,Mathlouthi, A.,Ben Ayed, F.,Zegaya, M.**. [Results of talc administration using thoracoscopy in neoplastic pleurisies. Apropos of 218 cases]. *Revue des Maladies Respiratoires.* 1989. 6:147-50	Level 1, Form Scr - T & A (Human), Is this study related to ovarian cancer? -> No
18	**Adler, R. H.,Sayek, I.**. Treatment of malignant pleural effusion: a method using tube thoracostomy and talc. *Annals of Thoracic Surgery.* 1976. 22:8-15	Level 1, Form Scr - T & A (Human), Is this study related to ovarian cancer? -> No
19	**Shedbalkar, A. R.,Head, J. M.,Head, L. R.,Murphy, D. J.,Mason, J. H.**. Evaluation of talc pleural symphysis in management of malignant pleural effusion. *Journal of*	Level 1, Form Scr - T & A (Human), Is this study related to ovarian cancer? -> No

Serial	Reference	Exclusion Criteria
	Thoracic & Cardiovascular Surgery. 1971. 61:492-7	
20	**Adler, R. H.,Rappole, B. W.**. Recurrent malignant pleural effusions and talc powder aerosol treatment. *Surgery.* 1967. 62:1000-6	Level 1, Form Scr - T & A (Human), Is this study related to ovarian cancer? -> No
21	**Sreter, K. B.,Jakopovic, M.,Janevski, Z.,Samarzija, M.,Zarogoulidis, P.,Kioumis, I.,Mparmpetakis, N.,Pataka, A.,Zarogoulidis, K.,Tsiouda, T.,Kosmidis, C.,Mpaka, S.,Huang, H.,Hohenforst-Schmidt, W.,Charalampidis, C.,Machairiotis, N.,Zaric, B.,Milovancev, A.**. A pilot study-is there a role for mitoxantrone pleurodesis in the management of pleural effusion due to lung cancer?. *Annals of Translational Medicine.* 2016. 4 (9) (no pagination):#pages#	Level 1, Form Scr - T & A (Human), Is this study related to ovarian cancer? -> No
22	**Khaleeq, G.,Musani, A. I.**. Emerging paradigms in the management of malignant pleural effusions. *Respiratory Medicine.* 2008. 102:939-948	Level 1, Form Scr - T & A (Human), Is this study related to ovarian cancer? -> No
23	**Neragi-Miandoab, S.**. Surgical and other invasive approaches to recurrent pleural effusion with malignant etiology. *Supportive Care in Cancer.* 2008. 16:1323-1331	Level 1, Form Scr - T & A (Human), Is this study related to ovarian cancer? -> No
24	**Laisaar, T.,Palmiste, V.,Vooder, T.,Umbleja, T.**. Life expectancy of patients with malignant pleural effusion treated with video-assisted thoracoscopic talc pleurodesis. *Interactive Cardiovascular and Thoracic Surgery.* 2006. 5:307-310	Level 1, Form Scr - T & A (Human), Is this study related to ovarian cancer? -> No
25	**Szeto, C. C.,Chow, K. M.**. Pathogenesis and management of hydrothorax complicating peritoneal dialysis. *Current Opinion in Pulmonary Medicine.* 2004. 10:315-319	Level 1, Form Scr - T & A (Human), Is this study related to ovarian cancer? -> No

Serial	Reference	Exclusion Criteria
26	**Critchley, L. A.,Au, H. K.,Yim, A. P.**. Reexpansion pulmonary edema occurring after thoracoscopic drainage of a pleural effusion. *Journal of Clinical Anesthesia.* 1996. 8:591-4	Level 1, Form Scr - T & A (Human), Is this study related to Talc? (inc... -> No
27	**Shlebak, A. A.,Clark, P. I.,Green, J. A.**. Hypersensitivity and cross-reactivity to cisplatin and analogues. *Cancer Chemotherapy & Pharmacology.* 1995. 35:349-51	Level 1, Form Scr - T & A (Human), Is this study related to Talc? (inc... -> No
28	**Nanthakomon, T.,Pongrojpaw, D.**. The efficacy of ginger in prevention of postoperative nausea and vomiting after major gynecologic surgery. *J Med Assoc Thai.* 2006. 89 Suppl 4:S130-6	Level 1, Form Scr - T & A (Human), Is this study related to Talc? (inc... -> No
29	**Gupta, P. C.,Ray, C. S.**. Smokeless tobacco and health in India and South Asia. *Respirology.* 2003. 8:419-31	Level 1, Form Scr - T & A (Human), Is this study related to Talc? (inc... -> No
30	**Yun, T. K.,Choi, S. Y.**. Preventive effect of ginseng intake against various human cancers: a case-control study on 1987 pairs. *Cancer Epidemiol Biomarkers Prev.* 1995. 4:401-8	Level 1, Form Scr - T & A (Human), Is this study related to Talc? (inc... -> No
31	**Campitiello, F.,Della Corte, A.,Fattopace, A.,D'Acunzi, D.,Canonico, S.**. The use of artificial dermis in the treatment of chronic and acute wounds: Regeneration of dermis and wound healing. *Acta Biomedica de l'Ateneo Parmense.* 2005. 76:69-71	Level 1, Form Scr - T & A (Human), Is this study related to Talc? (inc... -> No
32	**Dalley, V. M.**. The role of radiotherapy and chemotherapy in the treatment of cancer of the ovary. *International Journal of Radiation Oncology Biology Physics.* 1982. 8:251-255	Level 1, Form Scr - T & A (Human), Is this study related to Talc? (inc... -> No
33	**Freundlich, I. M.,Lind, T. A.**. Calcification of the heart and great vessels. *CRC critical reviews in clinical radiology and nuclear medicine.* 1975. 6:171-216	Level 1, Form Scr - T & A (Human), Is this study related to Talc? (inc... -> No

Serial	Reference	Exclusion Criteria
34	**N. H. S. Greater Glasgow,Clyde,,Rocket Medical, plc**. Manometry vs Clinical Assessment in the Detection of Trapped Lung in Patients With Suspected Pleural Malignancy. *#journal#.* 2017. #volume#:#pages#	Level 1, Form Scr - T & A (Human), Is this study related to Talc? (inc... -> No
35	**Postgraduate Institute of Medical, Education,Research,**. A Study to Compare the Efficacy and Safety of Intrapleural Doxycycline Versus Iodopovidone for Performing Pleurodesis in Malignant Pleural Effusion. *#journal#.* 2017. #volume#:#pages#	Level 1, Form Scr - T & A (Human), Is this study related to Talc? (inc... -> No
36	**Oxford University Hospitals, N. H. S. Trust,National Cancer, Institute**. Ibuprofen or Morphine in Treating Pain in Patients Undergoing Pleurodesis for Malignant Pleural Effusion. *#journal#.* 2009. #volume#:#pages#	Level 1, Form Scr - T & A (Human), Is this study related to Talc? (inc... -> No
37	**Seoul National University, Hospital,Pharmbio Korea Co, Ltd**. The Effects of High Dose Pancreatic Enzyme Replacement Therapy After Pancreatoduodenectomy. *#journal#.* 2018. #volume#:#pages#	Level 1, Form Scr - T & A (Human), Is this study related to Talc? (inc... -> No
38	**NCT02002117**. Genotyping of Non-small Cell Lung Cancer. *#journal#.* 2013. #volume#:#pages#	Level 1, Form Scr - T & A (Human), Is this study related to Talc? (inc... -> No
39	**EUCTR2013-002380-25-SE**. A randomized study to evaluate whether there is a difference in survival between two different forms of anaesthesia. The study will evaluate patients that are scheduled for a surgery for either breast-, colon- or rectalcancer.. *#journal#.* 2013. #volume#:#pages#	Level 1, Form Scr - T & A (Human), Is this study related to Talc? (inc... -> No
40	**EUCTR2007-007834-21-DK**. FLOX + Erbitux. 1. line treatment to patients with metastatic colorectal cancer and wild type	Level 1, Form Scr - T & A (Human), Is this study related to Talc? (inc... -> No

Serial	Reference	Exclusion Criteria
	K-RAS tumor. A phase II study. - Nordic 7.5. *#journal#.* 2008. #volume#:#pages#	
41	**NCT00718055**. Magnetic Resonance Imaging (MRI) Using Liverspecific Oral Contrast Agent in Metastatic Colorectal Cancer Patients. *#journal#.* 2006. #volume#:#pages#	Level 1, Form Scr - T & A (Human), Is this study related to Talc? (inc... -> No
42	**NCT00111774**. Evaluating ABX-EGF in Patients With Metastatic ColorectalCarcinoma. *#journal#.* 2005. #volume#:#pages#	Level 1, Form Scr - T & A (Human), Is this study related to Talc? (inc... -> No
43	. Prospective blinded randomised placebo controlled trial investigating whether oxycodone modified release reduces parenteral opioid use following intermediate thoracic surgery . *#journal#.* #year#. #volume#:#pages#	Level 1, Form Scr - T & A (Human), Is this study related to Talc? (inc... -> No
44	. Examining the effect of intravenous zoledronic acid on pleural fluid production, breathlessness and quality of life in patients with a malignant pleural effusion . *#journal#.* #year#. #volume#:#pages#	Level 1, Form Scr - T & A (Human), Is this study related to Talc? (inc... -> No
45	. Rifaximin for Preventing Relapse of Clostridium Associated Diarrhoea . *#journal#.* #year#. #volume#:#pages#	Level 1, Form Scr - T & A (Human), Is this study related to Talc? (inc... -> No
46	. A Multicentre Study to Evaluate the Safety and Efficacy of AD 923 in Comparison to MSIR for the Treatment of CBP in Subjects . *#journal#.* #year#. #volume#:#pages#	Level 1, Form Scr - T & A (Human), Is this study related to Talc? (inc... -> No
47	. Study to Assess the Efficacy and Safety of Raxone in LHON Patients . *#journal#.* #year#. #volume#:#pages#	Level 1, Form Scr - T & A (Human), Is this study related to Talc? (inc... -> No
48	. Efficacy, Pharmacokinetics, and Safety of Presatovir in Hospitalized Adults With Respiratory Syncytial Virus (RSV) Infection . *#journal#.* #year#. #volume#:#pages#	Level 1, Form Scr - T & A (Human), Is this study related to Talc? (inc... -> No

Serial	Reference	Exclusion Criteria
49	. Bortezomib in Treating Patients With Malignant Pleural Mesothelioma . *#journal#.* #year#. #volume#:#pages#	Level 1, Form Scr - T & A (Human), Is this study related to Talc? (inc... -> No
50	. Efavirenz to Dolutegravir Switch in Patients With CNS Toxicity . *#journal#.* #year#. #volume#:#pages#	Level 1, Form Scr - T & A (Human), Is this study related to Talc? (inc... -> No
51	. SSAT061: PK of DTG and EVT/COBI in Healthy Volunteers . *#journal#.* #year#. #volume#:#pages#	Level 1, Form Scr - T & A (Human), Is this study related to Talc? (inc... -> No
52	. REPAIR: Right vEntricular Remodeling in Pulmonary ArterIal hypeRtension . *#journal#.* #year#. #volume#:#pages#	Level 1, Form Scr - T & A (Human), Is this study related to Talc? (inc... -> No

Level 2 – Reasons for exclusion

Serial	Reference	Exclusion Criteria
1	**Ainsworth, S.**. Not safe for babies' bottom?. *Practising Midwife.* 2009. 12:42	Level 2, Form Scr - FTxt (Humans), Is the full-article PDF available? -> No
2	**Horiuchi, A.,Konishi, I.**. [Prevention of ovarian cancer development]. *Nippon Rinsho - Japanese Journal of Clinical Medicine.* 2004. 62 Suppl 10:597-600	Level 2, Form Scr - FTxt (Humans), Is the full-article PDF available? -> No
3	**Tamaya, T.**. [Epidemiology of ovarian cancer]. *Nippon Rinsho - Japanese Journal of Clinical Medicine.* 2004. 62 Suppl 10:435-40	Level 2, Form Scr - FTxt (Humans), Is the full-article PDF available? -> No
4	**Williams, K. A.,Labidi-Galy, S. I.,Terry, K. L.,Vitonis, A. F.,Welch, W. R.,Goodman, A.,Cramer, D. W.**. Prognostic significance and predictors of the neutrophil-to-lymphocyte ratio in ovarian cancer. *Gynecologic Oncology.* 2014. 132:542-50	Level 2, Form Scr - FTxt (Humans), Is this study related to ovarian cancer? -> No
5	**Crawford, L.,Reeves, K. W.,Luisi, N.,Balasubramanian, R.,Sturgeon, S. R.**. Perineal powder use and risk of endometrial cancer in postmenopausal women. *Cancer Causes & Control.* 2012. 23:1673-80	Level 2, Form Scr - FTxt (Humans), Is this study related to ovarian cancer? -> No
6	**Neill, A. S.,Nagle, C. M.,Spurdle, A. B.,Webb, P. M.**. Use of talcum powder and endometrial cancer risk. *Cancer Causes & Control.* 2012. 23:513-9	Level 2, Form Scr - FTxt (Humans), Is this study related to ovarian cancer? -> No
7	**Karageorgi, S.,Gates, M. A.,Hankinson, S. E.,De Vivo, I.**. Perineal use of talcum powder and endometrial cancer risk. *Cancer Epidemiology, Biomarkers & Prevention.* 2010. 19:1269-75	Level 2, Form Scr - FTxt (Humans), Is this study related to ovarian cancer? -> No
8	**Lowe, K. A.,Shah, C.,Wallace, E.,Anderson, G.,Paley, P.,McIntosh, M.,Andersen, M. R.,Scholler, N.,Bergan, L.,Thorpe, J.,Urban,**	Level 2, Form Scr - FTxt (Humans), Is this study related to ovarian cancer? -> No

Serial	Reference	Exclusion Criteria
	**N.,Drescher, C. W.**. Effects of personal characteristics on serum CA125, mesothelin, and HE4 levels in healthy postmenopausal women at high-risk for ovarian cancer. *Cancer Epidemiology, Biomarkers & Prevention.* 2008. 17:2480-7	
9	**Pauler, D. K.,Menon, U.,McIntosh, M.,Symecko, H. L.,Skates, S. J.,Jacobs, I. J.**. Factors influencing serum CA125II levels in healthy postmenopausal women. *Cancer Epidemiology, Biomarkers & Prevention.* 2001. 10:489-93	Level 2, Form Scr - FTxt (Humans), Is this study related to ovarian cancer? -> No
10	**Rosenblatt, K. A.,Mathews, W. A.,Daling, J. R.,Voigt, L. F.,Malone, K.**. Characteristics of women who use perineal powders. *Obstetrics & Gynecology.* 1998. 92:753-6	Level 2, Form Scr - FTxt (Humans), Is this study related to ovarian cancer? -> No
11	**Heller, D. S.,Westhoff, C.,Gordon, R. E.,Katz, N.**. The relationship between perineal cosmetic talc usage and ovarian talc particle burden. *American Journal of Obstetrics & Gynecology.* 1996. 174:1507-10	Level 2, Form Scr - FTxt (Humans), Is this study related to ovarian cancer? -> No
12	**Eltabbakh, G. H.,Piver, M. S.,Natarajan, N.,Mettlin, C. J.**. Epidemiologic differences between women with extraovarian primary peritoneal carcinoma and women with epithelial ovarian cancer. *Obstetrics & Gynecology.* 1998. 91:254-9	Level 2, Form Scr - FTxt (Humans), Is this study related to ovarian cancer? -> Other Types of Cancer?
13	**Karageorgis, S.,Gates, M.,Hankinson, S.,De Vivo, I.**. Perineal use of talcum powder and endometrial cancer risk. *Epidemiology.* 2011. 22:S122-S123	Level 2, Form Scr - FTxt (Humans), Is this study related to ovarian cancer? -> Other Types of Cancer?
14	**Crawford, L.,Sturgeon, S.,Luisi, N.,Balasubramanian, R.,Reeves, K.**. Perineal talc use and risk of endometrial	Level 2, Form Scr - FTxt (Humans), Is this study related

Serial	Reference	Exclusion Criteria
	cancer in postmenopausal women. *American Journal of Epidemiology.* 2011. 173:S233	to ovarian cancer? -> Other Types of Cancer?
15	**Trabert, B.,Pinto, L.,Hartge, P.,Kemp, T.,Black, A.,Sherman, M. E.,Brinton, L. A.,Pfeiffer, R. M.,Shiels, M. S.,Chaturvedi, A. K.,Hildesheim, A.,Wentzensen, N.**. Pre-diagnostic serum levels of inflammation markers and risk of ovarian cancer in the prostate, lung, colorectal and ovarian cancer (PLCO) screening trial. *Gynecologic Oncology.* 2014. 135:297-304	Level 2, Form Scr - FTxt (Humans), Is this study related to Talc? (inclu... -> No
16	**Lombardi, G.,Nicoletto, M. O.,Gusella, M.,Fiduccia, P.,Dalla Palma, M.,Zuin, A.,Fiore, D.,Donach, M.,Zagonel, V.**. Intrapleural paclitaxel for malignant pleural effusion from ovarian and breast cancer: a phase II study with pharmacokinetic analysis. *Cancer Chemotherapy & Pharmacology.* 2012. 69:781-7	Level 2, Form Scr - FTxt (Humans), Is this study related to Talc? (inclu... -> No
17	**Kim, S.,Dolecek, T. A.,Davis, F. G.**. Racial differences in stage at diagnosis and survival from epithelial ovarian cancer: a fundamental cause of disease approach. *Social Science & Medicine.* 2010. 71:274-81	Level 2, Form Scr - FTxt (Humans), Is this study related to Talc? (inclu... -> No
18	**Heller, D. S.,Gordon, R. E.,Westhoff, C.,Gerber, S.**. Asbestos exposure and ovarian fiber burden. *American Journal of Industrial Medicine.* 1996. 29:435-9	Level 2, Form Scr - FTxt (Humans), Is this study related to Talc? (inclu... -> No
19	**Koch, M.,Starreveld, A. A.,Hill, G. B.,Jenkins, H.**. The effect of tubal ligation on the incidence of epithelial cancer of the ovary. *Cancer Detection & Prevention.* 1984. 7:241-5	Level 2, Form Scr - FTxt (Humans), Is this study related to Talc? (inclu... -> No

Serial	Reference	Exclusion Criteria
20	**Canadian Agency for Drugs and Technologies in Health (CADTH),** . Powders as a Skin Care Aide for Patients in Hospitals or Long Term Care Facilities: Clinical Evidence and Guidelines . *Rapid Response Reports.* 2012. #volume#:#pages#	Level 2, Form Scr - FTxt (Humans), Is this study related to Talc? (inclu... -> No
21	**Goodman,M.T.,Lurie,G.,Thompson,P.J.,McDuffie,K.E.,Carney,M.E.**. Association of two common single-nucleotide polymorphisms in the CYP19A1 locus and ovarian cancer risk. *Endocr.Relat Cancer.* 2008. 15:1055-1060	Level 2, Form Scr - FTxt (Humans), Is this study related to Talc? (inclu... -> No
22	**Lo-Ciganic,W.H.,Zgibor,J.C.,Bunker,C.H.,Moysich,K.B.,Edwards,R.P.,Ness,R.B.**. Aspirin, nonaspirin nonsteroidal anti-inflammatory drugs, or acetaminophen and risk of ovarian cancer. *Epidemiology.* 2012. 23:311-319	Level 2, Form Scr - FTxt (Humans), Is this study related to Talc? (inclu... -> No
23	**Morgan, L.,Goh, C.,Kamal, R.,Daniels, R.,Ng, B.,Wilson, P.,Flynn, P.**. PleurX catheters provide safe and effective control of malignant pleural effusion. *Journal of Thoracic Oncology.* 2012. 3):S179	Level 2, Form Scr - FTxt (Humans), Is this study related to Talc? (inclu... -> No
24	**Su, S.**. Mesothelioma: Path to Multimodality Treatment. *Seminars in Thoracic and Cardiovascular Surgery.* 2009. 21:125-131	Level 2, Form Scr - FTxt (Humans), Is this study related to Talc? (inclu... -> No
25	**Ness, R. B.**. Ovarian cancer, inflammation and endometriosis. *CME Journal of Gynecologic Oncology.* 2003. 8:33-40	Level 2, Form Scr - FTxt (Humans), Is this study related to Talc? (inclu... -> No
26	**Kelly, M. G.,Pejovic, T.,Nezhat, F. R.**. What is the relationship between endometriosis and epithelial ovarian cancer?. *CME Journal of Gynecologic Oncology.* 2003. 8:41-47	Level 2, Form Scr - FTxt (Humans), Is this study related to Talc? (inclu... -> No

Serial	Reference	Exclusion Criteria
27	**Porzio, G.,Marchetti, P.,Paris, I.,Narducci, F.,Ricevuto, E.,Ficorella, C.**. Hypersensitivity reaction to carboplatin: Successful resolution by replacement with cisplatin. *European Journal of Gynaecological Oncology.* 2002. 23:335-336	Level 2, Form Scr - FTxt (Humans), Is this study related to Talc? (inclu... -> No
28	**Tortolero-Luna, G.,Mitchell, M. F.,Rhodes-Morris, H. E.**. Epidemiology and screening of ovarian cancer. *Obstetrics and Gynecology Clinics of North America.* 1994. 21:1-23	Level 2, Form Scr - FTxt (Humans), Is this study related to Talc? (inclu... -> No
29	**Dietl, J.,Marzusch, K.**. Ovarian surface epithelium and human ovarian cancer. *Gynecologic and Obstetric Investigation.* 1993. 35:129-135	Level 2, Form Scr - FTxt (Humans), Is this study related to Talc? (inclu... -> No
30	**Kerr, D. J.,Los, G.**. Pharmacokinetic principles of locoregional chemotherapy. *Cancer Surveys.* 1993. 17:105-122	Level 2, Form Scr - FTxt (Humans), Is this study related to Talc? (inclu... -> No
31	**Goff, B. A.,Mueller, P. R.,Muntz, H. G.,Rice, L. W.**. Small chest-tube drainage followed by bleomycin sclerosis for malignant pleural effusions. *Obstetrics and Gynecology.* 1993. 81:993-996	Level 2, Form Scr - FTxt (Humans), Is this study related to Talc? (inclu... -> No
32	**Rasmussen, Christina B.,Kjaer, Susanne K.,Albieri, Vanna,Bandera, Elisa V.,Doherty, Jennifer A.,Høgdall, Estrid,Webb, Penelope M.,Jordan, Susan J.,Rossing, Mary Anne,Wicklund, Kristine G.,Goodman, Marc T.,Modugno, Francesmary,Moysich, Kirsten B.,Ness, Roberta B.,Edwards, Robert P.,Schildkraut, Joellen M.,Berchuck, Andrew,Olson, Sara H.,Kiemeney, Lambertus A.,Massuger, Leon F. A. G.**. Pelvic Inflammatory Disease and the Risk of Ovarian Cancer and Borderline Ovarian Tumors: A Pooled Analysis of 13	Level 2, Form Scr - FTxt (Humans), Is this study related to Talc? (inclu... -> No

Serial	Reference	Exclusion Criteria
	Case-Control Studies. *American Journal of Epidemiology.* 2017. 185:8-20	
33	. Pharmacokinetic Effect of Evotaz/Microgynon Co-administration . *#journal#.* #year#. #volume#:#pages#	Level 2, Form Scr - FTxt (Humans), Is this study related to Talc? (inclu... -> No
34	. SSAT067 PK of Atazanavir/Cobicistat and Darunavir/Cobicistat . *#journal#.* #year#. #volume#:#pages#	Level 2, Form Scr - FTxt (Humans), Is this study related to Talc? (inclu... -> No
35	**Wehner, A. P.**. Cosmetic talc should not be listed as a carcinogen: comments on NTP's deliberations to list talc as a carcinogen. *Regulatory Toxicology & Pharmacology.* 2002. 36:40-50	Level 2, Form Scr - FTxt (Humans), What is the route of exposure? -> Inhalational
36	**Bulbulyan, M. A.,Ilychova, S. A.,Zahm, S. H.,Astashevsky, S. V.,Zaridze, D. G.**. Cancer mortality among women in the Russian printing industry. *American Journal of Industrial Medicine.* 1999. 36:166-71	Level 2, Form Scr - FTxt (Humans), What is the route of exposure? -> Inhalational
37	**Langseth, H.,Andersen, A.**. Cancer incidence among women in the Norwegian pulp and paper industry. *American Journal of Industrial Medicine.* 1999. 36:108-13	Level 2, Form Scr - FTxt (Humans), What is the route of exposure? -> Inhalational
38	**Hartge, P.,Stewart, P.**. Occupation and ovarian cancer: a case-control study in the Washington, DC, metropolitan area, 1978-1981. *Journal of Occupational Medicine.* 1994. 36:924-7	Level 2, Form Scr - FTxt (Humans), What is the route of exposure? -> Inhalational
39	**Verma, A.,Taha, A.,Venkateswaran, S.,Tee, A.**. Effectiveness of medical thoracoscopy and thoracoscopic talc poudrage in patients with exudative	Level 2, Form Scr - FTxt (Humans), What is the route of exposure? -> Others (eg: injections)

Serial	Reference	Exclusion Criteria
	pleural effusion. *Singapore Medical Journal.* 2015. 56:268-73	
40	**Lumachi, F.,Mazza, F.,Ermani, M.,Chiara, G. B.,Basso, S. M.**. Talc pleurodesis as surgical palliation of patients with malignant pleural effusion. Analysis of factors affecting survival. *Anticancer Research.* 2012. 32:5071-4	Level 2, Form Scr - FTxt (Humans), What is the route of exposure? -> Others (eg: injections)
41	**Whitworth, J. M.,Schneider, K. E.,Fauci, J. M.,Bryant, A. S.,Cerfolio, R. J.,Straughn, J. M., Jr.**. Outcomes of patients with gynecologic malignancies undergoing video-assisted thoracoscopic surgery (VATS) and pleurodesis for malignant pleural effusion. *Gynecologic Oncology.* 2012. 125:646-8	Level 2, Form Scr - FTxt (Humans), What is the route of exposure? -> Others (eg: injections)
42	**Barbetakis, N.,Asteriou, C.,Papadopoulou, F.,Samanidis, G.,Paliouras, D.,Kleontas, A.,Lyriti, K.,Katsikas, I.,Tsilikas, C.**. Early and late morbidity and mortality and life expectancy following thoracoscopic talc insufflation for control of malignant pleural effusions: a review of 400 cases. *Journal Of Cardiothoracic Surgery.* 2010. 5:27	Level 2, Form Scr - FTxt (Humans), What is the route of exposure? -> Others (eg: injections)
43	**Horn, D.,Dequanter, D.,Lothaire, P.**. Palliative treatment of malignant pleural effusions. *Acta Chirurgica Belgica.* 2010. 110:32-4	Level 2, Form Scr - FTxt (Humans), What is the route of exposure? -> Others (eg: injections)
44	**Buz'Zard, A. R.,Lau, B. H.**. Pycnogenol reduces talc-induced neoplastic transformation in human ovarian cell cultures. *Phytotherapy Research.* 2007. 21:579-86	Level 2, Form Scr - FTxt (Humans), What is the route of exposure? -> Others (eg: injections)
45	**Griffo, S.,Gravino, E.,Luciano, A.,Ferrante, G.**. The treatment by V.A.T.S. and M.A.C. of secondary neoplastic pleural effusion in the old	Level 2, Form Scr - FTxt (Humans), What is the route of exposure? -> Others (eg: injections)

Serial	Reference	Exclusion Criteria
	patient (> 70 years). *Acta Bio-Medica de l Ateneo Parmense.* 2005. 76 Suppl 1:72-5	
46	**Kolschmann, S.,Ballin, A.,Gillissen, A.**. Clinical efficacy and safety of thoracoscopic talc pleurodesis in malignant pleural effusions. *Chest.* 2005. 128:1431-5	Level 2, Form Scr - FTxt (Humans), What is the route of exposure? -> Others (eg: injections)
47	**Chi, D. S.,Abu-Rustum, N. R.,Sonoda, Y.,Chen, S. W.,Flores, R. M.,Downey, R.,Aghajanian, C.,Barakat, R. R.**. The benefit of video-assisted thoracoscopic surgery before planned abdominal exploration in patients with suspected advanced ovarian cancer and moderate to large pleural effusions. *Gynecologic Oncology.* 2004. 94:307-11	Level 2, Form Scr - FTxt (Humans), What is the route of exposure? -> Others (eg: injections)
48	**Sahn, S. A.**. Malignancy metastatic to the pleura. *Clinics in Chest Medicine.* 1998. 19:351-61	Level 2, Form Scr - FTxt (Humans), What is the route of exposure? -> Others (eg: injections)
49	**Saka, H.,Shimokata, K.**. [State of the art: treatment of malignant pleural and pericardial effusions]. *Gan to Kagaku Ryoho [Japanese Journal of Cancer & Chemotherapy].* 1997. 24 Suppl 3:418-25	Level 2, Form Scr - FTxt (Humans), What is the route of exposure? -> Others (eg: injections)
50	**Kupryjanczyk, J.**. Adenomatoid tumour of the ovary and uterus in the same patient. *Zentralblatt fur Allgemeine Pathologie und Pathologische Anatomie.* 1989. 135:437-44	Level 2, Form Scr - FTxt (Humans), What is the route of exposure? -> Others (eg: injections)
51	**Dietl, J.,Buchholz, F.,Stoll, P.**. [Ovarian surface epithelium and its histogenic relation to ovarian cancer]. *Geburtshilfe und Frauenheilkunde.* 1986. 46:561-6	Level 2, Form Scr - FTxt (Humans), What is the route of exposure? -> Others (eg: injections)
52	**Natow, A. J.**. Talc: need we beware?. *Cutis.* 1986. 37:328-9	Level 2, Form Scr - FTxt (Humans), What is the route of

Serial	Reference	Exclusion Criteria
		exposure? -> Others (eg: injections)
53	**Curie, P.,Sussmann, M.,Treisser, A.,Renaud, R.**. [Epidemiologic factors in ovarian cancer]. *Revue Francaise de Gynecologie et d Obstetrique.* 1985. 80:379-82	Level 2, Form Scr - FTxt (Humans), What is the route of exposure? -> Others (eg: injections)
54	**Greene, M. H.,Clark, J. W.,Blayney, D. W.**. The epidemiology of ovarian cancer. *Seminars in Oncology.* 1984. 11:209-26	Level 2, Form Scr - FTxt (Humans), What is the route of exposure? -> Others (eg: injections)
55	**Roe, F. J.**. Controversy: cosmetic talc and ovarian cancer. *Lancet.* 1979. 2:744	Level 2, Form Scr - FTxt (Humans), What is the route of exposure? -> Others (eg: injections)
56	**Henderson, W. J.,Hamilton, T. C.,Griffiths, K.**. Talc in normal and malignant ovarian tissue. *Lancet.* 1979. 1:499	Level 2, Form Scr - FTxt (Humans), What is the route of exposure? -> Others (eg: injections)
57	**Griffiths, K.,Chandler, J. A.,Henderson, W. J.,Joslin, C. A.**. Ovarian cancer: some new analytical approaches. *Postgraduate Medical Journal.* 1973. 49:69-72	Level 2, Form Scr - FTxt (Humans), What is the route of exposure? -> Others (eg: injections)
58	**Henderson, W. J.,Joslin, C. A.,Turnbull, A. C.,Griffiths, K.**. Talc and carcinoma of the ovary and cervix. *Journal of Obstetrics & Gynaecology of the British Commonwealth.* 1971. 78:266-72	Level 2, Form Scr - FTxt (Humans), What is the route of exposure? -> Others (eg: injections)
59	**Mitrofan, C.,Aldea, A.,Grigorescu, C.,Jitaru, I.,Iosep, G.,Arama, A.,Danciu, C. E.,Bolog, S.,Mihaescu, T.**. [Thoracoscopic pleurodesis in malignant pleural effusions]. *Rev Med Chir Soc Med Nat Iasi.* 2005. 109:799-803	Level 2, Form Scr - FTxt (Humans), What is the route of exposure? -> Others (eg: injections)

Serial	Reference	Exclusion Criteria
60	**Mandal, J.**. The key to solving the mystery of a refractory pleural effusion is... a pelvic exam?. *Journal of General Internal Medicine.* 2016. 1):S753-S754	Level 2, Form Scr - FTxt (Humans), What is the route of exposure? -> Others (eg: injections)
61	**Ogunrombi, A. B.,Onakpoya, U. U.,Ekrikpo, U.,Aderibigbe, A. S.,Aladesuru, O. A.**. Recurrence of malignant pleural effusion following pleurodesis: Is there a difference between use of povidone-iodine or cyclophosphamide?. *Annals of African Surgery.* 2014. 11:21-24	Level 2, Form Scr - FTxt (Humans), What is the route of exposure? -> Others (eg: injections)
62	**Baruzzo, E.,Arteni, AI,Ganzitti, L.,Della Martina, M.,Vogrig, E.,Fabiani, G.,Marchesoni, D.**. High grade serous ovarian epithelial carcinoma presenting with skin and breast metastasis: A case report. *International Journal of Gynecological Cancer. Conference: ESGO.* 2014. 24:#pages#	Level 2, Form Scr - FTxt (Humans), What is the route of exposure? -> Others (eg: injections)
63	**Kurbacher, C. M.,Horn, O.,Schweitzer, C.,Nymbach, N.,Herz, S.,Wessling, G.,Lepique, J.,Kurbacher, J. A.**. Outpatient treatment of malignant pleural effusions due to metastatic breast and ovarian carcinoma by intrapleural catumaxomab instillation. *Oncology Research and Treatment.* 2014. 37:30	Level 2, Form Scr - FTxt (Humans), What is the route of exposure? -> Others (eg: injections)
64	**Cumplido, J. D.,Soto, A.,Toral, J. C.**. Management of malignant pleural effusion. Experience in our center. *International Journal of Gynecological Cancer.* 2013. 1):1169	Level 2, Form Scr - FTxt (Humans), What is the route of exposure? -> Others (eg: injections)
65	**Thomas, R.,Lee, G.**. Indwelling pleural catheter (IPC) as a new management strategy for malignant pleural effusions (MPE). *Internal Medicine Journal.* 2013. 43:9-10	Level 2, Form Scr - FTxt (Humans), What is the route of exposure? -> Others (eg: injections)

Serial	Reference	Exclusion Criteria
66	**Schniewind, B.,Rose, T.,Woltmann, N.,Walter, J.,Becker, T.,Dohrmann, P.,Kuchler, T.,Kurdow, R.**. Clinical outcomes and health-related quality of life after thoracoscopic talc pleurodesis. *Journal of Palliative Medicine.* 2012. 15:37-42	Level 2, Form Scr - FTxt (Humans), What is the route of exposure? -> Others (eg: injections)
67	**Karkhanis, V. S.,Joshi, J. M.**. Pleural effusion: Diagnosis, treatment, and management. *Open Access Emergency Medicine.* 2012. 4:31-52	Level 2, Form Scr - FTxt (Humans), What is the route of exposure? -> Others (eg: injections)
68	**Takimoto, R.**. Malignant pleural and peritoneal carcinomatosis treatment. *Annals of Oncology.* 2012. 23:xi65	Level 2, Form Scr - FTxt (Humans), What is the route of exposure? -> Others (eg: injections)
69	**Lumachi, F.,Mazza, F.,Santeufemia, D. A.,Tumolo, S.,Del Conte, A.,Ermani, M.,Chiara, G. B.,Basso, S. M.**. Prognostic factors for survival of symptomatic patients with malignant pleural effusion who underwent palliative talc pleurodesis. *Journal of Thoracic Oncology.* 2012. 1):S78	Level 2, Form Scr - FTxt (Humans), What is the route of exposure? -> Others (eg: injections)
70	**Froudarakis, M. E.,Pataka, A.,Makris, D.,Kouliatsis, G.,Anevlavis, S.,Sotiriou, I.,Steiropoulos, P.,Eleftheriadis, S.,Bouros, D.**. Respiratory muscle strength and lung function in patients undergoing medical thoracoscopy. *Respiration.* 2010. 80:220-227	Level 2, Form Scr - FTxt (Humans), What is the route of exposure? -> Others (eg: injections)
71	**Anonymous,**. Studies of cancer in humans. *#journal#.* 2010. #volume#:348-382	Level 2, Form Scr - FTxt (Humans), What is the route of exposure? -> Others (eg: injections)
72	**Rodriguez-Freixinos, V.,Sanchez-Olle, G.,Argiles, G.,Nunez, I.,Mulet, N.,Martinez, P.,Cedres, S.,Lopez, I.,Canela, M.,Felip, E.**. Pleurodesis (P) outcome in a series of metastatic cancer	Level 2, Form Scr - FTxt (Humans), What is the route of exposure? -> Others (eg: injections)

Serial	Reference	Exclusion Criteria
	patients (PTS) with symtomatic malignant pleural effusion (MPE). *Annals of Oncology.* 2010. 21:viii398	
73	**Lombardi, G.,Gusella, M.,Dalla Palma, M.,Fiduccia, P.,Zustovich, F.,Pastorelli, D.,Farina, M.,Nicoletto, M. O.**. A phase II study of intrapleural paclitaxel against malignant pleural effusion (MPE): Effectiveness, safety and pharmacokinetic. *Annals of Oncology.* 2010. 21:viii158	Level 2, Form Scr - FTxt (Humans), What is the route of exposure? -> Others (eg: injections)
74	**Musani, A. I.**. Treatment options for malignant pleural effusion. *Current Opinion in Pulmonary Medicine.* 2009. 15:380-387	Level 2, Form Scr - FTxt (Humans), What is the route of exposure? -> Others (eg: injections)
75	**Heffner, J. E.**. Diagnosis and management of malignant pleural effusions. *Respirology.* 2008. 13:5-20	Level 2, Form Scr - FTxt (Humans), What is the route of exposure? -> Others (eg: injections)
76	**Lieber, A.,Doil, D.,Uschinsky, K.,Dusel, W.**. Relapse of a Meigs Syndrome Five Years after Bilateral Ovarectomy and Hysterectomy?. [German]. *Geburtshilfe und Frauenheilkunde.* 2004. 64:193-197	Level 2, Form Scr - FTxt (Humans), What is the route of exposure? -> Others (eg: injections)
77	**Ahmed, Z.,Shrager, J. B.**. Mediastinal talcoma masquerading as thymoma. *Annals of Thoracic Surgery.* 2003. 75:568-569	Level 2, Form Scr - FTxt (Humans), What is the route of exposure? -> Others (eg: injections)
78	**Harter, P.,Du Bois, A.**. Does tubal sterilization protect against ovarian cancer?. [German]. *Gynakologische Praxis.* 2003. 27:455-458	Level 2, Form Scr - FTxt (Humans), What is the route of exposure? -> Others (eg: injections)
79	**Bronner, G. M.,Baas, P.,Beijnen, J. H.**. Pleurodesis in malignant pleural effusions. [Dutch]. *Nederlands Tijdschrift voor Geneeskunde.* 1997. 141:1810-1814	Level 2, Form Scr - FTxt (Humans), What is the route of exposure? -> Others (eg: injections)

Serial	Reference	Exclusion Criteria
80	**Hott, J. W.**. Malignant pleural effusions. *Seminars in Respiratory and Critical Care Medicine.* 1995. 16:333-339	Level 2, Form Scr - FTxt (Humans), What is the route of exposure? -> Others (eg: injections)
81	**Gori, G. B.**. Session II: Introduction-ovarian exposure concerns. *Regulatory Toxicology and Pharmacology.* 1995. 21:252-253	Level 2, Form Scr - FTxt (Humans), What is the route of exposure? -> Others (eg: injections)
82	**Boente, M. P.,Godwin, A. K.,Hogan, W. M.**. Screening, imaging, and early diagnosis of ovarian cancer. *Clinical Obstetrics and Gynecology.* 1994. 37:377-391	Level 2, Form Scr - FTxt (Humans), What is the route of exposure? -> Others (eg: injections)
83	**Ladjimi, S.,M'Raihi, L.,Djemel, A.,Mathlouthi, A.,Ben Ayed, F.,Zegaya, M.**. Pleural talc therapy using thoracoscopy for pleural neoplasia (based on 218 cases). [French]. *Revue des Maladies Respiratoires.* 1989. 6:147-150	Level 2, Form Scr - FTxt (Humans), What is the route of exposure? -> Others (eg: injections)
84	**Markman, M.**. Intracavitary chemotherapy. *Critical Reviews in Oncology/Hematology.* 1985. 3:205-233	Level 2, Form Scr - FTxt (Humans), What is the route of exposure? -> Others (eg: injections)
85	**Klemm, C.**. [Not Available]. [Polyglot]. *Helvetica chirurgica acta.* 1947. 14:181-201	Level 2, Form Scr - FTxt (Humans), What is the route of exposure? -> Others (eg: injections)
86	**Alliance for Clinical Trials in, Oncology,National Cancer, Institute**. Standard Chest Tube Compared With a Small Catheter in Treating Malignant Pleural Effusion in Patients With Cancer. *#journal#.* 2005. #volume#:#pages#	Level 2, Form Scr - FTxt (Humans), What is the route of exposure? -> Others (eg: injections)
87	**Eastern Cooperative Oncology, Group,National Cancer, Institute,North Central Cancer Treatment, Group**. Bleomycin, Doxycycline, or Talc in Treating Patients With Malignant Pleural	Level 2, Form Scr - FTxt (Humans), What is the route of exposure? -> Others (eg: injections)

Serial	Reference	Exclusion Criteria
	Effusions. *#journal#.* 2003. #volume#:#pages#	
88	**Alliance for Clinical Trials in, Oncology,National Cancer, Institute,Eastern Cooperative Oncology, Group,Radiation Therapy Oncology, Group,North Central Cancer Treatment, Group**. Talc in Treating Patients With Malignant Pleural Effusion. *#journal#.* 2005. #volume#:#pages#	Level 2, Form Scr - FTxt (Humans), What is the route of exposure? -> Others (eg: injections)
89	**Theagenio Cancer, Hospital**. Video-assisted Hyperthermic Pleural Chemoperfusion vs Talc Pleurodesis for Refractory Malignant Pleural Effusions. *#journal#.* 2014. #volume#:#pages#	Level 2, Form Scr - FTxt (Humans), What is the route of exposure? -> Others (eg: injections)
90	**Papworth Hospital, N. H. S. Foundation Trust**. Video-Assisted Surgery or Talc Pleurodesis in Treating Patients With Malignant Mesothelioma. *#journal#.* 2013. #volume#:#pages#	Level 2, Form Scr - FTxt (Humans), What is the route of exposure? -> Others (eg: injections)
91	**Rambam Health Care, Campus**. Management of Malignant Pleural Effusion - Indwelling Pleural Catheter or Talc Pleurodesis. *#journal#.* 2017. #volume#:#pages#	Level 2, Form Scr - FTxt (Humans), What is the route of exposure? -> Others (eg: injections)
92	**M. D. Anderson Cancer Center,Bard, Incorporated**. Comparison of Progel Sealant to Standard of Care (SOC) for Patients Undergoing Decortication. *#journal#.* 2018. #volume#:#pages#	Level 2, Form Scr - FTxt (Humans), What is the route of exposure? -> Others (eg: injections)
93	**National University Hospital, Singapore**. Multicentre Study Comparing Indwelling Pleural Catheter With Talc Pleurodesis for Malignant Pleural Effusion Management. *#journal#.* 2015. #volume#:#pages#	Level 2, Form Scr - FTxt (Humans), What is the route of exposure? -> Others (eg: injections)

Serial	Reference	Exclusion Criteria
94	**Postgraduate Institute of Medical, Education,Research,**. A Comparative Study of the Safety and Efficacy of Face Talc Slurry and Iodopovidone for Pleurodesis. *#journal#.* #year#. #volume#:#pages#	Level 2, Form Scr - FTxt (Humans), What is the route of exposure? -> Others (eg: injections)
95	**University of Rome Tor, Vergata**. Comparison of Thoracoscopic Talc Pleurodesis by Thoracic Epidural or General Anesthesia. *#journal#.* 2010. #volume#:#pages#	Level 2, Form Scr - FTxt (Humans), What is the route of exposure? -> Others (eg: injections)
96	**Chiang Mai, University**. Efficacy of Iodopovidone Versus Talc in Palliative Malignant Pleural Effusion. *#journal#.* 2017. #volume#:#pages#	Level 2, Form Scr - FTxt (Humans), What is the route of exposure? -> Others (eg: injections)
97	**University of California, Davis**. The First Therapeutic Intervention in Malignant Pleural Effusion Trial. *#journal#.* 2014. #volume#:#pages#	Level 2, Form Scr - FTxt (Humans), What is the route of exposure? -> Others (eg: injections)
98	**Singapore General, Hospital**. A Prospective, Randomized Controlled Trial for a Rapid Pleurodesis Protocol for the Management of Pleural Effusions. *#journal#.* 2013. #volume#:#pages#	Level 2, Form Scr - FTxt (Humans), What is the route of exposure? -> Others (eg: injections)
99	**Istituto Clinico, Humanitas**. 11C-Methionine PET/CT Imaging in Patients Affected by Malignant Pleural Mesothelioma (MPM). *#journal#.* 2017. #volume#:#pages#	Level 2, Form Scr - FTxt (Humans), What is the route of exposure? -> Others (eg: injections)
100	**University of Sao, Paulo**. Talc Pleurodesis in Patients With Recurrent Malignant Pleural Effusion. *#journal#.* 2008. #volume#:#pages#	Level 2, Form Scr - FTxt (Humans), What is the route of exposure? -> Others (eg: injections)
101	**Guy's,,St Thomas, N. H. S. Foundation Trust,CareFusion,**. Out Patient Talc Slurry Via Indwelling Pleural Catheter for Malignant Pleural Effusion Vs Usual	Level 2, Form Scr - FTxt (Humans), What is the route of exposure? -> Others (eg: injections)

Serial	Reference	Exclusion Criteria
	Inpatient Management. *#journal#.* 2020. #volume#:#pages#	
102	**Heidelberg, University**. Tunneled Pleural Catheter in Partially Entrapped Lung. *#journal#.* 2011. #volume#:#pages#	Level 2, Form Scr - FTxt (Humans), What is the route of exposure? -> Others (eg: injections)
103	**University of, Oxford**. Using Thoracic Ultrasound to Predict Pleurodesis Success in Malignant Pleural Effusions: a Pilot Study. *#journal#.* 2016. #volume#:#pages#	Level 2, Form Scr - FTxt (Humans), What is the route of exposure? -> Others (eg: injections)
104	**ISRCTN12709516**. Debulking Surgery and Hyperthermic Chemotherapy for pleural mesothelioma: a pilot study.. *#journal#.* 2017. #volume#:#pages#	Level 2, Form Scr - FTxt (Humans), What is the route of exposure? -> Others (eg: injections)
105	**NCT02825095**. Management of Malignant Pleural Effusion - Indwelling Pleural Catheter or Talc Pleurodesis. *#journal#.* 2016. #volume#:#pages#	Level 2, Form Scr - FTxt (Humans), What is the route of exposure? -> Others (eg: injections)
106	**ISRCTN15503522**. A trial looking at quality of life in the treatment of patients with malignant pleural effusion. *#journal#.* 2015. #volume#:#pages#	Level 2, Form Scr - FTxt (Humans), What is the route of exposure? -> Others (eg: injections)
107	**EUCTR2012-000599-40-GB**. A randomised controlled trial to determine whether the use of indwelling pleural catheters in conjunction with talc slurry is superior to using an indwelling pleural catheter alone, in patients with malignant pleural effusion.. *#journal#.* 2012. #volume#:#pages#	Level 2, Form Scr - FTxt (Humans), What is the route of exposure? -> Others (eg: injections)
108	**ISRCTN47845793**. A randomised trial to determine the best method for delivering talc for the management of malignant pleural effusions in patients with a good performance status. *#journal#.* 2012. #volume#:#pages#	Level 2, Form Scr - FTxt (Humans), What is the route of exposure? -> Others (eg: injections)

Serial	Reference	Exclusion Criteria
109	**ISRCTN34321019**. Prospective randomised controlled trial of video assisted cytoreductive pleurectomy compared to talc pleurodesis in patients with suspected or proven mesothelioma. *#journal#.* 2004. #volume#:#pages#	Level 2, Form Scr - FTxt (Humans), What is the route of exposure? -> Others (eg: injections)
110	**ISRCTN35591640**. A study to compare the efficiency of sterile talc, tetracycline and bleomycin as sclerosing agents for medical pleurodesis in the treatment of malignant pleural effusions. *#journal#.* 2003. #volume#:#pages#	Level 2, Form Scr - FTxt (Humans), What is the route of exposure? -> Others (eg: injections)
111	**NCT00002872**. Bleomycin, Doxycycline, or Talc in Treating Patients With Malignant Pleural Effusions. *#journal#.* 1999. #volume#:#pages#	Level 2, Form Scr - FTxt (Humans), What is the route of exposure? -> Others (eg: injections)
112	**NCT00002622**. Talc in Treating Patients With Malignant Pleural Effusion. *#journal#.* 1999. #volume#:#pages#	Level 2, Form Scr - FTxt (Humans), What is the route of exposure? -> Others (eg: injections)
113	. Evaluating the efficacy of thoracoscopy and talc poudrage versus pleurodesis using talc slurry: a randomised trial to determine the most effective method for the management of malignant pleural effusions in patients with a good performance status. *#journal#.* #year#. #volume#:#pages#	Level 2, Form Scr - FTxt (Humans), What is the route of exposure? -> Others (eg: injections)
114	. The efficacy of Indwelling Pleural Catheter placement versus IPC placement PLUS sclerosant (talc) in patients with malignant pleural effusions managed exclusively as out-patients. *#journal#.* #year#. #volume#:#pages#	Level 2, Form Scr - FTxt (Humans), What is the route of exposure? -> Others (eg: injections)
115	. Randomised controlled trial comparing outpatient management of malignant pleural effusion via an indwelling pleural catheter and talc pleurodesis versus standard inpatient management in	Level 2, Form Scr - FTxt (Humans), What is the route of exposure? -> Others (eg: injections)

Serial	Reference	Exclusion Criteria
	improving health related quality of life. *#journal#.* #year#. #volume#:#pages#	
116	. The Efficacy of Sonographic and Biological Pleurodesis Indicators of Malignant Pleural Effusion (SIMPLE) - a randomised trial. *#journal#.* #year#. #volume#:#pages#	Level 2, Form Scr - FTxt (Humans), What is the route of exposure? -> Others (eg: injections)
117	. Talc Pleurodesis: A Comparative Pilot Study Comparing Doctor vs Nurse Lead Procedure . *#journal#.* #year#. #volume#:#pages#	Level 2, Form Scr - FTxt (Humans), What is the route of exposure? -> Others (eg: injections)
118	. A randomised controlled trial to evaluate whether use of intrapleural urokinase aids the drainage of multi-septated pleural effusion compared to placebo . *#journal#.* #year#. #volume#:#pages#	Level 2, Form Scr - FTxt (Humans), What is the route of exposure? -> Others (eg: injections)
119	. Using Ultrasound to Predict the Results of Draining Pleural Effusions . *#journal#.* #year#. #volume#:#pages#	Level 2, Form Scr - FTxt (Humans), What is the route of exposure? -> Others (eg: injections)
120	. The first Therapeutic Interventions in Malignant Effusion trial . *#journal#.* #year#. #volume#:#pages#	Level 2, Form Scr - FTxt (Humans), What is the route of exposure? -> Others (eg: injections)
121	**Narod, S. A.**. Talc and ovarian cancer. *Gynecologic Oncology.* 2016. 141:410-2	Level 2, Form Scr - FTxt (Humans), What is the study type? -> Editorial/Commentary with no primary ...
122	**Wentzensen, N.,Wacholder, S.**. Talc use and ovarian cancer: epidemiology between a rock and a hard place. *Journal of the National Cancer Institute.* 2014. 106:10	Level 2, Form Scr - FTxt (Humans), What is the study type? -> Editorial/Commentary with no primary ...
123	**Muscat, J.,Huncharek, M.,Cramer, D. W.**. Talc and anti-MUC1 antibodies. *Cancer Epidemiology, Biomarkers &*	Level 2, Form Scr - FTxt (Humans), What is the study type? -> Editorial/Commentary with no primary ...

Serial	Reference	Exclusion Criteria
	Prevention. 2005. 14:2679; author reply 2680	
124	**Cramer, D. W.**. Perineal talc exposure and subsequent epithelial ovarian cancer: a case-control study. *Obstetrics & Gynecology.* 1999. 94:160-1	Level 2, Form Scr - FTxt (Humans), What is the study type? -> Editorial/Commentary with no primary ...
125	**Muscat, J. E.,Wynder, E. L.**. Re: "Perineal powder exposure and the risk of ovarian cancer". *American Journal of Epidemiology.* 1997. 146:786	Level 2, Form Scr - FTxt (Humans), What is the study type? -> Editorial/Commentary with no primary ...
126	**Kasper, C. S.,Chandler, P. J., Jr.**. Possible morbidity in women from talc on condoms. *JAMA.* 1995. 273:846-7	Level 2, Form Scr - FTxt (Humans), What is the study type? -> Editorial/Commentary with no primary ...
127	**Newhouse, M. L.**. Cosmetic talc and ovarian cancer. *Lancet.* 1979. 2:528	Level 2, Form Scr - FTxt (Humans), What is the study type? -> Editorial/Commentary with no primary ...
128	**Trabert, B.**. Body powder and ovarian cancer risk - What is the role of recall bias?. *Cancer Epidemiology Biomarkers and Prevention.* 2016. 25:1369-1370	Level 2, Form Scr - FTxt (Humans), What is the study type? -> Editorial/Commentary with no primary ...
129	**The Lancet, Oncology**. When is a carcinogen not a carcinogen?. *The Lancet Oncology.* 2016. 17:681	Level 2, Form Scr - FTxt (Humans), What is the study type? -> Editorial/Commentary with no primary ...
130	**Cramer, D. W.,Piver, M. S.**. Perineal talc exposure and subsequent epithelial ovarian cancer: A case- control study [4] (multiple letters). *Obstetrics and Gynecology.* 1999. 94:160-161	Level 2, Form Scr - FTxt (Humans), What is the study type? -> Editorial/Commentary with no primary ...
131	**Cook, L. S.,Kamb, M. L.,Weiss, N. S.**. Erratum: Perineal powder exposure and the risk of ovarian cancer (American Journal of Epidemiology (1997) 145 (459-65)). *American Journal of Epidemiology.* 1998. 148:410	Level 2, Form Scr - FTxt (Humans), What is the study type? -> Editorial/Commentary with no primary ...

Serial	Reference	Exclusion Criteria
132	**Ainsworth, S.**. Last word. Not safe for babies' bottoms?. *Practising Midwife.* 2009. 12:42-42	Level 2, Form Scr - FTxt (Humans), What is the study type? -> Editorial/Commentary with no primary ...
133	**Hunn, J.,Rodriguez, G. C.**. Ovarian cancer: etiology, risk factors, and epidemiology. *Clinical Obstetrics & Gynecology.* 2012. 55:3-23	Level 2, Form Scr - FTxt (Humans), What is the study type? -> Literature Review
134	**Cramer, D. W.**. The epidemiology of endometrial and ovarian cancer. *Hematology - Oncology Clinics of North America.* 2012. 26:1-12	Level 2, Form Scr - FTxt (Humans), What is the study type? -> Literature Review
135	**Huncharek, M.,Muscat, J.**. Perineal talc use and ovarian cancer risk: a case study of scientific standards in environmental epidemiology. *European Journal of Cancer Prevention.* 2011. 20:501-7	Level 2, Form Scr - FTxt (Humans), What is the study type? -> Literature Review
136	**Cramer, D. W.,Finn, O. J.**. Epidemiologic perspective on immune-surveillance in cancer. *Current Opinion in Immunology.* 2011. 23:265-71	Level 2, Form Scr - FTxt (Humans), What is the study type? -> Literature Review
137	**Sueblinvong, T.,Carney, M. E.**. Current understanding of risk factors for ovarian cancer. *Current Treatment Options in Oncology.* 2009. 10:67-81	Level 2, Form Scr - FTxt (Humans), What is the study type? -> Literature Review
138	**Sueblinvong, T.,Carney, M. E.**. Ovarian cancer: risks. *Hawaii Medical Journal.* 2009. 68:40-6	Level 2, Form Scr - FTxt (Humans), What is the study type? -> Literature Review
139	**Salehi, F.,Dunfield, L.,Phillips, K. P.,Krewski, D.,Vanderhyden, B. C.**. Risk factors for ovarian cancer: an overview with emphasis on hormonal factors. *Journal of Toxicology & Environmental Health Part B: Critical Reviews.* 2008. 11:301-21	Level 2, Form Scr - FTxt (Humans), What is the study type? -> Literature Review
140	**Langseth, H.,Hankinson, S. E.,Siemiatycki, J.,Weiderpass, E.**. Perineal use of talc and risk of ovarian	Level 2, Form Scr - FTxt (Humans), What is the study type? -> Literature Review

Serial	Reference	Exclusion Criteria
	cancer. *Journal of Epidemiology & Community Health.* 2008. 62:358-60	
141	**Muscat, J. E.,Huncharek, M. S.**. Perineal talc use and ovarian cancer: a critical review. *European Journal of Cancer Prevention.* 2008. 17:139-46	Level 2, Form Scr - FTxt (Humans), What is the study type? -> Literature Review
142	**La Vecchia, C.**. Epidemiology of ovarian cancer: a summary review. *European Journal of Cancer Prevention.* 2001. 10:125-9	Level 2, Form Scr - FTxt (Humans), What is the study type? -> Literature Review
143	**Whysner, J.,Mohan, M.**. Perineal application of talc and cornstarch powders: evaluation of ovarian cancer risk. *American Journal of Obstetrics & Gynecology.* 2000. 182:720-4	Level 2, Form Scr - FTxt (Humans), What is the study type? -> Literature Review
144	**Ness, R. B.,Cottreau, C.**. Possible role of ovarian epithelial inflammation in ovarian cancer. *Journal of the National Cancer Institute.* 1999. 91:1459-67	Level 2, Form Scr - FTxt (Humans), What is the study type? -> Literature Review
145	**Shen, N.,Weiderpass, E.,Antilla, A.,Goldberg, M. S.,Vasama-Neuvonen, K. M.,Boffetta, P.,Vainio, H. U.,Partanen, T. J.**. Epidemiology of occupational and environmental risk factors related to ovarian cancer. *Scandinavian Journal of Work, Environment & Health.* 1998. 24:175-82	Level 2, Form Scr - FTxt (Humans), What is the study type? -> Literature Review
146	**Daly, M.,Obrams, G. I.**. Epidemiology and risk assessment for ovarian cancer. *Seminars in Oncology.* 1998. 25:255-64	Level 2, Form Scr - FTxt (Humans), What is the study type? -> Literature Review
147	**Tortolero-Luna, G.,Mitchell, M. F.**. The epidemiology of ovarian cancer. *Journal of Cellular Biochemistry - Supplement.* 1995. 23:200-7	Level 2, Form Scr - FTxt (Humans), What is the study type? -> Literature Review
148	**Harlow, B. L.,Hartge, P. A.**. A review of perineal talc exposure and risk of ovarian cancer. *Regulatory Toxicology & Pharmacology.* 1995. 21:254-60	Level 2, Form Scr - FTxt (Humans), What is the study type? -> Literature Review

Serial	Reference	Exclusion Criteria
149	**Herbst, A. L.**. The epidemiology of ovarian carcinoma and the current status of tumor markers to detect disease. *American Journal of Obstetrics & Gynecology.* 1994. 170:1099-105; discussion 1105-7	Level 2, Form Scr - FTxt (Humans), What is the study type? -> Literature Review
150	**Lauchlan, S. C.**. The secondary mullerian system revisited. *International Journal of Gynecological Pathology.* 1994. 13:73-9	Level 2, Form Scr - FTxt (Humans), What is the study type? -> Literature Review
151	**Baker, T. R.,Piver, M. S.**. Etiology, biology, and epidemiology of ovarian cancer. *Seminars in Surgical Oncology.* 1994. 10:242-8	Level 2, Form Scr - FTxt (Humans), What is the study type? -> Literature Review
152	**Shoham, Z.**. Epidemiology, etiology, and fertility drugs in ovarian epithelial carcinoma: where are we today?. *Fertility & Sterility.* 1994. 62:433-48	Level 2, Form Scr - FTxt (Humans), What is the study type? -> Literature Review
153	**Wehner, A. P.**. Biological effects of cosmetic talc. *Food & Chemical Toxicology.* 1994. 32:1173-84	Level 2, Form Scr - FTxt (Humans), What is the study type? -> Literature Review
154	**Longo, D. L.,Young, R. C.**. Cosmetic talc and ovarian cancer. *Lancet.* 1979. 2:1011-2	Level 2, Form Scr - FTxt (Humans), What is the study type? -> Literature Review
155	**Pelfrene, A.,Shubik, P.**. [Is talc a carcinogen? Review of current data]. *Nouvelle Presse Medicale.* 1975. 4:801-3	Level 2, Form Scr - FTxt (Humans), What is the study type? -> Literature Review
156	**Jourdan,S.J.,Purdie,D.M.,Whiteman,D.C.,Webb,P.M.**. Risk factors for epithelial ovarian cancer. *Cancer Forum.* 2003. 27:148-151	Level 2, Form Scr - FTxt (Humans), What is the study type? -> Literature Review
157	**Kenemans,P.,Jurgen,M.J.P.**. Oral contraceptives, HRT and ovarian cancer. *113th World Congress on the Menopause.* 2011. #volume#:#pages#	Level 2, Form Scr - FTxt (Humans), What is the study type? -> Literature Review

Serial	Reference	Exclusion Criteria
158	**Reid, B. M.,Permuth, J. B.,Sellers, T. A.**. Epidemiology of ovarian cancer: a review. *Cancer Biology and Medicine.* 2017. 14:9-32	Level 2, Form Scr - FTxt (Humans), What is the study type? -> Literature Review
159	**Silva, E. G.**. The Origin of Epithelial Neoplasms of the Ovary: An Alternative View. *Advances in Anatomic Pathology.* 2016. 23:50-57	Level 2, Form Scr - FTxt (Humans), What is the study type? -> Literature Review
160	**Fiume, M. M.,Boyer, I.,Bergfeld, W. F.,Belsito, D. V.,Hill, R. A.,Klaassen, C. D.,Liebler, D. C.,Marks, J. G.,Shank, R. C.,Slaga, T. J.,Snyder, P. W.,Andersen, F. A.**. Safety Assessment of Talc as Used in Cosmetics. *International Journal of Toxicology.* 2015. 34:66S-129S	Level 2, Form Scr - FTxt (Humans), What is the study type? -> Literature Review
161	**Webb, P. M.**. Environmental (nongenetic) factors in gynecological cancers: Update and future perspectives. *Future Oncology.* 2015. 11:295-307	Level 2, Form Scr - FTxt (Humans), What is the study type? -> Literature Review
162	**Anonymous,**. Mechanistic and other relevant data. *#journal#.* 2010. #volume#:391-405	Level 2, Form Scr - FTxt (Humans), What is the study type? -> Literature Review
163	**McLemore, M. R.,Miaskowski, C.,Aouizerat, B. E.,Chen, L. M.,Dodd, M. J.**. Epidemiological and genetic factors associated with ovarian cancer. *Cancer Nursing.* 2009. 32:281-288	Level 2, Form Scr - FTxt (Humans), What is the study type? -> Literature Review
164	**Dogan, S.,Agic, A.,Eilers, W.,Finas, D.,Diedrich, K.,Hornung, D.**. Endometriosis and risk of malignancy. [German]. *Geburtshilfe und Frauenheilkunde.* 2006. 66:739-744	Level 2, Form Scr - FTxt (Humans), What is the study type? -> Literature Review
165	**Elmasry, K.,Gayther, S. A.**. Ovarian cancer aetiology: Facts and fiction. *Journal of Family Planning and Reproductive Health Care.* 2006. 32:82-86	Level 2, Form Scr - FTxt (Humans), What is the study type? -> Literature Review

Serial	Reference	Exclusion Criteria
166	**Elmasry, K.,Gayther, S. A.**. Genetic mutations in gynaecological cancers. *Reviews in Gynaecological and Perinatal Practice.* 2006. 6:115-125	Level 2, Form Scr - FTxt (Humans), What is the study type? -> Literature Review
167	**Jordan, S. J.,Purdie, D. M.,Whiteman, D. C.,Webb, P. M.**. Risk factors for epithelial ovarian cancer. *Cancer Forum.* 2003. 27:148-151	Level 2, Form Scr - FTxt (Humans), What is the study type? -> Literature Review
168	**Mad'Ar, R.,Straka, S.,Baska, T.**. Is ovarian cancer associated with talcum powder?. [Slovak]. *Hygiena.* 2002. 47:239-242	Level 2, Form Scr - FTxt (Humans), What is the study type? -> Literature Review
169	**Muscat, J. E.,Barish, M.**. Epidemiology of talc exposure and ovarian cancer: A critical assessment. *Comments on Toxicology.* 1998. 6:327-335	Level 2, Form Scr - FTxt (Humans), What is the study type? -> Literature Review
170	**Wehner, A. P.**. Talc: An overview. *Comments on Toxicology.* 1998. 6:309-311	Level 2, Form Scr - FTxt (Humans), What is the study type? -> Literature Review
171	**Wehner, A. P.**. Is cosmetic talc 'safe'?. *Comments on Toxicology.* 1998. 6:337-366	Level 2, Form Scr - FTxt (Humans), What is the study type? -> Literature Review
172	**Shen, N.,Weiderpass, E.,Anttila, A.,Goldberg, M. S.,Vasama-Neuvonen, K. M.,Boffetta, P.,Vainio, H. U.,Partanen, T. J.**. Epidemiology of occupational and environmental risk factors related to ovarian cancer. *Scandinavian Journal of Work, Environment and Health.* 1998. 24:175-182	Level 2, Form Scr - FTxt (Humans), What is the study type? -> Literature Review
173	**Bernal, L. S.,Ramos, C. L. M.**. Risk factors for ovary carcinoma. [Spanish]. *Revista del Instituto Nacional de Cancerologia.* 1996. 42:213-220	Level 2, Form Scr - FTxt (Humans), What is the study type? -> Literature Review
174	**Curie, P.,Sussmann, M.,Treisser, A.,Renaud, R.**. Epidemiologic factors in ovarian carcinoma. [French]. *Revue*	Level 2, Form Scr - FTxt (Humans), What is the study type? -> Literature Review

Serial	Reference	Exclusion Criteria
	Francaise de Gynecologie et d'Obstetrique. 1985. 80:379-382	
175	**Richardson, G. S.,Scully, R. E.,Nikrui, N.,Nelson Jr, J. H.**. Common epithelial cancer of the ovary. (Second of two parts). *New England Journal of Medicine.* 1985. 312:474-482	Level 2, Form Scr - FTxt (Humans), What is the study type? -> Literature Review
176	**Venter, P. F.**. Ovarian epithelial cancer and chemical carcinogenesis. *Gynecologic Oncology.* 1981. 12:281-285	Level 2, Form Scr - FTxt (Humans), What is the study type? -> Literature Review
177	**Terry, K. L.,Karageorgi, S.,Shvetsov, Y. B.,Merritt, M. A.,Lurie, G.,Thompson, P. J.,Carney, M. E.,Weber, R. P.,Akushevich, L.,Lo-Ciganic, W. H.,Cushing-Haugen, K.,Sieh, W.,Moysich, K.,Doherty, J. A.,Nagle, C. M.,Berchuck, A.,Pearce, C. L.,Pike, M.,Ness, R. B.,Webb, P. M.,Australian Cancer, Study,Australian Ovarian Cancer Study, Group,Rossing, M. A.,Schildkraut, J.,Risch, H.,Goodman, M. T.,Ovarian Cancer Association, Consortium**. Genital powder use and risk of ovarian cancer: a pooled analysis of 8,525 cases and 9,859 controls. *Cancer Prevention Research.* 2013. 6:811-21	Level 2, Form Scr - FTxt (Humans), What is the study type? -> Meta-Analysis without Systematic Review
178	**Huncharek, M.,Muscat, J.,Onitilo, A.,Kupelnick, B.**. Use of cosmetic talc on contraceptive diaphragms and risk of ovarian cancer: a meta-analysis of nine observational studies. *European Journal of Cancer Prevention.* 2007. 16:422-9	Level 2, Form Scr - FTxt (Humans), What is the study type? -> Meta-Analysis without Systematic Review
179	**Huncharek, M.,Geschwind, J. F.,Kupelnick, B.**. Perineal application of cosmetic talc and risk of invasive epithelial ovarian cancer: a meta-analysis of 11,933 subjects from sixteen observational studies. *Anticancer Research.* 2003. 23:1955-60	Level 2, Form Scr - FTxt (Humans), What is the study type? -> Meta-Analysis without Systematic Review

Serial	Reference	Exclusion Criteria
180	**Gross, A. J.,Berg, P. H.**. A meta-analytical approach examining the potential relationship between talc exposure and ovarian cancer. *Journal of Exposure Analysis and Environmental Epidemiology.* 1995. 5:181-95	Level 2, Form Scr - FTxt (Humans), What is the study type? -> Meta-Analysis without Systematic Review
181	**Muscat,J.,Huncharek,M.**. Meta-analysis of talc-dusted sanitary napkin and ovarian cancer risk. *114th Biennial Meeting of the International Gynecologic Cancer Society, IGCS 2012.* #year#. #volume#:#pages#	Level 2, Form Scr - FTxt (Humans), What is the study type? -> Meta-Analysis without Systematic Review
182	**Cramer, D. W.,Welch, W. R.,Berkowitz, R. S.,Godleski, J. J.**. Presence of talc in pelvic lymph nodes of a woman with ovarian cancer and long-term genital exposure to cosmetic talc. *Obstetrics & Gynecology.* 2007. 110:498-501	Level 2, Form Scr - FTxt (Humans), What is the study type? -> Other
183	**Cramer, D. W.,Titus-Ernstoff, L.,McKolanis, J. R.,Welch, W. R.,Vitonis, A. F.,Berkowitz, R. S.,Finn, O. J.**. Conditions associated with antibodies against the tumor-associated antigen MUC1 and their relationship to risk for ovarian cancer. *Cancer Epidemiology, Biomarkers & Prevention.* 2005. 14:1125-31	Level 2, Form Scr - FTxt (Humans), What is the study type? -> Other
184	**Meisler, J. G.**. Toward optimal health: the experts discuss ovarian cancer. *Journal of Womens Health & Gender-Based Medicine.* 2000. 9:705-10	Level 2, Form Scr - FTxt (Humans), What is the study type? -> Other
185	**Cramer,D.W.,Titus-Ernstoff,L.,Vitonis,A.F.**. Genital talc use and ovarian cancer: Influence of histologic type and menopausal status on strength and dose response of the association. *102nd Annual Meeting of the American Association for Cancer Research, AACR 2011.* #year#. #volume#:#pages#	Level 2, Form Scr - FTxt (Humans), What is the study type? -> Other

Serial	Reference	Exclusion Criteria
186	**Peres, L. C.,Abbott, S. E.,Alberg, A. J.,Bandera, E. V.,Barnholtz-Sloan, J.,Bondy, M.,Cote, M. L.,Funkhouser, E.,Peters, E. S.,Schwartz, A. G.,Terry, P. D.,Crankshaw, S.,Camacho, F.,Wang, F.,Moorman, P. G.,Schildkraut, J. M.**. Body powder use and ovarian cancer: the African American Cancer Epidemiology Study. *Cancer Research. Conference: 107th Annual Meeting of the American Association for Cancer Research, AACR.* 2016. 76:#pages#	Level 2, Form Scr - FTxt (Humans), What is the study type? -> Other
187	**Gordon, R. E.**. Asbestos contaminated talc as a cause of mesotheliomas in women. *American Journal of Respiratory and Critical Care Medicine. Conference: American Thoracic Society International Conference, ATS.* 2014. 189:#pages#	Level 2, Form Scr - FTxt (Humans), What is the study type? -> Other
188	**Kurta, M.,Diergaarde, B.**. The impact of fertility drug use, infertility, and lifetime ovulation on ovarian cancer risk. *Cancer Prevention Research. Conference: AACR International Conference on Frontiers in Cancer Prevention Research.* 2011. 4:#pages#	Level 2, Form Scr - FTxt (Humans), What is the study type? -> Other
189	**Bailey, J. E.**. Session I. Introduction: overview-scope of the workshop. *Regulatory Toxicology and Pharmacology.* 1995. 21:216-217	Level 2, Form Scr - FTxt (Humans), What is the study type? -> Other
190	**Carr, C. J.**. Talc: Consumer uses and health perspectives. *Regulatory Toxicology and Pharmacology.* 1995. 21:211-215	Level 2, Form Scr - FTxt (Humans), What is the study type? -> Other
191	**Cesario, Sandra K.**. Risks, Triggers, and Protective Factors Related to the Expression of Ovarian Cancer. *JOGNN: Journal of Obstetric, Gynecologic & Neonatal Nursing.* 2015. 44:S65-S65	Level 2, Form Scr - FTxt (Humans), What is the study type? -> Other

Serial	Reference	Exclusion Criteria
192	. On Your Mind. *#journal#.* 2016. 28:12-12	Level 2, Form Scr - FTxt (Humans), What is the study type? -> Other
193	**Ness, R.**. DOES TALC EXPOSURE CAUSE OVARIAN CANCER?: IGCS-0015 Ovarian Cancer. *International Journal of Gynecological Cancer.* 2015. 25 Suppl 1:51	Level 2, Form Scr - FTxt (Humans), What is the study type? -> Systematic Review
194	**Berge, W.,Mundt, K.,Luu, H.,Boffetta, P.**. Genital use of talc and risk of ovarian cancer: a meta-analysis. *Eur J Cancer Prev.* 2017. #volume#:#pages#	Level 2, Form Scr - FTxt (Humans), What is the study type? -> Systematic Review and Meta-Analysis

Level 3 – Reasons for exclusion

Serial	Reference	Exclusion Criteria
1	**Urban, N.,Hawley, S.,Janes, H.,Karlan, B. Y.,Berg, C. D.,Drescher, C. W.,Manson, J. E.,Palomares, M. R.,Daly, M. B.,Wactawski-Wende, J.,O'Sullivan, M. J.,Thorpe, J.,Robinson, R. D.,Lane, D.,Li, C. I.,Anderson, G. L.**. Identifying post-menopausal women at elevated risk for epithelial ovarian cancer. *Gynecologic Oncology.* 2015. 139:253-60	Level 3, Form Scr - FTxt (Humans), What is the study type? -> Other<br>Excluded in favor or Houghton et al, 2014 (refID 10), which had a more detailed analysis of the same study population
2	**Vitonis, A. F.,Titus-Ernstoff, L.,Cramer, D. W.**. Assessing ovarian cancer risk when considering elective oophorectomy at the time of hysterectomy. *Obstetrics & Gynecology.* 2011. 117:1042-50	Level 3, Form Scr - FTxt (Humans), What is the study type? -> Other<br>Excluded as it was a subset of the population included in Cramer et al, 2016 (refID 4)
3	**Cramer, D. W.,Liberman, R. F.,Titus-Ernstoff, L.,Welch, W. R.,Greenberg, E. R.,Baron, J. A.,Harlow, B. L.**. Genital talc exposure and risk of ovarian cancer. *International Journal of Cancer.* 1999. 81:351-6	Level 3, Form Scr - FTxt (Humans), What is the study type? -> Other<br>Excluded as it was a subset of the population included in Cramer et al, 2016 (refID 4)
4	**Cramer, D. W.,Xu, H.**. Epidemiologic evidence for uterine growth factors in the pathogenesis of ovarian cancer. *Annals of Epidemiology.* 1995. 5:310-4	Level 3, Form Scr - FTxt (Humans), What is the study type? -> Other<br>Excluded in favor of Cramer et al, 1982 (refID 123) which provide more relevant information on the outcome of interest
5	**Purdie, D.,Green, A.,Bain, C.,Siskind, V.,Ward, B.,Hacker, N.,Quinn, M.,Wright, G.,Russell, P.,Susil, B.**. Reproductive and other factors and risk of epithelial ovarian cancer: an Australian case-control study. Survey of Women's Health Study Group. *International Journal of Cancer.* 1995. 62:678-84	Level 3, Form Scr - FTxt (Humans), What is the study type? -> Other<br>Excluded in favor of Green et al, 1997 (refID 88), where effect estimates were adjusted for more risk factors including parity.

Supplementary Material V: Newcastle-Ottawa Scale for quality assessment of non-randomized studies

The quality of non-randomized studies included in the review was assessed using the Newcastle-Ottawa scale (NOS) [3], as recommended in the Cochrane Handbook [13]. The Newcastle-Ottawa scale attributes a maximum of nine stars to each study based on its methodological design. The study assessment is divided into three broad components: 1) the selection of the study participants, 2) the comparability of the treatment groups, and 3) the methods for ascertainment of the outcome of interest (cohort studies) or the exposure of interest (case-control studies). Each study can be awarded a maximum of one star for each item in the selection and exposure categories, and a maximum of two stars in the comparability category. Age and parity were the two factors selected to gauge comparability of the exposure groups: studies that reported risk estimates adjusted for these two variables were given two stars for the comparability component. Parity was selected in addition to age, since childbirth has commonly been reported to be an important risk factor for ovarian cancer in the literature [15].

V.1. Newcastle-Ottawa Scale for case-control studies

**Selection**	
1. Is the case definition adequate?	a) Yes, with independent validation *<br>b) Yes, e.g. record linkage or based on self-reports<br>c) No description
2. Representativeness of the cases	a) Consecutive or obviously representative series of cases *<br>b) Potential for selection biases or not stated
3. Selection of Controls	a) Community controls *<br>b) Hospital controls<br>c) No description
4. Definition of Controls	a) No history of disease (endpoint) *<br>b) No description of source
**Comparability**	
5. Comparability of cases and controls on the basis of the design or analysis	a) Study controls for _______________ (Select the most important factor.) *<br>b) Study controls for any additional factor * (This criterion could be modified to indicate specific control for a second important factor.)
**Exposure**	
6. Ascertainment of exposure	a) Secure record (e.g. surgical records) *<br>b) Structured interview where blind to case/control status *<br>c) Interview not blinded to case/control status<br>d) Written self-report or medical record only<br>e) No description
7. Same method of ascertainment for cases and controls	a) Yes *<br>b) No
8. Non-Response rate	a) Same rate for both groups *<br>b) Non-respondents described<br>c) Rate different and no designation

Note: A study can be awarded a maximum of one star for each numbered item within the Selection and Exposure categories. A maximum of two stars can be given for Comparability.

V.2. Newcastle-Ottawa Scale for cohort studies

**Selection**	
1. Representativeness of the exposed cohort	a) Truly representative of the average ______________ (describe) in the community * b) Somewhat representative of the average __________ in the community * c) Selected group of users eg nurses, volunteers d) No description of the derivation of the cohort
2. Selection of the non-exposed cohort	a) Drawn from the same community as the exposed cohort * b) Drawn from a different source c) No description of the derivation of the non-exposed cohort
3. Ascertainment of exposure	a) Secure record (eg surgical records) * b) Structured interview * c) Written self-report d) No description
4. Demonstration that outcome of interest was not present at start of study	a) Yes * b) No
**Comparability**	
5. Comparability of cohorts on the basis of the design or analysis	a) Study controls for _____________ (select the most important factor) * b) Study controls for any additional factor * (This criteria could be modified to indicate specific control for a second important factor.)
**Outcome**	
6. Assessment of outcome	a) Independent blind assessment * b) Record linkage * c) Self-report d) No description
7. Was follow-up long enough for outcomes to occur	a) Yes (select an adequate follow up period for outcome of interest) * b) No
8. Adequacy of follow up of cohorts	a) Complete follow up - all subjects accounted for * b) Subjects lost to follow up unlikely to introduce bias - small number lost - > ____ % (select an adequate %) follow up, or description provided of those lost) * c) Follow up rate < ____% (select an adequate %) and no description of those lost

d) No statement

Note: A study can be awarded a maximum of one star for each numbered item within the Selection and Outcome categories. A maximum of two stars can be given for Comparability.

Supplementary Material VI: Summary of studies included in the systematic review of human epidemiologic studies

Study (Study design)	Sample size (Cases/controls or cases/total cohort)	Eligibility criteria	Confounding adjustment	Adjusted effect estimate(s) (95% CI)	Dose response	Overall author conclusion
Booth et al, (1989), (Case-control) [5]	235/451	**Inclusion**<br>*Cases*<br>- <65 years of age<br>- Diagnosed with ovarian cancer within two years of interview.<br>*Controls*<br>- Women treated at the same participating hospitals<br>**Exclusion**<br>- Had bilateral oophorectomy or had conditions related to reproductive history or OC (all circulatory and gynaecological diseases, gallbladder and thyroid diseases,	Adjusted for: age and socio-economic status.	***By frequency***<br>- Rare use vs never use, RR 0.9 (0.3 - 2.4);<br>- Monthly use vs never use, RR 0.7 (0.3 - 1.8);<br>- Weekly use vs never use, RR 2.0 (1.3 - 3.4);<br>- Daily use vs never use, RR 1.3 (0.8 - 1.9);<br>- P value for trend 0.007;	No trend found	Possible association with > weekly use.

Study (Study design)	Sample size (Cases/controls or cases/total cohort)	Eligibility criteria	Confounding adjustment	Adjusted effect estimate(s) (95% CI)	Dose response	Overall author conclusion
		rheumatoid arthritis, malignant disease of the breast, uterus and bladder, and melanoma).				
Chang and Risch, (1997), (Case-control) [12]	450/564	**Inclusion**<br>Cases<br>- Between 35 and 79 years<br>- Living att western end of Lake Ontario<br>- Histologically confirmed primary invasive or borderline epithelial ovarian tumors diagnosed between 1989 and 1992.<br>Controls<br>- Population based identified through Ontario Ministry of Finance	Matched by: residence and age.<br><br>Adjusted for OC use, full term pregnancies, breastfeeding, tubal ligation, hysterectomy, and having a mother or sister diagnosed with breast	- Regular talc use vs nonuse, OR 1.420 (1.08,1.86);<br><br>***By method of use***<br>- Sanitary napkin talc use vs nonuse, OR 1.26 (0.81,1.96);<br>- After bathing talc use vs nonuse, OR 1.31 (1.00,1.73);<br><br>***By frequency of use after bathing***<br>- <10 times per month vs nonuse, 1.836 (1.24,2.73);	Possible dose response with frequency and duration of use	Positive association

Study (Study design)	**Sample size** (Cases/controls or cases/total cohort)	Eligibility criteria	Confounding adjustment	Adjusted effect estimate(s) (95% CI)	Dose response	Overall author conclusion
		- Randomly selected, living in same area during the same 3-year period as the cases<br>- Matched to case within 15-year age group	or ovarian cancer.	- 10-25 times per month vs nonuse, OR 1.128 (0.74,1.72);<br>- >25 times per month vs nonuse, OR 0.951 (0.61,1.49)<br><br>***By duration (number of years) of use after bathing***<br>- <30 years vs nonuse, OR 1.697 (1.09,2.64);<br>- 30-40 years vs nonuse, OR 1.435 (0.96,2.15);<br>- >40 years vs nonuse, OR 0.865 (0.54,1.38)<br><br>***By number of years used before or after 1970***<br>- Before 1970 use vs nonuse, OR 1.090		

**Study (Study design)**	**Sample size** (Cases/controls or cases/total cohort)	**Eligibility criteria**	**Confounding adjustment**	**Adjusted effect estimate(s) (95% CI)**	**Dose response**	**Overall author conclusion**
				(0.98,1.22) per 10 years of use;<br>- After 1970 use vs nonuse, OR 1.095 (0.89,1.35) per 10 years of use<br><br>***By status of tubal ligation***<br>- Use before tubal ligation/hysterectomy vs nonuse, OR 1.105 (0.99,1.24) per 10 years of use;<br>- Use after tubal ligation/hysterectomy vs nonuse, OR 1.031 (0.82,1.29) per 10 years of use<br><br>***By histology***		

Study (Study design)	Sample size (Cases/controls or cases/total cohort)	Eligibility criteria	Confounding adjustment	Adjusted effect estimate(s) (95% CI)	Dose response	Overall author conclusion
				- Invasive, ever use vs nonuse, OR: 1.513 (1.13,2.02);<br>- Borderline, ever use vs nonuse, OR1.237 (0.76,2.02);<br>- Serous, ever use vs nonuse, OR: 1.336 (0.96,1.85);<br>- Mucinous, ever use vs nonuse, OR: 1.585 (0.97,2.58);<br>- Endometrioid, ever use vs nonuse, OR: 1.671 (1.00,2.79)		
Chen et al, (1992), (Case-control) [7]	112/224	**Inclusion**<br>*Cases:*<br>- Newly diagnosed cases of EOC occurring during the period 1984 – 1986<br>*Controls:*	Adjusted for: education and parity.	Use of dusting powder in women with a history of long-term use (>3 months) vs nonuse, RR: 3.9 (0.9-10.6)	No trend analysis conducted	Positive association with use >3 months.

Study (Study design)	Sample size (Cases/controls or cases/total cohort)	Eligibility criteria	Confounding adjustment	Adjusted effect estimate(s) (95% CI)	Dose response	Overall author conclusion
		- Living in the same locations as cases (using census data)<br>- Matched on age (+/- 1 year)<br>**Exclusion**<br>- Prior serious illness (including gynaecological diseases and a variety of abdominal abnormalities)				
Cook et al, (1997), (Case-control) [13]	313/422	**Inclusion**<br>Cases:<br>- White women<br>- Aged 20-79 years<br>- Diagnosed with ovarian cancer between January 1, 1986, and December 31, 1988<br>- Resided in three counties of western Washington (King,	Adjusted for: age, education, income, marital status, BMI, OC use and parity.	*Adjusted for age:*<br>- Any use of genital powder vs nonuse, RR: 1.5 (1.1-2);<br>- Exclusive use of perineal dusting only vs nonuse, RR: 1.8 (1.2-2.9);<br>- Diaphragm storage In powder only vs nonuse, RR: 0.8 (0.4-1.4);	No trend found	Positive association.

Study (Study design)	Sample size (Cases/controls or cases/total cohort)	Eligibility criteria	Confounding adjustment	Adjusted effect estimate(s) (95% CI)	Dose response	Overall author conclusion
		Pierce, and Snohomish counties).<br>*Controls:*<br>- Identified by random digit dialing;<br>- Aged similar to identified cases and living in the same counties<br>**Exclusion**<br>- Non-white women<br>- >79 years<br>- History of bilateral oophorectomy, uncertainty concerning a history of bilateral oophorectomy<br>- Unknown genital powder use		- Powder on sanitary napkins only vs nonuse, RR: 1.5 (0.6-3.6);<br>- Genital deodorant spray only vs nonuse, RR: 1.5 (0.8-3);<br><br>*Adjusted for age and other method of genital powder applications:*<br>**Perineal dusting:**<br>- Any perineal dusting vs none, RR: 1.6 (1.1-2.3);<br>- Perineal dusting by cumulative lifetime days<br>- *≤2,000 days vs one, RR: 1.8 (0.9-3.5);*<br>- *2,001-5,000 days vs none, RR: 1.6 (0.9-2.9);*<br>- *5,001-10,000 days vs none, RR: 1.2 0 (0.6-2.4);*		

Study (Study design)	**Sample size** (Cases/controls or cases/total cohort)	Eligibility criteria	Confounding adjustment	Adjusted effect estimate(s) (95% CI)	Dose response	Overall author conclusion
				- *>10,000 vs none, RR: 1.8 (0.9-3.4)*<br>**Diaphragm storage in powder**<br>- Any vs none, RR: 1.0 (0.6-1.6);<br>- Cumulative lifetime months of diaphragm storage in powder:<br>- *≤60 months, RR: 1.1 (0.6-1.9);*<br>- *>60 months, RR: 0.8 (0.4-1.7);*<br>- *Usually washed before, any use vs none, RR: 1.4 (0.7-3.0);*<br>- *Not washed before use, any use vs none, RR: 0.7 (0.4-1.4)*<br>**Powder use on sanitary napkins**		

Study (Study design)	Sample size (Cases/controls or cases/total cohort)	Eligibility criteria	Confounding adjustment	Adjusted effect estimate(s) (95% CI)	Dose response	Overall author conclusion
				- Any vs none, RR: 0.9 (0.5-1.5);<br>- Cumulative lifetime months use on sanitary napkins<br>- *≤120, RR: 1.3 (0.7-2.4);*<br>- *>120, RR: 0.5 (0.2-1.1)*<br><br>**Lifetime applications on sanitary napkins**<br>- ≤1,000, RR: 1.3 (0.7-2.5);<br>- >1,000, RR: 0.6 (0.3-1.2)<br><br>**Genital deodorant spray**<br>- Any use vs none, RR: 1.9 (1.1-3.1);<br>- Cumulative lifetime months of genital deodorant spray use		

Study (Study design)	Sample size (Cases/controls or cases/total cohort)	Eligibility criteria	Confounding adjustment	Adjusted effect estimate(s) (95% CI)	Dose response	Overall author conclusion
				- *≤12 months, RR: 1.5 (0.9-2.8);*<br>- *>12 months, RR: 2.7 (1.1-6.6);*<br>- *P value for trend <0.05*<br><br>**Lifetime applications of genital deodorant spray**<br>- ≤500 applications, RR: 1.7 (1.0-2.9);<br>- >500 applications, RR: 2.6 (0.9-7.6);<br>- P value for trend <0.05<br><br>*Adjusted for age:*<br>**By type of powder used with perineal dusting**<br>Exclusive use<br>- Talcum powder only vs none, RR: 1.2 (0.6-2.5);		

Study (Study design)	Sample size (Cases/controls or cases/total cohort)	Eligibility criteria	Confounding adjustment	Adjusted effect estimate(s) (95% CI)	Dose response	Overall author conclusion
				- Baby powder only vs none, RR: 1.4 (0.8-2.4);<br>- Cornstarch only vs none, RR: 0.9 (0.3-2.9);<br>- Deodorizing powder only vs none, RR: 1.0 (0.4-2.6);<br>- Bath/body powder only vs none, RR: 1.6 (0.9-3.0)<br><br>*Adjusted for age and other types of powder used:*<br>Use of<br>- Any talcum powder vs none, RR: 1.6 (0.9-2.8);<br>- Any baby powder vs none, RR: 1.1 (0.7-1.8);<br>- Any cornstarch vs none, RR: 0.8 (0.3-2.0);		

Study (Study design)	Sample size (Cases/controls or cases/total cohort)	Eligibility criteria	Confounding adjustment	Adjusted effect estimate(s) (95% CI)	Dose response	Overall author conclusion
				- Any deodorizing powder vs none, RR: 1.1 (0.6-2.0);<br>- Any bath/body powder vs none, RR: 1.5 (0.9-2.4)<br><br>*Adjusted for age:*<br>**By histology**<br>- Serous tumors, any powder use vs no use, RR: 1.7 (1.1-2.5);<br>- Mucinous tumors, any powder use vs no use, RR: 0.7 (0.4-1.4);<br>- Endometrioid tumors, any powder use vs no use, RR: 1.2 (0.6-2.3);<br>- Other tumors, any powder use vs no use, RR: 1.8 (1.1-2.8)		

Study (Study design)	Sample size (Cases/controls or cases/total cohort)	Eligibility criteria	Confounding adjustment	Adjusted effect estimate(s) (95% CI)	Dose response	Overall author conclusion
Cramer et al, (1982), (Case-control) [2]	215/215	**Inclusion**<br>*Cases:*<br>- English speaking women;<br>- 18-80 years;<br>- Living in Massachusetts;<br>- Diagnosed with ovarian cancer between November 1978 and September 1981<br>*Controls:*<br>- Identified through the Massachusetts Town Books;<br>- Randomly selected randomly and matched to cases by precinct of residence, race, and age (+/- 2 years)<br><br>**Exclusion**	Adjusted for: parity, menopausal status, religion, marital status, educational level, ponderal index, age at menarche, OC use, menopausal hormone use, and smoking.	*Any talc use vs nonuse* RR: 1.61 (1.04 - 2.49) *(adjusted for all variables listed)*<br><br>*Adjusted for parity and menopausal status:*<br>- Had pelvic surgery, use vs nonuse, RR: 1.17 (0.76 - 1.79);<br>- Had pelvic surgery prior to 1950, use vs nonuse, RR: 1.12 (0.69 - 1.82);<br>- Talc use on condoms vs nonuse, RR: 0.77 (0.41 - 1.44);<br>- Talc use on diaphragm vs nonuse, RR: 1.19 (0.69 - 2.05);<br>Any perineal exposure vs nonuse, RR: 1.92 (1.27 - 2.89);<br>- Use as dusting powder but not on napkins or on	No trend analysis conducted)	Positive association

Study (Study design)	Sample size (Cases/controls or cases/total cohort)	Eligibility criteria	Confounding adjustment	Adjusted effect estimate(s) (95% CI)	Dose response	Overall author conclusion
		Cases:<br>- Women whose statements could not be verified were included or excluded on the basis of their recollection of the surgery.<br>- (Cases were not excluded due to prior hysterectomy or other types of pelvic operations.<br><br>*Controls*<br>- History of bilateral salpingo-oophorectomy		napkins but not as dusting powder vs nonuse, RR: 1.55 (0.98 - 2.4);<br>- Use on napkins and as dusting powder vs nonuse, RR: 3.28 (1.68 - 6.42)		
Cramer et al. (2016), (Case-	2,041/2,100	**Inclusion**<br>Cases<br>- Diagnosed with epithelial tumors of ovarian, primary	Matched by: age, study center and phase.<br><br>Adjusted	Talc use vs nonuse, adjusted for all variables, OR 1.32 (1.15, 1.53)<br>The following were adjusted for age, study	Significant trend for years since exposure, frequency and duration	Positive association

Study (Study design)	Sample size (Cases/controls or cases/total cohort)	Eligibility criteria	Confounding adjustment	Adjusted effect estimate(s) (95% CI)	Dose response	Overall author conclusion
control) **[52]**		peritoneal, or Fallopian tube origin<br><br>Controls:<br>- Matched for age, study center and study phase<br><br>**Exclusion**<br>- Died, moved outside study area or did not having a working telephone number;<br>- Cases with non-epithelial or mixed mesodermal ovarian tumors;<br>- Cases with non-ovarian primary tumors;<br>- Controls who do not speak English;<br>- Controls who have had bilateral oophorectomy;	for: race, Jewish ethnicity, BMI, height, weight, parity, breastfeeding, OC use, IUD use, ovulatory cycles, endometriosis, family history of ovarian or early onset breast cancer, personal history of breast cancer, hysterectomy or tubal ligation,	center and study phase (matching variables<br>**Personal use**<br>- Body use only vs nonuse, OR: 0.99 (0.84, 1.16);<br>- Genital use only vs nonuse, OR: 1.42 (1.04, 1.96);<br>- Body and genital use vs nonuse, OR: 1.30 (1.12, 1.52)<br><br>**Potential exposure in women with no personal use**<br>- Diaphragm only, OR: 0.73 (0.57, 0.93);<br>- Condoms, with or without diaphragm vs nouse, OR: 0.82 (0.66, 1.01);<br>- Partner use, with or without diaphragm or	of use, and number of lifetime applications	

**Study (Study design)**	**Sample size** (Cases/controls or cases/total cohort)	**Eligibility criteria**	**Confounding adjustment**	**Adjusted effect estimate(s) (95% CI)**	**Dose response**	**Overall author conclusion**
		- Controls seriously ill	menopausal status, HT use, smoking, alcohol use, asthma, acetaminophen use, and aspirin or ibuprofen use.	condoms, OR: 0.96 (0.68, 1.35)<br><br>**Type of genital powder used**<br>- Any genital powder use vs nonuse, OR: 1.33 (1.16, 1.52);<br>- Cornstarch use only vs nonuse, OR: 0.58 (0.19, 1.74);<br>- Johnson and Johnson Baby Powder or Shower to Shower vs nonuse, OR: 1.30 (1.10, 1.54);<br>- Other brand(s) vs nonuse, OR: 1.35 (1.12, 1.64)<br><br>**Age first used genital powder**<br>- <20 vs never use, OR: 1.19 (1.01, 1.41);		

Study (Study design)	Sample size (Cases/controls or cases/total cohort)	Eligibility criteria	Confounding adjustment	Adjusted effect estimate(s) (95% CI)	Dose response	Overall author conclusion
				- 20–29 vs never use, OR: 1.71 (1.34, 2.17);<br>- ≥30 vs never use, OR: 1.31 (0.95, 1.80)<br><br>**Time since exposure ended**<br><br>- ≥35 years vs no use, OR: 1.18 (0.79, 1.75);<br>- 25–34 years vs no use, OR: 1.24 (0.91, 1.70);<br>- 15–24 years vs no use, OR: 1.30 (0.94, 1.80);<br>- 5–14 years vs no use, OR: 1.36 (1.00, 1.85);<br>- Currently using or recently stopped vs no use, OR: 1.38 (1.15, 1.65);<br>- P value for trend <0.0001<br><br>**Frequency of use**		

Study (Study design)	Sample size (Cases/controls or cases/total cohort)	Eligibility criteria	Confounding adjustment	Adjusted effect estimate(s) (95% CI)	Dose response	Overall author conclusion
				- 1-7 days per month vs no use, OR: 1.17 (0.96, 1.44);<br>- 8–29 days per month vs no use, OR: 1.37 (1.05, 1.78);<br>- ≥30 days per month vs no use, OR: 1.46 (1.20, 1.78);<br>- P value for trend <0.0001<br><br>**Years used**<br>- <8 vs never used, OR: 1.31 (1.03, 1.68);<br>- 8–19 vs never used, OR: 1.31 (1.02, 1.68);<br>- 20–35 vs never used, OR: 1.35 (1.07, 1.70);<br>- >35 vs never used, OR: 1.33 (1.03, 1.71);<br>- P value for trend 0.002<br><br>**Months per year of use**		

Study (Study design)	**Sample size** (Cases/controls or cases/total cohort)	Eligibility criteria	Confounding adjustment	Adjusted effect estimate(s) (95% CI)	Dose response	Overall author conclusion
				- 1–3 months per year vs no use, OR: 1.11 (0.77, 1.61);<br>- 4–11 months per year vs no use, OR: 1.13 (0.77, 1.66);<br>- 12 months per year vs no use, OR: 1.35 (1.09, 1.67);<br>- P value for trend 0.006<br><br>**Total genital talc applications among only those who reported months per year of use**<br>- ≤360 apps (equivalent to 1 year of daily use), OR: 1.10 (0.83, 1.47);<br>- 361–1,800 apps (equivalent to >1–5 years of daily use), OR: 1.38 (1.01, 1.88);		

**Study (Study design)**	**Sample size** (Cases/controls or cases/total cohort)	**Eligibility criteria**	**Confounding adjustment**	**Adjusted effect estimate(s) (95% CI)**	**Dose response**	**Overall author conclusion**
				- 1,801–7,200 apps (equivalent to >5–20 years of daily use), OR: 1.16 (0.80, 1.66);<br>- >7,200 apps (equivalent to >20 years of daily use), OR: 1.49 (1.06, 2.10);<br>- P value for trend 0.02<br><br>**Total genital talc applications among all (assuming 12 months/year when missing months per year of use)**<br>- ≤360 apps (equivalent to 1 year of daily use), OR: 1.15 (0.89, 1.47);<br>- 361–1,800 apps (equivalent to >1–5 years of daily use), OR: 1.36 (1.06, 1.75);		

Study (Study design)	Sample size (Cases/controls or cases/total cohort)	Eligibility criteria	Confounding adjustment	Adjusted effect estimate(s) (95% CI)	Dose response	Overall author conclusion
				- 1,801–7,200 apps (equivalent to >5–20 years of daily use), OR: 1.41 (1.10, 1.80);<br>- >7,200 apps (equivalent to >20 years of daily use), OR: 1.39 (1.11, 1.75);<br>- P value for trend 0.003<br><br>**By age**<br>- Talc use vs nonuse, <50, OR: 1.42 (1.13, 1.80);<br>- Talc use vs nonuse, 50–64, OR: 1.25 (1.03, 1.53);<br>- Talc use vs nonuse, ≥65, OR: 1.35 (0.98, 1.85)<br><br>**By study center**<br>- Talc use vs nonuse, New Hampshire, OR: 1.52 (1.08, 2.14);		

Study (Study design)	Sample size (Cases/controls or cases/total cohort)	Eligibility criteria	Confounding adjustment	Adjusted effect estimate(s) (95% CI)	Dose response	Overall author conclusion
				- Talc use vs nonuse, Massachusetts, OR: 1.29 (1.11, 1.50)<br><br>**By study phase**<br>- Talc use vs nonuse, first phase, OR: 1.71 (1.27, 2.30);<br>- Talc use vs nonuse, second phase, OR: 1.23 (0.97, 1.55);<br>- Talc use vs nonuse, third phase, OR: 1.25 (1.02, 1.54)<br><br>**By race**<br>- Talc use vs nonuse, White, OR: 1.35 (1.17, 1.55) ;<br>- Talc use vs nonuse, African American, OR: 5.08 (1.32, 19.6);		

Study (Study design)	Sample size (Cases/controls or cases/total cohort)	Eligibility criteria	Confounding adjustment	Adjusted effect estimate(s) (95% CI)	Dose response	Overall author conclusion
				- Talc use vs nonuse, Hispanic, OR: 1.10 (0.30, 4.12);<br>- Talc use vs nonuse, Asian, OR: 0.04 (0.01, 0.34)<br><br>**By BMI**<br>- Talc use vs nonuse, <24.9, OR: 1.25 (1.03, 1.53);<br>- Talc use vs nonuse, ≥24.9, OR: 1.38 (1.14, 1.67)<br><br>**By height (m)**<br>- Talc use vs nonuse, <1.63 OR:1.28 (1.06, 1.56);<br>- Talc use vs nonuse, ≥1.63, OR: 1.37 (1.13, 1.66)		

**Study (Study design)**	**Sample size** (Cases/controls or cases/total cohort)	**Eligibility criteria**	**Confounding adjustment**	**Adjusted effect estimate(s) (95% CI)**	**Dose response**	**Overall author conclusion**
				**By weight (lbs)**<br>- Talc use vs nonuse, <148, OR: 1.24 (1.01, 1.52); Talc use vs nonuse, ≥148, OR: 1.38 (1.15, 1.66)<br><br>**By parity**<br>- Talc use vs nonuse, nulliparous, OR: 1.28 (0.96, 1.71);<br>- Talc use vs nonuse, parous, OR: 1.34 (1.15, 1.57)<br><br>**By ever breastfed**<br>- Talc use vs nonuse, never breastfed, OR: 1.21 (1.01, 1.45);		

Study (Study design)	Sample size (Cases/controls or cases/total cohort)	Eligibility criteria	Confounding adjustment	Adjusted effect estimate(s) (95% CI)	Dose response	Overall author conclusion
				- Talc use vs nonuse, breastfed, OR: 1.48 (1.19, 1.85)<br><br>**By oral contraceptive (OC) use**<br>- Talc use vs nonuse, No OC use or <3 months; OR: 1.25 (1.01, 1.55);<br>- Talc use vs nonuse, ≥3 months OC use, OR: 1.39 (1.16, 1.67)<br><br>**By intrauterine device use**<br>- Talc use vs nonuse, no IUD use, OR: 1.35 (1.16, 1.56);<br>- Talc use vs nonuse, IUD use, OR: 1.20 (0.85, 1.70)		

Study (Study design)	Sample size (Cases/controls or cases/total cohort)	Eligibility criteria	Confounding adjustment	Adjusted effect estimate(s) (95% CI)	Dose response	Overall author conclusion
				**By number of ovulatory cycles**<br>- Talc use vs nonuse, <366 cycles, OR: 1.28 (1.02, 1.61);<br>- Talc use vs nonuse, ≥366 cycles, OR: 1.37 (1.13, 1.65)<br><br>**By endometriosis or painful periods**<br>- Talc use vs nonuse, no endo or painful periods, OR: 1.29 (1.08, 1.55);<br>- Talc use vs nonuse, had endo or painful periods, OR: 1.35 (1.09, 1.67)<br><br>**By Jewish ethnicity**		

Study (Study design)	Sample size (Cases/controls or cases/total cohort)	Eligibility criteria	Confounding adjustment	Adjusted effect estimate(s) (95% CI)	Dose response	Overall author conclusion
				- Talc use vs nonuse, no, OR: 1.33 (1.15, 1.53);<br>- Talc use vs nonuse, yes, OR: 1.39 (0.83, 2.33)<br><br>**By family history of ovarian or early onset breast cancer**<br>- Talc use vs nonuse, no, OR: 1.34 (1.16, 1.55);<br>- Talc use vs nonuse, Yes OR: 1.19 (0.73, 1.93)<br><br>**By personal history of breast cancer**<br>- Talc use vs nonuse, No, OR: 1.38 (1.20, 1.59);<br>- Talc use vs nonuse, Yes, OR: 0.67 (0.37, 1.22)		

Study (Study design)	**Sample size** (Cases/controls or cases/total cohort)	Eligibility criteria	Confounding adjustment	Adjusted effect estimate(s) (95% CI)	Dose response	Overall author conclusion
				**By hysterectomy or tubal ligation**<br>- Talc use vs nonuse, no, OR: 1.22 (1.04, 1.43);<br>- Talc use vs nonuse, yes, OR: 1.73 (1.31, 2.27)<br><br>**By menopausal status and HT**<br>- Talc use vs nonuse, premenopausal, OR: 1.41 (1.13, 1.75);<br>- Talc use vs nonuse, postmenopausal, no HT, OR: 0.97 (0.78, 1.20);<br>- Talc use vs nonuse, postmenopausal, HT, OR: 2.21 (1.63, 3.00)		

Study (Study design)	Sample size (Cases/controls or cases/total cohort)	Eligibility criteria	Confounding adjustment	Adjusted effect estimate(s) (95% CI)	Dose response	Overall author conclusion
				**By current smoking status**<br>- Talc use vs nonuse, no, OR: 1.35 (1.16, 1.56);<br>- Talc use vs nonuse, yes, OR: 1.19 (0.84, 1.69)<br><br>**By ever smoked**<br>- Talc use vs nonuse, no, OR: 1.34 (1.10, 1.64);<br>- Talc use vs nonuse, yes, OR: 1.31 (1.09, 1.58)<br><br>**By asthma**<br>- Talc use vs nonuse, no, OR: 1.34 (1.16, 1.55);<br>- Talc use vs nonuse, yes, OR: 1.25 (0.78, 2.01)<br><br>**Alcohol (grams per day)**		

Study (Study design)	Sample size (Cases/controls or cases/total cohort)	Eligibility criteria	Confounding adjustment	Adjusted effect estimate(s) (95% CI)	Dose response	Overall author conclusion
				- Talc use vs nonuse, ≤2.32, OR: 1.19 (0.98, 1.45);<br>- Talc use vs nonuse, >2.32, OR: 1.43 (1.17, 1.75)<br><br>**Any acetaminophen use**<br>- Talc use vs nonuse, no, OR: 1.30 (1.10, 1.53);<br>- Talc use vs nonuse, yes, OR: 1.41 (1.09, 1.82)<br><br>**Any aspirin or ibuprofen use**<br>- Talc use vs nonuse, no, OR: 1.32 (1.10, 1.59);<br>- Talc use vs nonuse, yes, OR: 1.36 (1.11, 1.68)		
Gates et al, (2008),	NECC: 1,175/1,202	**Inclusion**<br>***New England Study***	Adjusted for: age, study center	- Regular use (>1/week) vs nonuse, all cases,	Significant trend for	Positive association

Study (Study design)	Sample size (Cases/controls or cases/total cohort)	Eligibility criteria	Confounding adjustment	Adjusted effect estimate(s) (95% CI)	Dose response	Overall author conclusion
(Case-control) [43]	NHS: 210/600	*Cases:*<br>- Diagnosed with EOC identified through hospital tumor boards and state cancer registries.<br><br>*Controls*<br>- Identified through RDD, drivers' license records, and Massachusetts' town resident lists;<br>- Matched to cases by age and state of residence.<br><br>***NHS Study***<br>*Cases*<br>- New cases of ovarian cancer, diagnosed before June 1, 2004;	(NECC only), duration of OC use, parity, tubal ligation, BMI and duration of post menopausal hormone (PMH) use.	pooled, RR: 1.36 (1.14-1.63);<br>- Regular use (>1/week) vs nonuse, serous invasive tumors, pooled, RR: 1.60 (1.26-2.02)<br><br>**By frequency (all cases)**<br>- <1/week vs nonuse, pooled, RR: 0.82 (0.55-1.20);<br>- 1-6 times/ week vs nonuse, pooled, RR: 1.26 (0.97-1.63);<br>- Daily use vs nonuse, pooled, RR: 1.41 (1.14-1.76)<br>- P-value for trend <0.001<br><br>***By Frequency (serous invasive tumors)***<br>- <1/week vs nonuse, pooled, RR: 0.70 (0.39-1.24);	frequency of use	

Study (Study design)	Sample size (Cases/controls or cases/total cohort)	Eligibility criteria	Confounding adjustment	Adjusted effect estimate(s) (95% CI)	Dose response	Overall author conclusion
		- No history of a prior cancer, other than non-melanoma skin cancer;<br>- Cases were identified on each questionnaire and also obtained information on deaths due to ovarian cancer through family members, the National Death Index, and the U.S. Postal Service.<br><br>*Controls*<br>- Randomly selected women who gave a buccal cell or blood specimen;<br>- No history of cancer, other than non-melanoma skin cancer;<br>- Matched to cases on month and year of birth,		- 1-6/week vs nonuse, RR: 1.58 (1.12-2.21);<br>- Daily use vs nonuse, RR: 1.56 (1.17-2.08)<br>- P value for trend <0.001;		

Study (Study design)	Sample size (Cases/controls or cases/total cohort)	Eligibility criteria	Confounding adjustment	Adjusted effect estimate(s) (95% CI)	Dose response	Overall author conclusion
		DNA type, and menopausal status at diagnosis.<br><br>**Exclusion**<br>***New England Study***<br>*Cases*<br>- Did not speak English;<br>- Had a non–ovarian primary tumor after review;<br>- Lived outside the study area.<br><br>*Controls*<br>- Previous oophorectomy.<br><br>***NHS Study***<br>*Cases*				

**Study (Study design)**	**Sample size** (Cases/controls or cases/total cohort)	**Eligibility criteria**	**Confounding adjustment**	**Adjusted effect estimate(s) (95% CI)**	**Dose response**	**Overall author conclusion**
		- No history of a prior cancer, other than non-melanoma skin cancer.<br><br>*Controls*<br>- Bilateral oophorectomy;<br>- history of cancer (other than non-melanoma skin cancer);<br>- Unavailability of genotyping data;<br>- Later diagnosis of ovarian cancer.				
Gates et al, (2010), (cohort) [54]	797/108,870	**Inclusion**<br>-diagnosed with EOC or primary peritoneal cancer between baseline and June 2006, and confirmed by pathology report review.<br><br>**Exclusion**	Reported findings were not adjusted.	***By histology***<br>- Talc use (≥once/week) vs. <once/week, all cancers, unadjusted RR: 1.06 (0.89 - 1.28);<br>- Talc use (≥once/week) vs. <once/week, serous invasive, unadjusted RR: 1.06 (0.84 - 1.35);	No trend analysis conducted	Possible association that varies by subtype. No association with

Study (Study design)	Sample size (Cases/controls or cases/total cohort)	Eligibility criteria	Confounding adjustment	Adjusted effect estimate(s) (95% CI)	Dose response	Overall author conclusion
		- bilateral oophorectomy<br>- menopause due to pelvic irradiation<br>-cancer other than nonmelanoma skin cancer.<br>- missing data on any exposure of interest except breastfeeding duration, talc use, and family history of ovarian cancer and age at natural menopause		Talc use (≥ once/week) vs. <once/week, endometrioid, unadjusted RR: 1.06 (0.66 - 1.69);<br>- Talc use (≥once/week) vs. <once/week, mucinous, unadjusted RR: 1.50 (0.84 - 2.66);		mucinous tumors.
Gertig et al, (2000), (cohort) [20]	307/78,630	**Inclusion**<br>- Residing in one of the 11 states;<br>- Between 30-55 years;<br>- Completed questionnaires on medical history and potential cancer risk factors;<br>- Reported diagnosis of epithelial ovarian	Adjusted for: age, parity, OC use, BMI, tubal ligation, smoking and postmenopausal	Ever perineal use vs never, RR 1.09 (0.86,1.37);<br>***By frequency***<br>- <1/week vs never, RR: 1.14 (0.81,1.59);<br>- 1–6/week vs never, RR: 0.99 (0.67,1.46);<br>- Daily vs never, RR: 1.12 (0.82,1.55)	No trend found	Possible association (modest increase for serous invasive subtype).

Study (Study design)	Sample size (Cases/controls or cases/total cohort)	Eligibility criteria	Confounding adjustment	Adjusted effect estimate(s) (95% CI)	Dose response	Overall author conclusion
		cancer and confirmed by medical record review or death certification;<br>- Occurring between 1982 and 1996<br><br>**Exclusion**<br>- No response on talc exposure questions;<br>- Cancer diagnosis other than nonmelanoma skin cancer before 1982;<br>- Bilateral oophorectomy;<br>- Surgery with one or two ovaries removed;<br>- History of radiation therapy.	hormone use.	***By method of use***<br>- Ever use sanitary napkins vs never, RR: 0.89 (0.61,1.28);<br>- Ever perineal use vs never, RR: 1.09 (0.86,1.37);<br>- Either perineal and sanitary napkins, ever use vs never use, RR: 1.15 (0.90,1.46);<br>- Use on both sanitary napkins and perineum, RR: 0.90 (0.59,1.37)<br><br>***By histology***<br>- All serous cancers, ever vs never, RR: 1.26 (0.94,1.69);<br>- Serous invasive cancers, ever vs never, RR: 1.40 (1.02,1.91);<br>- Endometrioid cancers, ever vs never, RR: 0.91 (0.49,1.87);		

**Study (Study design)**	**Sample size** (Cases/controls or cases/total cohort)	**Eligibility criteria**	**Confounding adjustment**	**Adjusted effect estimate(s) (95% CI)**	**Dose response**	**Overall author conclusion**
				- Mucinous cancers, ever vs never, RR: 0.93 (0.53,1.66)		
Godard et al, (1998), (Case-control) [15]	153/152	**Inclusion**<br>*Cases:*<br>- Identified from two oncology clinics from Montreal hospitals;<br>- Diagnosis with ovary cancer in 1995 and 1996<br>*Controls:*<br>- Population based;<br>- Identified through modified RDD (from same telephone directlory page as case);<br>- Matched by age (+/- 1 year)<br><br>**Exclusion**<br>*Cases:*	Adjusted factors not clearly reported in paper. Authors report that findings were adjusted for all factors that were significant in univariate analyses: OC use, age at menarche, age at last childbirth, interval	- All cases, ever vs never, RR: 2.49 (0.94,6.58);<br>- Sporadic cases, ever vs never, RR: 2.45 (0.85,7.07);<br>- Familial cases, ever vs never, RR: 3.25 (0.85,12.4)	No trend analysis conducted	No association

Study (Study design)	Sample size (Cases/controls or cases/total cohort)	Eligibility criteria	Confounding adjustment	Adjusted effect estimate(s) (95% CI)	Dose response	Overall author conclusion
		- Refused to be interviewed;<br>- Died before interview;<br>- Unavailable or lost to followup;<br>- Ovarian tumor was non-epithelial in origin	between 1st and last childbirth.			
Gonzalez et al., (2016), (cohort) [56]	154/41,654	**Inclusion**<br>- 35 to 74 years old;<br>- No history of breast cancer;<br>- Has a sister diagnosed with breast cancer<br><br>**Exclusion**<br>- Bilateral oophorectomy;<br>- History of ovarian cancer prior to enrollment;<br>- Missing follow-up information	Adjusted for: Patency, menopausal status, duration of OC use, parity, race, and BMI.	- Ever vs never, HR: 0.73 (0.44, 1.2);<br>- Pre-pubertal use of talc, ever vs never, HR: 1.1 (0.74, 1.7)] ;	No trend analysis conducted	No association

**Study (Study design)**	**Sample size** (Cases/controls or cases/total cohort)	**Eligibility criteria**	**Confounding adjustment**	**Adjusted effect estimate(s) (95% CI)**	**Dose response**	**Overall author conclusion**
Green et al, (1997), (Case-control) [41]	824/860	**Inclusion**<br>*Cases:*<br>- Aged 18–79;<br>- Diagnosed with ovarian cancer;<br>- Living in the states of New South Wales, Victoria and Queensland<br><br>*Controls*:<br>- Selected from the electoral roll;<br>- Matched on age and urban/rural district of residence.<br>**Exclusion**<br>Prior history of ovarian cancer or bilateral oophorectomy	Adjusted for: age, parity, OC use, education, BMI, smoking and history of ovarian cancer in a first-degree relative.	- Perineal use vs nonuse, RR: 1.3 (1.1, 1.6);<br>- Prior tubal ligation, use vs nonuse, RR: 0.61 (0.46, 0.85);<br>- Prior hysterectomy, use vs nonuse, RR: 0.64 (0.48, 0.85);<br>- No prior surgery, use vs nonuse, RR: 1.3 (1.0, 1.7);	No trend found	Positive association

**Study (Study design)**	**Sample size** (Cases/controls or cases/total cohort)	**Eligibility criteria**	**Confounding adjustment**	**Adjusted effect estimate(s) (95% CI)**	**Dose response**	**Overall author conclusion**
Harlow et al, (1989), (Case-control) [6]	116/158	**Inclusion**<br>*Cases:*<br>- Age 20-79 years;<br>- Diagnosed with a serous or mucinous borderline ovarian tumor during 1980-1985;<br>- Residents of three urban counties of western Washington State<br><br>*Controls:*<br>- White women;<br>- Identified by RDD;<br>- Matched to cases on age and residence<br><br>**Exclusion**<br>- Non-white controls;	Adjusted for: age, parity, and OC use.	Any perineal powder exposure vs none, RR 1.1 (0.7 - 2.1);<br><br>***By method of use***<br>- Diaphragm storage only, use vs nonuse, RR: 0.5 (0.2 - 1.4);<br>- Diaphragm storage only or with other methods, use vs nonuse, RR: 0.5 (0.2 - 1.3)];<br>- After bathing only, use vs nonuse, RR: 1.2 (0.6 - 2.6);<br>- After bathing only or with other methods, use vs nonuse, RR: 1.3 (0.8 - 2.7);<br>- Sanitary napkins only, use vs nonuse, RR: 2.2 (0.8 - 19.8);<br>- Sanitary napkins only or with other methods use	No trend analysis conducted	No association

Study (Study design)	Sample size (Cases/controls or cases/total cohort)	Eligibility criteria	Confounding adjustment	Adjusted effect estimate(s) (95% CI)	Dose response	Overall author conclusion
		- Underwent bilateral oophorectomy.		vs nonuse, RR: 1.9 (0.9 - 6.9);<br>- After bathing and on sanitary napkins, use vs nonuse, RR: 2.2 (0.9 - 19.8)<br><br>***By type of powder used***<br>- Cornstarch only, use vs nonuse, RR: 0.8 (0.2 - 3.8);<br>- Baby powder only, use vs nonuse, RR: 0.8 (0.4 - 1.9);<br>- Baby powder only or combined use, use vs nonuse, RR: 9.0 (0.5 - 2.0);<br>- Talc, unspecified (no combined use), use vs nonuse, RR: 1.0 (0.4 - 2.4);		

Study (Study design)	Sample size (Cases/controls or cases/total cohort)	Eligibility criteria	Confounding adjustment	Adjusted effect estimate(s) (95% CI)	Dose response	Overall author conclusion
				- Deodorizing powder only, use vs nonuse, RR: 3.5 (1.2 - 28.7);<br>- Deodorizing powder only or combined use, use vs nonuse, RR: 2.8 (1.1 - 11.7);<br><br>***Method and type of powder used***<br>- Powder use after bathing, any use of deodorizing powder, RR: 3.1 (0.8 - 10.9);<br>- Powder use after bathing, no use of deodorizing powder, RR: 1.1 (0.5 - 2.4);<br>- Any powder use on sanitary napkins, any use of deodorizing powder, RR: 2.6 (0.9 - 22.4);<br>- Any powder use on sanitary napkins, no use		

Study (Study design)	Sample size (Cases/controls or cases/total cohort)	Eligibility criteria	Confounding adjustment	Adjusted effect estimate(s) (95% CI)	Dose response	Overall author conclusion
				of deodorizing powder, RR: 1.5 (4.0 - 6.5)		
Harlow et al, (1992), (Case-control) [8]	235/239	**Inclusion**<br>*Cases*<br>- Aged 18-76 years;<br>- Diagnosed with borderline or malignant epithelial ovarian cancer at one of ten participating hospitals in the Boston metropolitan area.<br><br>*Controls*<br>- Selected from the Massachusetts Town Books;<br>- Matched to cases on residence, race, age (+/- 2 years)	Adjusted for: age, parity, education, marital status, religion, use of sanitary napkins, douching and weight.	Any perineal use vs none, OR 1.5 (1.0 - 2.1);<br><br>***By method of application***<br>- Only sanitary napkins and/or underwear vs none, OR: 1.1 (0.4 - 2.8);<br>- Partner use or applications to diaphragm (includes combinations with sanitary napkins or underwear) vs none, OR: 1.2 (0.6 - 2.4);<br>- Dusting powder to perineum, includes combinations with sanitary napkins or underwear) vs none, OR: 1.7 (1.1 - 2.7)	Significant trend for monthly frequency of use	Positive associations in certain subgroups (talc used before 1960, women <50 years old, women with 1 or 2 live births)

Study (Study design)	Sample size (Cases/controls or cases/total cohort)	Eligibility criteria	Confounding adjustment	Adjusted effect estimate(s) (95% CI)	Dose response	Overall author conclusion
		**Exclusion**<br>*Cases:*<br>Secondary ovarian tumors<br>*Controls:*<br>Prior bilateral oophorectomy.		***By frequency of applications***<br>- <5/month vs none, OR: 1.5 (0.8 - 2.7);<br>- 5 - 29/month vs none, OR: 1.2 (0.6 - 2.2)];<br>- ≥30/month vs none, OR: 1.8 (1.1 - 3.0)<br>***By years of talc use***<br>- <10 years vs none, OR: 1.2 (0.5 - 2.6);<br>- 10-29 years vs none, OR: 1.6 (1.0 - 2.7);<br>- ≥30 years vs none, OR: 1.6 (1.0 - 2.7)];<br>***By age at first talc use***<br>- <20 years vs none, OR: 1.7 (1.1 - 2.7);		

**Study (Study design)**	**Sample size** (Cases/controls or cases/total cohort)	**Eligibility criteria**	**Confounding adjustment**	**Adjusted effect estimate(s) (95% CI)**	**Dose response**	**Overall author conclusion**
				- 20-25 years vs none, OR: 1.2 (0.6 - 2.2);<br>- ≥25 years vs none, OR: 1.6 (0.8 - 3.2)<br><br>***Years since last talc use***<br>- Within last 6 months vs none, OR: 2.3 (1.3 - 4.0);<br>- 6 months - 10 years vs none, OR: 1.1 (0.7 - 1.9);<br>- ≥10 years vs none, OR: 1.4 (0.8 - 2.6)<br><br>***Time of use***<br>*(Restricted to women older than 10 years in 1960)*<br>- Exclusive use after 1960 vs none, OR: 1.1 (0.6 - 2.1);		

Study (Study design)	**Sample size** (Cases/controls or cases/total cohort)	Eligibility criteria	Confounding adjustment	Adjusted effect estimate(s) (95% CI)	Dose response	Overall author conclusion
				- Any use before 1960 vs none, OR: 1.7 (1.1 - 2.7);<br><br>***By type of powder***<br>- Brand or generic baby powder vs none, OR: 1.6 (1.1 - 2.5);<br>- Deodorizing or other scented powders vs none OR: 1.2 (0.6 - 2.5)<br><br>***By lifetime applications of perineal talc use***<br>(10,000 applications are equivalent to daily use for 30 years)<br>- < 1000 applications vs none, OR: 1.3 (0.7 - 2.7);		

Study (Study design)	**Sample size** (Cases/controls or cases/total cohort)	Eligibility criteria	Confounding adjustment	Adjusted effect estimate(s) (95% CI)	Dose response	Overall author conclusion
				- 1000 -10,000 applications vs none, OR: 1.5 (0.9 - 2.4)];<br>- ≥10,000 applications vs none, OR: 1.8 (1.0 - 3.0)<br><br>***By total applications excluding use after hysterectomy or tubal ligation***<br>- < 1000 applications vs none, OR: 1.4 (0.7 - 2.9);<br>- 1000 -10,000 applications vs none, OR: 1.5 (0.9 - 2.4);<br>- ≥10,000 applications vs none, OR: 1.7 (1.0 - 3.0);<br>- *P value for trend 0.077*		

Study (Study design)	Sample size (Cases/controls or cases/total cohort)	Eligibility criteria	Confounding adjustment	Adjusted effect estimate(s) (95% CI)	Dose response	Overall author conclusion
				Applications excluding use after hysterectomy, tubal ligation and while on OC, pregnant or breast-feeding, or occurring after menopause<br>- <1000 applications vs none, OR: 1.5 (0.8 - 2.9);<br>- 1000 -10,000 applications vs none, OR: 1.3 (0.8 - 2.0);<br>- ≥10,000 applications vs none OR: 2.8 (1.4 - 5.4);<br>- *P value for trend 0.015*<br><br>***By indicators of ovulation and tubal occlusion***		

Study (Study design)	**Sample size** (Cases/controls or cases/total cohort)	Eligibility criteria	Confounding adjustment	Adjusted effect estimate(s) (95% CI)	Dose response	Overall author conclusion
				- All subjects, use vs nonuse, OR: 1.5 (1.0 - 2.1);<br>- Subjects with no midcycle pain, use vs nonuse, OR: 1.4 (0.9 - 2.2);<br>- Subjects with midcycle pain, use vs nonuse, OR: 2.0 (0.8 - 5.2);<br>- Subjects with irregular periods, use vs nonuse, OR: 1.1 (0.4 - 3.4);<br>- Subjects with regular periods, use vs nonuse, OR: 1.7 (1.1 - 2.5);<br>- Subjects with no PID or ectopic pregnancy, use vs nonuse, OR: 1.6 (1.1 - 2.4);<br>- Subjects with PID or ectopic pregnancy, use vs nonuse, OR: 0.1 (0.01 - 7.0)		

Study (Study design)	Sample size (Cases/controls or cases/total cohort)	Eligibility criteria	Confounding adjustment	Adjusted effect estimate(s) (95% CI)	Dose response	Overall author conclusion
				**By histologic type**<br>- Serous, use vs nonuse, OR: 1.4, (0.9 - 2.2);<br>- Mucinous, use vs nonuse, OR: 1.2 (0.6 - 2.5);<br>- Endometrioid, use vs nonuse, OR: 2.8 (1.2 - 6.4);<br>- Other, use vs nonuse, OR: 1.6 (0.8 - 3.3)<br><br>***By tumour grade***<br>- Borderline, use vs nonuse, OR: 2.4 (1.2 - 4.5);<br>- Grade 1, use vs nonuse, OR: 1.0 (0.3 - 2.8);<br>- Grade 2, use vs nonuse, OR: 1.5 (0.7 - 3.0);		

Study (Study design)	Sample size (Cases/controls or cases/total cohort)	Eligibility criteria	Confounding adjustment	Adjusted effect estimate(s) (95% CI)	Dose response	Overall author conclusion
				- Grade 3, use vs nonuse, OR: 1.5 (0.8 - 2.8);<br>- Undifferentiated, use vs nonuse, OR: 1.2 (0.7 - 2.2)		
Hartge et al, (1983), (Case-control) [3]	135/171	**Inclusion**<br>*Cases:*<br>- Confirmed primary EOC treated in participating hospitals.<br><br>*Controls*<br>- Treated at the same hospitals for conditions other than gynecologic, psychiatric, or malignant diseases or pregnancy;<br>- Frequency matched to the cases on age, race, and hospital.	Reported findings were not adjusted.	Any talc use vs nonuse, unadjusted RR 0.7 (0.4 - 1.1);<br><br>***By method of use***<br><br>- Talc use on diaphragm vs nonuse, unadjusted RR: 0.8 (0.4 - 1.4);<br>- Some body talc use vs nonuse unadjusted RR: 0.8 (0.5 - 1.2);<br>- All over use vs nonuse, unadjusted RR: 0.7 (0.4 - 1.2);<br>- Use on genitals, sanitary napkins, or underwear vs nonuse,	No trend analysis conducted	No association

Study (Study design)	Sample size (Cases/controls or cases/total cohort)	Eligibility criteria	Confounding adjustment	Adjusted effect estimate(s) (95% CI)	Dose response	Overall author conclusion
				unadjusted RR: 2.5 (0.7 - 10.0);<br>- Non-genital use vs nonuse, unadjusted RR: 0.8 (0.3 - 2.5);<br>- Use (unknown locations) vs nonuse, unadjusted RR: 0.3 (0.1 - 1.2);<br>- *RRs were unaffected by adjustment for race, age and gravidity*		
Houghton et al, (2014), (cohort) [55]	429/61,285	**Inclusion**<br>- Postmenopausal, between 50 to 79 at enrolment;<br>- Planned to reside in the area for at least three years<br>**Exclusion**<br>- Prior bilateral oophorectomy or	Adjusted for: age, race, oral contraceptive duration, hormone replacement therapy duration, family history of breast/ovari	***By use/nonuse***<br>- Perineal use, ever vs never, HR: 1.12 (0.92,1.36);<br>- Perineal use, <9 years vs never, HR: 1.23 (0.98,1.54);<br>- Perineal use, 10+ years vs never, HR: 0.98 (0.75,1.29)	No trend found	No association

Study (Study design)	Sample size (Cases/controls or cases/total cohort)	Eligibility criteria	Confounding adjustment	Adjusted effect estimate(s) (95% CI)	Dose response	Overall author conclusion
		reported an unknown number of ovaries at baseline;<br>- History of any cancer at baseline (except nonmelanoma skin cancer);<br>- Participating in another clinical trial;<br>- Unlikely to survive three years due to medical conditions;<br>- Has medical conditions that would interfere with study participation;<br>- Missing exposure or follow up information	an cancer, age at last birth, BMI, smoking, tubal ligation and parity.	***By method/frequency of use***<br>- Sanitary napkin use, ever vs never, HR: 0.95 (0.76,1.20);<br>- Sanitary napkin use, <9 years vs never, HR: 0.96 (0.73,1.26);<br>- Sanitary napkin use, 10+ years vs never, HR: 0.95 (0.65,1.37);<br>- Diaphragm use, ever vs never, HR: 0.92 (0.68,1.23);<br>- Diaphragm use, <9 years vs never, HR: 0.91 (0.64,1.30);<br>- Diaphragm use, 10+ years vs never, HR: 0.95 (0.58,1.56);<br>- Combined use, ever vs never, HR: 1.06 (0.87,1.28);		

Study (Study design)	**Sample size** (Cases/controls or cases/total cohort)	Eligibility criteria	Confounding adjustment	Adjusted effect estimate(s) (95% CI)	Dose response	Overall author conclusion
				- Combined use, <9 years vs never, HR: 1.09 (0.88,1.36);<br>- Combined use, 10+ years vs never, HR: 1.02 (0.80,1.30)<br><br>***By Application Site***<br>- Only genital powder, ever vs never, HR: 1.13 (0.88,1.45);<br>- Only diaphragm powder, ever vs never, HR: 0.80 (0.50,1.29);<br>- Only sanitary napkin powder, ever vs never, HR: 1.01 (0.68,1.50);<br>- Genital and sanitary napkin powder, ever vs never, HR: 1.08 (0.80,1.46);		

Study (Study design)	**Sample size** (Cases/controls or cases/total cohort)	Eligibility criteria	Confounding adjustment	Adjusted effect estimate(s) (95% CI)	Dose response	Overall author conclusion
				- Genital and diaphragm powder, ever vs never, HR: 1.45 (0.95,2.23);<br>- Diaphragm and sanitary napkin powder, ever vs never, HR: 1.02 (0.38,2.74);<br>- Genital, diaphragm, and sanitary napkin powder, ever vs never, HR: 0.50 (0.21,1.22)<br><br>***By tumor histology***<br>- Serous, ever vs never, HR: 1.16 (0.88,1.53);<br>- Serous invasive, ever vs never, HR: 1.13 (0.84,1.51);<br>- Mucinous, ever vs never, HR: 1.03 (0.47,2.27);<br>- Endometrioid, ever vs never, HR: 1.29 (0.64,2.61);		

**Study (Study design)**	**Sample size** (Cases/controls or cases/total cohort)	**Eligibility criteria**	**Confounding adjustment**	**Adjusted effect estimate(s) (95% CI)**	**Dose response**	**Overall author conclusion**
				- Other, ever vs never, HR: 1.04 (0.70,1.54);		
Kurta et al, (2012), (Case-control) **[**49**]**	902/1,802	**Inclusion**<br>*Cases*<br>- Diagnosed with primary epithelial ovarian, peritoneal, or fallopian tube cancers diagnosed between 2003 and 2008 within nine months of recruitment;<br>- >25 years<br><br>*Controls*<br>Matched on age (+/- 5 years) and residence<br><br>**Exclusion**<br>Bilateral oophorectomy or prior ovarian cancer diagnosis	Adjusted for: Age, race, education Matched for: age (+/-5 years) and telephone area code	- Ever vs never use, OR: 1.40 (1.16–1.69)	No trend analysis conducted	Positive association

**Study (Study design)**	**Sample size** (Cases/controls or cases/total cohort)	**Eligibility criteria**	**Confounding adjustment**	**Adjusted effect estimate(s) (95% CI)**	**Dose response**	**Overall author conclusion**
Langseth and Kjærheim, (2004), (Case-control) [42]	46/179	**Inclusion**<br>All subjects selected from the pulp and paper cohort from 10 different mills.<br><br>*Cases*<br>- From pulp and paper cohort during a follow-up period from 1953-1999;<br>- Have epithelial ovarian cancer;<br>- Registered with the Cancer Registry of Norway;<br>- Histological records reviewed by oncologist<br><br>*Controls*	Reported findings were not adjusted.<br><br>*Authors reported that adjusting for number of children, breastfeeding, age at birth of first and last child, age at menarche and menopause, smoking habits, and family history of ovarian or breast*	- Perineal exposure, ever vs never, unadjusted OR: 1.15 (0.41–3.21)	No trend analysis conducted	No association

**Study (Study design)**	**Sample size** (Cases/controls or cases/total cohort)	**Eligibility criteria**	**Confounding adjustment**	**Adjusted effect estimate(s) (95% CI)**	**Dose response**	**Overall author conclusion**
		- 4 controls for each case from cohort (by incidence density sampling);<br>- Matched by age (+/- 2 yrs);<br>- Free of ovarian cancer, intact ovaries<br><br>**Exclusion**<br>*Cases*<br>Germ cell tumor<br><br>*Controls*<br>- Died or emigrated;<br>- Cannot be interviewed due to illness	*cancer did not result in substantially different results.*			
Merritt et al, (2008),	1,576/1,509	**Inclusion**<br>*Cases*	Matched by: age (+/-5 years) and	Talc use on underarms/abdomen/chest vs non-use, OR 1.01 (0.84,	No trend found	Positive association strongest

**Study (Study design)**	**Sample size** (Cases/controls or cases/total cohort)	**Eligibility criteria**	**Confounding adjustment**	**Adjusted effect estimate(s) (95% CI)**	**Dose response**	**Overall author conclusion**
(Case-control) **[45]**		- Selected from the Australian Ovarian Cancer Study;<br>- Incident case of invasive and low malignant potential (LMP) cancer of ovaries, peritoneum or fallopian tube;<br>- Diagnosed in women (aged 18–79 years) between January 2002 and June 2005;<br>- Histopathologically confirmed LMP<br><br>*Controls*<br>- Randomly identified from Australian Electoral Rolls;<br>- Matched to age (+/- 5 years) and state of residence	residence.<br><br>Adjusted for: age, education, parity and OC use.	1.30)<br><br>***By histology***<br>- Ever vs never use, all cases, OR: 1.17 (1.01,1.36);<br>- Ever vs never use, serous, OR: 1.21 (1.03,1.44);<br>- Ever vs never use, mucinous, OR: 1.10 (0.80,1.52);<br>- Ever vs never use, endometrioid, OR: 1.18 (0.81,1.70);<br>- Ever vs never use, clear cell, OR: 1.08 (0.68,1.72)<br><br>***By duration and histology among women with no surgery, or use pre-surgery***		for serous and endometrioid subtypes.

Study (Study design)	Sample size (Cases/controls or cases/total cohort)	Eligibility criteria	Confounding adjustment	Adjusted effect estimate(s) (95% CI)	Dose response	Overall author conclusion
		**Exclusion**<br>*Cases*<br>- No physician consent to contact cases (were too sick);<br>- Could not be contacted (reason not given);<br>- Had language difficulties, mental incapacity and illness;<br>- Non-epithelial, non-ovarian or benign tumours;<br>- Cancer first diagnosed before the start of the study period.<br>*Controls*<br>- Language difficulties;<br>- Illness;<br>- Declined;		- 0–10 years vs never use, all cases, OR: 1.13 (0.90,1.41);<br>- >10–25 years vs never use, all cases, OR: 1.08 (0.87,1.34);<br>- >25 years vs never use, all cases, OR: 1.29 (1.04,1.58);<br>- *P value for trend 0.021*<br><br>- 0–10 years vs never use, serous, OR: 1.26 (0.98,1.63);<br>- >10–25 years vs never use, serous, OR: 1.03 (0.80,1.32);<br>- >25 years vs never use, serous, OR: 1.34 (1.06,1.68);<br>- *P value for trend 0.022*<br><br>- 0–10 years vs never use, mucinous, OR: 0.79 (0.47,1.33);		

Study (Study design)	Sample size (Cases/controls or cases/total cohort)	Eligibility criteria	Confounding adjustment	Adjusted effect estimate(s) (95% CI)	Dose response	Overall author conclusion
		- Had previous ovarian cancer/bilateral oophorectomy		- >10–25 years vs never use, mucinous, OR: 1.34 (0.86,2.08);<br>- >25 years vs never use, mucinous, aOR: 1.21 (0.75,1.97);<br>- *P value for trend 0.27*<br><br>- 0–10 years vs never use, endometrioid , OR: 1.05 (0.59,1.85);<br>>10–25 years vs never use, endometrioid , OR: 1.14 (0.67,1.94);<br>- >25 years vs never use, endometrioid , OR: 1.31 (0.80,2.16);<br>- *P value for trend 0.28*<br><br>- 0–10 years vs never use, clear cell, OR: 1.08 (0.52,2.27);<br>- >10–25 years vs never use, clear cell, OR: 0.96 (0.48,1.90);		

Study (Study design)	Sample size (Cases/controls or cases/total cohort)	Eligibility criteria	Confounding adjustment	Adjusted effect estimate(s) (95% CI)	Dose response	Overall author conclusion
				- >25 years vs never use, clear cell, OR: 1.18 (0.63,2.22);<br>- *P value for trend 0.69*<br><br>***By duration and histology among women post-surgery (genital tract obstructed):***<br>- 0–10 years vs never use, all cases, OR: 1.08 (0.71,1.62);<br>- >10–25 years vs never use, all cases, OR: 1.14 (0.82,1.57);<br>- >25 years vs never use, all cases, OR: 1.00 (0.64,1.51);<br>- *P value for trend 0.61*<br><br>- 0–10 years vs never use, serous, OR: 1.07 (0.67,1.69);		

Study (Study design)	Sample size (Cases/controls or cases/total cohort)	Eligibility criteria	Confounding adjustment	Adjusted effect estimate(s) (95% CI)	Dose response	Overall author conclusion
				- >10–25 years vs never use, serous, OR: 1.03 (0.72,1.48);<br>- >25 years vs never use, serous, OR: 1.09 (0.69,1.71);<br>- *P value for trend 0.60*<br><br>- 0–10 years vs never use, mucinous, OR: 1.39 (0.60,3.19);<br>- >10–25 years vs never use, mucinous, OR: 2.04 (1.09,3.79);<br>- >25 years vs never use, mucinous, OR: 0.91 (0.27,3.05);<br>- *P value for trend 0.12*<br><br>- 0–10 years vs never use, endometrioid, OR: 0.97 (0.34,2.77);<br>- >10–25 years vs never use, endometrioid, OR: 1.03 (0.45,2.32);		

Study (Study design)	Sample size (Cases/controls or cases/total cohort)	Eligibility criteria	Confounding adjustment	Adjusted effect estimate(s) (95% CI)	Dose response	Overall author conclusion
				- >25 years vs never use, endometrioid, OR: 0.79 (0.23,2.64);<br>- *P value for trend 0.81*<br><br>- 0–10 years vs never use, clear cell, OR: 0.64 (0.15,2.81);<br>- >10–25 years vs never use, clear cell, OR: 0.44 (0.11,1.88);<br>- >25 years vs never use, clear cell, OR: 0.43 (0.06,3.22);<br>- *P value for trend 0.16*<br><br>***By age at diagnosis/recruitment and histology among women with unobstructed genital tract***		

Study (Study design)	**Sample size** (Cases/controls or cases/total cohort)	Eligibility criteria	Confounding adjustment	Adjusted effect estimate(s) (95% CI)	Dose response	Overall author conclusion
				- <50 years vs never use, all cases, OR: 1.16 (0.86,1.57);<br>- <50 years vs never use, serous, OR: 1.53 (1.06,2.19);<br>- <50 years vs never use, mucinous, OR: 1.42 (0.89,2.25);<br>- <50 years vs never use, endometrioid, OR: 0.66 (0.28,1.55);<br>- <50 years vs never use, clear cell, OR: 0.98 (0.41,2.29)<br><br>- 50–59 years vs never use, all cases, OR: 1.22 (0.93,1.59);<br>- 50–59 years vs never use, serous, OR: 1.20 (0.89,1.62);<br>- 50–59 years vs never use, mucinous, OR: 0.76 (0.46,1.26);		

Study (Study design)	**Sample size** (Cases/controls or cases/total cohort)	Eligibility criteria	Confounding adjustment	Adjusted effect estimate(s) (95% CI)	Dose response	Overall author conclusion
				- 50–59 years vs never use, endometrioid, OR: 1.41 (0.78,2.54);<br>- 50–59 years vs never use, clear cell, OR 1.67 (0.88,3.15)<br><br>- 60–69 years vs never use, all cases, OR: 0.93 (0.70,1.23);<br>- 60–69 years vs never use, serous, OR: 0.95 (0.70,1.29);<br>- 60–69 years vs never use, mucinous, OR: 0.83 (0.49,1.40);<br>- 60–69 years vs never use, endometrioid, OR: 1.31 (0.62,2.75);<br>- 60–69 years vs never use, clear cell, OR: 0.87 (0.40,1.85)		

Study (Study design)	Sample size (Cases/controls or cases/total cohort)	Eligibility criteria	Confounding adjustment	Adjusted effect estimate(s) (95% CI)	Dose response	Overall author conclusion
				- ≥70 years vs never use, all cases, aOR: 1.61 (1.10,2.36);<br>- ≥70 years vs never use, serous, OR: 1.66 (1.08,2.56);<br>- ≥70 years vs never use, mucinous, OR: 0.91 (0.42,1.97);<br>- ≥70 years vs never use, endometrioid, OR: 1.32 (0.50,3.49);<br>- ≥70 years vs never use, clear cell, OR: 1.41 (0.58,3.35)		
Mills et al, (2004), (Case-control) [19]	249/1,105	**Inclusion**<br>*Cases:*<br>- In one of the two population-based cancer registries in Central Valley of California (Cancer Registry of Central California (CRCC) and	Adjusted for: age, race, duration of OC use and breast feeding.	- Ever vs never, OR: 1.37 (1.02,1.85)<br>***By frequency of use***<br>- Rarely to several times per month vs never, OR: 0.34 (0.87–2.08);	No trend found	Positive association for invasive and serous invasive tumours.

Study (Study design)	Sample size (Cases/controls or cases/total cohort)	Eligibility criteria	Confounding adjustment	Adjusted effect estimate(s) (95% CI)	Dose response	Overall author conclusion
		Cancer Surveillance Program (CSP);<br>- All newly diagnosed histologically confirmed EOC patients in 2000 and 2001;<br>- Living in Central Valley from Jan 2000-Dec 2001;<br>- Physician consent for interview with patient<br><br>*Controls*<br>- 18+ years identified by RDD;<br>- Residents of the area;<br>- No diagnosis of EOC and at least one intact ovary at time of interview;<br>- Matched on race and race	- 1–3 times per week vs never, OR: 1.16 (0.74–1.81);<br>- 4–7 times per week vs never, OR: 1.74 (1.14–2.64);<br>- *P-value for trend 0.015*<br><br>***By duration of use***<br>- ≤ 3 years vs never, OR: 1.01 (0.58–1.76);<br>- 4–12 years vs never, OR: 1.86 (1.16–2.98);<br>- 13–30 years vs never, OR: 1.45 (0.90–2.32;<br>- > 30 years vs never, OR: 1.22 (0.72–2.08);<br>- *P value for trend 0.045*<br><br>***By cumulative use (frequency × duration):***			

Study (Study design)	Sample size (Cases/controls or cases/total cohort)	Eligibility criteria	Confounding adjustment	Adjusted effect estimate(s) (95% CI)	Dose response	Overall author conclusion
		**Exclusion**<br>*Cases*<br>- Subjects that could not communicate in English or Spanish;<br>- Subjects with speech/ hearing impairment;<br>- Death before being contacted;<br>- Refusal to participate by physician;<br>- Too ill to participate<br><br>*Controls*<br>- Subjects that could not communicate in English or Spanish;<br>- Prior bilateral oophorectomy;<br>- Refuse to participate		- First quartile (lowest exposure) vs never; OR: 1.03 (0.59–1.80);<br>- Second quartile vs never, OR: 1.81 (1.10–2.97);<br>- Third quartile vs never, OR: 1.74 (1.11–2.73);<br>- Fourth quartile (highest exposure) vs never, OR: 1.06 (0.62–1.83);<br>- *P value for trend 0.051*<br><br>***By invasiveness and histologic subtype***<br>- All invasive, use vs non-use, OR: 1.51 (1.07–2.12);<br>- Serous invasive, use vs non-use, OR: 1.77 (1.12–2.81);		

Study (Study design)	Sample size (Cases/controls or cases/total cohort)	Eligibility criteria	Confounding adjustment	Adjusted effect estimate(s) (95% CI)	Dose response	Overall author conclusion
				- Mucinous invasive, use vs non-use, OR: 2.56 (0.89–7.39);<br>- Endometrioid, use vs non-use, OR: 1.28 (0.62–2.62);<br>- Clear cell, use vs non-use, OR: 0.63 (0.15–2.64);<br>- Other epithelial, use vs non-use, OR: 1.06 (0.45–2.48);<br>- All borderline, use vs non-use, OR: 1.09 (0.65–1.83);<br>- Serous borderline, use vs non-use, OR: 1.28 (0.71–2.31);<br>- Mucinous borderline, use vs non-use, OR: 0.76 (0.28–2.07)<br>***By Timing of Talc Use***<br>- Year of first use before/during 1975 vs		

Study (Study design)	Sample size (Cases/controls or cases/total cohort)	Eligibility criteria	Confounding adjustment	Adjusted effect estimate(s) (95% CI)	Dose response	Overall author conclusion
				never use, OR: 1.22 (0.84–1.77);<br>- Year of first use after 1975 vs never use, OR: 1.92 (1.27–2.91);<br>- Age at first use <20 years vs never use, OR: 0.95 (0.61–1.48);<br>- Age at first use 20–24 years vs never use, OR: 2.41 (1.43–4.09);<br>- Age at first use ≥ 25 years vs never use, OR: 1.80 (1.19–2.73)<br><br>***By Relation to First Birth***<br>- First use at or prior to first birth vs never use, OR: 0.98 (0.64–1.48);<br>- First use after first birth vs never use, OR: 2.51 (1.63–3.87)		

Study (Study design)	**Sample size** (Cases/controls or cases/total cohort)	Eligibility criteria	Confounding adjustment	Adjusted effect estimate(s) (95% CI)	Dose response	Overall author conclusion
				By Duration Since Last Use<br>- Current users vs never use, OR: 1.27 (0.81–1.98);<br>- 1–2 years since last use vs never use, OR: 2.40 (1.43–4.05);<br>- 3–20 years since last use vs never use, OR: 1.57 (0.90–2.73);<br>- > 20 years since last use vs never use, OR: 1.13 (0.66–1.94)<br><br>***By Levels of Modifiers***<br>*Pelvic Surgery*<br>- Tubal ligation, ever vs never use, OR: 0.88 (0.46–1.68);<br>- No tubal ligation, ever vs never use, OR: 1.54 (1.10–2.16);		

Study (Study design)	Sample size (Cases/controls or cases/total cohort)	Eligibility criteria	Confounding adjustment	Adjusted effect estimate(s) (95% CI)	Dose response	Overall author conclusion
				- Hysterectomy, ever vs never use, OR: 1.79 (0.91–3.52);<br>- No hysterectomy, ever vs never use, OR: 1.33 (0.95–1.87)<br><br>*Pregnancy*<br>- Ever pregnant, ever vs never use, OR: 1.44 (1.05–1.97);<br>- Never pregnant, ever vs never use, OR: 0.93 (0.37–2.34);<br>- Ever parous, ever vs never use, OR: 1.34 (0.97–1.85);<br>- Nulliparous, ever vs never use, OR: 4.91 (0.68–35.25)<br><br>*Oral Contraceptive Use*		

**Study (Study design)**	**Sample size** (Cases/controls or cases/total cohort)	**Eligibility criteria**	**Confounding adjustment**	**Adjusted effect estimate(s) (95% CI)**	**Dose response**	**Overall author conclusion**
				- Ever OC use, ever vs never use, OR: 1.26 (0.86–1.83);<br>- Never OC use, ever vs never use, OR: 1.63 (1.0–2.64);<br>- HRT, ever vs never use, OR: 1.41 (0.89–2.24);<br>- No HRT, ever vs never use, OR: 1.30 (0.87–1.93)<br><br>*BMI*<br>- BMI < 25, ever vs never use, OR: 1.23 (0.74–2.04);<br>- BMI ≥ 25, ever vs never use, OR: 1.36 (0.92–1.99)		
Moorman et al, (2009), (Case-	African-American: 143/189;	**Inclusion**<br>*Pool:*<br>- Newly diagnosed cases of EOC;	Adjusted for: age.	- Whites, use vs nonuse, OR: 1.04 (0.82 - 1.33);	No trend analysis conducted	No association

Study (Study design)	Sample size (Cases/controls or cases/total cohort)	Eligibility criteria	Confounding adjustment	Adjusted effect estimate(s) (95% CI)	Dose response	Overall author conclusion
control) [46]	White 943/868	- Identified through the North Carolina Central Cancer Registry<br><br>*Cases*<br>- 20–74 years at diagnosis;<br>- No prior history of ovarian cancer;<br>- Resided in the study area;<br>- Cognitively able to give consent;<br>- Could complete an interview in English<br><br>*Controls:*<br>- Frequency-matched by age and race/ethnicity to the cases;<br>- Recruited from the same geographic region		- African-Americans, use vs nonuse, OR: 1.19 (0.68 - 2.09)		

Study (Study design)	Sample size (Cases/controls or cases/total cohort)	Eligibility criteria	Confounding adjustment	Adjusted effect estimate(s) (95% CI)	Dose response	Overall author conclusion
		using list-assisted RDD dialing;<br>- Same eligibility criteria as cases<br><br>**Exclusion**<br>- Prior history of bilateral oophorectomy				
Ness et al, (2000), (Case-control) [18]	767/1,367	**Inclusion**<br>*Cases*<br>- Age 20-69 years;<br>- Diagnosed with EOC within the 6 months before the interview;<br>- Identified from 39 hospitals in Eastern Pennsylvania, Southern New Jersey, and Delaware<br><br>*Controls:*	Adjusted for: age, number of pregnancies, family history of ovarian cancer, race, OC use, tubal ligation, hysterectomy, and breast-feeding.	- On genital/rectal area, ever vs never, OR: 1.5 (1.1 - 2.0)<br><br>***By method of application***<br>- On feet, ever vs never, OR: 1.4 (1.1 - 1.6);<br>- On sanitary napkins, ever vs never, OR: 1.6 (1.1 - 2.3);<br>- On underwear, ever vs never, OR: 1.7 (1.2 - 2.4);	No trend found	Positive association for any method of use.

Study (Study design)	Sample size (Cases/controls or cases/total cohort)	Eligibility criteria	Confounding adjustment	Adjusted effect estimate(s) (95% CI)	Dose response	Overall author conclusion
		- Women <66;<br>- Identified by RDD and Health Care Financing Administration (HCFA) lists;<br>- Frequency matched to cases by 5-year age groups and three-digit telephone exchange<br><br>**Exclusion**<br>*Cases:*<br>- Prior ovarian cancer, diagnosed more than 6 months before interview;<br>- Did not speak English, mentally incompetent, critically ill or dead<br><br>*Controls*		- On diaphragm/cervical cap, ever vs never, OR: 0.6 (0.3 - 1.2);<br>- On male partner, ever vs never, OR: 1.0 (0.7 - 1.4);<br><br>***Duration of use***<br>- <1 year vs never, OR: 2.0 (1.0 - 4.0);<br>- 1 - 4 years vs never, OR: 1.6 (1.1 - 2.3);<br>- 5 - 9 years vs never, OR: 1.2 (0.8 - 1.9);<br>- 10+ years vs never, OR: 1.2 (1.0 - 1.5);		

**Study (Study design)**	**Sample size** (Cases/controls or cases/total cohort)	**Eligibility criteria**	**Confounding adjustment**	**Adjusted effect estimate(s) (95% CI)**	**Dose response**	**Overall author conclusion**
		- Residence outside of the target counties;<br>- Prior ovarian cancer, bilateral oophorectomy;<br>- Not speaking English, mentally incompetent, in critical illness, being untraceable or dead				
Rosenblatt et al. (1992) (Case-control) [9]	77/46 (analysed)	**Inclusion**<br>*Cases:*<br>- Diagnosed with EOC<br>*Controls*<br>- In-patients without gynecologic or malignant conditions;<br>- Initially matched to cases by age (within 5 years), race, and date of admission;<br>- Unmatched cases were therefore matched a posteriori to controls, to	Adjusted for: number of live births, education, and religion.	- Genital fiber exposure, use vs. non-use, OR: 1 (0.2-4.0), adjusted for number of live births;<br>***Method of use***<br>- Diaphragm use with powder, use vs nonuse, OR: 3 (0.8-10.8), adjusted for number of live births and education;<br>- Genital bath talc, use vs nonuse, OR: 1.7 (0.7- 3.9);	Positive trend for duration of use since tubal ligation	Possible association

Study (Study design)	**Sample size** (Cases/controls or cases/total cohort)	Eligibility criteria	Confounding adjustment	Adjusted effect estimate(s) (95% CI)	Dose response	Overall author conclusion
		form matched triplets of 2 cases and 1 control<br><br>**Exclusion**<br>- Gynecologic or malignant conditions		- Sanitary napkin with talc, use vs nonuse, OR: 4.8 (1.3-17.8), adjusted for weight<br><br>***Pelvic Operations***<br>- Women with tubal ligation, years of use after subtraction of the time since tubal ligation, ≥ 37.4 years vs. <37.4 years, OR: 2.4 (1.0-5.8), adjusted for religion;<br><br>- Women with ovarian biopisies, talc use vs nonuse, OR: 1.1 (0.3-4.4);<br>- Women with unilateral oophorectomy, talc use vs nonuse, OR: 0.8 (0.2-2.5);		

**Study (Study design)**	**Sample size** (Cases/controls or cases/total cohort)	**Eligibility criteria**	**Confounding adjustment**	**Adjusted effect estimate(s) (95% CI)**	**Dose response**	**Overall author conclusion**
				- Women with tubal ligation, talc use vs nonuse, OR: 0.2 (0.03 - 0.9), adjusted for education on subject and for highest weight 20 years prior to diagnosis;<br><br>- Women with hysterectomy, talc use vs nonuse, OR: 0.7 (0.3 - 1.74), adjusted for education;<br><br>- Condom use, talc use vs nonuse, OR: 1.6 (0.6-3.9), adjusted for number of live births and education.		
Rosenblatt et al. (2011), (Case-	812/1,313	**Inclusion**<br>*Cases*	Adjusted for age, calendar year of diagnosis/re	Use' defined as regular use for at least one year.	No trend found	Possible association

Study (Study design)	Sample size (Cases/controls or cases/total cohort)	Eligibility criteria	Confounding adjustment	Adjusted effect estimate(s) (95% CI)	Dose response	Overall author conclusion
control) [48]		- Resident of 13 counties in western Washington State;<br>- Between 35–74 years of age;<br>- Diagnosed with a primary invasive or borderline epithelial ovarian tumor between 1 January 2002 and 31 December 2005;<br>- Identified through a population-based cancer registry, the Cancer Surveillance System, part of the Surveillance, Epidemiology, and End Results Program (SEER);<br>- Cases were restricted to English-speaking women who had	ference date, county of residence, number of full-term births, and duration of OC use.	- Perineal use of powder after bathing vs non-use, all tumors, OR: 1.27 (0.97,1.66)<br><br>***By tumor histology***<br>- Use of perineal powder after bathing vs non-use, mucinous borderline tumors, OR: 1.78 (0.98,3.23);<br>- Use of perineal powder after bathing vs non-use, serous borderline tumors, OR: 1.47 (0.84,2.55);<br>- Use of perineal powder after bathing vs non-use, serous invasive tumor, OR: 1.01 (0.69,1.47);<br>- Use of perineal powder after bathing vs non-use, endometrioid/clear		

Study (Study design)	Sample size (Cases/controls or cases/total cohort)	Eligibility criteria	Confounding adjustment	Adjusted effect estimate(s) (95% CI)	Dose response	Overall author conclusion
		residential telephones at the time of diagnosis<br><br>*Controls*<br>- Selected by RDD using stratified sampling in 5-year age categories, 1-year calendar intervals, and two county strata;<br>- 2:1 ratio to women with invasive cancer;<br>- English speaking;<br>- With at least one ovary and no prior history of ovarian cancer		invasive tumor, OR: 1.53 (0.91,2.57);<br>- Use of perineal powder after bathing vs non-use, other nonmucinous invasive tumor, OR: 1.48 (0.85,2.58)<br><br>***By method of use and histology***<br>- Perineal use of powder after bathing vs non-use, all tumors, OR: 1.27 (0.97,1.66);<br>- Perineal use of powder after bathing vs non-use, borderline tumors, OR: 1.55 (1.02,2.37);<br>- Perineal use of powder after bathing vs non-use, invasive tumors, OR: 1.17 (0.87,1.58);		

Study (Study design)	Sample size (Cases/controls or cases/total cohort)	Eligibility criteria	Confounding adjustment	Adjusted effect estimate(s) (95% CI)	Dose response	Overall author conclusion
				- Perineal powder on sanitary napkins vs non-use, all tumors, OR: 0.82 (0.58,1.16);<br>- Perineal powder on sanitary napkins vs non-use, borderline tumors, OR: 1.03 (0.58,1.84);<br>- Perineal powder on sanitary napkins vs non-use, invasive tumors, OR: 0.75 (0.51,1.12);<br>- Perineal powder use on diaphragm vs non-use, all tumors, OR: 0.72 (0.48,1.10);<br>- Perineal powder use on diaphragm vs non-use, borderline tumors, OR: 0.60 (0.27,1.33);<br>- Perineal powder use on diaphragm vs non-use, invasive tumors, OR: 0.77 (0.49,1.21);		

Study (Study design)	Sample size (Cases/controls or cases/total cohort)	Eligibility criteria	Confounding adjustment	Adjusted effect estimate(s) (95% CI)	Dose response	Overall author conclusion
				- Perineal use of vaginal deodorant spray vs non-use, all tumors, OR: 1.15 (0.85,1.56);<br>- Perineal use of vaginal deodorant spray vs non-use, borderline tumors, OR: 1.20 (0.74,1.95);<br>- Perineal use of vaginal deodorant spray vs non-use, invasive tumors, OR: 1.14 (0.81,1.59)<br><br>***By duration of use after bathing and histology***<br>- 1–9.9 years vs never used, borderline tumors, OR: 1.33 (0.61,2.87);<br>- 1–9.9 years vs never used, invasive tumors, OR: 1.42 (0.83,2.43);		

**Study (Study design)**	**Sample size** (Cases/controls or cases/total cohort)	**Eligibility criteria**	**Confounding adjustment**	**Adjusted effect estimate(s) (95% CI)**	**Dose response**	**Overall author conclusion**
				- 1–9.9 years vs never used, all tumors, OR: 1.39 (0.85,2.28);<br>- 10–19.9 years vs never used, borderline tumors, OR: 1.97 (0.93,4.17);<br>- 10–19.9 years vs never used, invasive tumors, OR: 1.28 (0.71,2.29);<br>- 10–19.9 years vs never used, all tumors, OR: 1.46 (0.87,2.45);<br>- 20–34.9 years vs never used, borderline tumors, OR: 1.83 (0.88,3.80);<br>- 20–34.9 years vs never used, invasive tumors, OR: 1.11 (0.63,1.95);<br>- 20–34.9 years vs never used, all tumors, OR: 1.28 (0.78, 2.10);<br>- 35+ years vs never used, borderline tumors, OR: 1.08 (0.37,3.15);		

Study (Study design)	Sample size (Cases/controls or cases/total cohort)	Eligibility criteria	Confounding adjustment	Adjusted effect estimate(s) (95% CI)	Dose response	Overall author conclusion
				- 35+ years vs never used, invasive tumors, OR: 0.86 (0.46,1.60);<br>- 35+ years vs never used, all tumors, OR: 0.91 (0.51,1.62)<br><br>***By lifetime number of applications after bathing and histology:***<br>- 1–1,599 applications vs never used, borderline tumors, OR: 1.05 (0.42,2.61);<br>- 1–1,599 applications vs never used, invasive tumors, OR: 1.26 (0.71,2.25);<br>- 1–1,599 applications vs never used, all tumors, OR: 1.21 (0.71,2.06);<br>- 1,600–4,799 applications vs never used, borderline		

Study (Study design)	Sample size (Cases/controls or cases/total cohort)	Eligibility criteria	Confounding adjustment	Adjusted effect estimate(s) (95% CI)	Dose response	Overall author conclusion
				tumors,<br>OR: 3.11 (1.67,5.78);<br>- 1,600–4,799 applications vs never used, invasive tumors,<br>OR: 1.72 (1.03–2.88);<br>- 1,600–4,799 applications vs never used, all tumors,<br>OR: 2.08 (1.32–3.27);<br>- 4,800-9,999 applications vs never used, borderline tumors,<br>OR: 1.19 (0.49,2.92);<br>- 4,800-9,999 applications vs never used, invasive tumors,<br>OR: 0.78 (0.41,1.48);<br>- 4,800-9,999 applications vs never used, all tumors,<br>OR: 0.87 (0.50,1.53);<br>- 10,000 applications vs never used, borderline		

Study (Study design)	**Sample size** (Cases/controls or cases/total cohort)	Eligibility criteria	Confounding adjustment	Adjusted effect estimate(s) (95% CI)	Dose response	Overall author conclusion
				tumors, OR: 0.98 (0.34,2.85);<br>- 10,000 applications vs never used, invasive tumors, OR: 0.84 (0.44,1.59);<br>- 10,000 applications vs never used, all tumors, OR: 0.87 (0.48,1.57)<br><br>***By age at first use of regular use after bathing and histology:***<br>- <15 years of age vs never used, borderline tumors, OR: 0.89 (0.30,2.66);<br>- <15 years of age vs never used, invasive tumors, OR: 0.67 (0.30,1.53);		

Study (Study design)	Sample size (Cases/controls or cases/total cohort)	Eligibility criteria	Confounding adjustment	Adjusted effect estimate(s) (95% CI)	Dose response	Overall author conclusion
				- <15 years of age vs never used, all tumors, OR: 0.74 (0.37,1.50);<br>- 15 – <20 years of age vs never used, borderline tumors, OR: 1.46 (0.64,3.31);<br>- 15 – <20 years of age vs never used, invasive tumors, OR: 1.10 (0.61,1.97);<br>- 15 – <20 years of age vs never used, all tumors, OR 1.20 (0.71,2.03);<br>- 20 – <30 years of age vs never used, borderline tumors, OR: 1.93 (0.98,3.80);<br>- 20 – <30 years of age vs never used, invasive tumors, OR: 1.04 (0.59,1.81);<br>- 20 – <30 years of age vs never used, all		

Study (Study design)	**Sample size** (Cases/controls or cases/total cohort)	Eligibility criteria	Confounding adjustment	Adjusted effect estimate(s) (95% CI)	Dose response	Overall author conclusion
				tumors, OR: 1.25 (0.77,2.03);<br>- 30+ years of age vs never used, borderline tumors, OR 1.68 (0.79,3.60);<br>- 30+ years of age vs never used, invasive tumors, OR: 1.68 (1.04,2.72);<br>- 30+ years of age vs never used, all tumors, OR: 1.69 (1.08,2.64)<br><br>***By age at last use after bathing and histology***<br>- <35 years of age vs never used, borderline tumors, OR: 1.54 (0.72,3.28);<br>- <35 years of age vs never used, invasive		

Study (Study design)	Sample size (Cases/controls or cases/total cohort)	Eligibility criteria	Confounding adjustment	Adjusted effect estimate(s) (95% CI)	Dose response	Overall author conclusion
				tumors, OR: 0.97 (0.51,1.83);<br>- <35 years of age vs never used, all tumors, OR: 1.14 (0.66,1.97);<br>- 35 – <50 years of age vs never used, borderline tumors, OR: 2.07 (1.09,3.93);<br>- 35 – <50 years of age vs never used, invasive tumors, OR: 1.15 (0.65,2.03);<br>- 35 – <50 years of age vs never used, all tumors, OR: 1.42 (0.88,2.31);<br>- 50 – <60 years of age vs never used, borderline tumors, OR: 1.39 (0.56,3.44);<br>- 50 – <60 years of age vs never used, invasive tumors, OR: 1.20 (0.67,2.15);		

**Study (Study design)**	**Sample size** (Cases/controls or cases/total cohort)	**Eligibility criteria**	**Confounding adjustment**	**Adjusted effect estimate(s) (95% CI)**	**Dose response**	**Overall author conclusion**
				- 50 – <60 years of age vs never used, all tumors, OR: 1.25 (0.73,2.13);<br>- 60+ years of age vs never used, borderline tumors, OR: 0.64 (0.15,2.74);<br>- 60+ years of age vs never used, invasive tumors, OR: 1.30 (0.76,2.25);<br>- 60+ years of age vs never used, all tumors, OR: 1.21 (0.72,2.05)<br><br>***By calendar year of first use after bathing and histology***		

Study (Study design)	Sample size (Cases/controls or cases/total cohort)	Eligibility criteria	Confounding adjustment	Adjusted effect estimate(s) (95% CI)	Dose response	Overall author conclusion
				- ≤1959 vs never use, borderline tumors, OR: 1.47 (0.55,3.92);<br>- ≤1959 vs never use, invasive tumors, OR: 0.73 (0.38,1.40);<br>- ≤1959 vs never use, all tumors, OR: 0.86 (0.48,1.53);<br>- 1960-1969 vs never use, borderline tumors, OR: 0.82 (0.28,2.38);<br>- 1960-1969 vs never use, invasive tumors, OR: 1.18 (0.66,2.09);<br>- 1960-1969 vs never use, all tumors, OR: 1.10 (0.65,1.89);<br>- 1970-1979 vs never use, borderline tumors, OR: 1.65 (0.81,3.37);<br>- 1970-1979 vs never use, invasive tumors, OR: 0.91 (0.49,1.69);		

Study (Study design)	**Sample size** (Cases/controls or cases/total cohort)	Eligibility criteria	Confounding adjustment	Adjusted effect estimate(s) (95% CI)	Dose response	Overall author conclusion
				- 1970-1979 vs never use, all tumors, OR: 1.12 (0.66,1.89);<br>- 1980+ vs never use, borderline tumors, OR: 2.20 (1.11,4.34);<br>- 1980+ vs never use, invasive tumors, OR: 1.97 (1.18,3.28);<br>- 1980+ vs never use, all tumors, OR: 2.03 (1.28,3.24)<br><br>***By time since first use (years) of perineal powder after bathing and histology***<br>- ≤25 years vs never used, borderline tumors, OR: 1.78 (0.89,3.54);<br>- ≤25 years vs never used, invasive tumors, OR: 1.76 (1.07,2.89);		

Study (Study design)	Sample size (Cases/controls or cases/total cohort)	Eligibility criteria	Confounding adjustment	Adjusted effect estimate(s) (95% CI)	Dose response	Overall author conclusion
				- ≤25 years vs never used, all tumors, OR: 1.77 (1.12,2.78);<br>- 25 – <38 years vs never used, borderline tumors, OR: 1.98 (1.03,3.79);<br>- 25 – <38 years vs never used, invasive tumors, OR: 1.25 (0.73,2.13);<br>- 25 – <38 years vs never used, all tumors, OR: 1.46 (0.91,2.32);<br>- 38 – <45 years vs never used, borderline tumors, OR: 0.79 (0.23,2.69);<br>- 38 – <45 years vs never used, invasive tumors, OR: 0.88 (0.45,1.72);<br>- 38 – <45 years vs never used, all tumors, OR: 0.87 (0.47,1.61);<br>- 45+ years vs never used, borderline tumors, OR: 1.30 (0.44,3.83);		

Study (Study design)	**Sample size** (Cases/controls or cases/total cohort)	Eligibility criteria	Confounding adjustment	Adjusted effect estimate(s) (95% CI)	Dose response	Overall author conclusion
				- 45+ years vs never used, invasive tumors, OR: 0.72 (0.36,1.43);<br>- 45+ years vs never used, all tumors, OR: 0.82 (0.44,1.52)<br><br>***By years since last use of perineal powder after bathing and histology:***<br>- Current user vs never used, borderline tumor, OR: 1.35 (0.71,2.59);<br>- Current user vs never used, invasive tumor, OR: 1.28 (0.85,1.94);<br>- Current user vs never used, all tumor, OR: 1.30 (0.89,1.91);<br>- ≤12 years vs never used, borderline tumor, OR: 2.11 (0.94,4.77);		

Study (Study design)	Sample size (Cases/controls or cases/total cohort)	Eligibility criteria	Confounding adjustment	Adjusted effect estimate(s) (95% CI)	Dose response	Overall author conclusion
				- ≤12 years vs never used, invasive tumor, OR: 1.59 (0.83,3.02);<br>- ≤12 years vs never used, all tumor, OR: 1.74 (0.98,3.10);<br>- 13-23 years vs never used, borderline tumor, OR: 1.80 (0.75,4.34);<br>- 13-23 years vs never used, invasive tumor, OR: 0.55 (0.24,1.29);<br>- 13-23 years vs never used, all tumor, OR: 0.85 (0.44,1.66);<br>- 24+ years vs never used, borderline tumor, OR: 1.22 (0.45,3.29);<br>- 24+ years vs never used, invasive tumor, OR: 1.10 (0.56,2.17);<br>- 24+ years vs never used, all tumor, OR: 1.13 (0.61,2.08);		

Study (Study design)	Sample size (Cases/controls or cases/total cohort)	Eligibility criteria	Confounding adjustment	Adjusted effect estimate(s) (95% CI)	Dose response	Overall author conclusion
Schildkraut et al., 2016 (Case-control) [53]	584/745	**Inclusion**<br>*Cases*<br>- 20 to 79 years<br>- Newly diagnosed with invasive EOC<br><br>*Controls*<br>- Has at least one intact ovary;<br>- No history of ovarian cancer;<br>- Identified through RDD;<br>- Matched to cases for age and residence<br><br>**Exclusion**<br>- Missing data on body powder use and all covariates	Matched by: age; education ; body mass index; parity ; history of tubal litigation; duration of oral contraceptives ; family history of breast or ovarian cancer in a first-degree relative; menopausal status; hormone therapy; smoking ;	- Ever vs never, OR: 1.39 (1.10–1.76);<br><br>***By location of use:***<br>- Any genital use vs non-use, OR: 1.44 (1.11–1.86);<br>- Any genital use vs non-genital use, OR: 1.31 (0.95–1.79)<br><br>***By date of interview***<br>- Interview <2014, any genital use vs non-use, OR: 1.19 (0.87–1.63);<br>- Interview <2014, any genital use vs non-genital use, OR: 1.26 (0.69–2.32);<br>- Interview >2014, any genital use vs non-use, OR: 2.91 (1.70–4.97);	Significant trend with frequency and duration of use, and number of lifetime applications	Positive association

Study (Study design)	Sample size (Cases/controls or cases/total cohort)	Eligibility criteria	Confounding adjustment	Adjusted effect estimate(s) (95% CI)	Dose response	Overall author conclusion
			hysterectomy; duration of use; occupational exposure.<br><br>Adjusted for: age, study site, education, parity, duration of OC use, tubal ligation, family history of breast or ovarian cancer, BMI and year of interview.	- Interview >2014, any genital use vs non-genital use, OR: 1.26 (0.69–2.32)<br><br>***By frequency of use***<br>- Less than daily, any genital use vs non-use, OR: 1.12 (0.80–1.58);<br>- Daily use, any genital use vs non-genital use, OR: 1.71 (1.26–2.33); *P-value for trend <0.01;*<br>- Less than daily, non-genital use never use, OR: 1.15 (0.78–1.71);<br>- Daily, non-genital use vs never use, OR: 1.53 (1.00–2.35); *P-value for trend 0.09*<br><br>***By duration of use***		

Study (Study design)	Sample size (Cases/controls or cases/total cohort)	Eligibility criteria	Confounding adjustment	Adjusted effect estimate(s) (95% CI)	Dose response	Overall author conclusion
				- <20 years, genital use vs never use, OR: 1.33 (0.95–1.86);<br>- ≥20 years, genital use vs never use, OR: 1.52 (1.11–2.07);<br>*P-value for trend 0.02;*<br>- <20 years, non-genital use vs never use, OR: 1.37 (0.91–2.07);<br>- ≥20 years, non-genital use vs never use, OR: 1.28 (0.85–1.93);<br>*P-value for trend 0.13*<br><br>***By number of lifetime body powder applications***<br>- <3,600 applications, genital use vs never use, OR: 1.16 (0.83–1.63);<br>- ≥3,600 applications, genital use vs never use,		

**Study (Study design)**	**Sample size** (Cases/controls or cases/total cohort)	**Eligibility criteria**	**Confounding adjustment**	**Adjusted effect estimate(s) (95% CI)**	**Dose response**	**Overall author conclusion**
				OR: 1.67 (1.23–2.26);<br>*P-value for trend <0.01;*<br>- <3,600 applications, non-genital use vs never use, OR: 1.35 (0.90–2.03);<br>- >3,600 applications, nongenital use vs never use, OR: 1.30 (0.86–1.97);<br>*P-value for trend 0.14*<br><br>***By menopausal status/ HT use***<br>- Pre-menopausal women, genital use vs never use, OR: 1.50 (0.87–2.57);<br>- Post-menopausal women, genital ever vs never use, OR: 1.41 (1.03–1.92);<br>- Post-menopausal women, HT use, genital		

Study (Study design)	Sample size (Cases/controls or cases/total cohort)	Eligibility criteria	Confounding adjustment	Adjusted effect estimate(s) (95% CI)	Dose response	Overall author conclusion
				use ever vs never, OR: 2.68 (1.33–5.40);<br>- Post-menopausal women, HT never use, genital use ever vs never, OR: 1.24 (0.87–1.79)<br><br>***By cancer histology***<br>- Serous, genital use vs never use, OR: 1.38 (1.03–1.85);<br>Serous, non-genital use vs never use,<br>- OR: 1.10 (0.76-1.58);<br>Non-serous, genital use vs never use, OR: 1.63 (1.04–2.55);<br>- Non-serous, non-genital use vs never use, O:R 2.28 (1.39–3.74)		

**Study (Study design)**	**Sample size** (Cases/controls or cases/total cohort)	**Eligibility criteria**	**Confounding adjustment**	**Adjusted effect estimate(s) (95% CI)**	**Dose response**	**Overall author conclusion**
Tzonou et al, 1993 (Case-control) [10]	189/200	**Inclusion**<br>*Cases*<br>- Residents of Greater Athens;<br>- Less than 75 years;<br>- Underwent surgery for common epithelial ovarian tumor in the 2 major cancer hospitals of Athens between 1989 and 1991<br><br>*Controls*<br>- Hospital controls;<br>- Residents of same area;<br>- Under 75 years;<br>- Visiting patients hospitalized in same wards as the cancer patients at the same time	Matched by residence.<br><br>Adjusted for: age, years of schooling, weight before onset of the disease, age at menarche, menopausal status and age at menopause, parity and age at first birth, tobacco smoking, coffee drinking, consumptio	Perineal talc use vs nonuse, RR: 1.05 (0.28-3.98);	No trend analysis conducted	No association

Study (Study design)	**Sample size** (Cases/controls or cases/total cohort)	Eligibility criteria	Confounding adjustment	Adjusted effect estimate(s) (95% CI)	Dose response	Overall author conclusion
			n of alcoholic beverages, hair dyeing, mutual (analgesics-tranquilizers /hypnotics) confounding influences.			
Whittemore et al, 1988 (Case-control) [4]	188/539	**Inclusion**<br>*Cases*<br>- Residents of Northern California;<br>- Diagnosed with primary EOC at one of the seven hospitals in Santa Clara County or at the University of California, San Francisco Medical Center.	Adjusted for: parity and OC use.	***By method of use*** *(adjusted for parity and OC use)*<br>- Perineal use only vs no use, RR: 1.45 (0.81 - 2.60);<br>- Sanitary pads only vs no use, RR: 0.62 (0.21 - 1.80);<br>- Diaphragm only vs no use, RR: 1.50 (0.63 - 3.58);<br>- Any two of perineum, pads, and diaphragm vs	No trend found	Possible association

**Study (Study design)**	**Sample size** (Cases/controls or cases/total cohort)	**Eligibility criteria**	**Confounding adjustment**	**Adjusted effect estimate(s) (95% CI)**	**Dose response**	**Overall author conclusion**
		Controls<br>- Half of the controls were hospitalized women without overt cancer, and the other half were chosen from the general population by RDD;<br>- Matched to cases on age (+/- 5 years), race, education (> 12 years), term pregnancies, age at menarche, menopausal status and OC use<br><br>**Exclusion**<br>- Bilateral oophorectomy;<br>- Admitted for psychiatric, obstetric, gynecologic, or malignant conditions		no use, RR: 1.36 (0.91 - 2.04);<br>- All three of perineum, pads, and diaphragm vs no use, RR: 0.35 (0.04 - 2.94)<br>***Length of perineal talc use prior to tubal ligation or hysterectomy*** *(adjusted for parity)*<br>- 1 - 9 years, use vs nonuse, RR: 1.60 (1.00 - 2.57);<br>- 10+ years, use vs nonuse, RR: 1.11 (0.74 - 1.65);<br>- *Overall increase in risk for any 10-year increase in duration of use, RR: 1.01; p-value 0.56*		

Study (Study design)	Sample size (Cases/controls or cases/total cohort)	Eligibility criteria	Confounding adjustment	Adjusted effect estimate(s) (95% CI)	Dose response	Overall author conclusion
				Frequency of perineal talc use *(adjusted for parity)*<br>- 1 - 20 times/month, use vs nonuse, RR: 1.27 (0.82 - 1.96);<br>- >20 times/month, use vs nonuse, RR: 1.45 (0.94 - 2.22);<br>- *Overall trend for 30 uses per month, RR: 1.30 (0.88 - 1.92)*<br><br>***By history of surgical sterilization*** *(adjusted for parity)*<br>- No surgical sterilization, use vs nonuse, RR: 1.33 (0.88 - 2.01);<br>- With surgical sterilization, use vs nonuse, RR: 0.75 (0.43 - 1.29);		

Study (Study design)	Sample size (Cases/controls or cases/total cohort)	Eligibility criteria	Confounding adjustment	Adjusted effect estimate(s) (95% CI)	Dose response	Overall author conclusion
				- With and without surgical sterilization, use vs nonuse, RR: 1.37 (0.97 - 1.95)		
Wong et al, 1999 (Case-control) [17]	462/693	**Inclusion**<br>*Cases:*<br>- Treated for epithelial ovarian cancer at Roswell Park Cancer Institute between Oct 1982-October 1995<br>- Identified from the Roswell Park Tumor Registry using ICD codes<br>Controls<br>- Matched to cases by age (+/- 5 years)<br>- Randomly selected from Roswell Park Tumor Registry<br>- Treated for nongynecologic	Matched by: age.<br><br>Adjusted for: parity, OC use, smoking history, family history of epithelial ovarian cancer, age at menarche, menopausal status, income, education, geographic location, and history of tubal ligation	***By site of use***<br>- Sanitary napkin use vs never used, OR: 0.9 (0.4, 2.0);<br>- Genital or thigh area use vs never used, OR: 1.0 (0.8,1.3);<br>- Genital/thigh area use and sanitary napkin use vs never used, OR: 1.1 (0.7, 1.7)<br><br>***By duration of use***<br>- 1-9 years vs no use, OR: 0.9 (0.6, 1.5);<br>- 10–19 years vs no use, OR: 1.4 (0.9, 2.2);	No trend found	No association

Study (Study design)	**Sample size** (Cases/controls or cases/total cohort)	Eligibility criteria	Confounding adjustment	Adjusted effect estimate(s) (95% CI)	Dose response	Overall author conclusion
		malignancies during the same period as cases	or hysterectomy	- 20+ years vs no use, OR: 0.9 (0.6, 1.2)<br><br>***By genital tract obstruction***<br>- No history of genital tract interruption vs history of tubal ligation/ hysterectomy, OR: 1.2 (0.8, 1.6);<br>- History of tubal ligation or hysterectomy vs no history, OR 0.8 (0.5, 1.2);<br>- History of hysterectomy vs no history, OR: 0.9 (0.4, 2.2)		
Wu et al, 2009 (Case-control) **[47]**	609/688	**Inclusion**<br>*Cases*<br>- English speaking residents of Los Angeles County between 18 and 74 years old<br>-histologically confirmed	Matched by: age and ethnicity.<br><br>Adjusted for: race/ethnicit	***By location of talc use***<br>- Talc use on body vs nonuse, RR: 1.48 (1.15,1.91);<br>- Talc use on non-perineal area vs nonuse, RR: 1.43 (1.03,1.98);	Significant trend for frequency and duration of use, and number of	Positive association

Study (Study design)	Sample size (Cases/controls or cases/total cohort)	Eligibility criteria	Confounding adjustment	Adjusted effect estimate(s) (95% CI)	Dose response	Overall author conclusion
		invasive or borderline ovarian cancers first diagnosed from 1998 to 2002.<br>-identified by the Cancer Surveillance Program (CSP), part of the National Cancer Institute's Surveillance, Epidemiology and End Results (SEER) Program, covering all residents of Los Angeles County<br>*Controls*<br>-residents of LA Country<br>-at least one intact ovary<br>-no previous cancer (except non melanoma skin cancer)<br>- selected using a neighbourhood control selection algorithm<br>- matched on race/ethnicity, year of birth (+/- 5)	y, age, education, tubal ligation, family history of breast/ovarian cancer, menopausal status, OC use and parity.	- Talc use on perineal area vs nonuse, RR: 1.53 (1.13, 2.09);<br><br>***By method of use***<br>- talc use on sanitary vs nonuse, RR: 1.61 (0.93, 2.78);<br>- talc use on underwear vs nonuse, RR: 1.71, (0.99, 2.97);<br>- talc use on diaphragm/cervical caps, RR: 1.14 (0.46, 2.87);<br><br>***By histology of cancer***<br>- serous ovarian cancer, talc use vs nonuse, RR: 1.70 (1.27, 2.28);<br>- mucinous ovarian cancer, talc use vs	lifetime applications	

**Study (Study design)**	**Sample size** (Cases/controls or cases/total cohort)	**Eligibility criteria**	**Confounding adjustment**	**Adjusted effect estimate(s) (95% CI)**	**Dose response**	**Overall author conclusion**
		**Exclusion**<br>*Cases*<br>-had died, moved away or were too ill for interview<br>-could not be located<br>-later identified not to have ovarian cancer<br>-had previous cancer (before ovarian) (excluding non-melanoma skin cancer)		nonuse, RR: 0.99 (95% CI NR[5]);<br>- clear/endometrioid ovarian cancer, talc use vs nonuse, RR: 1.19 (95% CI NR);<br>- other cell types ovarian cancer, talc use vs nonuse, RR: 1.46 (95% CI NR);<br><br>***By cancer stage***<br>- Invasive cancers, talc use vs nonuse, RR: 1.31 (0.85, 2.01);<br>- Localized stage, talc use vs nonuse, RR: 1.66 (1.22, 2.26);		

[5] NR: not reported

Study (Study design)	Sample size (Cases/controls or cases/total cohort)	Eligibility criteria	Confounding adjustment	Adjusted effect estimate(s) (95% CI)	Dose response	Overall author conclusion
				- Advanced stage and LMP, talc use vs nonuse, RR: 1.32 (0.88, 2.22);<br><br>***By frequency and duration of talc use***<br>- 1 to 20 yrs and up to 10 times/month vs nonuse, RR: 1.36 (0.79, 2.32);<br>- 1 to 20 yrs and 11 to 30 times/month vs nonuse, RR: 1.16 (0.63, 2.12);<br>- 1 to 20 yrs and 30+ times/month vs nonuse, RR: 1.23 (0.63, 2.41);<br>- >20 yrs and 10 times/month vs nonuse, RR: 1.27 (0.80, 2.01);<br>- >20 yrs and 11 to 30 times/month vs nonuse, RR 1.57 (0.99, 2.50);<br>- >20 yrs and >30 times/month vs nonuse,		

Study (Study design)	Sample size (Cases/controls or cases/total cohort)	Eligibility criteria	Confounding adjustment	Adjusted effect estimate(s) (95% CI)	Dose response	Overall author conclusion
				RR: 2.08 (1.34, 3.23);<br>*P value for trend 0.032*<br><br>***By total times of talc use***<br>- up to 5,200 times vs nonuse, RR: 1.20 (0.77, 1.88);<br>- 5,201 - 15600 times vs nonuse, RR: 1.38 (0.87, 2.20);<br>- 15,601 - 52000 times vs nonuse, RR: 1.34 (0.89, 2.02);<br>- > 52,000 times vs nonuse, RR: 1.99 (1.34, 2.96);<br>*P value for trend 0.0004*<br><br>***By total times of talc use before 1975***		

Study (Study design)	Sample size (Cases/controls or cases/total cohort)	Eligibility criteria	Confounding adjustment	Adjusted effect estimate(s) (95% CI)	Dose response	Overall author conclusion
				- up to 5,200 times vs nonuse, RR: 0.84 (0.47, 1.51);<br>- 5,201 - 15,600 times vs nonuse, RR: 1.41 (0.79, 2.53);<br>- 15,601 - 52,000 times vs nonuse, RR: 1.45 (0.91, 2.31);<br>- > 52,000 times vs nonuse, RR: 1.99 1.93 (1.29, 2.88);<br><br>***By total times of talc use before 1975***<br>- up to 5,200 times vs nonuse, RR: 1.95 (0.98, 3.89);<br>- 5,201 - 15,600 times vs nonuse, RR: 1.17 (0.56, 2.48);		

Study (Study design)	**Sample size** (Cases/controls or cases/total cohort)	Eligibility criteria	Confounding adjustment	Adjusted effect estimate(s) (95% CI)	Dose response	Overall author conclusion
				- >15,600 times vs nonuse, RR: 1.45 (0.91, 2.31);<br>- > 52,000 times vs nonuse, RR: 0.98 (0.45, 2.13);		
Wu et al, 2015 (Case-control) **[51]**	1,701/2,391	**Inclusion**<br>*Cases:*<br>- newly diagnosed with invasive EOC<br>- identified from USC Cancer Surveillance Program between 1992 and 2008.<br>- between 18-79 years of age at diagnosis (up to age 74 for cases diagnosed before 2003)<br>- residents of LA county<br>- non-Hispanic white, Hispanic, or African American race<br>*Controls:*<br>- residents of LA Country	Matched by: age (+/-5 years), neighborhood and race.<br><br>Adjusted for: nulliparity, OC use, tubal ligation, history of endometriosis, family history of ovarian cancer,	***By use/nonuse***<br>- All races, genital talc use, use vs (non-use or <1 yr use), OR: 1.46 (1.27, 1.69);<br>- Non-Hispanic whites, genital talc use, use vs (non-use or <1 yr use), OR: 1.41 (1.21, 1.67);<br>- Hispanics, genital talc use, use vs (non-use or <1 yr use), OR: 1.77 (1.20, 2.62);<br>- African Americans, genital talc use, use vs (non-use or <1 yr use), OR: 1.56 (0.80, 3.04);	No trend analysis conducted	Positive association among Hispanics and non-Hispanic whites, but not African Americans.

Study (Study design)	**Sample size** (Cases/controls or cases/total cohort)	Eligibility criteria	Confounding adjustment	Adjusted effect estimate(s) (95% CI)	Dose response	Overall author conclusion
		- has at least one intact ovary<br>- selected using a neighbourhood control selection algorithm<br>- matched on race/ethnicity, year of birth (+/- 5)<br><br>**Exclusion**<br>-cases who had previous cancer or prior bilateral oophorectomy	menopausal status, age at menarche, HT use, BMI, family income and education.	***By duration***<br>- All races, genital talc use, per 5 yrs use vs (non-use or <1 yr use), OR: 1.14 (1.09, 1.20);<br>- Non-Hispanic whites, genital talc use, per 5 yrs use vs (non-use or <1 yr use, OR: 1.14 (1.08, 1.21);<br>- Hispanics, genital talc use, per 5 yrs use vs (non-use or <1 yr use), OR: 1.18 (1.02, 1.36);<br>- African Americans, genital talc use, Per 5 yrs use vs (non-use or <1 yr use), OR: 1.15 (0.90, 1.47);		

Supplementary Material VII: Strengths and limitations of included studies identified by the original study authors and the authors of the current review

Study	Strengths reported by original study authors	Limitations reported by original study authors	Strengths noted by authors of the current review	Limitations noted by authors of the current review
Booth et al. (1989), UK [5]		No information on duration of use /exposure to perineal talc		
Chang and Risch (1997), Canada [12]		- No information on talc concentration in powders used<br>- possible errors in talc use self-reports<br>- Failure to interview all eligible subjects		
Chen et al. (1992), China [7]		- Exclusion of some ovarian cancer cases due to the nature of cancer registration in China<br>- Exclusion of controls with current health problems, some ovarian cancer.<br>- The high rate of loss due to deaths particularly in the cases group.		Study did not provide information whether or not they adjusted for confounders such as age and age at menstruation.

Study	Strengths reported by original study authors	Limitations reported by original study authors	Strengths noted by authors of the current review	Limitations noted by authors of the current review
Cook et al. (1997), USA [13]		- Low proportion of eligible women who participated in the study.<br>- Recall bias for powder usage.<br>- Lack of ascertainment on whether or not perineal powder application correctly estimates actual exposure<br>- Difference in the used powder brands, lots of the same brand, or chemical composition and impurities among different brands.		
Cramer et al. (1982), USA [2]	Inclusion of more than 50% of ovarian cancer cases diagnosed in Boston residents in the study period.	Selection bias due to high refusal rate by controls	Adjustment to many potential confounders	
Cramer et al. (2016), USA [52]	The authors address recall bias and confounding and find no evidence that these explain the observed association.	Inherent limitation in quantifying a dose-response is "a lack of metrics for how much talc is in an 'application', how much enters the vagina, and how much reaches the upper genital tract where, presumably, any deleterious effect is mediated."	- Availability of data on a large number of potential confounders.<br>- Analyses of effect modification of the association between talc	Low response rate for controls

Study	Strengths reported by original study authors	Limitations reported by original study authors	Strengths noted by authors of the current review	Limitations noted by authors of the current review
			and ovarian cancer.	
Gates et al. (2008), USA [43]	- Analysis and the assessment of gene-talc interactions in two independent study populations, one with a large number of cases and the other with prospective data on talc use and ovarian cancer incidence.<br>- Exposure definition of genital talc use at least once a week may have decreased the influence of recall bias in this analysis, because habitual talc use is likely to be recalled more accurately than sporadic use.			- Exclusion of non-English speaking women<br>- Study was more focused on talc-gene interactions

Study	Strengths reported by original study authors	Limitations reported by original study authors	Strengths noted by authors of the current review	Limitations noted by authors of the current review
Gates et al. (2010), USA [54]	- Prospective data with repeated measures for most exposures<br>- Large combined study population<br>- Analytic methods allowed for estimation of separate associations with each subtype simultaneously,	- Limited number of cases of certain histologic subtypes such as endometrioid or mucinous epithelial cancers<br>- Incomplete data for a few exposures such as talc use may have influenced the observed association with ovarian cancer<br>- Use of a single summary measure for certain exposures may have limited the ability to detect an association	-	- Study was focused on all risk factors of ovarian cancer and not on genital talc use, which did not allow the examination of its association with ovarian cancer, or adjustment to potential confounders.
Gertig et al. (2000), USA [20]	- Prospective analysis<br>- Low potential for recall bias since talc exposure was ascertained before cancer<br>- Controlled for known factors	- Talc use was classified as ever use<br>- No information on age at which women began using talc or the duration of use.<br>- Unable to assess the potential effect of talc use before first pregnancy<br>- Relatively short follow up period, may be inadequate to detect an association<br>- Potential for misclassification of exposure due to question on frequency of ever use, higher prevalence of talc use compared to other studies		

Study	Strengths reported by original study authors	Limitations reported by original study authors	Strengths noted by authors of the current review	Limitations noted by authors of the current review
Godard et al. (1998), Canada [15]	Looked into relatives cancer history	-	Stratified results by familial and sporadic cancer cases (sporadic meaning negative family history of cancer)	- Relatively small sample size<br>- Talc assessed as use vs never use, potential for misclassification and recall bias<br>- Focus of paper not on talc<br>- Adjusting in analyses is not clear<br>- Did not adjust for parity
Gonzalez et al. (2016), USA [56]	- Large prospective cohort study<br>- Many potentially confounding factors were controlled for.<br>- Exposure information was complete, with only 2% missing the personal care products questionnaire entirely.	Few factors might contribute to null/inverse findings and should be considered with caution:<br>- "Ovarian" cancer was designated as ovarian (88%), fallopian (5%), peritoneal (3%), or those designated as uncertain but either ovarian, fallopian, or peritoneal (5%). No analysis was conducted for cases of ovarian cancer only.<br>- The exposure was categorized based on the 12 months prior to enrollment as a dichotomous ever/never factor		

Study	Strengths reported by original study authors	Limitations reported by original study authors	Strengths noted by authors of the current review	Limitations noted by authors of the current review
		rather than a quantitative measure of total applications.<br>- Follow up period was not accounted for the latency of ovarian cancer (15-20 years) - inadequate.<br>- The sub-cohort of women mainly included women with hysterectomy and/or tubal ligation, in addition, women with a previous history of breast cancer were excluded, limiting participants who were at increased risk for ovarian cancer.<br>- Detailed information about specific products used in douching was not collected and it was impossible to estimate exposure to other chemical compounds (e.g., contamination by asbestos, etc.).<br>- Limited details and discussion are available on pre-pubertal use of talc powder which indicates on existing and		

Study	Strengths reported by original study authors	Limitations reported by original study authors	Strengths noted by authors of the current review	Limitations noted by authors of the current review
		increased risk of ovarian cancer.		
Green et al. (1997), Australia [41]		Potential for recall bias.		
Harlow et al. (1989), USA [6]		- Inability to include approximately 30 % of potentially eligible cases and controls due to refusals and other reasons for nonparticipation.<br>- Lack of information on the timing and duration of use of		Researchers decided to include in the analysis 33 cases whose tumors had not been reviewed by the pathologists.

Study	Strengths reported by original study authors	Limitations reported by original study authors	Strengths noted by authors of the current review	Limitations noted by authors of the current review
		each type of talc-containing powder.		
Harlow et al. (1992), USA [8]		- Possible over- or under-reporting of talc exposure since researchers were able to interview 69% of eligible cases and 81% of eligible controls.<br>- Most subjects reported use as just "baby powder", which rendered the researchers unable to confirm a previous finding that powders with "deodorizing" agents were associated with particular risk.	A highly focused study examining in full details the association of perineal talc exposure with ovarian cancer	Study limited to hospitals in one city and to one racial/linguistic subgroup (English-speaking white females).
Hartge et al. (1983), USA [3]		Potential for:<br>- Selection bias<br>- Observation bias<br>- Recall bias		
Houghton et al. (2014), USA [55]	- Large sample size with large number of cancer cases<br>- Prospective design<br>- Good case ascertainment	- Minor potential for including women who are not at risk for ovarian cancer, ex. those that had oophorectomy after baseline	- As the focus was on talc and ovarian cancer, authors were able to stratify results by	-

Study	Strengths reported by original study authors	Limitations reported by original study authors	Strengths noted by authors of the current review	Limitations noted by authors of the current review
	- Detailed information on ovarian cancer risk factors<br>- Duration of powder use info collected<br>- Compared risk by age group, to determine whether asbestos contamination could explain ovarian cancer risk, found no evidence (because duration lapsed 1976 in some cases)	- Potential for non-differential mis-classifications of exposure<br>- Exposure information not collected after baseline, potential for never users to become users<br>- No information on frequency of use<br>- Small sizes in subgroup analysis of cancer subtype<br>- Exposure considered as perineal use of powder, not specific to talc (minor limitation)	subtype, and method of talc use<br>- Adjustment of potential confounders	
Kurta et al. (2012), USA [49]	- Large sample size<br>- Detailed info on reproductive and medical histories<br>- Stratified analyses		-	- Objective of this study is infertility drugs and ovarian cancer.<br>- Exposure was assessed as "ever use of talc powder", no indication of years used<br>- This paper does not stratify by type of cancer.<br>- Authors suggest that their findings mean increased risk of talc

Study	Strengths reported by original study authors	Limitations reported by original study authors	Strengths noted by authors of the current review	Limitations noted by authors of the current review
				on ovarian cancer, though talc exposure assessment was ever used talc --Too strong of a statement based on their OR.
Langseth & Kjaerheim (2004), Norway [42]	- Based on a large cohort of pulp and paper workers<br>- Complete job description and exposure histories	- Small sample, low statistical power<br>- Not all participants answered the questions on hygienic talc -- uncertainty in these results	-	- Analysis on perineal exposure is limited by small sub-sample<br>- Adjusted estimates not reported (though authors report no differences between adjusted and nonadjusted)<br>- Focus of paper is on occupational exposure of talc<br>- Age of participants not reported<br>- Follow up duration not clear

Study	Strengths reported by original study authors	Limitations reported by original study authors	Strengths noted by authors of the current review	Limitations noted by authors of the current review
Rosenblatt et al. (1992), USA [9]	Large size, population based, nationwide	- Potential selection bias or over representation of healthy controls due to low response rate among controls<br>- Medical conditions based on self-reports	- Stratified analyses by histology and duration of use<br>- Analysis allowed trend analysis	Self reported exposure of powder or talcum powder/no info on contents of powder
Rosenblatt et al. (2011), USA [48]	Consistent with prior findings	- Relatively small sample size<br>- Relatively low response rate<br>- Potential for recall bias for talc use<br>- Not able to differentiate between use of perineal powders containing talc and those containing cornstarch, which may have driven the odds ratio toward the null.<br>- Type of application, direct perineal use, dusting sanitary napkins, underwear and diaphragms was also not assessed.	Many subgroup analyses	
Schildkraut et al. (2016), USA [53]		- Small sample size made it difficult to ascertain which associations were true vs. chance findings, and to conduct subgroup analysis or yield meaningful results.		- Potential selection bias stemming from inclusion of patients from only one state (North Carolina)

Study	Strengths reported by original study authors	Limitations reported by original study authors	Strengths noted by authors of the current review	Limitations noted by authors of the current review
		- Small African-American women participation<br>- Participation bias, where there was a possibility that cases who participated differed from those who did not.		- Limited focus on talc and its association with ovarian cancer.
Tzonou et al. (1993), Greece [10]		- Low participation rates among cases and controls.<br>- Recall bias<br>- Many of the effect sizes were modest.		
Rosenblatt et al. (1992), USA [9]		- Small sample size<br>- Potential selection bias stemming from inclusion of patients from only one hospital (Johns Hopkins Hospital, Baltimore, Maryland, USA).<br>- Difficulty of finding matching controls	- Adjustment for many potential confounders	
Rosenblatt et al. (2011), USA [48]	Large population-based study	- Potential for exposure misclassification<br>- Nonresponse among cases and controls may affect findings<br>- Results will always be ambiguous in the absence of details of the composition of powders used	- Stratified and detailed findings presented	

Study	Strengths reported by original study authors	Limitations reported by original study authors	Strengths noted by authors of the current review	Limitations noted by authors of the current review
Schildkraut et al. (2016), USA [53]		- The possibility of differential misclassification of exposure exists and residual confounding.<br>- "Because of the relatively small number of women who reported having only used genital powder (43 cases and 44 controls), this exposure category was merged with those who reported use of both non-genital and genital powder, creating an exposure category of "any" genital powder use."	-	
Tzonou et al. (1993), Greece [10]		- Relatively small sample size, limited power<br>- Potential for selection and information bias<br>- Frequency of talc use was low in the study population	-	- Controls not matched on age<br>- Focus was not talc, thus potential for exposure misclassification<br>- No mention of confirmation of ovarian cancer diagnosis other than that patients had prior surgery for it

Study	Strengths reported by original study authors	Limitations reported by original study authors	Strengths noted by authors of the current review	Limitations noted by authors of the current review
				- Very low frequency of talc use in sample
Whittemore et al. (1988), USA [4]		- Failure to interview all eligible ovarian cancer patients and a completely random sample of controls<br>- Combining the two types of control groups.<br>- Confounding by differential talc use among women with characteristics predictive of ovarian cancer (such as menstruation).<br>- Random error in reported talc use, which tends to attenuate relative risk estimates.		
Wong et al. (1999, 2009), USA [17, 47]	- Adjustment for many confounders,	- Potential for recall bias, many subjects stated they do not remember talc use in the past<br>- No assessment of talc use on condoms and diaphragms		- No mention of case ascertainment other than from registry<br>- Hospital controls
Wu et al. (2009), USA [47]	- Assessed dose response<br>- Collected info on frequency and duration of use		- Inclusive of different races	- Relatively low number of subjects for the subgroup analyses (in the range of 17-70 subjects)

Study	Strengths reported by original study authors	Limitations reported by original study authors	Strengths noted by authors of the current review	Limitations noted by authors of the current review
Wu et al. (2015), USA [51]	- Focused on Hispanic women (fills knowledge gap)<br>- Comparability of risk factors among 3 different races<br>- Findings from 4 studies, all used same questionnaires	- Modest sample sizes for Hispanics and African Americans<br>- Sample sizes did not allow for analysis by histologic types<br>- Modest response rate (though comparable to other similar studies)	- Adjustment for many potential confounders	- Potential for recall bias in the number of years of talc use<br>- No measure of frequency of talc use<br>- Duration of use among women extends to period before talc was asbestos free/ potential confounder

Critical Review of the Association between Perineal Use of Talc and Risk of Ovarian Cancer

Supplementary Material VIII, IX, X

Non-Human Studies

Supplementary Material VIII: Literature search strategy for non-human studies

VIII.1. Literature search strategy and identification of relevant non-human studies

We conducted a critical review of relevant non-human studies identified in three major bibliographic databases to identify potentially relevant animal and in vitro studies: Embase, MEDLINE and Toxline. Search strategies included combinations of subject headings and keywords relevant to talc, carcinogenesis and toxicokinetics (See Supplementary Material VIII.3). Additional searches of reference lists from selected articles were conducted to identify other studies focusing on the evaluation of perineal application of talc and ovarian cancer. In this regard, all references cited in the 2010 IARC[6] monograph [1] were considered as relevant and appropriate for inclusion in the current review, and were obtained and reviewed. Search results were imported into EndNote and duplicates were removed. A priori inclusion/exclusion criteria for studies selection were established (Supplementary Material IX).

Only studies that focused on perineal/genital exposure to talc powder, as a general term, were included. For outcomes, studies that focused on any type of cancer but with a focus on ovarian cancer and perineal exposure were considered. Available animal studies on talc administered by parenteral routes were included, along with in vitro studies, as relevant to identification of the carcinogenic potential and mechanism of

[6] IARC: International Agency for Research on Cancer

action of talc. All retrieved articles were examined for relevance, reliability and overall quality using the Klimisch scoring system [65, 66] (Supplementary Material VII, VIII and IX). Accordingly, studies are classified into one of the following four categories of reliability: 1) reliable without restriction, 2) reliable with restrictions, 3) not reliable and 4) not assignable. Additionally, category (5) is assigned to special studies focusing on pharmacologic or mechanistic investigations without particular relevance to hazard identification or risk assessment.

VIII.2. Database search strategy

Embase[7]

	Search Terms	Results
1	talc/	4565
2	talc*.mp.	5748
3	genital powder.mp.	6
4	perineal powder.mp.	7
5	baby powder.mp.I	61
6	sclerosol.mp.	8
7	1 or 2 or 3 or 4 or 5 or 6	5781
8	neoplasm/	768403
9	neoplasm*.mp.	1003529
10	tumour*.mp.	320210
11	cancer*.mp.	2871964
12	tumor*.mp.	2593976
13	cancer*.mp.	2871964
14	carcino*.mp.	1323560
15	carcinoma/	82459
16	malignan*.mp.	716411
17	mutagenic agent/	13517
18	mutagen testing/	6430
19	mutation/	382448
20	mutagen*.mp.	166584
21	mutation*.mp.	903543
22	genotox*.mp.	43339
23	toxicogenetics/c	980
24	toxicogenetic*.mp.	1044
25	micronucle*.mp.	16989

[7] 1974 to 2017 April 20

	Search Terms	Results
26	electrophil*.mp.	19234
27	dna repair/	68607
28	(dna adj3 repair).mp.	90018
29	gene* instability.mp.	4945
30	genomic instability.mp.	21096
31	genomic instability/	18186
32	epigenetic*.mp.	96313
33	oxidative stress.mp.	260788
34	oxidative stress/	240539
35	dna damage/	101098
36	dna damage.mp.	118540
37	chronic inflamm*.mp.	80548
38	immortaliz*.mp.	26309
39	Immunosuppressive Agents/	65313
40	Immunosuppressive Agent*.mp.	80603
41	receptor mediated effect*.mp.	1159
42	cell transformation/	48124
43	cell* transformation.mp.	57229
44	cell proliferation/	419064
45	cell* proliferation.mp.	470591
46	cell death/	152901
47	cell* death.mp.	213136
48	structure activity relation/	161927
49	structure activity relation*.mp.	179681
50	toxicokinetics/	11310
51	toxicokinetic*.mp.	12734
52	adme.mp.	3279
53	pharmacokinetics/	223526
54	pharmacokinetic*.mp.	558942
55	8 or 9 or 10 or 11 or 12 or 13 or 14 or 15 or 16 or 17 or 18 or 19 or 20 or 21 or 22 or 23 or 24 or 25 or 26 or 27 or 28 or 29 or 30 or 31 or	6629853

	Search Terms	Results
	32 or 33 or 34 or 35 or 36 or 37 or 38 or 39 or 40 or 41 or 42 or 43 or 44 or 45 or 46 or 47 or 48 or 49 or 50 or 51 or 52 or 53 or 54	
56	7 and 55	1904

Medline Ovid[8]

	Search Terms	Results
1	talc/	1993
2	talc.mp.	2985
3	genital powder.mp.	5
4	perineal powder.mp.	6
5	baby powder.mp.	55
6	sclerosol.mp.	1
7	or/1-6	3017
8	Neoplasms/	378784
9	neoplasm*.mp.	2517702
10	tumor*.mp.	1650827
11	tumour*.mp.	242060
12	cancer*.mp.	1471025
13	carcino*.mp.	913137
14	Carcinoma/	84533
15	malignan*.mp.	500768
16	Mutagens/	28271
17	mutagen*.mp.	185510
18	Mutagenicity Tests/	16435
19	mutation/	373941
20	mutation*.mp.	734065
21	genotox*.mp.	29353
22	toxicogenetics/	751
23	toxicogenetic*.mp.	823
24	micronucle*.mp.	13946
25	electrophil*.mp.	12972
26	Carcinogenesis/	6766

[8] *MEDLINE(R) Epub Ahead of Print, In-Process & Other Non-Indexed Citations, Ovid MEDLINE(R) Daily and Ovid MEDLINE(R) 1946 to Present.*

	Search Terms	Results
27	dna damage/	57731
28	dna damage.mp.	87181
29	oxidative stress/	103324
30	oxidative stress.mp.	170284
31	epigenetic*.mp.	59583
32	genomic instability/	6150
33	genomic instability.mp.	11411
34	gene* instability.mp.	3775
35	(dna adj3 repair).mp.	75410
36	dna repair/	45004
37	chronic inflamm*.mp.	50075
38	immortaliz*.mp.	18498
39	Immunosuppressive Agents/	87244
40	Immunosuppressive Agent*.mp.	92070
41	receptor mediated effect*.mp.	1004
42	Cell Transformation, Neoplastic/	56678
43	cell* transformation.mp.	73838
44	cell proliferation/	169098
45	cell* proliferation.mp.	290047
46	cell death/	40123
47	cell* death.mp.	156718
48	Structure-Activity Relationship/	157890
49	structure activity relation*.mp.	175030
50	toxicokinetics/	250
51	toxicokinetic*.mp.	3630
52	adme.mp.	1902
53	pharmacokinetics/	8097
54	pharmacokinetic*.mp.	144889
55	or/8-54	5033642
56	7 and 55	960

Toxline Proquest

Set#	Searched for	Databases	Results
S1	all(talc* OR baby NEAR/1 powder OR sclerose OR perineal NEAR/1 powder OR genital NEAR/1 powder) AND peer(yes)		2305°
S2	All(Neoplasm* OR tumo* OR cancer* OR Carcino* OR malignan* OR Mutagenic NEAR/1 agent OR mutagen NEAR/1 testing OR mutation OR mutagenicity OR mutag* OR genotox* OR dominant NEAR/1 lethal OR chromosome NEAR/1 abberat* OR micronucle*) AND (Neoplasm? OR tumo? OR cancer* OR Carcino? OR malignan? OR Mutagenic agent OR mutagen testing OR mutation OR mutagen? OR genotox? OR chromosom? abberat? OR micronucle?) AND peer(yes)	American Periodicals, ARTbibliographies Modern (ABM), British Periodicals, Business Premium Collection, Canadian Business & Current Affairs Database, Canadian Major Dailies, Canadian Research Index, Colonial State Papers, ComDisDome, Digital National Security Archive, ebrary® e-books, ebrary® e-books, EconLit, ERIC, GenderWatch, Index Islamicus, International Bibliography of Art (IBA), Linguistics and Language Behavior Abstracts (LLBA), MLA International Bibliography, Music Periodicals Database, Nursing & Allied Health Database, Periodicals Archive Online, Philosopher's Index, Physical Education Index, PILOTS: Published International Literature On Traumatic Stress, Political Science Database, PRISMA Database, PRISMA Database with HAPI Index, ProQuest Dissertations & Theses: UK & Ireland, ProQuest Dissertations & Theses Global, ProQuest Historical Annual Reports, ProQuest Historical Newspapers: The Globe and Mail, ProQuest Sociology Collection, PsycARTICLES, PsycBOOKS, Social Services Abstracts, The Annual Register: A Record of	372956°
S3	All(electrophil* or DNA near/1 repair OR gene* near/1 instability OR genomic near/1 instability OR epigenetic* OR oxidative near/1 stress OR DNA near/1 damage OR chronic near/1 inflammation OR immortaliz* OR immunosuppresive near/1 agent OR receptor near/1 mediated near/1 effect OR cell near/1 transform* OR cell near/1 proliferation OR cell near/1 death OR structure near/1 activity near/1 relation*) AND peer(yes)		65551°
S4	(toxicokinetic* OR adme OR pharmacokinetic*) AND peer(yes)		73682°
S5	S1 and (S2 or S3 or S4)		146°

Set#	Searched for	Databases	Results
		World Events, The Vogue Archive	

° Duplicates are removed from your search and from your result count.

VIII.3. Database search results

After removal of duplicates, the bibliographic database searches on non-human studies initially yielded 1,165 references. The 51 retained animal studies focus on the carcinogenicity of talc, mechanism of action, and toxicokinetics.

Supplementary Material IX: Inclusion-exclusion criteria for non-human studies

Relevant areas	Include	Details
**Exposure**	Exposure	Talc, Magnesium silicate monohydrate,<br>CAS RN 14807-96-6.<br><br>*Include*:<br>Perineal/genital exposure; inhalation, oral, dermal routes of exposure and parenteral administration (intratracheal, intraperitoneal, intrapeural, subcutaneous injections.
**Toxicokinetics**	ADME	*Include:*<br>Animal (and in-vitro studies)
**Adverse Health Effects**	Health Effects – Animals	*Include*: All available information on different types of ovarian cancer, genotoxicity and carcinogenicity studies on talc (including mechanism of action studies).<br><br>*Exclude*: The animal models not suitable for human health risk assessment (ruminant species, birds, fish, reptiles, amphibians).
	In-vitro studies	*Include:* Bacterial and animal cell lines suitable for human health risk assessment.<br><br>*Exclude*: Animal models not suitable for human health risk assessment (birds, fish, reptiles, amphibians) and their respective cell lines.
	Mechanism of action	*Include:*<br>*A*ll available information on mechanism of genotoxicity and carcinogenicity.
**Talc species**	*Include:*	*Details:*
	Talc	Talc powder, type of talc powder (if possible).
**Document types**	***Include:***	***Sources:***
	Peer-reviewed publications, reviews	MEDLINE, EMBASE, TOXLINE

Relevant areas	Include	Details
	Reports from the International Public Health Regulatory Committee /Government/ Other Organizations	Agency for Toxic Substances and Disease Registry (ATSDR), Food and Agriculture Organization of the United Nations/World Health Organization (FAO/WHO), Health Canada, Health Council of the Netherlands, the Netherlands (Health Council of the Netherlands), Environment Protection Agency, United states (US EPA), European Chemical Agency (ECHA), European Food Safety Authority (EFSA), German Research Foundation (GRF), Germany, International Agency for Research on Cancer (IARC), International Toxicity Estimates for Risk (ITER)/Toxicology Excellence for Risk Assessment (TERA), United States, Joint FAO/WHO Expert Committee on Food Additives (JECFA), the National Toxicology Program, United States (US NTP), National Industrial Chemicals Notification and Assessment Scheme (NICNAS) of Australia, National Institute for Public Health and the Environment, the Netherlands (RIVM), New Zealand Ministry of Health, World Health Organization (WHO).
	Exclude:	Editorials/Letters/Comments with no primary data.
**Language**	No exclusions based on language at the screening stage.	

Supplementary Material X: Summary of non-human studies included in this review

X.1. Animal studies on talc (published up to 2006)

Reference	Test material	Animal model	Results	Perineal route of exposure (Yes/No)
National Toxicology Program (1993) [67]	Talc (non-asbestiform).<br>CAS No 14807-6.<br>High purity, microtalc, MP (10-52 Grade), max particle size 10 µm, contained no tremolite or asbestiforms; free of silica - 1 particle of silica was detected in 1,466 particles examined;<br>obtained from Walsh and Associates, USA.	Male and female Fisher rats (n=50/sex/group).<br>Exposure route: inhalation, 6h/5days/week.<br>Concentration: 0, 6, 18 mg/m^3 for 6h/5days per week.<br>Duration of exposure: 103-122 weeks.	Tumours of ovaries or other female reproductive organs:<br>No neoplastic macro- or micro-abnormalities were reported in ovaries, uterus or Fallopian tubes of talc-exposed rats.<br><u>Neoplastic effects in lungs</u><br>Female rats:<br>*The incidence of alveolar/bronchiolar adenoma*:<br>Control: 1/50<br>Low-dose: 0/48<br>High dose: 9/50<br>*The incidence of alveolarbronchiolar carcinoma*:<br>Control: 0/50<br>Low-dose: 0/48	No

Reference	Test material	Animal model	Results	Perineal route of exposure (Yes/No)
			High dose: 5/50<br><br>*The incidence of alveolar bronchiolar adenoma or carcinoma*:<br>Control: 1/50<br>Low-dose: 0/48<br>High-dose: 13/50<br>Adrenal gland<br>*The incidence of adrenal medulla: benign or malignant pheochromocytoma*:<br>Control group: 13/48<br>Low dose: 14/47<br>High dose group: 23/49<br><br>Males<br>Adrenal gland<br>*The incidence rate of adrenal medulla: benign or malignant pheochromocytoma*:<br>Control: 26/49<br>Low dose: 32/48<br>High dose: 37/47	

Reference	Test material	Animal model	Results	Perineal route of exposure (Yes/No)
			<u>Non-neoplastic effects in lungs</u><br>Females<br><u>Lung</u><br>*Granulomatous inflammation*:<br>Control: 2/50 (females) – 2/49 (males)<br>Low dose: 47/48 (females) – 50/50 (males)<br>High dose:50/50) (females) – 49/50 (males);<br><br>*Interstitial fibrosis*:<br>Control: 1/50 (females) – 1/49 (males)<br>Low dose:24/48 (females) – 16/50 (males)<br>High dose: 44/50 (females) – 33/50 (males)<br><br>*Alveolar epithelial hyperplasia*:<br>Control: 2/50 (females) – 5/49 (males)<br>Low dose: 27/48 (females) – 26/50 (males)	

Reference	Test material	Animal model	Results	Perineal route of exposure (Yes/No)
			High dose: 47/50 (females) – 38/50 (males)<br><br>*Cyst:*<br>Control: 0/50 (females) – 0/50 (males)<br>Low dose: 1/48 (females) – 0/50 (males)<br>High dose:7/50 (females) – 3/50 (males)<br><br>*Alveolar squamous metaplasia*:<br>Control: 0/50 (females) – 0/49 (males)<br>Low dose: 0/48 (females) – 0/50 (males)<br>High dose: 8/50 (females) – 2/50 (males)<br><br>Overall conclusion:<br>Clear evidence for carcinogenic activity in female rats<br>Some evidence for carcinogenic activity in male rats.	
Boorman and Seely (1995) [68]	Talc (non-asbestiform).<br>CAS NO 14807-6.	Female Fisher 344/F rats (n=10/group).	Tumours of ovaries or other female reproductive organs: no talc-related ovarian lessions were detected in female rats.	No

Reference	Test material	Animal model	Results	Perineal route of exposure (Yes/No)
(data based on NTP Study (1993) [67])	High purity, microtalc, MP (10-52 Grade), max particle size is 10 µm and contained no tremolite or asbestiforms, free of silica - 1 particle of silica was detected in 1,466 particles examined; obtained from Walsh and Associates, USA.	Concentrations: 0, 6 and 18 mg/m^3.<br><br>Control group: Female Fisher 344/F rats<br>(n=10/group).<br><br>Duration of exposure:<br>rats-lifetime.	Female rats (incidence rate of ovarian lessions):<br><br>*Ovarian cyst*:<br>Control: 5<br>Low dose: 0<br>High dose: 1<br><br>*Granulosa cell tumor:*<br>Control: 1<br>Low dose: 0<br>High dose: 0<br><br>*Granulosa cell tumor, benign*:<br>Control: 0<br>Low dose: 2<br>High dose: 0<br><br>*Granulosa theca tumor, benign*:<br>Control: 0<br>Low dose: 1<br>High dose: 0	

Reference	Test material	Animal model	Results	Perineal route of exposure (Yes/No)
			Granulosa theca tumor, malignant:<br>Control: 0<br>Low dose: 0<br>High dose: 1<br><br>Inhalation exposure route (whole body exposure) suggests that exposure was occurred through dermal and oral routes of exposure.	
National Toxicology Program (1993) [67]	High purity, microtalc,<br>MP (10-52 Grade), max particle size 10 µm and contained no tremolite or asbestiforms, free of silica - 1 particle of silica was detected in 1,466 particles examined; obtained from Walsh and	Male and female B6C3F1 mice (n=47-49/group).<br><br>Exposure route: inhalation.<br><br>Dose: 0, 6, 18 mg/m^3 for 6h/5days per week.<br><br>Duration of exposure: 104 weeks.	Tumours of ovaries or other reproductive organs: not found.<br><br>No significant difference was observed in the incidence of neoplasms compared to controls.<br><br><u>Non-neoplastic effects in lungs</u><br>Females<br><br><u>Lung</u><br>*Chronic inflammation*:<br>Control: 0/46 (females) – 0/45 (males)	No

Reference	Test material	Animal model	Results	Perineal route of exposure (Yes/No)
	Associates, USA.		Low dose: 25/48 (females) – 16/47 (males)<br>High dose:38/50 (females) – 40/48 (males).<br><br>*Macrophages hyperplasia:*<br>Control: 2/46 (females) – 3/45 (males)<br>Low dose: 45/48 (females) – 46/47 (males)<br>High dose:43/50 (females) – 43/48 (males).<br><br>"No evidence of carcinogenic activity" was observed in mice but male and female mice might tolerate higher doses compared to male and female rats (National Toxicology Program, 1993).	
Boorman and Seely (1995) [68]<br>*(data based on NTP Study (1993) [67])*	High purity, microtalc,<br>MP (10-52 Grade), max particle size is 10 µm and contained no tremolite or asbestiforms,	Female B6C3F1 mice (n=10/group).<br><br>Concentrations: 0, 6 or 18 mg/m^3.	No talc-treatment related ovarian lessions were detected in female mice.<br><br>Female mice (incidence rate of ovarian lessions):<br><br>*Ovarian cyst*:	No

Reference	Test material	Animal model	Results	Perineal route of exposure (Yes/No)
	free of silica - 1 particle of silica was detected in 1,466 particles examined (obtained from Walsh and Associates, USA).	Control group: female B6C3F1 mice (n=10).<br><br>Duration of exposure: 2 years.	Control: 6<br>Low dose: 11<br>High dose: 10<br><br>*Abscess of ovary*:<br>Control: 4<br>Low dose: 10<br>High dose: 7<br><br>*Thrombosis*:<br>Control: 1<br>Low dose: 2<br>High dose: 0<br><br>*Adenoma*:<br>Control: 1<br>Low dose: 1<br>High dose: 0<br><br>*Cystadenoma*:<br>Control: 0	

Reference	Test material	Animal model	Results	Perineal route of exposure (Yes/No)
			Low dose: 1<br>High dose: 0<br><br>*Luteoma*:<br>Control: 2<br>Low dose: 0<br>High dose: 0.<br><br>Inhalation exposure (whole body exposure) suggests that exposure occurred through dermal and oral routes of exposure.	
Pickrell et al. (1989) [69]	Asbestos- free talc, respirable talc (2.7 - 3.3 µm), contained 19.2-19.4% of magnesium (Mg)	Male and female F344/Crl rats (n=10/sex/group).<br><br>Male nd female B6C3F1 mice<br>(n=10/sex/group).<br><br>Exposure route: inhalation.	No exposure-related abnormalities were observed in lungs, tracheobronchial lymph nodes, sections of turbinates, trachea and larynx at necropsy of the animals.	No

Reference	Test material	Animal model	Results	Perineal route of exposure (Yes/No)
		Concentrations:<br>Rats: 0, 2.3, 4.3, 17 mg talc/m^3;<br><br>Mice: 0, 2.2, 5.7, 20.4 mg talc/m^3<br><br>Control group:<br>Animals exposed to filtered air.<br><br>Duration of exposure:<br>6 h per day/5 days per week/ 4 weeks (20 days).		
Wagner et al. (1977) [70]	Italian talc, 00000 grade; talc mean particle size 25 µm, maximum particle size 70 µm, asbestos fibers free; 92% talc (by wt), 3%	Male and females barrier protected Wistar rats (n=24/group)<br><br>Exposure route: inhalation.	Tumours of ovaries or other female reproductive organs : no talc treatment-related macro- and microscopic abnormalities were found in the ovaries, uterus, Fallopian tubes.<br><br><u>Lung</u><br>*6 months*	No

Reference	Test material	Animal model	Results	Perineal route of exposure (Yes/No)
	chlorite, 1% carbonate minerals; 0.5-1% quartz.	Dose: 10.8 mg/m^3, 7.5 h./5 days/week.<br><br>Control group (negative): ambient air.<br><br>Control group (positive): superfine asbestos (CFA).<br><br>Duration of exposure: 3, 6, or 12 months, life-span duration.	Talc or control group: no lung tumors (adenoma, adenomatosis, or adenocarcinoma) observed;<br><br>Chrysotile group: 1 adenomatosis was found.<br><br>*12 months*<br><br>Talc group: 1 adenoma was observed<br><br>Chrysotile group: 3 adenomas, 2 adenomatosis, and 1 adenocarcinoma were found.<br><br>No lung tumors were found in the controls.	
Wehner et al. (1977) [71]	Talc: commercial (talc) Johnson's baby powder (high grade cosmetic talc); lot 228p; 95% (w/w) platy talc with trace quantities of carbonates (magnesium	Male and females Syrian golden E1a: ENG strain hamster (n=25-50 sex/group).<br><br>Exposure route: inhalation.<br><br>Dose:	Tumours of ovaries or other female reproductive organs: No talc treatment-related macro- and microscopic abnormalities were found in the ovaries (one from each animal).<br><br>1 female had uterine leiomyoma (high exposure group (150 min/day for 300 days).<br><br>No uterine leiomyomas were found in the controls.	No

Reference	Test material	Animal model	Results	Perineal route of exposure (Yes/No)
	and dolomite) and platy chlorite and rutile.	Mean total aerosol concentration:<br>30 days: 37.1±7.4 µg/litre with a respirable fraction of 9.8±2.4 µg/litre, mass median aerodynamic diameter 4.9 µm.<br><br>300 days: 27.4±3.4 µg/litre with a respirable fraction of 8.1±0.1 µg/litre, mass median aerodynamic diameter 6.0 µm.<br><br>Control group: exposed to filtered room air.<br><br>Duration of exposure:<br>30 days: 3, 30, 150 minutes (2.5 h.) per day, 5 days/wk.	No primary tumors were observed in the lungs in any of the hamsters.<br><br>No differences in the type, incidence, and severity of lesions were observed between exposed and control groups<br><br>A marked sex-related difference in the mean survival time in all groups – males lived longer than females ($p<0.05$).<br><br>No treatment-related histopathological changes were observed in lungs, trachea, larynx, liver, one kidney, stomach, one testis, and all tissues showing gross lesions.	

Reference	Test material	Animal model	Results	Perineal route of exposure (Yes/No)
		300 days: 30, 150 minutes (2.5 h.) per day, 5 days/wk.<br><br>Animals were killed at 20 months.		
Wagner et al. (1977) [70]	Talc: Italian talc, 00000 grade; particle size 25 µm (mean particle size); maximal size, 70 µm; 92% talc (by wt), 3% chlorite, 1% carbonate minerals; 0.5-1% quartz; asbestos free talc.	Male and female barrier protected Wistar rats (n=24/group)<br><br>Exposure route: Intrapleural injection.<br><br>Dose: 20 mg/rat (0.4 ml normal saline).<br><br>Control group (negative): received saline injection.<br><br>Control group (positive):	Tumours of ovaries or other reproductive organs: full necropsy examination did not reveal any treatment – related abnormalities in females' reproductive organs – uterus or ovaries.<br><br>No treatment – related abnormalities were observed in other organs.<br><br>No mesotheliomas were observed in talc or saline treated group.<br><br>Injection-site (local) granulomas were common.<br><br>1 small pulmonary adenoma was found in 1 talc-treated rat.<br><br>No mesotheliomas were observed in saline control group.	No

Reference	Test material	Animal model	Results	Perineal route of exposure (Yes/No)
		Superfine asbestos (CFA).<br><br>Duration of exposure: Life-span duration (up to 655-691 days).	Chrysotile group: 18 mesotheliomas were found.<br><br>Woywodt et al.(2000) [72] suggested that talc granulomatosis causeshypercalcemia due to increased extra-renal 1-alpha-hydroxylation of 25-hydroxyvitamin D after talc exposure.	
Stenbäck & Rowlands (1978) [73]	Talc: USP (Fisher Scientific, Co., USA); silicon dioxide 61-63%, magnesium oxide 32-34%, other dusts – 0.85-1.06%, pH 7.7±0.3, specific gravity 2.7.	Male and female Syrian golden hamster (n=24/group).<br><br>Exposure route: Intratracheal exposure.<br><br>Group I (Talc): 3 mg of talc in 0.2 ml saline.<br><br>Group 2 (Talc+B(a)P): 3 mg of talc and 3 mg	Tumours of ovaries or other reproductive organs: No treatment-related abnormalities were reported in reproductive organs following full necropsy examination of all animals.<br><br>No respiratory tract tumors were observed in the talc-treated, saline-treated or untreated groups.<br><br>No detectable dust deposits, granuloma formation or mesothelial proliferations in the respiratory system were found.<br><br>A lymphoma, a melanoma and an adrenal cortex adenoma were observed in talc –	No

Reference	Test material	Animal model	Results	Perineal route of exposure (Yes/No)
		B(a)P in 0.2 ml saline.	treated animals but no relationship with talc treatment was found.	
		Control groups 3 and 4: received saline or were untreated.		
		Duration of exposure: 18 instillations, once weekly.		
Bischoff and Bryson (1976) [74]	Talc type: USP talc (no other details available).	Male Marsh mice (number per group - not reported).	1 dermal desmoplasia with a low-grade fibrosarcoma at the talc leackage site was observed in 1 male.	No
		Exposure route: intrathoracic injection.	5/47 talc-injected mice had tumours (two adenocarcinomas and three lymphoid tumours of the lung) compared with none of 48 saline-injected controls.	
		Dose: 10 mg per animal, single injection.	Foreign body granulomas were observed.	

Reference	Test material	Animal model	Results	Perineal route of exposure (Yes/No)
		Control group: received saline injection.<br><br>Duration of observation: 18-21 months.		
Bischoff and Bryson (1976)<br><br>[74]	Talc type: USP talc (no other details available).	Female Evans rats (n=26-32/group).<br><br>Exposure route: intrathoracic injection.<br><br>Dose: 50 mg/rat, single injection.<br><br>Control group: received saline injection or untreated animals.<br><br>Duration of observation: 18-21 months.	Intrathoracic reticulum cell sarcomas or lymphomas were observed in 7/30 talc-treated rats versus in 8/32 saline-treated rats and 7/28 in untreated controls.<br><br>Foreign body granulomas were observed.	No

Reference	Test material	Animal model	Results	Perineal route of exposure (Yes/No)
Bischoff and Bryson (1976) [74]	Talc USP grade (no other details available)	Female Marsh mice (n=22-28/group).<br><br>Exposure route: single intraperitoneal injection.<br><br>Dose: 20 mg in saline.<br><br>Control group: received saline injection.<br><br>Duration of observation: 18-21 months.	Intraperitoneal lymphoid tumors were observed in 5/22 talc-injected animals and in 6/28 saline injected controls.<br><br>Foreign body granulomas were observed.	No
Bischoff and Bryson (1976) [74]	Talc USP grade (no other details available)	Female Evans rat (n=26-27 /group).<br><br>Exposure route: intraperitoneal injection.	3/27 talc-treated rats developed tumours (one lymphosarcoma and one reticulum-cell sarcoma in the peritoneal cavity, one cystadenoma of the liver) compared with 0 in 26 saline-treated controls.	No

Reference	Test material	Animal model	Results	Perineal route of exposure (Yes/No)
		Dose: 100 mg, single injection.<br><br>Control group: received saline injection.<br><br>Duration of observation: 18-21 months.		
Jagatic et al. (1967) [75]	Talc: USP V grade 7023P-9108, lotB 1842	Male White mice (n=4-12/group).<br><br>Exposure route: Single intraperitoneal injection.<br><br>Dose: 0.5 mL suspension of talc (50%) in normal saline.<br><br>Control group: Talc group was used as a control group vs	No mesotheliomas or other neoplasms were found at histopathological examination.<br><br>Foreign body granulomas (F.B.G.) were noted at all days of post-exposure:<br><br>at 26 day – 12 F.B.G.<br>at 57 day - 4 F.B.G.<br>at 112 day-5 F.B.G.<br>at 147 day – 6 F.B.G.<br>at 170 day – 5 F.B.G.<br>at 343 day – 12 F.B.G.	No

Reference	Test material	Animal model	Results	Perineal route of exposure (Yes/No)
		asbestos exposed group.	F.B.G. was represented by fibrous tissue with necrotic calcified center.	
		Animals were killed at 26, 57, 112, 147, 170 and 343 days after injection.		
Özesmi et al. (1985) [76]	Commercial talc (unspecified)	Swiss albino mice (n=24-46/group).	No macro- or microscopic findings in ovaries or other reproductive organs were reported.	No
		Exposure route: intraperitoneal injection.	Talc-treated group: 3 mesotheliomas (in 12.5% of animals)	
		Dose: 20 mg. talc (sterilised) in 1 ml of saline as a control group for Karain dust (erionite).	Control group: 3 mesotheliomas and 1 lymphoma (in 8.7% of animals). No tumors occurred before 9 months of post-injection.	
		Control group: received injection of physiological saline.	No treatment-related tumors were observed at the injection site, parietal and visceral peritoneal mesothelium, peritoneal lymph nodes, spleen, liver,	

Reference	Test material	Animal model	Results	Perineal route of exposure (Yes/No)
		Duration of observation: life-time.	kidneys, adrenals, mediastinal lymph nodes.	
Pott et al. (1976, 1974) [77-79]	Talc USP grade	Wistar female rat (n=40-80/group).<br><br>Exposure route: intraperitoneal injection.<br><br>Dose: 25 mg. in 2 ml of saline, 4 times at weekly intervals.<br><br>Control group: received saline injection.<br><br>Duration of observation: until spontaneous death.	No macro- or microscopic findings in ovaries or other reproductive organs were reported.<br><br>Mesothelioma was observed in 1/36 talc-exposed rats with tumor rate 2.8% (first tumor at 587 days) compared to 0/72 in saline exposed rats, respectively.	No
Styles and Wilson (1973) [80]	Talc particle size 0.2-20 µm, no other details provided.	Male and female Specific pathogen free (SPF) albino Wistar rats (n=6/sex/dose).	No macroscopic findings on ovaries or other reproductive organs were reported.	No

Reference	Test material	Animal model	Results	Perineal route of exposure (Yes/No)
		Exposure route: intraperitoneal injection.	A granulomatous reaction (granulomas) was evident by 1 month with foreign-body giant cells containing refractive materials.	
		Dose: 50 mg/kg bw (10 mg/mL).	At 3 months, the lesions were still visible but fibrosis was not observed.	
		Control group: untreated animals.	Histopathological examination of omentum, spleen, liver and pancreas did not reveal abnormal findings.	
			Styles and Wilson (1973) concluded that the fibrogenicity of a talc is related to its cytotoxicity to macrophages.	
Bischoff and Bryson (1976) [74]	Talc USP grade (no other details available)	Female Marsh mice (n=24-26/group).	No tumors were observed at the injection site.	No
		Exposure route: single subcutaneous injection in saline.	Foreign body granulomas were observed.	
		Dose: 20 mg per animal.		

Reference	Test material	Animal model	Results	Perineal route of exposure (Yes/No)
		Control group: received saline injection.<br><br>Duration of observation: 18-21 months.		
Neukomm and de Trey (1961) [81]<br><br>*(paper in French)*	Talc - unspecified.	Female R3 mice (n=50 /group).<br><br>Exposure route: single subcutaneous injection.<br><br>Dose: 5 mg of talc (in 0.2 mL of peanut oil).<br><br>Control group: untreated animals.<br><br>Duration of observation: by the	Tumours of ovaries or other reproductive organs: No ovarian tumors were reported.<br><br>No dermal tumors at the site of injection were observed.<br><br>Latency period for the development of ovarian cysts decreased compared to the controls (416 versus 469 days, respectively) ($p>0.05$).<br><br>Latency period and the incidence rate of mammary tumors increased in talc-treated animals compared to control animals.	No

Reference	Test material	Animal model	Results	Perineal route of exposure (Yes/No)
		end of life (596 days).		
Gibel et al. (1976) [82]<br><br>*(paper in German)*	Cosmetic and pharmaceutical grade talc	Male and female Wistar rat (n=25 sex/group).<br><br>Exposure route: oral (diet).<br><br>Dose: diet with 53% chrysotile asbestos.<br><br>Control group I: diet with talc<br><br>Control group II: received diet only.<br><br>Duration of exposure: Life-span duration (up to 702 days).	Tumours of ovaries or other reproductive organs: no treatment-related abnormalities were reported in uterus or ovaries.<br><br>Talc (control group I)<br><br>Survival time was shorter compared to the controls (549 vs 702 days).<br><br>3 malignant liver carcinomas (2 in controls) and 4 benign tumors (5 in controls) were observed.<br><br>Asbestos group<br><br>Survival time was shorter compared to both control and talc groups (441 vs 549 or 702 days, respectively).<br><br>12 malignant tumors (1 lung sarcoma, 4 neuro-sarcoma, 3 reticulum cells sarcoma, 4 liver sarcomas) were observed.	No

Reference	Test material	Animal model	Results	Perineal route of exposure (Yes/No)
Wagner et al. (1977) [70]	Talc type: Italian talc, 00000 grade; talc mean particle size 25 µm, maximal size 70 µm, 92% talc (by wt), 3% chlorite, 1% carbonate minerals; 0.5-1% quartz, asbestos free.	Male and female Wistar rat (n=16/sex/group).<br><br>Exposure route: oral/diet.<br><br>Dose: 100 mg/rat (100 mg/day in feed).<br><br>Control group (negative): diet without talc.<br><br>Control group (positive): diet with super-fine chrysotile asbestos (SFA).<br><br>Duration of exposure: 101 days (5 months) following exposure to basal diet to the	Talc group<br>Mean survival (from start of feeding) - 614 days;<br><br>1 leiomyosarcoma of the stomach, 2 sarcomas of the uterus.<br><br>Controls (negative) group<br>Mean survival - 641 days.<br>1 adrenal adenoma.<br><br>Chrysotile group<br>Mean survival - 619 days.<br>1 possible leiomyosarcoma of the stomach, 1 sarcoma of the uterus, 1 lymphosarcoma.<br><br>Talc group<br>Full necropsy examination of liver, stomach, spleen, ileum, caecum, rectum, omentum, mesentery and parietal peritoneum, any other site of visible pathology did not reveal talc treatment-related abnormalities.	No

Reference	Test material	Animal model	Results	Perineal route of exposure (Yes/No)
		rest of their lifespan.	Wagner et al. (1977) reported that 2 sarcomas of the uterus in the talc group and 1 sarcoma of the uterus in the positive (SFA) group were not “considered as consequences of the feeding because of their location”. The authors stated that 3 sarcomas of the uterus were observed in the earlier study in 126 control rats.	
Hamilton et al. (1984) [83]	Italian talc (0.3-14 μm), asbestos-free	Female Sprague-Dawley rat (n=10/group).	Tumors of ovaries or other reproductive organs: No evidence of neoplasms, cellular or nuclear atypia, mitotic activity in the surface epithelium was found.	No
		Exposure route: Intrabursal injection (talc suspension in phosphate-buffered saline was injected into the surface of ovary).	1 to 12 months<br>Ovaries were macroscopically cystic in appearance; histologically, ovarian cysts were derived from bursal sac and not from the ovaries.	
		Concentration: 100 mg/mL in the sterilized phosphate buffered saline.	No gross changes were observed in controls.<br>Small focal areas of papillary change not consistent with pre-neoplastic processwas observed in the surface	

Reference	Test material	Animal model	Results	Perineal route of exposure (Yes/No)
		Control group: Sham-operated rats.<br><br>Duration of observation: 1,3,6,12 and 18 months.<br><br>Histological examination of ovaries was conducted at 12 months after injection.	epithelium of 4/10 treated animals compared to 0/6 controls.<br><br>Foreign body granulomas (without surrounding inflammation) were observed in 5 of talc injected ovaries.<br><br>No correlation between the presence of intra-ovarian granulomas and the presence of papillary changes were found.	

X.2. Animal studies on talc (published after 2006)

References	Test material	Study design	Results	Perineal route of exposure (Yes/No)
Celik et al. (2009) [84]	Asbestos-free and sterilized talc (no other details available)	New Zealand white rabbits (n=7-10/group).<br><br>Exposure route: Single intrapleural injection into right hemothorax.<br><br>Doses:<br>Talc group: 200 mg/kg (in 2 ml saline).<br>Polidocanol group: 15 mg/kg of 3% polidocanol (in 2 ml saline).	Exposure to talc caused:<br><br>↑[9]pleural inflammation;<br><br>↑ pleural cellularity;<br><br>↑ neovascularity;<br><br>↑ mesothelial cell proliferation compared to controls.<br><br>Talc granuloma was observed in the visceral pleura.	No

[9] ↑ ↓ - reported statistically significant changes in the effect compared to the control group.

References	Test material	Study design	Results	Perineal route of exposure (Yes/No)
		Ethanol group: 1 mL (4.2%) (in 2 mL saline).<br><br>Control group (negative): not established.<br><br>Animals were killed 4 weeks after injection.		
Genofre et al. (2005) [85]	Talc: Sterilized talc slurry.	New Zealand white rabbits (n=9/group).<br><br>Exposure route: intrapleural injection.<br><br>Dose: Single injection of talc:<br>400 mg/kg (in 2 ml saline, pH 8.4)<br><br>Control group: 2 mL of saline.	In talc – exposed group:<br><br>Disruption of the mesothelial cell's basement membrane,<br>cells migration and proliferation with various amount of mononuclear inflammatory cells was observed.<br><br>Talc-treated mesothelial cells released enzymes, cytokines and growth factors<br>proliferation of myoblasts was observed.	No

References	Test material	Study design	Results	Perineal route of exposure (Yes/No)
		Animals were sacrificed 15, min, 24 h., or 7 days after last injection.		
Genofre et al. (2009) [86]	Sterilized talc slurry.	New Zealand white rabbits (n=10/group).<br><br>Exposure route: intrapleural injection.<br><br>Dose:<br>Single injection of talc.<br><br>Group I (MP/mixed partilcles): 400 mg/kg (3 mL) in 2 ml saline (mean size): 25.4 µm).<br><br>Group II SP/ (small particles): 400	Systemic inflammatory response was observed in blood (serum) and pleura (not reported in table).<br><br>In both groups:<br>↑ leucocytes at 6,24 hr but returned to normal levels at 48 hr. (SP and MP);<br><br>↑ neutrophils at 6 and 24 hr in both groups and remined elevated after 48 hr in Group II (SP);<br><br>↑ serum LDH (lactate dehydrogenase) at 6 and 24 hr and reminded elevated after 48 hr in Group II (SP) only;<br><br>↑serum IL-8 (Interleukin-8) levels in both groups during entire observation period.	-

References	Test material	Study design	Results	Perineal route of exposure (Yes/No)
		mg/kg in 2 ml saline (mean size): 4.2 µ).<br><br>Control group: 2 mL of saline.<br><br>Animals were sacrificed 6, 24 or 48 hr post-injection.<br><br>Blood and pleural fluid were assessed to leucocytes, neutrophils, lactate dehydrogenase (LDH), Interleukin-8 (IL-8), vascular endothelial growth factor (VEGF), and transforming growth factor – beta (TGF-beta) content.	↑ serum VEGF (vascular endothelial growth factor) levels during entire period of observation.<br><br>↑ serum TGF-8 (transforming growth factor – beta) levels were elevated in Group I at 6,24 and 48 hr. and at 48 hr. in Group II.<br><br>No acute pulmonary lessons were observed .<br><br>Smaller particles induced more pronounced inflammatory response.	
Iwasaki et al. (2016) [87]	Steritalc (Novatech, France)	Male BALB/c (nude mice) (n=10/group).	↑ pleural thickening.<br><br>Talc did not affect vascular architecture of lung tissue.	No

References	Test material	Study design	Results	Perineal route of exposure (Yes/No)
		Exposure route: Single, intrapleural injection.<br><br>Mice were injected with the human lung adenocarcinoma (cell line PC14PE6). Two weeks after injection, mice were injected with talc and sacrificed 6 weeks after talc injection.<br><br>Dose: 400 mg of talc in 0.3 mL normal saline, particle size 24.5 µm.<br><br>Control group: animals were injected with 0.3 mL of normal saline.		

References	Test material	Study design	Results	Perineal route of exposure (Yes/No)
		Duration of exposure: 6 weeks.		
Marchi et al. (2006) [88]	Talc used in clinical practice (Magnesita, Brasil), particle length (mean) 24.5 µm, range 6.4-50.5 µm).	New Zealand white rabbits (n=10/group).<br><br>Exposure route: Single, intrapleural injection.<br><br>Dose: 200 mg/kg in 3 mL of endotoxin-free saline.<br><br>Control group: treated with 3 mL of endotoxin-free saline.<br><br>Duration of exposure: Pleural fluid collected at 6, 24 or 48 hr. of post-exposure.	↑ IL-8 (Interleukin) concentration in pleural fluid at 6 hr.<br><br>↑ VEGF (vascular endothelial growth factor) and<br><br>↑TGF-β1 (transforming growth factor) levels in pleural fluid steadily over 48 h.<br><br>↑inflammatory pleural reactions.	No

References	Test material	Study design	Results	Perineal route of exposure (Yes/No)
Miller et al. (2007) [89]	Talc was obtained from Sigma Chemical Co., USA.	New Zealand white rabbits (n=6-7/group).<br><br>Exposure route: Single, intrapleural injection.<br><br>Dose: 70 mg/kg (in 2 mL of saline).<br><br>Control group: animals treated with 2 mL of saline.<br><br>Post-exposure examination: 8, 16, or 30 days.	↑ fibrosis rate on 29% (2/7) and ↑ inflammation in 14% (1/7) of talc-treated animals.<br><br>↑ granulomatous depositions in 57% of treated animals (in the pleura, lung, diaphragm, and other surrounding tissues).<br><br>Note: 70 mg/kg is analogous dose that is used for human pleurodesis.	No
Rossi et al. (2010) [90]	Small particle talc (Signa Aldrich, Germany) and mixed particle size (Sao Paulo, Brazil), small particles	New Zealand white rabbits (n=5/group).<br><br>Exposure route: Intrapleural injection.	Blood and bronchoalveolar lavage (BAL):<br><br>No changes in the leukocyte count.	No

References	Test material	Study design	Results	Perineal route of exposure (Yes/No)
	size (median) – 6.4 μm, mixed particles size (median) - 21.15 μm.	Dose: 400 mg/kg in saline.<br><br>Group ST (small particles): animals were injected with talc with small particle size.<br><br>Group MT (mixed particles): animals were injected with particles of mixed sizes.<br><br>Control group: animals treated with saline.<br><br>Bronchoalveolar fluid and blood were collected at 6, 24, 72 or 96 hr. of post-exposure.	No changes in LDH (lactate dehydrogenase) measurements in talc-treated and control animals.<br><br>↑ serum levels of CRP (C-reactive protein) and IL-8 (interleukin) in the animals from group ST.<br><br>↑ serum levels of VEGF (vascular endothelial growth factor) after 24 h and remained high throughout the entire observation period in both groups.<br><br>ST induced a more pronounced inflammatory response based on CRP and IL-8 levels in blood and BAL.	
Puel et al. (2007) [91]	Talc was purchased	Wistar female rats (n=13/group).	Sh+T and OVX+T groups versus Sh and OVX groups:	

References	Test material	Study design	Results	Perineal route of exposure (Yes/No)
	from ICN Biomedicals, France, other details not provided.	Exposure route: Repeated subcutaneous injections.<br><br>I.Sh group (sham operated group received standard diet).<br><br>2.SH+T group (sham operated group with subcutaneous (s.c.) injection of talc).<br><br>3.OVX group (rats with surgical ovariectomy).<br><br>4. OVX+T group (rats with surgical ovariectomy (OVX) with s.c. injection of talc).	↑fibrinogen and a1-acid glycoprotein levels in blood (plasma protein synthesis).<br><br>↑ levels of granulocytes in blood.<br><br>↑ spleen (hypertrophy);<br><br>↑ the urinary excretion of isoprostane (a marker of oxidative stress).<br><br>↓ the total antioxidant capacity of the plasma (as indicated by ferric reducing ability of plasma values).<br><br>Puel et al. (2007) concluded that in OVX rats, a low grade granulomatosis inflammation caused by talc was characterized by a rise in inflammatory parameters such as fibrinogen, a1-acid glycoprotein, spleen weight and granulocyte level, and an impairment of oxidative status (a decrease in plasma	

References	Test material	Study design	Results	Perineal route of exposure (Yes/No)
		Dose: 4 subcutaneous injections of sterile talc (1 g. in 2 ml of saline, as described in Krempien et al. (1988)i paper). Duration of exposure: 21 days (from 61 to 84 days of study).	antioxidant capacity, a higher rate of isoprostane excretion)[10].	
Orsal et al. (2013), [92]	Talc was purchased from Sigma (Sigma Aldrich), other details not provided.	Wistar female rats (n=8/group). Exposure route: subcutaneous injection.	Repeated subcutaneous exposure to talc: ↑ release of proinflammatory cytokines IL-1, IL-6 and TNF-a was observed in both sham-operated rats and rats with ovariectomy.	No

[10] Prostanoids produced by peroxidation of arachidonic acid and quantification of F2-isoprostanes (F2-isoP) in plasma and urine are sensitive and specific indices of lipid peroxidation in vivo (in Hill DB, Awad JA. Increased urinary F2-isoprostane excretion in alcoholic liver disease. Free Radic Biol Med. 1999 Mar;26(5-6):656-60).

References	Test material	Study design	Results	Perineal route of exposure (Yes/No)
		1.Sh group (Sham operated group received standard diet).<br><br>2.SH+T group (Sham operated group with subcutaneous (s.c.) injection of talc).<br><br>3.OVX group (rats with surgical ovariectomy).<br><br>4. OVX+T group (rats with surgical ovariectomy (OVX) with s.c. injection of talc).<br><br>Dose: 4 subcutaneous injections of sterile	Orsal et al. (2013) stated that "estrogen has the ability to suppress the production of proinflammatory cytokines, and postmenopausal estrogen withdrawal associated with aging leads to inducing an unregulated chronic inflammatory process by increasing the local production of various cytokines.	

References	Test material	Study design	Results	Perineal route of exposure (Yes/No)
		talc (1 g. in 2 ml of saline).<br><br>Duration of exposure: 21 days (from 61 to 84 days of study).		
Polat et al. (2013)[93]	Talc	Wistar female rats (n=8/group).<br><br>Exposure route: subcutaneous injection.<br><br>1.Sh group (Sham operated group received standard diet).<br><br>2.SH+T group (Sham operated group with subcutaneous (s.c.) injections of talc).	↑release of pro-inflammatory cytokines interleukin (IL)-1beta, interleukin-6, and tumor necrosis factor (TNF) – alpha in both sham- and ovariectomized rats.<br><br>↑ inflammatory markers in OVX+T rats when compared to OVX and sham-operated animals.	No

References	Test material	Study design	Results	Perineal route of exposure (Yes/No)
		3.OVX group (ovariectomized group: Rats with surgical ovariectomy).<br><br>4. OVX+T group (rats with surgical ovariectomy with s.c. injections of talc).<br><br>Dose:<br>4 subcutaneous injections of sterile talc (3.2 g. in total per animal, in 2 ml of saline).<br><br>Duration of exposure: 21 days (from 59 to 80 days of study).		

References	Test material	Study design	Results	Perineal route of exposure (Yes/No)
Teixeira et al. (2011)[94]	Talc USP11 Pharmacy, Brazil)	New Zealand white rabbits (n=5/group).<br><br>Exposure route: intrapleural injection.<br><br>Dose: 400 mg/kg (2 mL) (no other details provided).<br><br>Control group: no data.<br><br>Duration of exposure: 7, 14, 28 days of post-exposure.	↑ pleural adhesion in a time dependent manner and vascular density at 7 and 14 days after injection.	No

[11] The following are the acceptance criteria for USP-grade talc: 17.0-19.5% magnesium; not more than 0.1% water-soluble substances with neutral pH; no more than 0.25% iron; not more than 10 ppm lead; not more than 0.9% calcium; not more than 2.0% aluminum; and a demonstration of an absence of asbestos.

References	Test material	Study design	Results	Perineal route of exposure (Yes/No)
Keskin et al. (2009) [95]	Talc: Test material is not described.	Female Sprague-Dawley rats (n=7/group).<br><br>Dose: Intravaginal administration of talc 100 mg (in 0.5 mL of saline).<br><br>Control group: recieved saline.<br><br>Duration of exposure:<br>Daily application for 3 months.	No pre-neoplastic or neoplastic changes in reproductive organs were observed.<br><br>↑ number of follicles in animals exposed to talc.<br><br>Talc group:<br>Vulvovaginitis was observed in 5 rats<br>Endometritis - in 6 rats<br>pelvic infection - in 4 rats<br>ovary infections - in 3 rats /7 ovaries.<br><br>Control group :<br>Endometritis was observed in 2 rat.	Yes
Keskin et al. (2009) [95]	Talc: Test material is not described	Female Sprague-Dawley rats (n=7/group).<br><br>Exposure route: Perineal administration of talc.	No pre-neoplastic or neoplastic changes in reproductive organs were observed.<br><br>↑ number of follicles in animals exposed to talc.<br><br>Vulvovaginitis was observed in 7 rats<br>endometritis in 4 rats	Yes

References	Test material	Study design	Results	Perineal route of exposure (Yes/No)
		Dose: 100 mg (in aerosol form).<br><br>Control group:<br><br>Untreated animals.<br><br>Duration of exposure:<br><br>Daily application for 3 months.	pelvic infection in 5 rats<br><br>ovarian infection in 4 rats (8 ovaries salpingitis)<br><br>tubal inclusion in 2 rats.<br><br>Control, untreated animals:<br><br>Vulvovaginitis and<br><br>endometritis with infection in both ovaries were observed in 2 rats and 1 rat developed salpingitis.	
Yumrutas et al. (2015) [96]	Talc as “talcum powder”, no other details provided.	Femal Wistar albino rats (n=7/group).<br><br>Exposure route: Surgical application of talc /insertion into intrauterine horn.<br><br>Dose: 100 mg/kg was inserted into right uterine horn of sexually mature rats.	> mRNA expression levels of antioxidant genes - Gsr and Sod1 in ovarian tissue ($p<0.05$) of treated animals.<br><br>Yumrutas et al. (2015) suggested that “cells respond … by invoking their antioxidant defence mechanisms. Sod1 and Gsr are a part of these defence mechanisms activated in response to free oxygen radicals “and proposed that “… antioxidant defense mechanisms are activated in response to the toxic effects of talcum powder”.	No

References	Test material	Study design	Results	Perineal route of exposure (Yes/No)
		Control group: No treatment.<br><br>Duration of exposure: 30 days.		

X.3. In-Vitro studies on talc

References	Test material	Study design	Results
Akhtar et al. (2010) [97]	Indigenous nanotalc:<br>80-130 nm.<br><br>Commercial nanotalc:<br>70-120 nm.<br><br>Indigenous microtalc:<br>50-65 μm.	Test system: Human lung adenocarcinoma (A549) cell line.<br><br>Dose: 50, 100 or 200 μg/mL, single treatment.<br><br>Control group: cells without talc treatment.<br><br>Duration of exposure: 48 h.	Microparticles contained 2.3 times more iron compared to nanoparticles.<br><br>↓ cell viability (all particles).<br><br>Cytotoxicity of microparticles was 3 times less than nanoparticles (IC 50 600-630 μg/mL vs IC50 190-325 μg/mL, respectively).<br><br>↑ LDH activity in a concentration-dependent manner.<br><br>↑ reactive oxygen species (ROS) generation in a concentration dependent manner.<br><br>↓ GSH levels in a concentration dependent manner.<br><br>↑ MDA levels in a concentration dependent manner.

References	Test material	Study design	Results
Akhtar et al. (2014) [98]	Indigenous nanotalc (average size) 94 nm, iron-0.19%;<br><br>Commercial nanotalc (average size):<br>91 nm, iron - 0.08%;<br>and respective iron-chelated particles.	Test system: Human lung adenocarcinoma (A549) cell line.<br><br>Dose: 200 µg/mL, single treatment.<br><br>Control group: cells without talc treatment.<br><br>Duration of exposure: 48 h.	The tendency of both particles to form aggregates was observed.<br><br>Both particles contained iron (0.19% and 0.08%, respectively).<br><br>Both types of particles:<br>< cells viability.<br><br>↑ activity of lactate dehydrogenase) (LDH).<br>↑ cytotoxicity of iron chelated nanoparticles compared to nanoparticles.<br><br>Caused alteration in cell cycle phases.<br><br>↑ reactive oxygen species (ROS).<br><br>↑ membrane lipid peroxidation (LPO) activity (formation of thiobarbituric acid reactive species (TBARS).

References	Test material	Study design	Results
			↓ activity of antioxidants enzymes (superoxide dismutase (SOD), catalase (CAT) and glutathione (GSH) activity).<br><br>Induced apoptosis (increased caspase-3 enzyme activity).<br><br>caused DNA fragmentation in A549 cells.<br><br>Akhtar et al. (2012) concluded that: “Both particles significantly induced cytotoxicity, oxidative stress, and apoptosis in human lung epithelial cells. Further, chelation of iron from both particles caused significantly less toxicity as compared to non-chelated particles. Therefore, iron content plays a significant role in the toxicity of nanoparticles, which may be mediated through ROS generation and oxidative stress.”
Buz’Zard et al. (2007) [99]	Talc - details on chemical and physical characteristics not provided.	Test system: Normal ovarian epithelial cells (OSE2a) or normal ovarian granulosa (stromal) cells (GS1a).<br><br>Dose: 0, 0.5, 5, 20, 50, 100, 200, 500 µg/mL single treatment.	Cell viability<br><br>OSE2a cells:<br>↑ with 5 µg/ml talc at 24 h<br>↓ at 200 µg/ml after 72 h and at 500 µg/ml after 24 and 72 h.

References	Test material	Study design	Results
		Control group: cells without talc treatment.<br><br>Duration of exposure: from 24 h to 72 h, and 120 h.	**GSC1a cells**:<br>↑ at 5, 20, and 100 µg/ml talc after 72 h<br>↓< at 500 µg/ml after 24 h.<br><br><u>Neoplastic transformation assay</u><br>OSE2a cells:<br>↑ the number of transformed colonies at 5 and 20 µg/ml.<br>↓ in transformed cells at 100 µg/ml.<br><br>**GSC1a cells**:<br>↑ the number of transformed colonies at 5, 20, and 100 µg/ml.<br><br><u>ROS inducing activity</u><br>OSE2a:<br>↓ in ROS generation (at 24 h).<br><br>↑ ROS generation:<br>At 20 µg/ml (72 and 120 h)<br>At 50 µg/ml (120 h.).

References	Test material	Study design	Results
			GSC1 cells<br>↑ROS at 0.5,20 and 50 µg/mL (72 and 120 h), as well as 5 and 100 µg/ mL (120 h) compared with the respective 24 h value.<br><br>PMN (polymorphonuclear neutrophils):<br>↑in ROS induction in a concentration-dependent manner at 0.5, 5, 20, and 50 µg/ml at 24 h and at 100 and 500 µg/ml at 24 and 72 h.<br><br>Buz'Zard et al. (2007) concluded: "Talc is capable of increasing cell proliferation, inducing neoplastic transformation of both the normal stromal and epithelial ovarian cells in vitro; and increasing ROS generation in these cells as well as the PMN cells. Talc may contribute to ovarian carcinogenesis in humans by way of inducing aberrant ROS generation."
Chamberlain and Brown (1978) [100]	Talc Italian 00000, particle size not provided.	Test system:<br>V79-4 Chinese hamster lung cells;<br>human alveolar Type II lung cells (A549).	V79-4 cells<br>50 µg/ml was not cytotoxic.<br><br>A549 cells<br>↓ the growth of A549 cells, no details on concentrations and inhibition values.

References	Test material	Study design	Results
Davies et al. (1983) [101]	Cosmetic grade talc (5 samples)<br>1 sample with 30-35% chlorite;<br>1 sample with 1-3% amphiboles.	Test model: Unstimulated mouse peritoneal macrophages.<br>Cosmetic-grade samples: 80-91.5% of the respirable dust (1.94-7.36% of the sample) - <7.5 µm.<br>Micronized cosmetic talc: 93.5% of the respirable dust (19.46% of the sample) - <7.5 µm.	All 7 talc samples were cytotoxic to macrophages compared to controls, but less cytotoxic when compared to the quartz sample.<br>The activity of talc samples was similar and not related to particle-size distribution.<br>Talc exposure caused in peritoneal macrophagoes:<br>↑ release of LDH,<br>↑ release of lysosomal β-glucuronidase was higher compared to LDH.
Ghio et al. (2012) [102]	Talc 10-50 µm, no fibers.	Test system: Mesothelial (Met-5A) cells.<br>Human Bronchial Epithelial Cells (BEAS-2B) cells.<br>Dose: 100 µg/mL, single treatment.<br>Control group: cells exposed to phosphate buffered saline (PBS) without talc treatment.	Mesothelial and airway epithelial cells:<br>↑iron accumulation<br>↑ concentration of the storage protein ferritin.<br>↑ levels of oxidants with a further complexation of surface iron by talc in a time-dependent and concentration-dependent manner.<br>↑ expression of proinflammatory mediators (IL-8).

References	Test material	Study design	Results
		Duration of exposure:<br>Cellular iron homeostasis: 4 h.	Changes in RNA for IL-8 and IL-6.<br><br>Lung tissue from patients treated with pleurodesis:<br><br>↑ iron content and expression of iron-related proteins, including ferritin, the importer divalent metal transport–1 and the exporter ferroportin-1;<br><br>Ghio et al., 2012 concluded: “The exposure to talc disrupts iron homeostasis in mesothelial and airway epithelial cells, and is associated with both oxidative stress and a biological effect, comparable to those of other particles. The resultant accumulation of iron and alterations in iron-related proteins are evident among patients with pleurodesis.”

References	Test material	Study design	Results
Henderson et al. (1975) [103]	Talc Italian 00000, particle size ≤10 μm.	Test system: rabbit lung fibroblasts.<br><br>Dose: 10 μm.<br><br>Exposure duration: 26 h, 8 days.	Talc particles from 0.3 μm up to 10 μm were observed in fibroblasts using electron microscopy and X-ray microanalysis.<br><br>Henderson et al. (1975) suggested that it might lead to the possible change in the morphology of the ovarian tissue surrounding the foreign inclusion. The presence of talc particles in human ovarian tissue could be related to some predisposing factors such as ovarian granulomas. After subcutaneous implantation of talc, granulomatous lesions were observed from 2 months to 36 years [104] .
Lee et al. (2010) [105]	Talc 2 μm.	Test system:<br>Lung adenocarcinoma cell line (LAC, A549).<br><br>Human pleural mesothelial cells (PMC).<br><br>Dose: 25, 50 and 75 μg/ml are<br>added to A549 cell cultures in endotoxin-free normal saline.<br><br>Control group: treated with silicone beads.	Talc caused apoptosis only in LAC cells in a dose- and time-dependent manner.<br><br>Limited description of study design and reported results.

References	Test material	Study design	Results
		Duration of exposure:24, 48 or 72 hr.	
Nasreen et al. (1998) [106]	Talc: in endotoxin-free water, particle size 2.1 µm	Test systems:<br>Pleural mesothelial cells (PMC).<br><br>Dose:<br>Study I. 0, 2-64 µg/cm^3 sterilised talc.<br><br>Study II. 4 µg/cm2 sterilized talc.<br><br>Controls: treated with 4 µg/cm^2 glass microspheres.<br><br>Duration of exposure:<br>24 h (Study I)<br>1-72 h. (Study II).	Study I<br>PMC viability decreased with increasing talc concentrations.<br>PMC viability with 64 µg/cm2 - 75%.<br><br>↑ release of IL-8 and MCP-1 compared to controls (all concentrations).<br><br>↑ chemotactic activity for neutrophils and monocytes compared to controls.<br><br>↑ transcriptional response of IL-8 and MCP-1 expression.<br><br>↑ intercellular adhesion molecule-1 (ICAM-1) expression on PMC.<br><br>Study II<br>↑ production of IL-8 and MCP-1 compared to controls.

References	Test material	Study design	Results
Nasreen et al. (2000) [107]	Talc in endotoxin-free 0.89% normal saline (4.0 mg/ml), particle size 2.1 µm.	Test systems: Pleural mesothelial cells (PMC), malignant mesothelioma cells (MMC).<br><br>Dose:<br><br>Study I. 0, 24 µg/cm^3 sterilised talc in serum – free medium.<br><br>Study II. 0-24 µg/cm2 sterilized talc in serum – free medium.<br><br>Controls I-II: cells were treated with 4 µg/cm^2 glass microspheres.<br><br>Study III. PMC/MMC cells were treated with 4 µg/cm^2.<br><br>Controls III: cells were treated with 6 µg/cm2 glass microspheres.<br><br>Duration of exposure: 72h., 24 h., and 24-72 h., respectively.	Study I<br><br>↓ PMC viability - 93%<br><br>↓ MMC viability with increasing concentration of talc; viability was from 62 to 84% depending on the cell line.<br><br>Study II<br><br>In PMC, significant apoptosis was not observed at any concentration<br><br>Increased apoptosis was observed in MMC in a concentration-dependent manner; no dose response was observed.<br><br>Study III<br><br>In PMC, apoptosis did not increase with time.<br><br>↑ apoptosis in MMC in a time-dependent manner ($p<0.05$).<br><br>Apoptotic DNA changes were observed in MMC only.

References	Test material	Study design	Results
Shukla et al. (2009) [108]	Non-fibrous, sterilized talc (MP 10-52); mean surface area – 16.03 m^2/g., particle size (mean) 1.1 µm.	Study I<br>Test system:<br>Human mesothelial cells (LP9/TERT-1).<br><br>Dose: 15 and 75 µm/cm^2.<br><br>Negative control groups:<br>cells exposed to fine $TiO2$ (15 µm/cm^2) at 8 and 24 hours<br><br>glass beads (75 µm/cm^2) at 24 h.<br><br>Exposure duration: 8 h.	Cell viability: non-toxic up to ≤163 µm2/cm^2; toxicity seen with ≥243 µm/cm^2.<br><br>Inconsistent changes in gene expression at low concentrations during observation period (8-24 h.) and > gene expression of 30 genes at high concentrations (at 8 h).
Shukla et al. (2009) [108]	Talc: non-fibrous, sterilized talc (MP 10-52); mean surface area – 16.03 m^2/g., particle size (mean) 1.1 µm	Study II<br>Test system: human ovarian epithelial cells (IOSE).<br><br>Dose: 75 µm/cm^2.<br><br>Negative control groups:	Cell viability: no toxic effects were noted up to 75 µm/cm^2.<br><br>No significant mRNA changes were observed at any concentration.<br><br>“IOSE cells are occasionally linked to inflammation and the development of ovarian

References	Test material	Study design	Results
		cells exposed to fine TiO_2 (15 μm/cm2) at 8 and 24 h.<br><br>glass beads (75 μm/cm2) at 24 h.<br><br>Exposure duration: 24 h.	cancer after use of talcum powder in the pelvic region, although such links are highly controversial.”
Styles et al. (1973) [80]	Non-fibrous talc, 0.2-20 μm.	Test system: Peritoneal (PM) and alveolar (AM) macrophages.<br><br>Dose: 0.5 mg/mL<br><br>Control group: untreated cells<br><br>Exposure time: 0, 1 and 2 h.	Number of talc particles visible by light microscope in 1 ml of culture medium containing 0.5 mg dust – 7.24-7.31.<br><br>Low cytotoxicity of dust to macrophages in vitro.<br><br>At 2 h of post-exposure (PM):<br>94.4% of cells were viable<br>51.5% of viable cells contained particles vs 88.7% of viable calls in controls<br>8.2% of non-viable cells contained particles.<br><br>At 2 h post-exposure (AM):<br>88 – 87.2% of cells were viable<br>31.8% of viable cells contained particles vs 88.7% of viable calls in controls

References	Test material	Study design	Results
			2.7% of non-viable cells contained particles.<br><br>The phagocytic activity of alveolar macrophages was less compared to peritoneal macrophages.<br><br>The mechanism of fibrogenesis by cytotoxic dusts is not simply due to membrane damage but most of all involves interaction of the dust with cell components such as lysosomes and mitochondria.
Endo-Capron et al. (1993) [109]	3 types of talc samples: Spanish talc (No. 5725), Italian talc (No. 5726)<br>and French talc (No. 7841).<br>Each talc sample contained 90-95% talc and chlorite and dolomite.	Unscheduled DNA synthesis (UDS) genotoxicity assay.<br><br>Test system: rat pleural mesothelial cells (RPMC).<br>Dose: 0, 10, 20 and 50 µg/cm^2 (or 0, 50, 100 and 250 µg/L)<br><br>Duration of exposure: 24 h.	Talc was taken by rat pleural mesothelial cells.<br><br>No increased UDSs was observed in talc-treated RPMC.
		Sister chromatid exchanges (SCEs) genotoxicity assay<br><br>Test system:	Talc particles were taken up by rat pleural mesothelial cells.

References	Test material	Study design	Results
		rat pleural mesothelial cells (RPMC)<br><br>Dose: 0, 2, 5, 10 or 15 μg/cm^2 (or 0, 15, 37.5, 75 or 112.5 μg/L) for 48 hours in the dark.<br><br>Positive controls: (Rhodesian chrysotile and crocidolite asbestos).	No increased SCEs was observed in talc-treated RPMC.<br><br>Chrysotile and crocidolite significantly increased SCEs.
IARC (1987) [110] (unpublished reports of Litton Bionetics, Inc)	Talc: details not available	Test systems:<br>I. Salmonella typhimurium strains TA1530 or HisG46<br><br>II.Saccharomyces cerevisiae<br><br>III. Human fibroblasts	I. No induced mutations were observed.<br><br>II. No induced mutations were observed.<br><br>III. No chromosomal aberrations were observed.

Abbreviations: GC1a = normal ovarian granulosa cells; IL-8 = interleukin-8; IOSE = human ovarian epithelial cells; LAC = lung adenocarcinoma cell line; LDH = lactate dehydrogenase; LP9 = human mesothelial LP9/TERT-1 cells; MCP-1 = monocyte chemotactic protein-1; MMC = human malignant mesothelioma cells; OSE2a = normal ovarian epithelial cells; PMC = human pleural mesothelial cells; PMN = polymorphonuclear neutrophils; ROS = reactive oxidative species.

X.4. Absorption, distribution and elimination of talc by different routes of exposure

Reference	Test material	Test model	Result	Klimisch score	Perineal exposure (Yes/No)
Boorman and Seely (1995) [68] *(data based on NTP Study (1993) [67])*	Talc: high purity, microtalc, MP (10-52 Grade, max particle size is 10 µm and contained no tremolite or asbestiforms, free of silica - 1 particle of silica was detected in 1,466 particles examined) was obtained from Walsh and Associates, USA.	Female Fisher 344/F rats (n=10/group) Dose: 0, 6 and 18 mg/m^3 Control group: Sham exposed animals (n=10) Duration of exposure: lifetime. Ovaries were examined histologically and under polarized light for the presence of talc particles.	No talc particles were detected in ovaries or ovarian bursa in any rats from any group. Exposure route: inhalation - whole body exposure which suggests that exposure occurred through dermal and oral routes as well as pulmonary.	5	No
Boorman and Seely (1995) [68] *(data based on NTP*	Talc: high purity, microtalc, MP (10-52 Grade, max particle size is 10 µm and contained	Female B6C3F1 mice (n=10/group). Dose: 0, 6 and 18 mg/m^3	No talc particles were detected in ovaries or ovarian bursa in any mice from any group. Exposure route: by inhalation - whole body exposure which	5	No

Reference	Test material	Test model	Result	Klimisch score	Perineal exposure (Yes/No)
Study (1993) [67])	no tremolite or asbestiforms, free of silica - 1 particle of silica was detected in 1,466 particles examined) was obtained from Walsh and Associates, USA.	Control group: Sham exposed animals (n=10) Duration of exposure: 2 years Ovaries were examined histologically and under polarized light for the presence of talc particles.	suggest that exposure was occurred through dermal and oral routes of exposure.		
Hanson et al. (1985) [111]	Respirable talc (8<µm aerodynamic diameter)	I. Fisher rats (n=5/sex/group). II. CD-1 mice (n= 10/group). Exposure route: Inhalation. Dose: I. Rats: 0, 2.3, 4.3 or 17 mg talc/m3.	More talc was deposited in mouse than in rat lungs at the highest exposure (17-20.6 mg/m3 talc): Rats - maximum levels of 806±135 ug/g lung Mice - 1150± 101 ug/g lung. >89% of Mg was recovered from lungs at 4 weeks of post-exposure.	5	No

Reference	Test material	Test model	Result	Klimisch score	Perineal exposure (Yes/No)
		II. Mice: 2.2, 6.3 or 20.6 mg talc/m3.<br><br>Control group: Untreated animals.<br><br>Duration of exposure: 6 h. per day/5 days per week for 4 weeks.	Data suggested that only minimal concentrations of talc are systemically available following inhalation.<br><br>A species – specific difference was observed of talc deposition in lungs of rodents.		
Pickrell et al. (1989) [69]	Asbestos- free talc, respirable talc (2.7 - 3.3 µm), contained 19.2-19.4% of magnesium (Mg)	Male and female F344/Crl rats (n=10/sex/group).<br><br>Male nd female B6C3F1 mice<br>(n=10/sex/group).<br><br>Exposure route: inhalation.<br>Duration:<br>Concentrations:<br>Rats: 0, 2.3, 4.3, 17 mg talc/m3;	A diffuse increase of talc-containing free macrophages within alveolar spaces in both rat and mouse groups exposed to the highest level of talc.<br><br>Pickrell et al. (1989) suggested that continued exposure and accumulation of talc in ovaries following perineal exposure can led to morphologic changes consistent with inhibited<br>phagocytic removal of dust (Wolff et al., (1984) [112].	5	No

Reference	Test material	Test model	Result	Klimisch score	Perineal exposure (Yes/No)
		Mice: 0, 2.2, 5.7, 20.4 mg talc/m^3<br><br>Control group:<br>Animals exposed to filtered air.<br><br>Duration of exposure:<br>6 h per day/5 days per week/ 4 weeks (20 days).	It was reported that the accumulation of 1 mg or more talc/g lung (wet weight) would cause lung burdens which might produce lung lesions in humans (Pickrell et al., 1989), however, no such data exists for ovaries.		
Wehner et al. (1977) [113]	Talc: high-grade cosmetic talc (Johnsons's baby Powder®, lot 228p) and consisted of 95% (w/w) platy talc mineral; median aerodynamic diameter (MMAD) - 6.4–6.9 µm.	Female<br>golden Syrian hamsters (n=1-5/group).<br><br>Exposure route:<br>Single inhalation exposure (nose-only).<br><br>Dose: 40–75 mg/m3 neutron-activated talc.<br><br>Control: talc-untreated animals.	Alveolar deposition was approximately 20–80 µg, which represented 6–8% of the inhaled amount.<br><br>Lung<br>The retention half-time of the deposited talc in the alveoli was 7–10 days, and a complete alveolar clearance was reported in approximately 4 months after exposure.<br><br>Ovary	5	

Reference	Test material	Test model	Result	Klimisch score	Perineal exposure (Yes/No)
		Duration of observation: 15 min – 132 days.	Talc radioactivity counts increased 52% from 100 min to 132 day post exposure (2.00 ± 1.14 versus 3.05 ± 0.60, respectively), and<br><br>39% at 132 day of post exposure compared to the controls (3.05 ± 0.60 versus 2.18 ± 0.62) (p>0.05).<br><br>Talc-associated radioactivity counts did not decrease with time in both talc-exposed and control animals.<br><br>It is not clear why talc-based radioactivity was observed in control animals.		
Phillips et al. (1978) [114]	Talc: tritium [3H] labelled talc (asbestos-free), 8.4 and 12-µm talc particles	Male rats (n=3/group), female mice (n=4/group) and female guinea pigs (n=3/group).<br><br>Exposure route: oral (gavage).	Rats<br><br>Single dose:<br><br>At 96 h.: 95.8% of the radioactivity was excreted in the feces and 1.67% of dose was excreted in urine from 0 - 96 h and 0. 08% of radioactivity	5	No

Reference	Test material	Test model	Result	Klimisch score	Perineal exposure (Yes/No)
		Group I (rats): 50 mg/kg bw, a single dose or repeated dose (6 days, daily)<br><br>Group II (mice): 40 mg/kg bw, a single dose.<br><br>Group III (guinea pigs): 25 mg/kg, a single dose.<br><br>Control group: Untreated animals.<br><br>Exposure duration: rats –4 and 10 days; mice – 6 h and 24 h., guinea pigs – 10 days.	remained in the gastrointestinal tract.<br><br>At 240 h.: no radioactivity was detected in liver, 0.02% of the administered dose was detected in kidneys.<br><br>Guinea pigs<br><br>At 96 h.: 94.4% of the radioactivity was excreted in the feces and 0.2% of dose was excreted in urine from 0 - 96 h<br><br>At 240 h.: less that 0. 03% of radioactivity remained in the gastrointestinal tract.<br><br>Mice<br><br>At 24 h.: 100% of the administered dose was recovered in feces and 1.38% was excreted in urine. No radioactivity was detected in the carcasses.		

Reference	Test material	Test model	Result	Klimisch score	Perineal exposure (Yes/No)
			Absorption of talc in guinea pigs was 8 times less compared to rats based on radioactivity excreted in urine (1.67% vs 0.2%, respectively).<br><br>In mice, the dose recovered from feces and urine at 24 h. was higher compared to rats at the same time of postexposure (100 and 1.38% versus 74.8 and 1.05%, respectively).		
Wehner et al. (1977) [115]	Talc: neutron-activated talc (Johnsons's baby Powder®, lot 228p)	Female golden Syrian hamsters (n=44/group).<br><br>Exposure route: oral (gavage).<br><br>Dose: 2.94 mg talc in 1% methyl cellulose /physiological saline/hamster.<br><br>Control group: untreated animals	At 24 h. post-exposure, approximately, 98% of the administered radioactivity was found in the feces and gastrointestinal tract (74.5 and 23.5%, respectively).<br><br>No significant differences in radioactivity between control and talc-treated animals were found in the lungs, kidneys and liver, urine accounted for 0.09% of the radioactivity, the skinned carcasses accounted for 1.9%.	5	No

Reference	Test material	Test model	Result	Klimisch score	Perineal exposure (Yes/No)
		Duration of exposure: 24 h.	Bioavailability of talc is low - approximately 2%.		
Fraticelli et al. (2002) [116]	Talc: asbestos-free calibrated talc with median particle size<br>31 µm	Wistar male rats (n=4-18/group).<br>Exposure route: Intrapleural instillation: 40 mg talc in 1 mL sterile saline solution.<br>Control group: animals received chest tube drainage without talc.<br>Duration of exposure: 24 and 72 h.	Talc was observed in lung and visceral pleura, parietal pleura, diaphragm and pericardium at 24 and 72 h after intrapleural administration.<br>Talc particles were observed (with a low incidence) in few extrapulmonary organs:<br>brain (1 particle in 1 animal, 24 h.), spleen (1 particle in 1 animal, 24 h.) and liver (5 and 75 particles in 1 animal at 24 and 72 h., respectively),but not in kidneys or blood.	5	No
Ferrer et al. (2002) [117]	8.4-µm asbestos-free talc particles and 12-µm talc particles	Rabbits (n=10/group).<br>Exposure route: Intrapleural instillation.	Talc particles were observed:<br>kidneys – 1/5 rats (Group I) 24 h post-exposure.<br>liver - in 3/5 rats; (Group I) 7 days after instillation.	5	No

Reference	Test material	Test model	Result	Klimisch score	Perineal exposure (Yes/No)
		Group I: 200 mg/kg bw (8.4 µm talc).<br><br>Group II:<br>200 mg/kg bw (12.4 µm talc).	spleen – 1/5 animals (Group I and II), 24 h post-exposure.		
Genofre et al. (2009) [86]	Talc: Sterilized talc slurry	New Zealand white rabbits (n=10/group).<br><br>Exposure route: Single intrapleural injection<br><br>Dose:<br>Group I (MP): 400 mg/kg (3 mL) in 2 ml saline ("mixed particles" (mean size): 25.4 µm)<br><br>Group II (SP): 400 mg/kg in 2 ml saline ("small particles" (mean size): 4.2 µ)	Talc particles were detected<br>in lungs,<br>in liver (parenchyma, perivascular regions),<br>in spleen (the highest levels, predominantly in red pulp) and<br>kidneys (medullary layer)<br><br>Significantly more particles were observed in internal organs of animals injected with smaller particles (Group II)<br><br>A higher ratio of deposition was observed in Group II (SP) versus Group I (MP)<br>in the lungs (65/2 for both right and left lung),	5	No

Reference	Test material	Test model	Result	Klimisch score	Perineal exposure (Yes/No)
		Control group: injected with 2 mL of saline<br><br>Animals were sacrificed 6, 24 or 48 hr post-injection.	liver (60/2),<br>spleen (140/120) and<br>kidney (10/0.5) ($p < 0.05$).		
Werebe et al. (1999) [118]	Talc: talc crystals employed for pleurodesis (no other details provided)	Wistar rats (n=9-10/group).<br><br>Exposure route: Intrapleural administration.<br><br>Group I: 20 mg of talc slurry in 1 mL of saline solution (extrapolated from the clinical dose of 5 g. in 70 kg man).<br><br>Group II: 10 mg of talc slurry in 1 mL of saline solution.<br><br>Control group: received saline alone.	In the lungs;<br>dust-laden macrophages with inflammatory infiltrates of lymphocytes associated with fibroblastic activity were observed.<br><br>Talc deposition was detected in both groups - in the chest wall, lungs, heart, brain, spleen and kidneys using polarised light), independent of the time of necropsy.<br><br>Dose-response was observed only for chest wall, lungs and kidneys.<br><br>Werebe et al. (1999) concluded that talc is rapidly absorbed	5	No

Reference	Test material	Test model	Result	Klimisch score	Perineal exposure (Yes/No)
		Exposure duration: 24 and 48 h.	through the pleura and reaches the systemic circulation and organs 24 hours after administration.		
Phillips et al. (1978) [114]	Talc: tritium [3H] labelled talc (asbestos-free), 8.4 and 12-μm talc particles	Male rats (n=2/group).<br><br>Route of exposure: s.c. injection<br><br>Dose: 10 mg in 0.1 ml.<br><br>Duration of exposure: 5 days.	97.3-99.4% of the talc-associated radioactivity was recovered in the s.c. granuloma excited from rats after talc injection.<br><br>No radioactivity was detected in the urine of rats at 5 day after talc injection.	5	No
Henderson et al. (1986) [119]	Talc: details are not available	Female Sprague-Dawley rats (n=6/group).<br><br>Dose:<br><br>100 mg/mL talc in phosphate-buffered saline (FBS).<br><br>Exposure route:<br><br>Intravaginal exposure.	Talc particles were detected in the ovaries of 2 rats received intravaginal talc after 4 days of post-exposure.<br><br>No talc was observed in ovaries from rats received intravaginal talc and sacrificed at 24 and 48 h. post-exposure.	5	Yes

Reference	Test material	Test model	Result	Klimisch score	Perineal exposure (Yes/No)
		Control group: received FSB saline.<br><br>Duration of exposure:<br>24, 24 h and 4 days.			
Phillips et al. (1978) [114]	Talc: tritium [3H] labelled talc (asbestos-free), 8.4 and 12-µm talc particles	Rabbits (n=3/group).<br><br>Exposure route: intravaginal administration.<br><br>Group: 0.5 mL of talc (in solution), a single dose or a repeated dose (6 days, daily).<br><br>Control group: Untreated animals.<br><br>Exposure duration:<br>single exposure: 3 days;	At 72 h. post-administration:<br>radioactivity was detected only “at the site of administration” – 0.004%±0.001%.<br><br>72 h. after the last of six daily intravaginal doses:<br>Radioactivity was detected “at the site of administration” - 0.035 ± 0.024% of dose and<br>in cervix, fallopian /uterine tubes – 0. 006 ± 0.003%<br>No radioactivity was found in ovaries.<br><br>Evidence suggest that talc particles can migrate inside of the reproductive tract of rabbits.	5	Yes

Reference	Test material	Test model	Result	Klimisch score	Perineal exposure (Yes/No)
		repeated exposure: 6 days. Animals were sacrificed 72 h after the last dose.			
Wehner et al. (1986)[120]	Talc: neutron-activated purified blend of cosmetic talc	Female Exbreeder cynomolgus monkeys (n=6/group).<br><br>Exposure route: Intravaginal administration.<br><br>Dose: 125 mg neutron activated talc suspended in 0.3 mL saline, contained 1% carboxymethyl cellulose.<br><br>Control group: untreated animals.<br><br>Duration of exposure: 30 days.	Two days following 30 daily administrations of talc, talc was detected only in the vagina and cervix of talc-treated animals.<br><br>Radioactivity was not found in peritoneal lavage fluid, ovaries, oviducts, uterus, vagina, or cervix.<br><br>In the earlier study, Wehner et al. (1985) reported in cynomolgus monkeys that no measurable quantities (<0.5 μg) of talc were translocated from vagina to the uterine cavity and beyond.	5	Yes

Reference	Test material	Test model	Result	Klimisch score	Perineal exposure (Yes/No)
Henderson et al. (1986) [119]	Talc: details are not available	Female Sprague-Dawley rats (n=4/group).<br><br>Dose:<br>100 mg/mL talc in phosphate-buffered saline (FBS).<br><br>Exposure route:<br>Intrauterine exposure.<br><br>Control group: received FSB saline.<br><br>Duration of exposure:<br>1 day, 6, 15, 22 and 30 days.	Talc particles were detected in the ovaries of all rats treated with intrauterine talc (no other details provided).	5	Yes
Hamilton et al. (1984) [83]	Talc: Italian talc (0.3-14 µm), asbestos-free.	Female Sprague-Dawley rat (n=10/group).<br><br>Exposure route: Talc suspension in phosphate-buffered saline was	Foreign body granulomas (without surrounding inflammation) were observed in 5 of talc injected ovaries.	5	

Reference	Test material	Test model	Result	Klimisch score	Perineal exposure (Yes/No)
		injected into the ovarian bursa.<br><br>Concentration: 100 mg/mL in the sterilized phosphate buffered saline.<br><br>Control group: Sham-operated rats<br><br>Duration of observation: 1,3,6,12 and 18 months.<br><br>Talc presence on the surface epithelium, ovarian cortex and matrix of the bursa was analysed using polarized light and electron microscope microanalysis.<br><br>The presence of talc in granulomas was analysed by electron microscopy.	Talc was detected on the surface epithelium, ovarian cortex and matrix of the bursa.<br><br>Talc was detected in granulomas.		

Critical Review of the Association between Perineal Use of Talc and Risk of Ovarian Cancer

Supplementary Material XI

Quantitative Analysis

Supplementary Material XI: Meta-analysis of retained human studies on perineal talc exposure

Methodology

We conducted a meta-analysis of perineal use of talc and the risk of ovarian cancer using quantitative risk estimates as reported in the original studies. The final analysis included 27 original studies comprising three cohort studies and twenty-four case-control studies. Studies that had analyzed overlapping study populations were assessed on a case-by-case basis for inclusion into the meta-analysis. The level of detail in the reported findings, including sample size and publication date, were considered when deciding which study to include in the case of overlap. Table 8 provides the rationale for the exclusion of certain studies from the meta-analysis. A similar approach was adopted by Berge and colleagues [38], leading to the inclusion of studies with most detailed analyses or the larger sample size.

The maximally adjusted odds ratios (ORs), hazard ratios (HRs) or relative risks (RRs) were extracted from the original studies. Summary effects, expressed as pooled odds ratios, were derived from individual studies by a random effects model using the inverse variance method with 95% confidence intervals [121]. RRs, ORs and HRs were assumed to be essential equivalent measures of risk because of the rare endpoint (ovarian cancer) assessed. While risk estimates could often be obtained from at least two original studies, this was not always the case in subgroup analyses (only one study could be identified for some subgroups). Although a subgroup analyses based on a single risk estimate is not sufficient on its own, it does add value when examined in

comparison with the other subgroups in the same analysis. A random effects model was used to account for heterogeneity among the included studies [121] .

The initial analysis involved an evaluation of the type of perineal use of talc powder (ever vs. never use). Other subgroup analyses assessed the duration and frequency of talc use, tumor histology, tumor behavior, and the possible effect of menopausal state, hormone use, and pelvic surgery. We assessed the included studies with respect to study design, type of study controls, quality score using the NOS [61], and publication year. Additionally, we assessed the influence of individual studies on the overall pooled effect in a systematic fashion by excluding one study at a time from the analysis [122]. All calculations were performed using Review Manager application version 5.3 [123].

Table 8: Rationale for exclusion of specific original studies from this review

**Relevant Studies**[12]	**Study Included in the Meta-Analysis**	**Rationale**
Purdie et al. (1995)<br>Green et al. (1997) [41]	Green et al. (1997) [41]	Both studies analyzed the same study sample; Green et al. (1997) reported findings adjusted for parity in addition to other factors not adjusted for in Purdie et al. (1995)
Cramer et al. (1999) [16]<br>Cramer et al. (2016) [52]	Cramer et al. (2016) [52]	Cramer et al. (1999) analyzed a subsample of Cramer et al. (2016)

[12] The presence of more than one reference per study reflects additional follow-up, updates, or further analyses on the same study group.

**Relevant Studies[12]**	**Study Included in the Meta-Analysis**	**Rationale**
Cramer et al. (2005) [124]<br>Cramer et al. (2016) [52]	Cramer et al. (2016) [52]	Cramer et al. (2005) analyzed a subsample of Cramer et al. (2016)
Vitonis et al. (2011) [125]<br>Cramer et al. (2016) [52]	Cramer et al. (2016) [52]	Vitonis et al. (2011) analyzed a subsample of Cramer et al. (2016)
Cramer & Xu (1995) [39]<br>Cramer et al. (1982)[2]	Cramer et al. (1982)[2]	Findings in Cramer et al. (1982) are more relevant to the outcome of interest although the study sample in Cramer et al (1982) is a subset of Cramer and Xu (1995).
Urban et al. (2015) [126]<br>Houghton et al. (2014) [55]	Houghton et al. (2014) [55]	Houghton et al. (2014) analyzed a subsample of Urban et al. (2015), but Houghton et al. (2014) reports more detailed findings relevant to the outcome of interest.
Kurta & Diergaarde (2011) [127]<br>Kurta et al. (2012) [49]	Kurta et al. (2012) [49]	We opted to use Kurta et al. (2012) since Kurta & Diergaarde (2011) is just a conference abstract.
Gertig et al. (2000) [20] & Gates et al. (2010) [54]	Gertig et al. (2000) [20]	Gates et al. (2010) reports effect estimates for a reference group (< 1 use talc/week) which was inconsistent with the reference groups of other studies (never used talc). Both studies were included in the systematic review, but only Gertig et al. (2000) was included in the meta-analysis.

Overall perineal talc use

Perineal talc use and ovarian cancer

Study or Subgroup	log[Odds Ratio]	SE	Weight	Odds Ratio IV, Random, 95% CI	Year
Cramer 1982	0.6523	0.2109	2.0%	1.92 [1.27, 2.90]	1982
Hartge 1983	-0.3567	0.2855	1.2%	0.70 [0.40, 1.22]	1983
Whittemore 1988	0.3716	0.2971	1.1%	1.45 [0.81, 2.60]	1988
Harlow 1989	0.0953	0.2306	1.7%	1.10 [0.70, 1.73]	1989
Harlow 1992	0.4055	0.2069	2.1%	1.50 [1.00, 2.25]	1992
Rosenblatt 1992	0	0.8212	0.2%	1.00 [0.20, 5.00]	1992
Tzonou 1993	0.0488	0.6744	0.2%	1.05 [0.28, 3.94]	1993
Green 1997	0.2624	0.0852	7.0%	1.30 [1.10, 1.54]	1997
Chang 1997	0.3507	0.1396	3.9%	1.42 [1.08, 1.87]	1997
Cook 1997	0.47	0.1912	2.4%	1.60 [1.10, 2.33]	1997
Godard 1998	0.9123	0.497	0.4%	2.49 [0.94, 6.60]	1998
Wong 1999	0	0.1139	5.1%	1.00 [0.80, 1.25]	1999
Gertig 2000	0.0862	0.1209	4.7%	1.09 [0.86, 1.38]	2000
Ness 2000	0.4055	0.1582	3.2%	1.50 [1.10, 2.05]	2000
Langseth 2004	0.1398	0.5262	0.4%	1.15 [0.41, 3.23]	2004
Mills 2004	0.3148	0.1505	3.5%	1.37 [1.02, 1.84]	2004
Merritt 2008	0.157	0.075	7.8%	1.17 [1.01, 1.36]	2008
Gates 2008	0.3075	0.09	6.6%	1.36 [1.14, 1.62]	2008
Moorman 2009	0.0583	0.1126	5.2%	1.06 [0.85, 1.32]	2009
Wu 2009	0.4253	0.1546	3.3%	1.53 [1.13, 2.07]	2009
Rosenblatt 2011	0.239	0.1375	4.0%	1.27 [0.97, 1.66]	2011
Kurta 2012	0.3365	0.0959	6.2%	1.40 [1.16, 1.69]	2012
Houghton 2014	0.1133	0.1004	5.9%	1.12 [0.92, 1.36]	2014
Wu 2015	0.3784	0.0711	8.1%	1.46 [1.27, 1.68]	2015
Schildkraut 2016	0.3646	0.1328	4.2%	1.44 [1.11, 1.87]	2016
Gonzalez 2016	-0.3147	0.2583	1.4%	0.73 [0.44, 1.21]	2016
Cramer 2016	0.2776	0.0703	8.2%	1.32 [1.15, 1.51]	2016
**Total (95% CI)**			**100.0%**	**1.28 [1.20, 1.37]**	

Heterogeneity: Tau² = 0.01; Chi² = 38.74, df = 26 (P = 0.05); I² = 33%
Test for overall effect: Z = 7.65 (P < 0.00001)

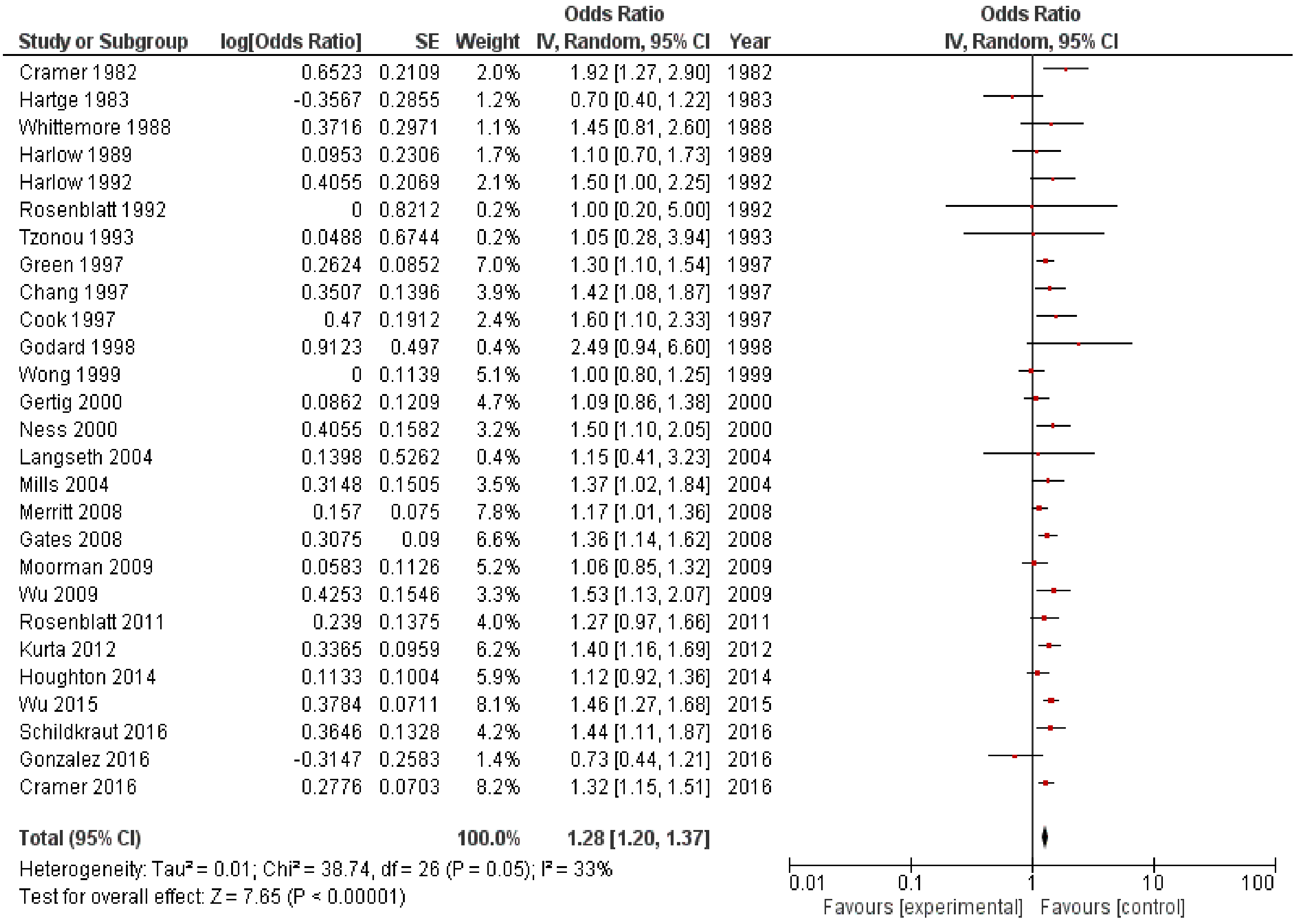

Forest plots of the subgroup analyses of perineal talc use

Perineal talc use and ovarian cancer by race/ethnicity

Study or Subgroup	log[Odds Ratio]	SE	Weight	Odds Ratio IV, Random, 95% CI	Year
**1.2.1 African Americans**					
Moorman 2009	0.174	0.2855	10.5%	1.19 [0.68, 2.08]	2009
Wu 2015	0.4447	0.3407	8.7%	1.56 [0.80, 3.04]	2015
Cramer 2016	1.6253	0.6876	3.1%	5.08 [1.32, 19.55]	2016
**Subtotal (95% CI)**			**22.3%**	**1.67 [0.90, 3.10]**	
Heterogeneity: Tau² = 0.14; Chi² = 3.82, df = 2 (P = 0.15); I² = 48%					
Test for overall effect: Z = 1.63 (P = 0.10)					
**1.2.2 Hispanics**					
Wu 2015	0.571	0.1983	14.2%	1.77 [1.20, 2.61]	2015
Cramer 2016	0.0953	0.6629	3.3%	1.10 [0.30, 4.03]	2016
**Subtotal (95% CI)**			**17.5%**	**1.70 [1.17, 2.47]**	
Heterogeneity: Tau² = 0.00; Chi² = 0.47, df = 1 (P = 0.49); I² = 0%					
Test for overall effect: Z = 2.80 (P = 0.005)					
**1.2.3 Whites**					
Moorman 2009	0.0392	0.1213	17.8%	1.04 [0.82, 1.32]	2009
Wu 2015	0.3436	0.078	19.6%	1.41 [1.21, 1.64]	2015
Cramer 2016	0.2852	0.0698	19.9%	1.33 [1.16, 1.53]	2016
**Subtotal (95% CI)**			**57.3%**	**1.28 [1.11, 1.49]**	
Heterogeneity: Tau² = 0.01; Chi² = 4.56, df = 2 (P = 0.10); I² = 56%					
Test for overall effect: Z = 3.29 (P = 0.001)					
**1.2.4 Asians**					
Cramer 2016	-3.2189	0.7073	2.9%	0.04 [0.01, 0.16]	2016
**Subtotal (95% CI)**			**2.9%**	**0.04 [0.01, 0.16]**	
Heterogeneity: Not applicable					
Test for overall effect: Z = 4.55 (P < 0.00001)					
**Total (95% CI)**			**100.0%**	**1.26 [0.97, 1.62]**	
Heterogeneity: Tau² = 0.08; Chi² = 35.51, df = 8 (P < 0.0001); I² = 77%					
Test for overall effect: Z = 1.74 (P = 0.08)					
Test for subgroup differences: Chi² = 26.91, df = 3 (P < 0.00001), I² = 88.9%					

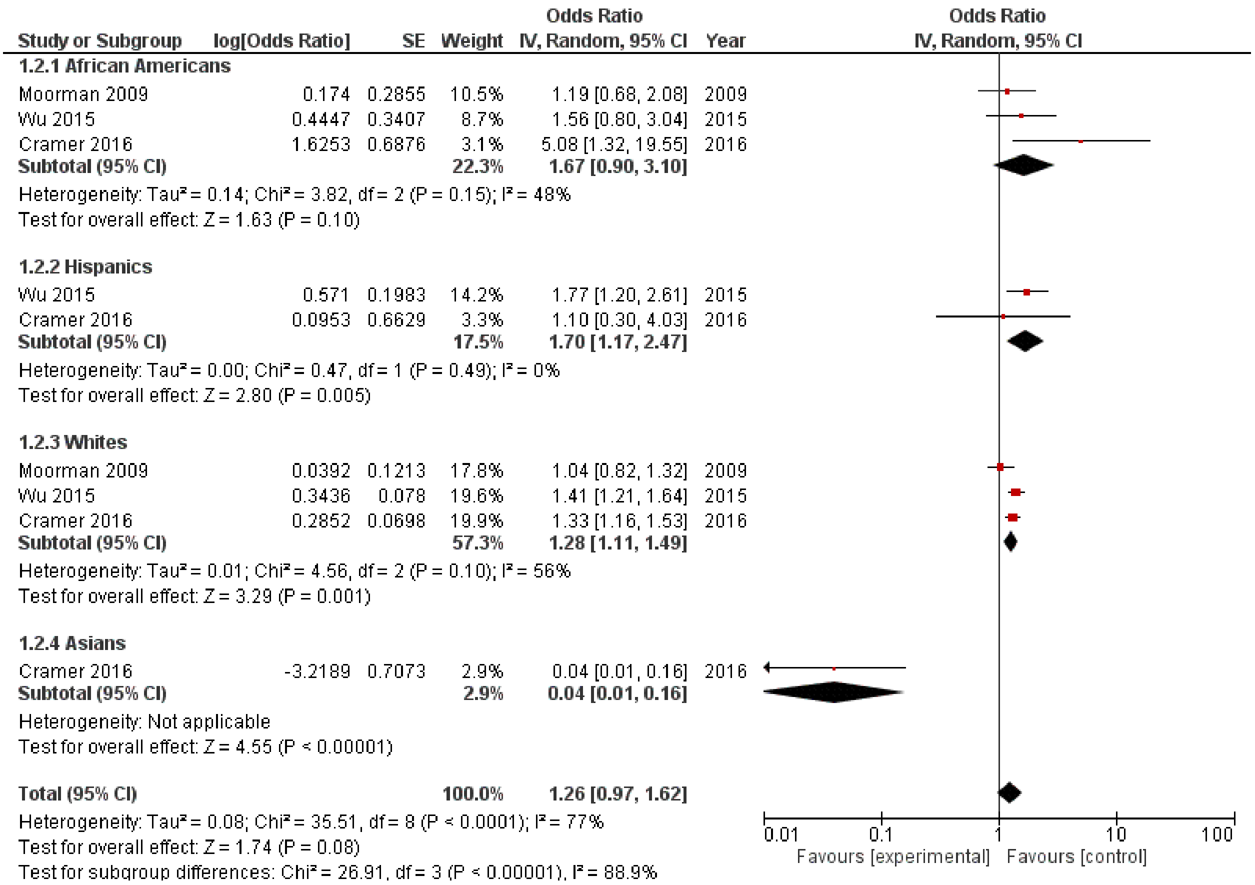

Perineal talc use – assessment of reporting studies

Perineal talc use and ovarian cancer, by study design

Study or Subgroup	log[Odds Ratio]	SE	Weight	Odds Ratio IV, Random, 95% CI	Year
**2.1.1 Case-Control**					
Cramer 1982	0.6523	0.2109	2.0%	1.92 [1.27, 2.90]	1982
Hartge 1983	-0.3567	0.2855	1.2%	0.70 [0.40, 1.22]	1983
Whittemore 1988	0.3716	0.2971	1.1%	1.45 [0.81, 2.60]	1988
Harlow 1989	0.0953	0.2306	1.7%	1.10 [0.70, 1.73]	1989
Rosenblatt 1992	0	0.8212	0.2%	1.00 [0.20, 5.00]	1992
Harlow 1992	0.4055	0.2069	2.1%	1.50 [1.00, 2.25]	1992
Tzonou 1993	0.0488	0.6744	0.2%	1.05 [0.28, 3.94]	1993
Chang 1997	0.3507	0.1396	3.9%	1.42 [1.08, 1.87]	1997
Cook 1997	0.47	0.1912	2.4%	1.60 [1.10, 2.33]	1997
Green 1997	0.2624	0.0852	7.0%	1.30 [1.10, 1.54]	1997
Godard 1998	0.9123	0.497	0.4%	2.49 [0.94, 6.60]	1998
Wong 1999	0	0.1139	5.1%	1.00 [0.80, 1.25]	1999
Ness 2000	0.4055	0.1582	3.2%	1.50 [1.10, 2.05]	2000
Langseth 2004	0.1398	0.5262	0.4%	1.15 [0.41, 3.23]	2004
Mills 2004	0.3148	0.1505	3.5%	1.37 [1.02, 1.84]	2004
Gates 2008	0.3075	0.09	6.6%	1.36 [1.14, 1.62]	2008
Merritt 2008	0.157	0.075	7.8%	1.17 [1.01, 1.36]	2008
Wu 2009	0.4253	0.1546	3.3%	1.53 [1.13, 2.07]	2009
Moorman 2009	0.0583	0.1126	5.2%	1.06 [0.85, 1.32]	2009
Rosenblatt 2011	0.239	0.1375	4.0%	1.27 [0.97, 1.66]	2011
Kurta 2012	0.3365	0.0959	6.2%	1.40 [1.16, 1.69]	2012
Wu 2015	0.3784	0.0711	8.1%	1.46 [1.27, 1.68]	2015
Cramer 2016	0.2776	0.0703	8.2%	1.32 [1.15, 1.51]	2016
Schildkraut 2016	0.3646	0.1328	4.2%	1.44 [1.11, 1.87]	2016
**Subtotal (95% CI)**			**88.0%**	**1.32 [1.24, 1.40]**	
Heterogeneity: Tau² = 0.00; Chi² = 29.38, df = 23 (P = 0.17); I² = 22%					
Test for overall effect: Z = 8.77 (P < 0.00001)					
**2.1.2 Cohort**					
Gertig 2000	0.0862	0.1209	4.7%	1.09 [0.86, 1.38]	2000
Houghton 2014	0.1133	0.1004	5.9%	1.12 [0.92, 1.36]	2014
Gonzalez 2016	-0.3147	0.2583	1.4%	0.73 [0.44, 1.21]	2016
**Subtotal (95% CI)**			**12.0%**	**1.06 [0.90, 1.25]**	
Heterogeneity: Tau² = 0.00; Chi² = 2.42, df = 2 (P = 0.30); I² = 17%					
Test for overall effect: Z = 0.69 (P = 0.49)					
**Total (95% CI)**			**100.0%**	**1.28 [1.20, 1.37]**	
Heterogeneity: Tau² = 0.01; Chi² = 38.74, df = 26 (P = 0.05); I² = 33%					
Test for overall effect: Z = 7.65 (P < 0.00001)					
Test for subgroup differences: Chi² = 5.70, df = 1 (P = 0.02), I² = 82.5%					

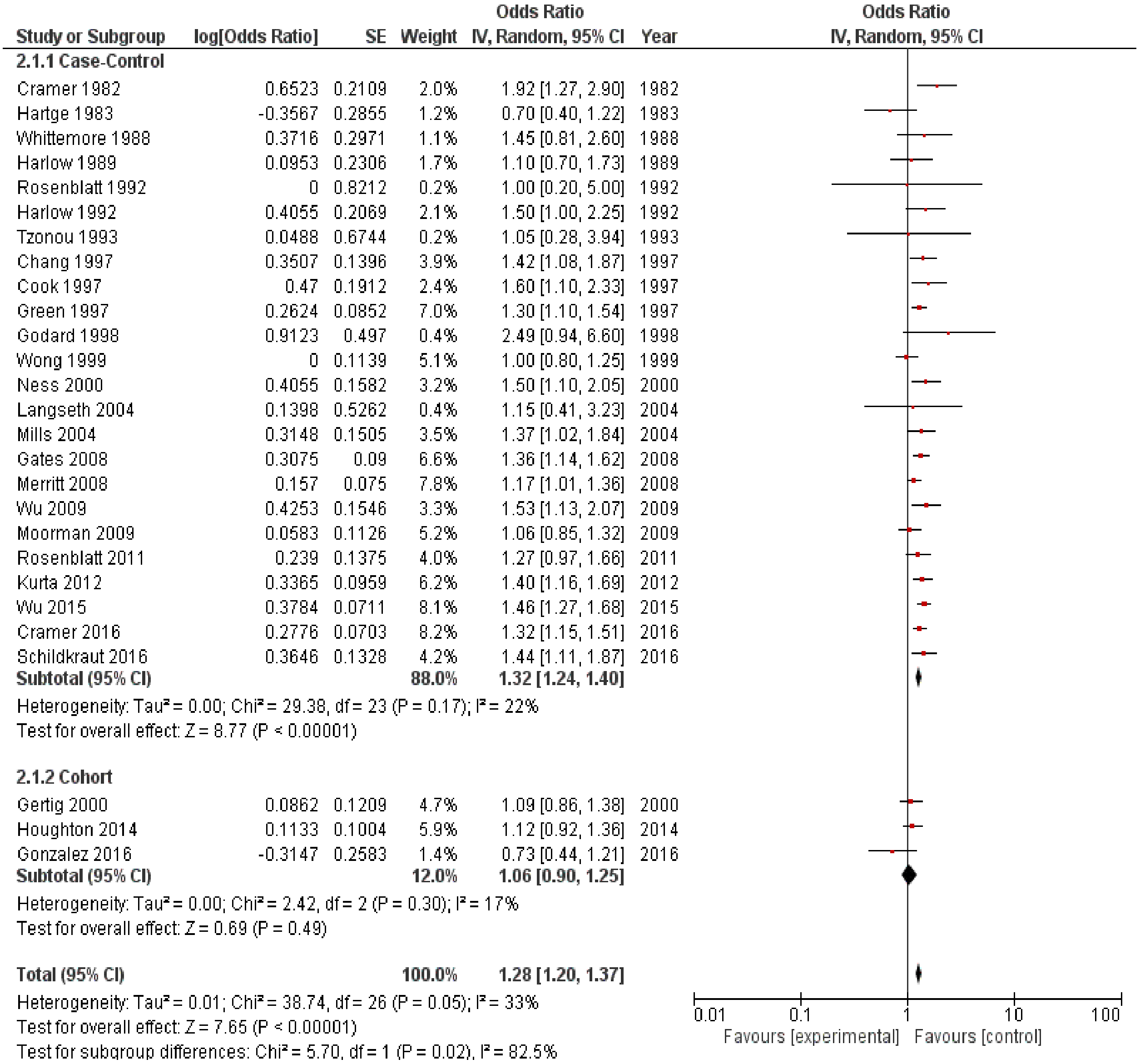

Perineal talc use and ovarian cancer, by type of study controls

Study or Subgroup	log[Odds Ratio]	SE	Weight	Odds Ratio IV, Random, 95% CI	Year
**2.2.1 Hospital-based**					
Hartge 1983	-0.3567	0.2855	1.2%	0.70 [0.40, 1.22]	1983
Rosenblatt 1992	0	0.8212	0.1%	1.00 [0.20, 5.00]	1992
Tzonou 1993	0.0488	0.6744	0.2%	1.05 [0.28, 3.94]	1993
Wong 1999	0	0.1139	5.6%	1.00 [0.80, 1.25]	1999
**Subtotal (95% CI)**			**7.2%**	**0.96 [0.78, 1.17]**	
Heterogeneity: Tau² = 0.00; Chi² = 1.37, df = 3 (P = 0.71); I² = 0%					
Test for overall effect: Z = 0.44 (P = 0.66)					
**2.2.2 Population-based**					
Cramer 1982	0.6523	0.2109	2.0%	1.92 [1.27, 2.90]	1982
Harlow 1989	0.0953	0.2306	1.7%	1.10 [0.70, 1.73]	1989
Harlow 1992	0.4055	0.2069	2.1%	1.50 [1.00, 2.25]	1992
Cook 1997	0.47	0.1912	2.4%	1.60 [1.10, 2.33]	1997
Chang 1997	0.3507	0.1396	4.1%	1.42 [1.08, 1.87]	1997
Green 1997	0.2624	0.0852	8.4%	1.30 [1.10, 1.54]	1997
Godard 1998	0.9123	0.497	0.4%	2.49 [0.94, 6.60]	1998
Ness 2000	0.4055	0.1582	3.3%	1.50 [1.10, 2.05]	2000
Mills 2004	0.3148	0.1505	3.6%	1.37 [1.02, 1.84]	2004
Langseth 2004	0.1398	0.5262	0.4%	1.15 [0.41, 3.23]	2004
Merritt 2008	0.157	0.075	9.7%	1.17 [1.01, 1.36]	2008
Gates 2008	0.3075	0.09	7.8%	1.36 [1.14, 1.62]	2008
Moorman 2009	0.0583	0.1126	5.7%	1.06 [0.85, 1.32]	2009
Wu 2009	0.4253	0.1546	3.5%	1.53 [1.13, 2.07]	2009
Rosenblatt 2011	0.239	0.1375	4.2%	1.27 [0.97, 1.66]	2011
Kurta 2012	0.3365	0.0959	7.2%	1.40 [1.16, 1.69]	2012
Wu 2015	0.3784	0.0711	10.3%	1.46 [1.27, 1.68]	2015
Schildkraut 2016	0.3646	0.1328	4.5%	1.44 [1.11, 1.87]	2016
Cramer 2016	0.2776	0.0703	10.4%	1.32 [1.15, 1.51]	2016
**Subtotal (95% CI)**			**91.8%**	**1.34 [1.27, 1.41]**	
Heterogeneity: Tau² = 0.00; Chi² = 17.78, df = 18 (P = 0.47); I² = 0%					
Test for overall effect: Z = 11.08 (P < 0.00001)					
**2.2.3 Combined**					
Whittemore 1988	0.3716	0.2971	1.1%	1.45 [0.81, 2.60]	1988
**Subtotal (95% CI)**			**1.1%**	**1.45 [0.81, 2.60]**	
Heterogeneity: Not applicable					
Test for overall effect: Z = 1.25 (P = 0.21)					
**Total (95% CI)**			**100.0%**	**1.32 [1.24, 1.40]**	

Heterogeneity: Tau² = 0.00; Chi² = 29.38, df = 23 (P = 0.17); I² = 22%
Test for overall effect: Z = 8.77 (P < 0.00001)
Test for subgroup differences: Chi² = 10.23, df = 2 (P = 0.006), I² = 80.4%

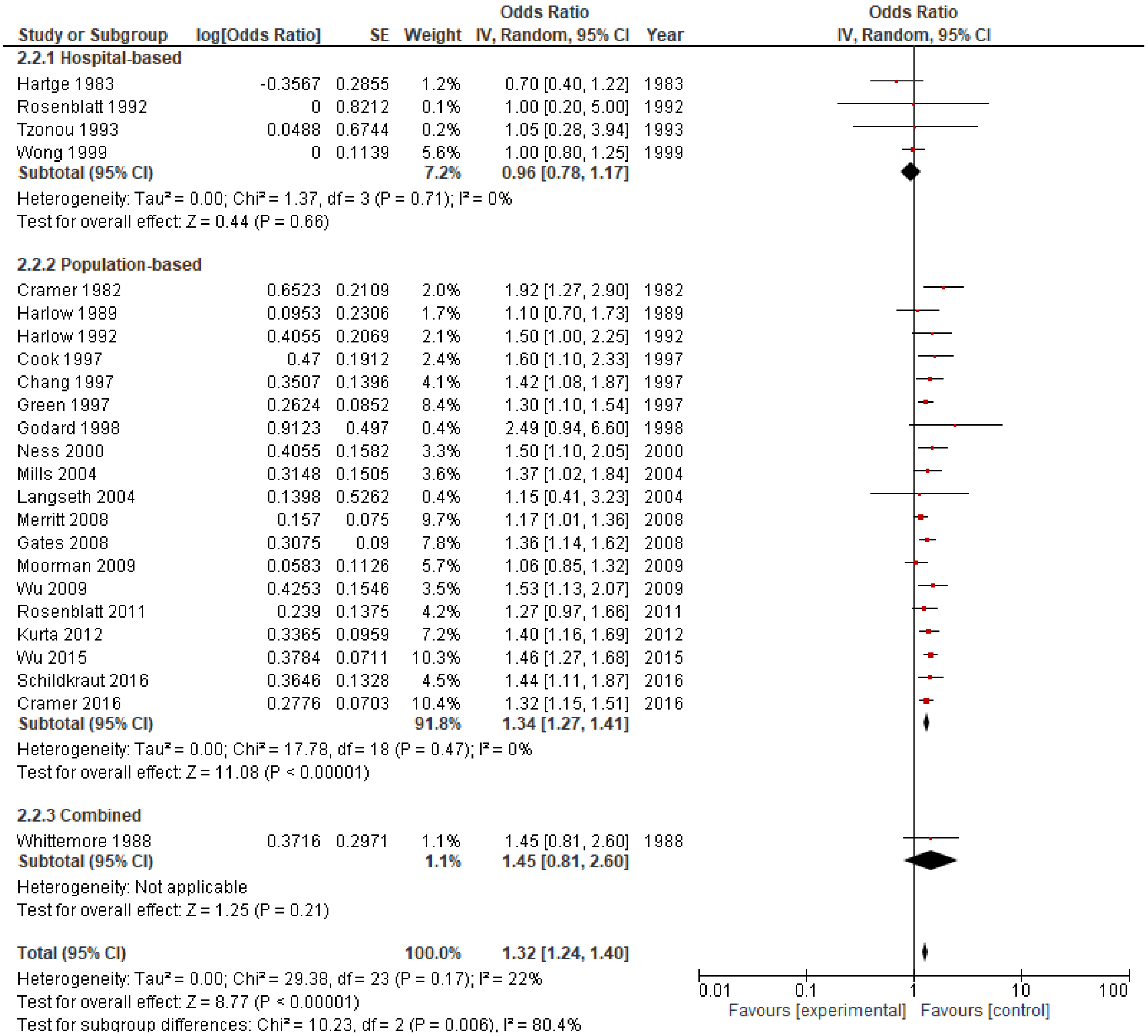

Perineal talc use and ovarian cancer, by score of the New Castle Ottawa Scale (NOS)

Study or Subgroup	log[Odds Ratio]	SE	Weight	Odds Ratio IV, Random, 95% CI	Year
**2.3.1 NOS >=7**					
Harlow 1989	0.0953	0.2306	1.7%	1.10 [0.70, 1.73]	1989
Harlow 1992	0.4055	0.2069	2.1%	1.50 [1.00, 2.25]	1992
Cook 1997	0.47	0.1912	2.4%	1.60 [1.10, 2.33]	1997
Green 1997	0.2624	0.0852	7.0%	1.30 [1.10, 1.54]	1997
Chang 1997	0.3507	0.1396	3.9%	1.42 [1.08, 1.87]	1997
Merritt 2008	0.157	0.075	7.8%	1.17 [1.01, 1.36]	2008
Gates 2008	0.3075	0.09	6.6%	1.36 [1.14, 1.62]	2008
Wu 2009	0.4253	0.1546	3.3%	1.53 [1.13, 2.07]	2009
Rosenblatt 2011	0.239	0.1375	4.0%	1.27 [0.97, 1.66]	2011
Houghton 2014	0.1133	0.1004	5.9%	1.12 [0.92, 1.36]	2014
Wu 2015	0.3784	0.0711	8.1%	1.46 [1.27, 1.68]	2015
Schildkraut 2016	0.3646	0.1328	4.2%	1.44 [1.11, 1.87]	2016
Cramer 2016	0.2776	0.0703	8.2%	1.32 [1.15, 1.51]	2016
**Subtotal (95% CI)**			**65.2%**	**1.32 [1.25, 1.40]**	
Heterogeneity: Tau² = 0.00; Chi² = 11.13, df = 12 (P = 0.52); I² = 0%					
Test for overall effect: Z = 9.70 (P < 0.00001)					
**2.3.2 NOS <7**					
Cramer 1982	0.6523	0.2109	2.0%	1.92 [1.27, 2.90]	1982
Hartge 1983	-0.3567	0.2855	1.2%	0.70 [0.40, 1.22]	1983
Whittemore 1988	0.3716	0.2971	1.1%	1.45 [0.81, 2.60]	1988
Rosenblatt 1992	0	0.8212	0.2%	1.00 [0.20, 5.00]	1992
Tzonou 1993	0.0488	0.6744	0.2%	1.05 [0.28, 3.94]	1993
Godard 1998	0.9123	0.497	0.4%	2.49 [0.94, 6.60]	1998
Wong 1999	0	0.1139	5.1%	1.00 [0.80, 1.25]	1999
Gertig 2000	0.0862	0.1209	4.7%	1.09 [0.86, 1.38]	2000
Ness 2000	0.4055	0.1582	3.2%	1.50 [1.10, 2.05]	2000
Langseth 2004	0.1398	0.5262	0.4%	1.15 [0.41, 3.23]	2004
Mills 2004	0.3148	0.1505	3.5%	1.37 [1.02, 1.84]	2004
Moorman 2009	0.0583	0.1126	5.2%	1.06 [0.85, 1.32]	2009
Kurta 2012	0.3365	0.0959	6.2%	1.40 [1.16, 1.69]	2012
Gonzalez 2016	-0.3147	0.2583	1.4%	0.73 [0.44, 1.21]	2016
**Subtotal (95% CI)**			**34.8%**	**1.21 [1.05, 1.39]**	
Heterogeneity: Tau² = 0.03; Chi² = 24.64, df = 13 (P = 0.03); I² = 47%					
Test for overall effect: Z = 2.61 (P = 0.009)					
**Total (95% CI)**			**100.0%**	**1.28 [1.20, 1.37]**	
Heterogeneity: Tau² = 0.01; Chi² = 38.74, df = 26 (P = 0.05); I² = 33%					
Test for overall effect: Z = 7.65 (P < 0.00001)					
Test for subgroup differences: Chi² = 1.36, df = 1 (P = 0.24), I² = 26.3%					

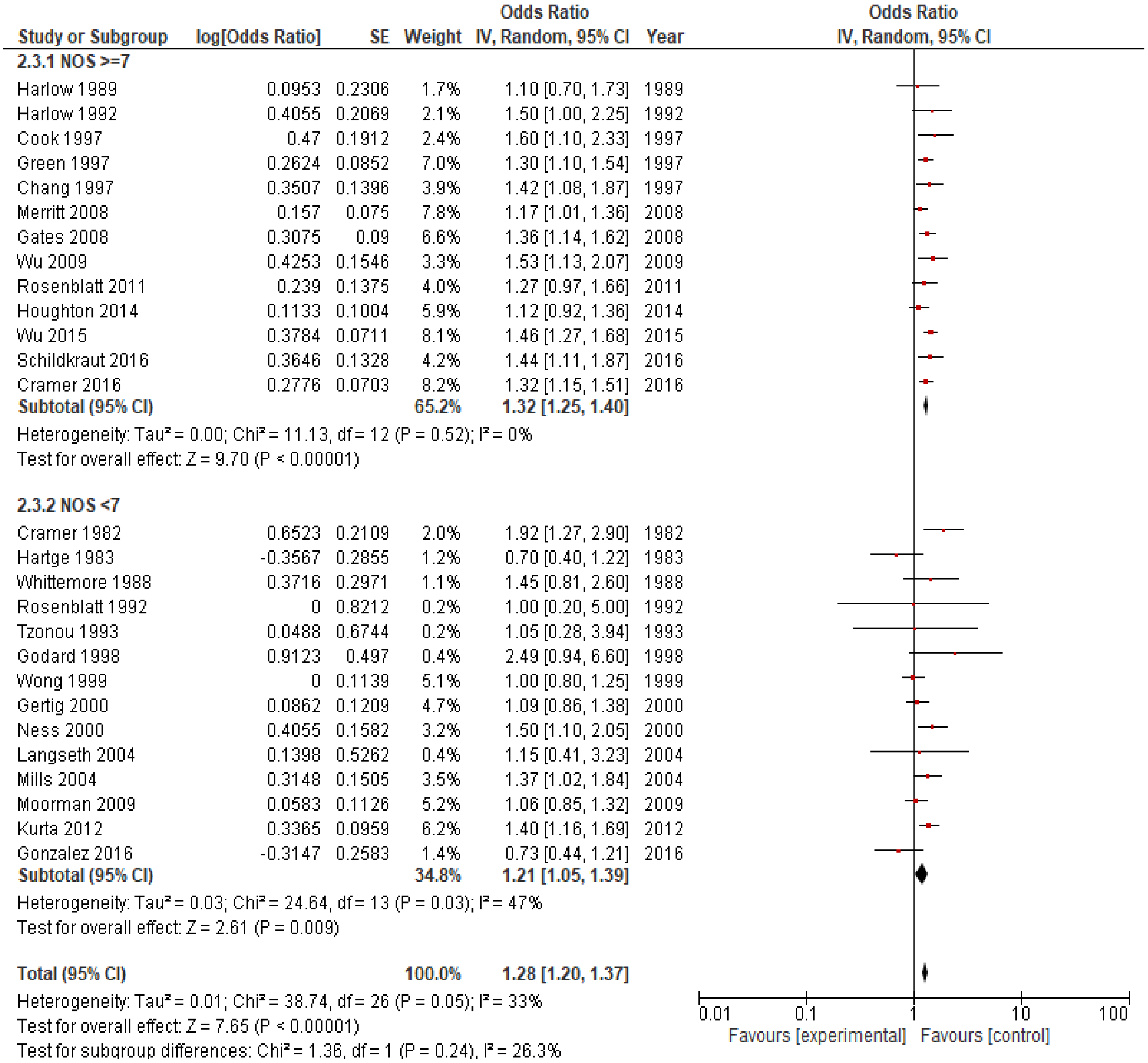

Perineal talc use (ever vs. never) and ovarian cancer, by the study's year of publication

Study or Subgroup	log[Odds Ratio]	SE	Weight	Odds Ratio IV, Random, 95% CI	Year
**2.4.1 1980-1989**					
Cramer 1982	0.6523	0.2109	2.0%	1.92 [1.27, 2.90]	1982
Hartge 1983	-0.3567	0.2855	1.2%	0.70 [0.40, 1.22]	1983
Whittemore 1988	0.3716	0.2971	1.1%	1.45 [0.81, 2.60]	1988
Harlow 1989	0.0953	0.2306	1.7%	1.10 [0.70, 1.73]	1989
**Subtotal (95% CI)**			**6.1%**	**1.23 [0.81, 1.88]**	
Heterogeneity: Tau² = 0.12; Chi² = 8.77, df = 3 (P = 0.03); I² = 66%					
Test for overall effect: Z = 0.97 (P = 0.33)					
**2.4.2 1990-1999**					
Harlow 1992	0.4055	0.2069	2.1%	1.50 [1.00, 2.25]	1992
Rosenblatt 1992	0	0.8212	0.2%	1.00 [0.20, 5.00]	1992
Tzonou 1993	0.0488	0.6744	0.2%	1.05 [0.28, 3.94]	1993
Chang 1997	0.3507	0.1396	3.9%	1.42 [1.08, 1.87]	1997
Green 1997	0.2624	0.0852	7.0%	1.30 [1.10, 1.54]	1997
Cook 1997	0.47	0.1912	2.4%	1.60 [1.10, 2.33]	1997
Godard 1998	0.9123	0.497	0.4%	2.49 [0.94, 6.60]	1998
Wong 1999	0	0.1139	5.1%	1.00 [0.80, 1.25]	1999
**Subtotal (95% CI)**			**21.2%**	**1.30 [1.13, 1.50]**	
Heterogeneity: Tau² = 0.01; Chi² = 9.20, df = 7 (P = 0.24); I² = 24%					
Test for overall effect: Z = 3.62 (P = 0.0003)					
**2.4.3 2000-2009**					
Gertig 2000	0.0862	0.1209	4.7%	1.09 [0.86, 1.38]	2000
Ness 2000	0.4055	0.1582	3.2%	1.50 [1.10, 2.05]	2000
Langseth 2004	0.1398	0.5262	0.4%	1.15 [0.41, 3.23]	2004
Mills 2004	0.3148	0.1505	3.5%	1.37 [1.02, 1.84]	2004
Gates 2008	0.3075	0.09	6.6%	1.36 [1.14, 1.62]	2008
Merritt 2008	0.157	0.075	7.8%	1.17 [1.01, 1.36]	2008
Wu 2009	0.4253	0.1546	3.3%	1.53 [1.13, 2.07]	2009
Moorman 2009	0.0583	0.1126	5.2%	1.06 [0.85, 1.32]	2009
**Subtotal (95% CI)**			**34.7%**	**1.25 [1.14, 1.37]**	
Heterogeneity: Tau² = 0.00; Chi² = 8.49, df = 7 (P = 0.29); I² = 18%					
Test for overall effect: Z = 4.62 (P < 0.00001)					
**2.4.4 2010 and beyond**					
Rosenblatt 2011	0.239	0.1375	4.0%	1.27 [0.97, 1.66]	2011
Kurta 2012	0.3365	0.0959	6.2%	1.40 [1.16, 1.69]	2012
Houghton 2014	0.1133	0.1004	5.9%	1.12 [0.92, 1.36]	2014
Wu 2015	0.3784	0.0711	8.1%	1.46 [1.27, 1.68]	2015
Cramer 2016	0.2776	0.0703	8.2%	1.32 [1.15, 1.51]	2016
Gonzalez 2016	-0.3147	0.2583	1.4%	0.73 [0.44, 1.21]	2016
Schildkraut 2016	0.3646	0.1328	4.2%	1.44 [1.11, 1.87]	2016
**Subtotal (95% CI)**			**38.0%**	**1.31 [1.18, 1.45]**	
Heterogeneity: Tau² = 0.01; Chi² = 10.81, df = 6 (P = 0.09); I² = 44%					
Test for overall effect: Z = 5.00 (P < 0.00001)					
**Total (95% CI)**			**100.0%**	**1.28 [1.20, 1.37]**	
Heterogeneity: Tau² = 0.01; Chi² = 38.74, df = 26 (P = 0.05); I² = 33%					
Test for overall effect: Z = 7.65 (P < 0.00001)					
Test for subgroup differences: Chi² = 0.51, df = 3 (P = 0.92), I² = 0%					

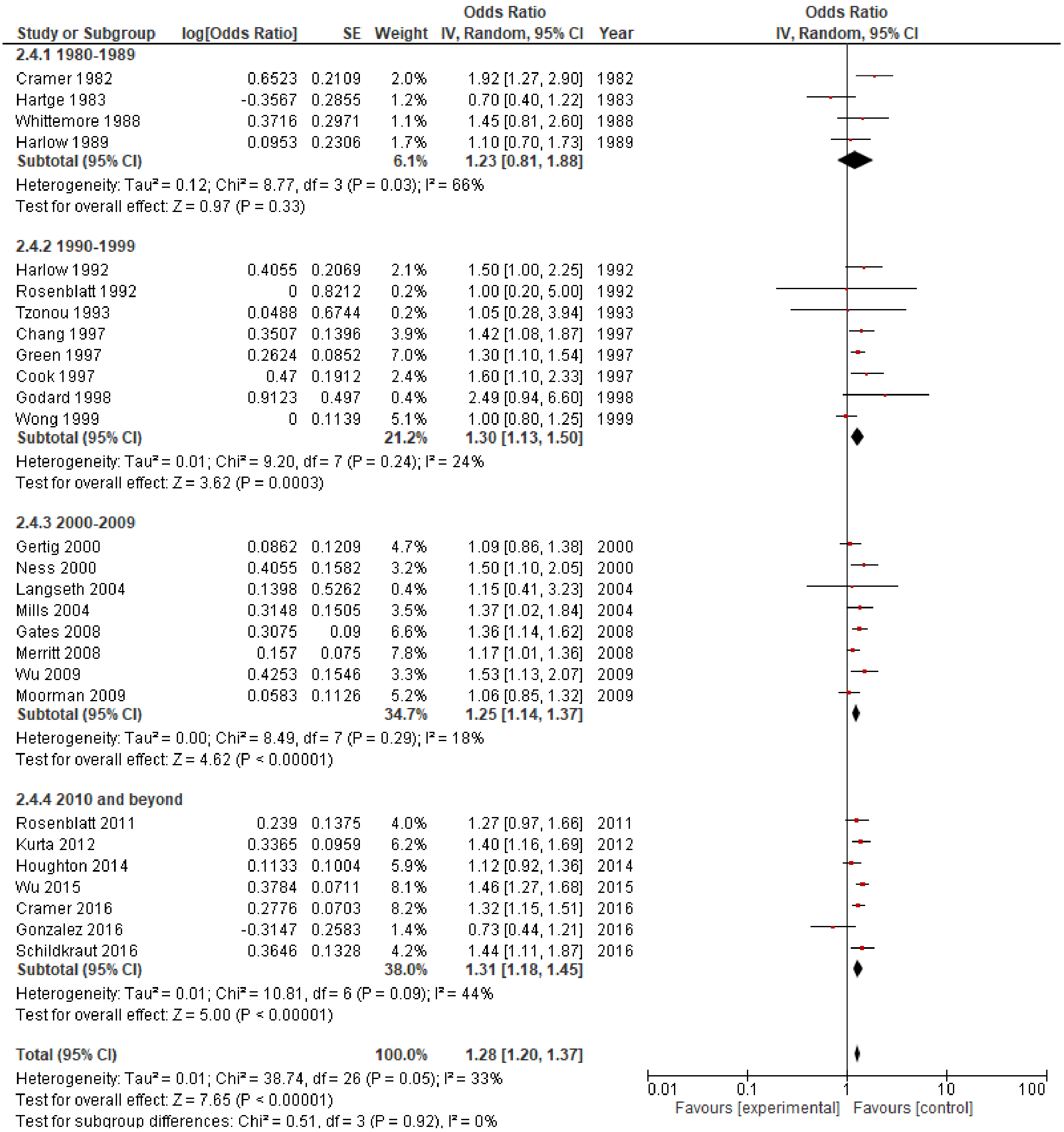

Perineal talc use – assessment of talc exposure

Perineal talc use and ovarian cancer, by frequency of use

Study or Subgroup	log[Odds Ratio]	SE	Weight	Odds Ratio IV, Random, 95% CI	Year
**3.1.1 Low**					
Harlow 1992	0.4055	0.3207	2.5%	1.50 [0.80, 2.81]	1992
Chang 1997	0.6076	0.2002	5.6%	1.84 [1.24, 2.72]	1997
Gertig 2000	0.131	0.1744	6.8%	1.14 [0.81, 1.60]	2000
Gates 2008	-0.1985	0.2038	5.4%	0.82 [0.55, 1.22]	2008
Cramer 2016	0.157	0.1009	12.6%	1.17 [0.96, 1.43]	2016
**Subtotal (95% CI)**			**32.9%**	**1.22 [0.96, 1.54]**	
Heterogeneity: Tau² = 0.04; Chi² = 8.64, df = 4 (P = 0.07); I² = 54%					
Test for overall effect: Z = 1.63 (P = 0.10)					
**3.1.2 Medium**					
Chang 1997	0.1204	0.2151	5.0%	1.13 [0.74, 1.72]	1997
Gates 2008	0.2311	0.1335	9.5%	1.26 [0.97, 1.64]	2008
**Subtotal (95% CI)**			**14.5%**	**1.22 [0.98, 1.53]**	
Heterogeneity: Tau² = 0.00; Chi² = 0.19, df = 1 (P = 0.66); I² = 0%					
Test for overall effect: Z = 1.77 (P = 0.08)					
**3.1.3 High**					
Booth 1989	0.2624	0.2477	4.0%	1.30 [0.80, 2.11]	1989
Harlow 1992	0.5878	0.2513	3.9%	1.80 [1.10, 2.95]	1992
Chang 1997	-0.0502	0.2266	4.6%	0.95 [0.61, 1.48]	1997
Gertig 2000	0.1133	0.1591	7.7%	1.12 [0.82, 1.53]	2000
Gates 2008	0.3436	0.1085	11.8%	1.41 [1.14, 1.74]	2008
Schildkraut 2016	0.5365	0.1558	7.9%	1.71 [1.26, 2.32]	2016
Cramer 2016	0.3784	0.1001	12.7%	1.46 [1.20, 1.78]	2016
**Subtotal (95% CI)**			**52.6%**	**1.39 [1.22, 1.58]**	
Heterogeneity: Tau² = 0.01; Chi² = 7.80, df = 6 (P = 0.25); I² = 23%					
Test for overall effect: Z = 4.86 (P < 0.00001)					
**Total (95% CI)**			**100.0%**	**1.30 [1.17, 1.44]**	
Heterogeneity: Tau² = 0.01; Chi² = 19.88, df = 13 (P = 0.10); I² = 35%					
Test for overall effect: Z = 4.80 (P < 0.00001)					
Test for subgroup differences: Chi² = 1.48, df = 2 (P = 0.48), I² = 0%					

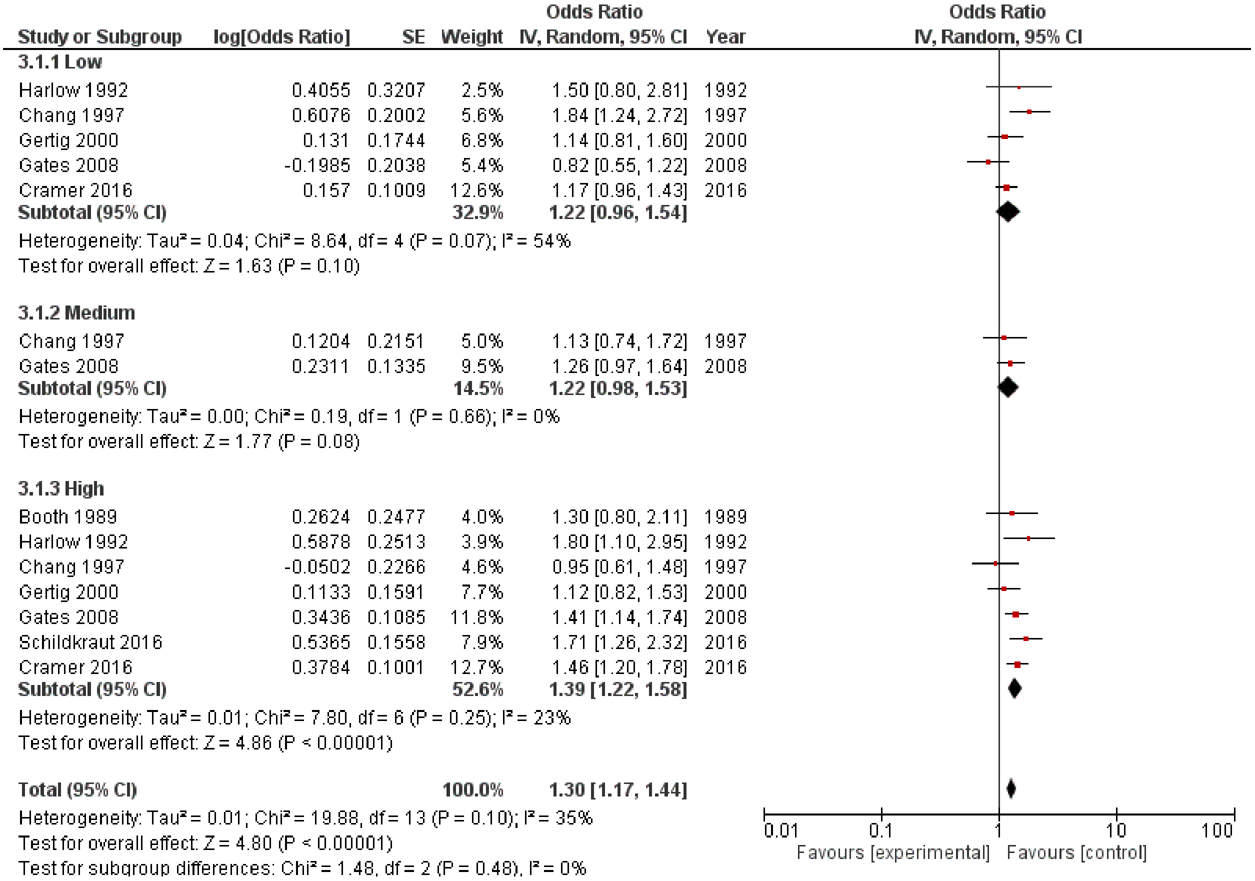

Low: *Once daily for 1 – <10 days/month;* ***Medium:*** *Once daily for 10 –25 days/month;* ***High:*** *Once daily for >25 days/month*

Perineal talc use and ovarian cancer, by duration of use

Study or Subgroup	log[Odds Ratio]	SE	Weight	Odds Ratio IV, Random, 95% CI	Year
**3.2.1 <10 Years**					
Whittemore 1988	0.47	0.2398	7.8%	1.60 [1.00, 2.56]	1988
Harlow 1992	0.1823	0.4467	2.3%	1.20 [0.50, 2.88]	1992
Wong 1999	-0.1054	0.2069	10.4%	0.90 [0.60, 1.35]	1999
Rosenblatt 2011	0.3293	0.2509	7.1%	1.39 [0.85, 2.27]	2011
Houghton 2014	0.207	0.1159	30.0%	1.23 [0.98, 1.54]	2014
**Subtotal (95% CI)**			**57.7%**	**1.22 [1.03, 1.45]**	
Heterogeneity: Tau² = 0.00; Chi² = 3.72, df = 4 (P = 0.45); I² = 0%					
Test for overall effect: Z = 2.34 (P = 0.02)					
**3.2.2 10 - <20 Years**					
Wong 1999	0.3365	0.2254	8.8%	1.40 [0.90, 2.18]	1999
Rosenblatt 2011	0.3784	0.2641	6.5%	1.46 [0.87, 2.45]	2011
**Subtotal (95% CI)**			**15.3%**	**1.42 [1.02, 1.99]**	
Heterogeneity: Tau² = 0.00; Chi² = 0.01, df = 1 (P = 0.90); I² = 0%					
Test for overall effect: Z = 2.07 (P = 0.04)					
**3.2.3 20+ Years**					
Wong 1999	-0.1054	0.2069	10.4%	0.90 [0.60, 1.35]	1999
Schildkraut 2016	0.4187	0.1604	16.7%	1.52 [1.11, 2.08]	2016
**Subtotal (95% CI)**			**27.1%**	**1.19 [0.71, 1.98]**	
Heterogeneity: Tau² = 0.10; Chi² = 4.01, df = 1 (P = 0.05); I² = 75%					
Test for overall effect: Z = 0.66 (P = 0.51)					
**Total (95% CI)**			**100.0%**	**1.26 [1.10, 1.44]**	
Heterogeneity: Tau² = 0.00; Chi² = 8.39, df = 8 (P = 0.40); I² = 5%					
Test for overall effect: Z = 3.36 (P = 0.0008)					
Test for subgroup differences: Chi² = 0.69, df = 2 (P = 0.71), I² = 0%					

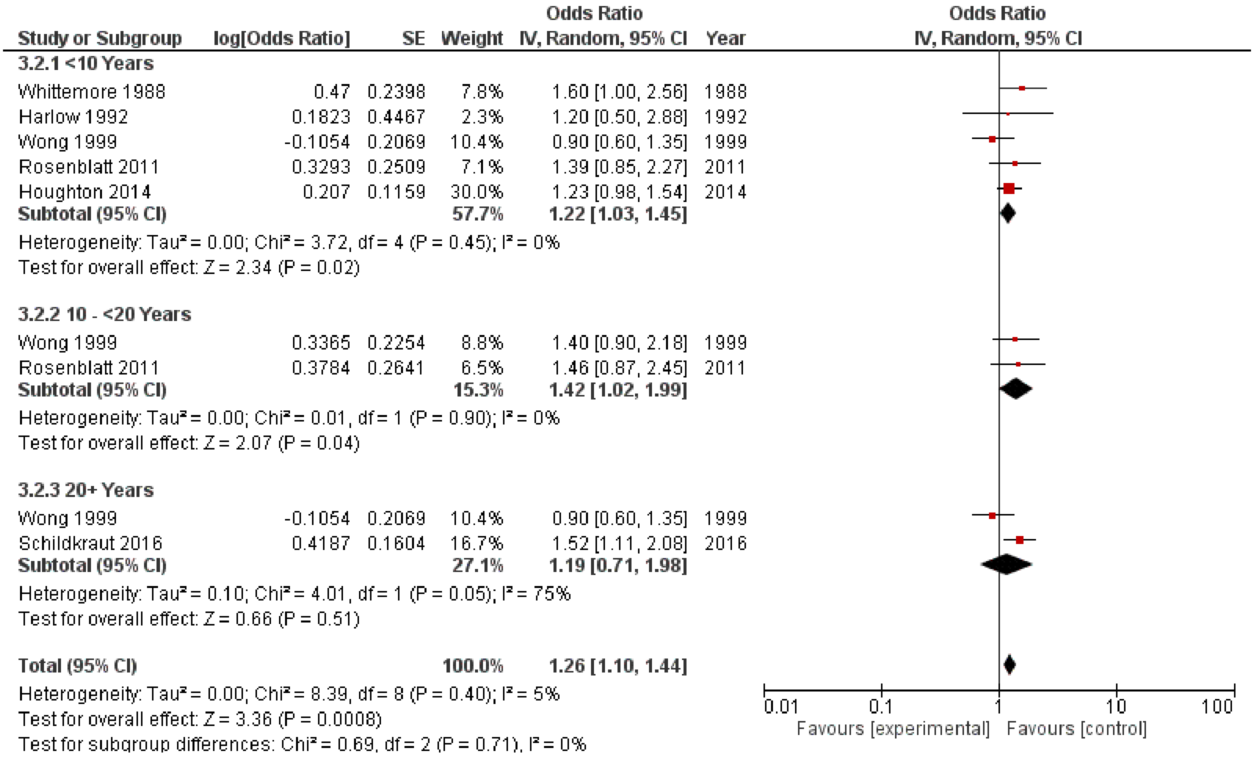

Perineal talc use and ovarian cancer, by type of use

Study or Subgroup	log[Odds Ratio]	SE	Weight	Odds Ratio IV, Random, 95% CI	Year
**3.3.1 Sanitary Napkin**					
Whittemore 1988	-0.478	0.5524	1.5%	0.62 [0.21, 1.83]	1988
Harlow 1989	0.7885	0.5161	1.6%	2.20 [0.80, 6.05]	1989
Rosenblatt 1992	1.5686	0.6665	1.1%	4.80 [1.30, 17.72]	1992
Chang 1997	0.2327	0.2262	4.8%	1.26 [0.81, 1.97]	1997
Cook 1997	-0.1054	0.2999	3.6%	0.90 [0.50, 1.62]	1997
Wong 1999	-0.1054	0.4137	2.3%	0.90 [0.40, 2.02]	1999
Gertig 2000	-0.1165	0.1927	5.5%	0.89 [0.61, 1.30]	2000
Ness 2000	0.47	0.1912	5.5%	1.60 [1.10, 2.33]	2000
Wu 2009	0.4762	0.28	3.9%	1.61 [0.93, 2.79]	2009
Rosenblatt 2011	-0.1985	0.1767	5.9%	0.82 [0.58, 1.16]	2011
Houghton 2014	-0.0513	0.1139	7.3%	0.95 [0.76, 1.19]	2014
**Subtotal (95% CI)**			**43.0%**	**1.12 [0.91, 1.39]**	
Heterogeneity: Tau^2 = 0.05; Chi^2 = 19.85, df = 10 (P = 0.03); I^2 = 50%					
Test for overall effect: Z = 1.05 (P = 0.29)					
**3.3.2 Diaphragm**					
Cramer 1982	0.174	0.2781	3.9%	1.19 [0.69, 2.05]	1982
Whittemore 1988	0.4055	0.4426	2.1%	1.50 [0.63, 3.57]	1988
Harlow 1989	-0.6931	0.4675	1.9%	0.50 [0.20, 1.25]	1989
Rosenblatt 1992	1.0986	0.6744	1.0%	3.00 [0.80, 11.25]	1992
Cook 1997	0	0.2606	4.2%	1.00 [0.60, 1.67]	1997
Ness 2000	-0.5108	0.3537	2.9%	0.60 [0.30, 1.20]	2000
Wu 2009	0.131	0.463	1.9%	1.14 [0.46, 2.82]	2009
Rosenblatt 2011	-0.3285	0.2069	5.2%	0.72 [0.48, 1.08]	2011
Houghton 2014	-0.0834	0.1542	6.4%	0.92 [0.68, 1.24]	2014
Cramer 2016	-0.3147	0.1262	7.1%	0.73 [0.57, 0.93]	2016
**Subtotal (95% CI)**			**36.6%**	**0.87 [0.72, 1.05]**	
Heterogeneity: Tau^2 = 0.02; Chi^2 = 12.00, df = 9 (P = 0.21); I^2 = 25%					
Test for overall effect: Z = 1.48 (P = 0.14)					
**3.3.3 Underwear**					
Ness 2000	0.5306	0.1777	5.8%	1.70 [1.20, 2.41]	2000
Wu 2009	0.5365	0.2789	3.9%	1.71 [0.99, 2.95]	2009
**Subtotal (95% CI)**			**9.7%**	**1.70 [1.27, 2.28]**	
Heterogeneity: Tau^2 = 0.00; Chi^2 = 0.00, df = 1 (P = 0.99); I^2 = 0%					
Test for overall effect: Z = 3.55 (P = 0.0004)					
**3.3.4 Male Condom**					
Cramer 1982	-0.2614	0.3216	3.3%	0.77 [0.41, 1.45]	1982
Rosenblatt 1992	0.47	0.5004	1.7%	1.60 [0.60, 4.27]	1992
Ness 2000	0	0.182	5.7%	1.00 [0.70, 1.43]	2000
**Subtotal (95% CI)**			**10.7%**	**0.99 [0.73, 1.32]**	
Heterogeneity: Tau^2 = 0.00; Chi^2 = 1.53, df = 2 (P = 0.46); I^2 = 0%					
Test for overall effect: Z = 0.10 (P = 0.92)					
**Total (95% CI)**			**100.0%**	**1.06 [0.92, 1.22]**	
Heterogeneity: Tau^2 = 0.06; Chi^2 = 52.01, df = 25 (P = 0.001); I^2 = 52%					
Test for overall effect: Z = 0.79 (P = 0.43)					
Test for subgroup differences: Chi^2 = 14.85, df = 3 (P = 0.002), I^2 = 79.8%					

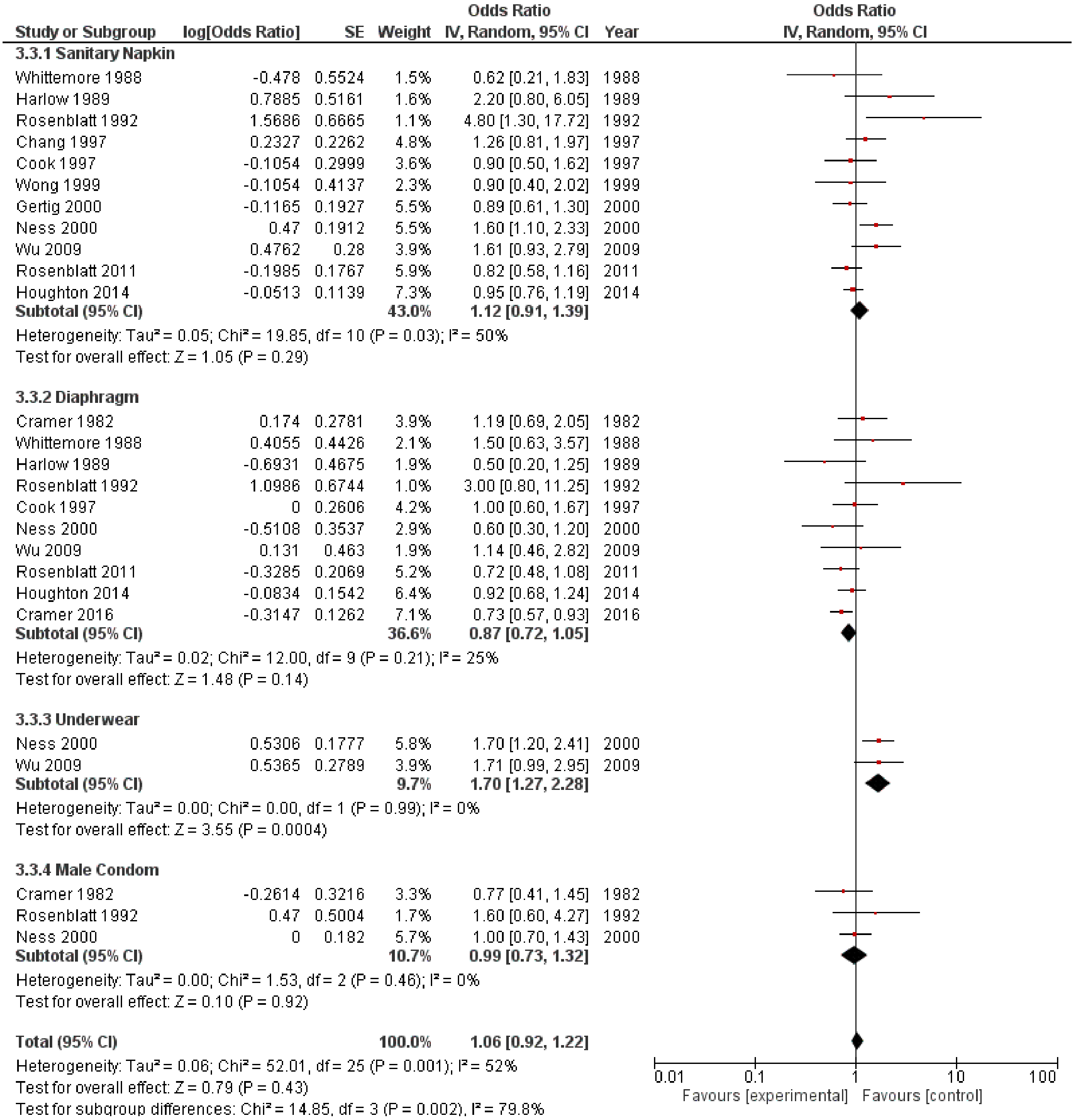

Perineal talc use – tumor histology

Perineal talc use and ovarian cancer, by tumor histology

Study or Subgroup	log[Odds Ratio]	SE	Weight	Odds Ratio IV, Random, 95% CI
**4.1.1 Serous**				
Chang 1997	0.2897	0.1686	7.2%	1.34 [0.96, 1.86]
Cook 1997	0.5306	0.2221	4.8%	1.70 [1.10, 2.63]
Gertig 2000	0.2311	0.1495	8.5%	1.26 [0.94, 1.69]
Harlow 1992	0.3365	0.2254	4.7%	1.40 [0.90, 2.18]
Houghton 2014	0.1484	0.1409	9.1%	1.16 [0.88, 1.53]
Schildkraut 2016	0.3221	0.1493	8.5%	1.38 [1.03, 1.85]
Wu 2015	0.5306	0.1488	8.5%	1.70 [1.27, 2.28]
**Subtotal (95% CI)**			**51.2%**	**1.38 [1.22, 1.56]**
Heterogeneity: Tau² = 0.00; Chi² = 4.78, df = 6 (P = 0.57); I² = 0%				
Test for overall effect: Z = 5.19 (P < 0.00001)				
**4.1.2 Mucinous**				
Chang 1997	0.4606	0.2505	3.9%	1.59 [0.97, 2.59]
Cook 1997	-0.3567	0.2855	3.1%	0.70 [0.40, 1.22]
Gates 2010	0.4055	0.2958	3.0%	1.50 [0.84, 2.68]
Gertig 2000	-0.0726	0.2869	3.1%	0.93 [0.53, 1.63]
Harlow 1992	0.1823	0.3537	2.1%	1.20 [0.60, 2.40]
Houghton 2014	0.0296	0.0861	14.8%	1.03 [0.87, 1.22]
**Subtotal (95% CI)**			**30.1%**	**1.09 [0.89, 1.33]**
Heterogeneity: Tau² = 0.02; Chi² = 6.51, df = 5 (P = 0.26); I² = 23%				
Test for overall effect: Z = 0.83 (P = 0.41)				
**4.1.3 Endometrioid**				
Chang 1997	0.5134	0.262	3.6%	1.67 [1.00, 2.79]
Cook 1997	0.1823	0.3537	2.1%	1.20 [0.60, 2.40]
Gates 2010	0.0583	0.2417	4.2%	1.06 [0.66, 1.70]
Gertig 2000	-0.0943	0.3158	2.6%	0.91 [0.49, 1.69]
Harlow 1992	1.0296	0.4323	1.5%	2.80 [1.20, 6.53]
Houghton 2014	0.2546	0.3576	2.1%	1.29 [0.64, 2.60]
Mills 2004	0.2469	0.3699	2.0%	1.28 [0.62, 2.64]
**Subtotal (95% CI)**			**18.2%**	**1.30 [1.03, 1.65]**
Heterogeneity: Tau² = 0.00; Chi² = 6.11, df = 6 (P = 0.41); I² = 2%				
Test for overall effect: Z = 2.17 (P = 0.03)				
**4.1.4 Clear Cell**				
Mills 2004	-0.462	0.7322	0.5%	0.63 [0.15, 2.65]
**Subtotal (95% CI)**			**0.5%**	**0.63 [0.15, 2.65]**
Heterogeneity: Not applicable				
Test for overall effect: Z = 0.63 (P = 0.53)				
**4.1.5 Undifferentiated**				
**Subtotal (95% CI)**				**Not estimable**
Heterogeneity: Not applicable				
Test for overall effect: Not applicable				
**Total (95% CI)**			**100.0%**	**1.27 [1.14, 1.41]**
Heterogeneity: Tau² = 0.01; Chi² = 25.90, df = 20 (P = 0.17); I² = 23%				
Test for overall effect: Z = 4.38 (P < 0.0001)				
Test for subgroup differences: Chi² = 4.83, df = 3 (P = 0.18), I² = 37.9%				

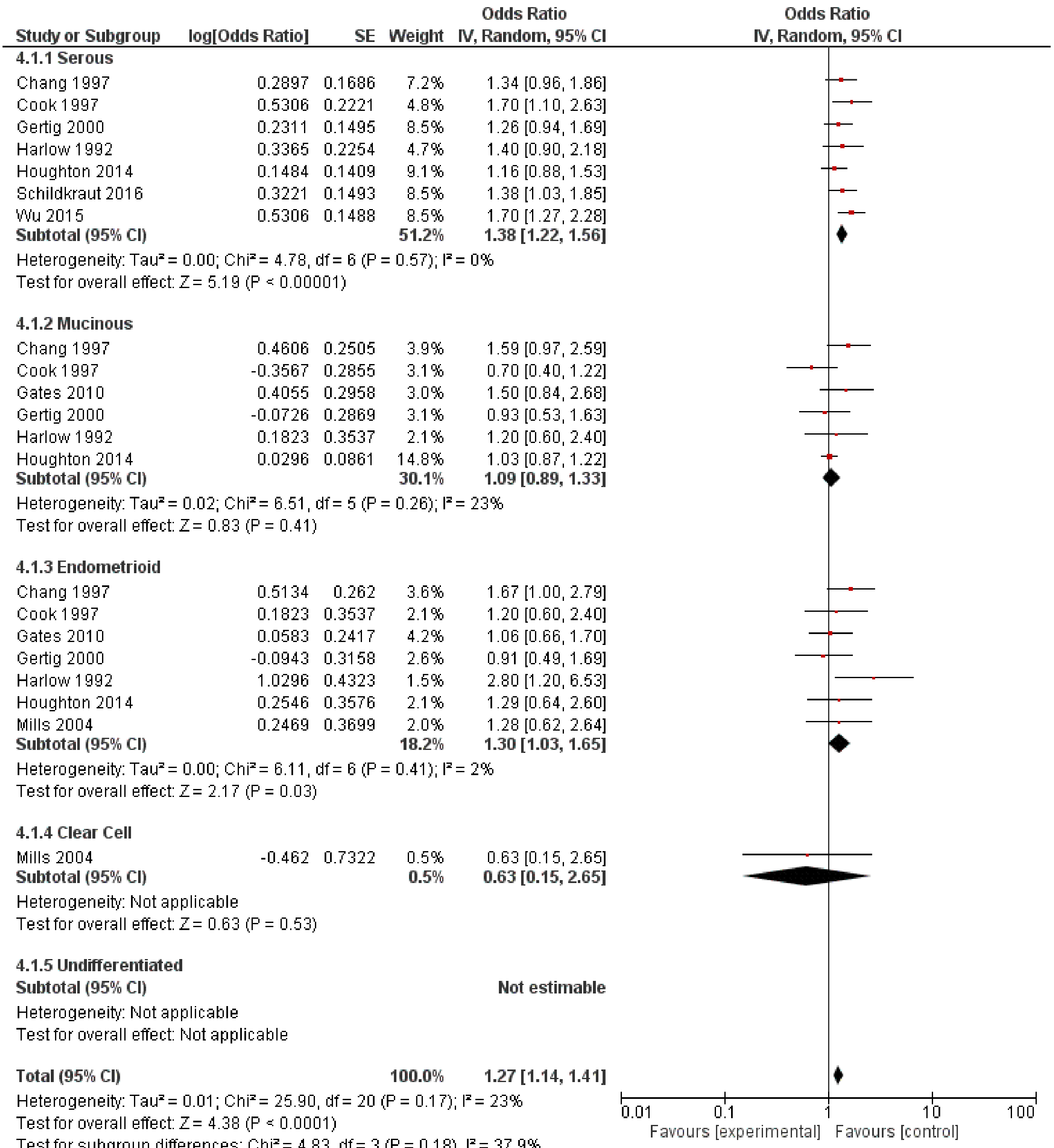

Perineal talc use– tumor behavior

Perineal talc use and ovarian cancer, by overall tumor behavior

Study or Subgroup	log[Odds Ratio]	SE	Weight	Odds Ratio IV, Random, 95% CI	Year
**5.1.1 All Invasive**					
Chang 1997	0.4141	0.1489	24.8%	1.51 [1.13, 2.03]	1997
Mills 2004	0.4121	0.1757	17.8%	1.51 [1.07, 2.13]	2004
Rosenblatt 2011	0.157	0.1512	24.1%	1.17 [0.87, 1.57]	2011
**Subtotal (95% CI)**			**66.7%**	**1.38 [1.15, 1.65]**	
Heterogeneity: Tau² = 0.00; Chi² = 1.84, df = 2 (P = 0.40); I² = 0%					
Test for overall effect: Z = 3.53 (P = 0.0004)					
**5.1.2 All Borderline**					
Harlow 1992	0.8755	0.3537	4.4%	2.40 [1.20, 4.80]	1992
Chang 1997	0.2127	0.2485	8.9%	1.24 [0.76, 2.01]	1997
Mills 2004	0.0862	0.2638	7.9%	1.09 [0.65, 1.83]	2004
Rosenblatt 2011	0.4383	0.2135	12.1%	1.55 [1.02, 2.36]	2011
**Subtotal (95% CI)**			**33.3%**	**1.43 [1.08, 1.89]**	
Heterogeneity: Tau² = 0.02; Chi² = 3.68, df = 3 (P = 0.30); I² = 19%					
Test for overall effect: Z = 2.47 (P = 0.01)					
**Total (95% CI)**			**100.0%**	**1.39 [1.20, 1.61]**	
Heterogeneity: Tau² = 0.00; Chi² = 5.56, df = 6 (P = 0.47); I² = 0%					
Test for overall effect: Z = 4.46 (P < 0.00001)					
Test for subgroup differences: Chi² = 0.04, df = 1 (P = 0.83), I² = 0%					

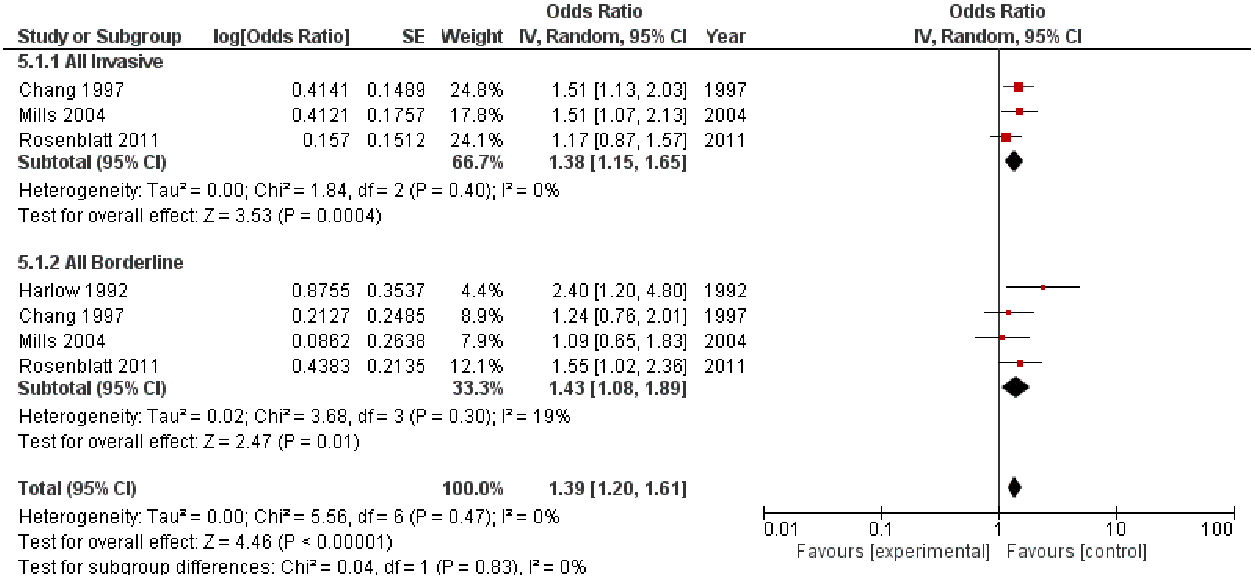

Perineal talc use and ovarian cancer, for serous ovarian tumors

Study or Subgroup	log[Odds Ratio]	SE	Weight	Odds Ratio IV, Random, 95% CI	Year
**5.2.1 Serous Invasive**					
Gertig 2000	0.3365	0.1616	12.2%	1.40 [1.02, 1.92]	2000
Mills 2004	0.571	0.2335	5.8%	1.77 [1.12, 2.80]	2004
Rosenblatt 2011	0.01	0.1944	8.4%	1.01 [0.69, 1.48]	2011
Houghton 2014	0.1222	0.1513	13.9%	1.13 [0.84, 1.52]	2014
Cramer 2016	0.3507	0.0902	39.2%	1.42 [1.19, 1.69]	2016
**Subtotal (95% CI)**			**79.6%**	**1.32 [1.13, 1.54]**	
Heterogeneity: Tau² = 0.01; Chi² = 5.29, df = 4 (P = 0.26); I² = 24%					
Test for overall effect: Z = 3.57 (P = 0.0004)					
**5.2.2 Serous Borderline**					
Mills 2004	0.2469	0.3007	3.5%	1.28 [0.71, 2.31]	2004
Rosenblatt 2011	0.3853	0.2855	3.9%	1.47 [0.84, 2.57]	2011
Cramer 2016	0.3365	0.1566	13.0%	1.40 [1.03, 1.90]	2016
**Subtotal (95% CI)**			**20.4%**	**1.39 [1.09, 1.78]**	
Heterogeneity: Tau² = 0.00; Chi² = 0.12, df = 2 (P = 0.94); I² = 0%					
Test for overall effect: Z = 2.65 (P = 0.008)					
**Total (95% CI)**			**100.0%**	**1.35 [1.21, 1.50]**	
Heterogeneity: Tau² = 0.00; Chi² = 5.49, df = 7 (P = 0.60); I² = 0%					
Test for overall effect: Z = 5.26 (P < 0.00001)					
Test for subgroup differences: Chi² = 0.12, df = 1 (P = 0.72), I² = 0%					

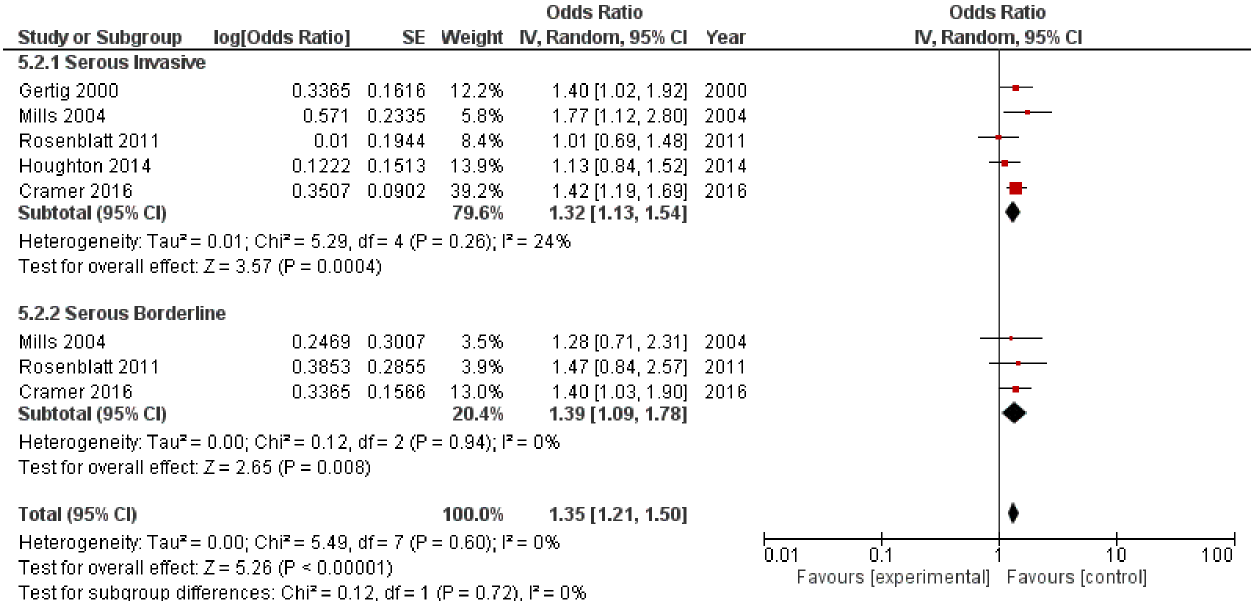

Perineal talc use and ovarian cancer, for mucinous ovarian tumors

Study or Subgroup	log[Odds Ratio]	SE	Weight	Odds Ratio IV, Random, 95% CI	Year
**5.3.1 Mucinous Invasive**					
Mills 2004	0.94	0.5391	9.5%	2.56 [0.89, 7.36]	2004
Cramer 2016	-0.1393	0.2529	26.8%	0.87 [0.53, 1.43]	2016
**Subtotal (95% CI)**			**36.4%**	**1.34 [0.48, 3.79]**	
Heterogeneity: Tau² = 0.41; Chi² = 3.29, df = 1 (P = 0.07); I² = 70%					
Test for overall effect: Z = 0.56 (P = 0.58)					
**5.3.2 Mucinous Borderline**					
Mills 2004	-0.2744	0.5095	10.5%	0.76 [0.28, 2.06]	2004
Rosenblatt 2011	0.5766	0.3045	21.8%	1.78 [0.98, 3.23]	2011
Cramer 2016	0.0198	0.2144	31.4%	1.02 [0.67, 1.55]	2016
**Subtotal (95% CI)**			**63.6%**	**1.18 [0.76, 1.82]**	
Heterogeneity: Tau² = 0.05; Chi² = 3.02, df = 2 (P = 0.22); I² = 34%					
Test for overall effect: Z = 0.73 (P = 0.46)					
**Total (95% CI)**			**100.0%**	**1.17 [0.82, 1.67]**	
Heterogeneity: Tau² = 0.06; Chi² = 6.43, df = 4 (P = 0.17); I² = 38%					
Test for overall effect: Z = 0.85 (P = 0.40)					
Test for subgroup differences: Chi² = 0.05, df = 1 (P = 0.82), I² = 0%					

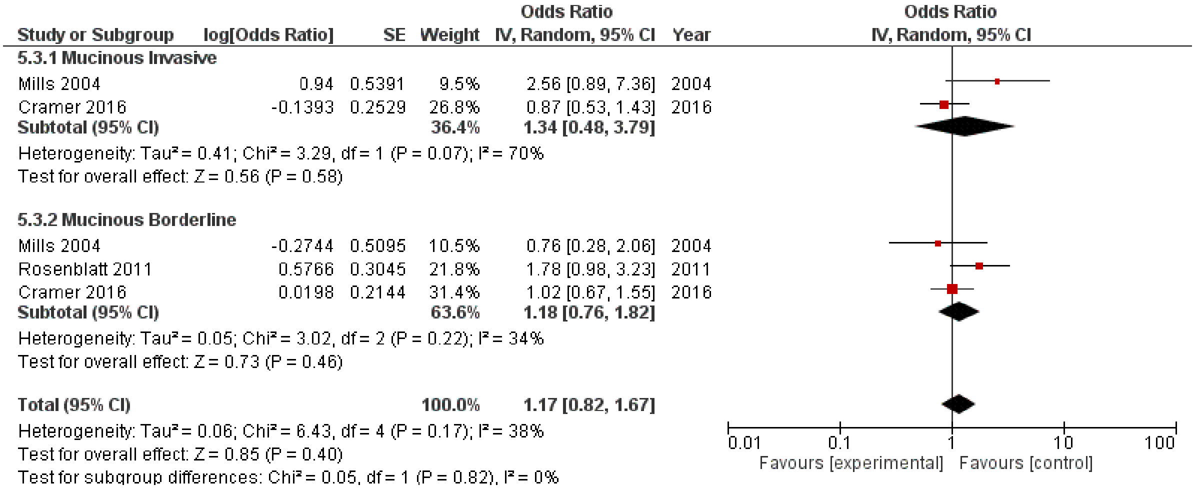

Perineal talc use and ovarian cancer, for endometrioid ovarian tumors

Study or Subgroup	log[Odds Ratio]	SE	Weight	Odds Ratio IV, Random, 95% CI	Year
**5.4.1 Endometrioid Invasive**					
Cramer 2016	0.3221	0.1346	100.0%	1.38 [1.06, 1.80]	2016
**Subtotal (95% CI)**			**100.0%**	**1.38 [1.06, 1.80]**	
Heterogeneity: Not applicable					
Test for overall effect: Z = 2.39 (P = 0.02)					
**5.4.2 Endometrioid Borderline**					
**Subtotal (95% CI)**				**Not estimable**	
Heterogeneity: Not applicable					
Test for overall effect: Not applicable					
**Total (95% CI)**			**100.0%**	**1.38 [1.06, 1.80]**	
Heterogeneity: Not applicable					
Test for overall effect: Z = 2.39 (P = 0.02)					
Test for subgroup differences: Not applicable					

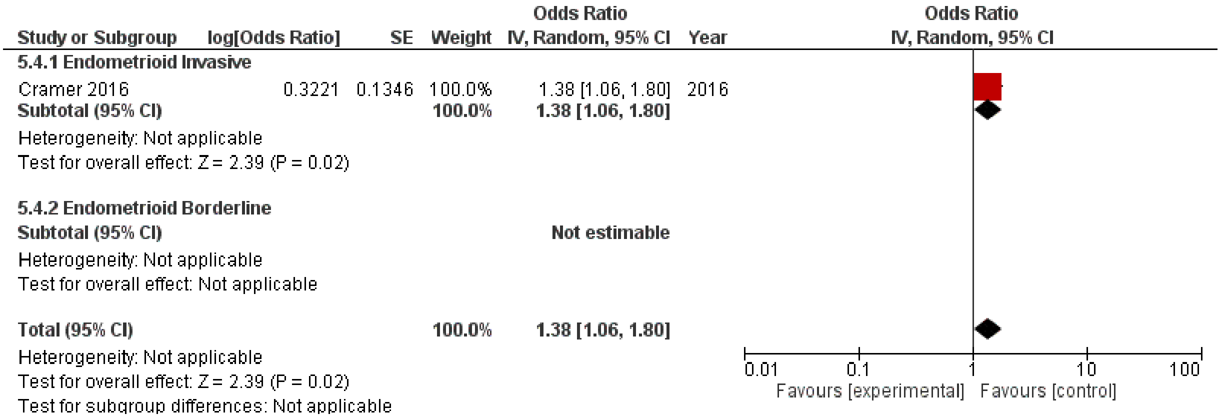

Perineal talc use and ovarian cancer, for clear-cell ovarian tumors

Study or Subgroup	log[Odds Ratio]	SE	Weight	Odds Ratio IV, Random, 95% CI	Year
**5.5.1 Clear Cell Invasive**					
Cramer 2016	0.01	0.2249	100.0%	1.01 [0.65, 1.57]	2016
**Subtotal (95% CI)**			**100.0%**	**1.01 [0.65, 1.57]**	
Heterogeneity: Not applicable					
Test for overall effect: Z = 0.04 (P = 0.96)					
**5.5.2 Clear Cell Borderline**					
**Subtotal (95% CI)**				**Not estimable**	
Heterogeneity: Not applicable					
Test for overall effect: Not applicable					
**Total (95% CI)**			**100.0%**	**1.01 [0.65, 1.57]**	
Heterogeneity: Not applicable					
Test for overall effect: Z = 0.04 (P = 0.96)					
Test for subgroup differences: Not applicable					

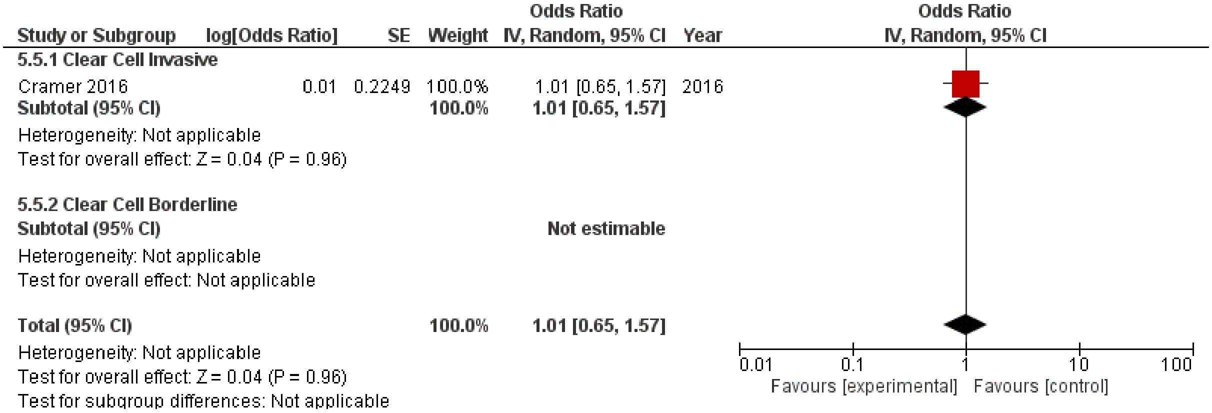

Perineal talc use – modifying factors

Perineal talc use and ovarian cancer, by menopausal state

Study or Subgroup	log[Odds Ratio]	SE	Weight	Odds Ratio IV, Random, 95% CI	Year
**6.1.1 Pre-menopausal**					
Schildkraut 2016	0.4055	0.2779	12.9%	1.50 [0.87, 2.59]	2016
Cramer 2016	0.3436	0.1129	20.6%	1.41 [1.13, 1.76]	2016
**Subtotal (95% CI)**			**33.4%**	**1.42 [1.16, 1.75]**	
Heterogeneity: Tau² = 0.00; Chi² = 0.04, df = 1 (P = 0.84); I² = 0%					
Test for overall effect: Z = 3.37 (P = 0.0008)					
**6.1.2 Post-Menopausal (HT)**					
Cramer 2016	0.793	0.1553	18.6%	2.21 [1.63, 3.00]	2016
Schildkraut 2016	0.9858	0.3575	10.0%	2.68 [1.33, 5.40]	2016
**Subtotal (95% CI)**			**28.6%**	**2.28 [1.72, 3.01]**	
Heterogeneity: Tau² = 0.00; Chi² = 0.24, df = 1 (P = 0.62); I² = 0%					
Test for overall effect: Z = 5.78 (P < 0.00001)					
**6.1.3 Post-Menopausal (no HT)**					
Cramer 2016	-0.0305	0.1112	20.6%	0.97 [0.78, 1.21]	2016
Schildkraut 2016	0.2151	0.1808	17.4%	1.24 [0.87, 1.77]	2016
**Subtotal (95% CI)**			**38.0%**	**1.05 [0.84, 1.32]**	
Heterogeneity: Tau² = 0.01; Chi² = 1.34, df = 1 (P = 0.25); I² = 25%					
Test for overall effect: Z = 0.44 (P = 0.66)					
**Total (95% CI)**			**100.0%**	**1.49 [1.11, 2.00]**	
Heterogeneity: Tau² = 0.10; Chi² = 23.10, df = 5 (P = 0.0003); I² = 78%					
Test for overall effect: Z = 2.68 (P = 0.007)					
Test for subgroup differences: Chi² = 17.74, df = 2 (P = 0.0001), I² = 88.7%					

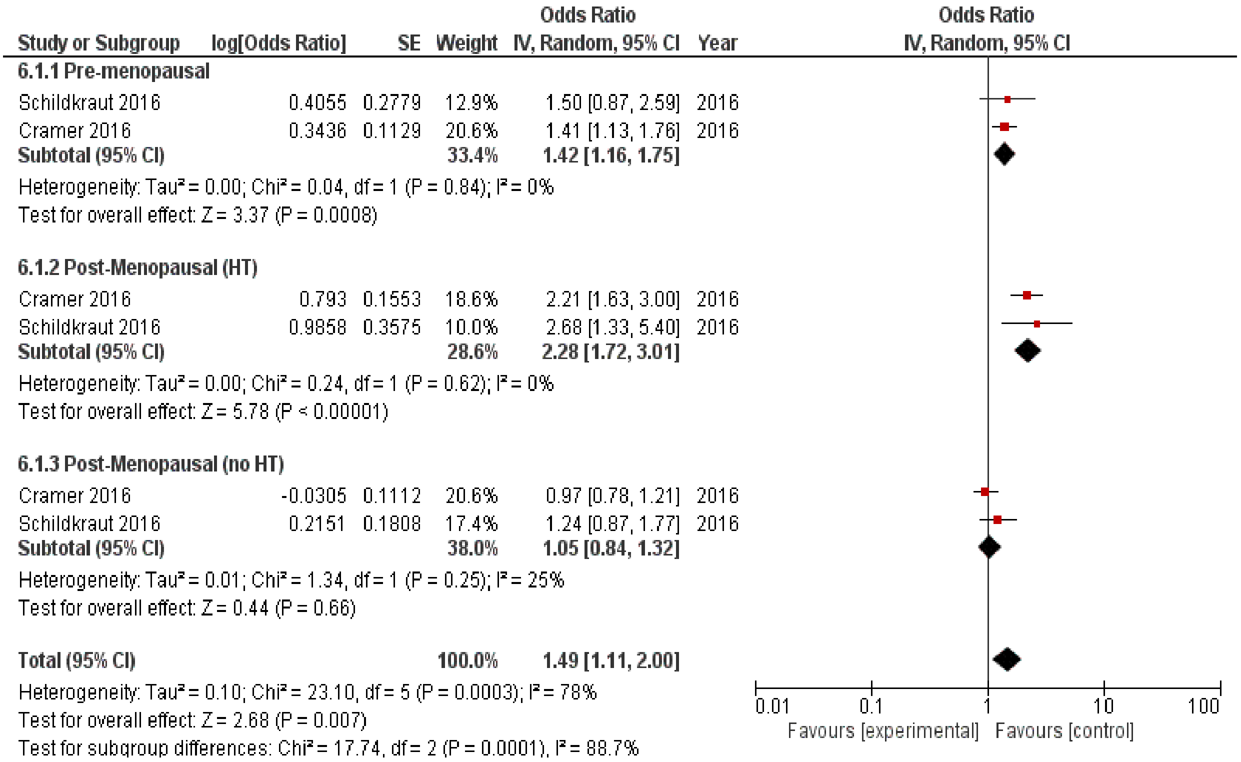

Perineal talc use and ovarian cancer, by pelvic surgery

Study or Subgroup	log[Odds Ratio]	SE	Weight	Odds Ratio IV, Random, 95% CI	Year
**6.2.1 Tubal Ligation**					
Rosenblatt 1992	-1.6094	0.9679	1.9%	0.20 [0.03, 1.33]	1992
Green 1997	-0.4943	0.144	12.6%	0.61 [0.46, 0.81]	1997
Mills 2004	-0.1278	0.331	8.2%	0.88 [0.46, 1.68]	2004
**Subtotal (95% CI)**			**22.7%**	**0.64 [0.45, 0.92]**	
Heterogeneity: Tau² = 0.03; Chi² = 2.47, df = 2 (P = 0.29); I² = 19%					
Test for overall effect: Z = 2.38 (P = 0.02)					
**6.2.2 Hysterectomy**					
Rosenblatt 1992	-0.3567	0.4323	6.3%	0.70 [0.30, 1.63]	1992
Green 1997	-0.4463	0.1468	12.5%	0.64 [0.48, 0.85]	1997
Wong 1999	-0.1054	0.4137	6.6%	0.90 [0.40, 2.02]	1999
Mills 2004	0.5822	0.3452	7.9%	1.79 [0.91, 3.52]	2004
**Subtotal (95% CI)**			**33.2%**	**0.89 [0.54, 1.46]**	
Heterogeneity: Tau² = 0.15; Chi² = 7.73, df = 3 (P = 0.05); I² = 61%					
Test for overall effect: Z = 0.46 (P = 0.65)					
**6.2.3 Combined**					
Cramer 1982	0.157	0.2201	10.8%	1.17 [0.76, 1.80]	1982
Whittemore 1988	-0.2877	0.2838	9.2%	0.75 [0.43, 1.31]	1988
Wong 1999	-0.2231	0.2398	10.3%	0.80 [0.50, 1.28]	1999
Cramer 2016	0.2927	0.0736	13.9%	1.34 [1.16, 1.55]	2016
**Subtotal (95% CI)**			**44.1%**	**1.06 [0.78, 1.42]**	
Heterogeneity: Tau² = 0.05; Chi² = 7.66, df = 3 (P = 0.05); I² = 61%					
Test for overall effect: Z = 0.35 (P = 0.72)					
**Total (95% CI)**			**100.0%**	**0.87 [0.66, 1.16]**	
Heterogeneity: Tau² = 0.14; Chi² = 45.68, df = 10 (P < 0.00001); I² = 78%					
Test for overall effect: Z = 0.94 (P = 0.35)					
Test for subgroup differences: Chi² = 4.27, df = 2 (P = 0.12), I² = 53.2%					

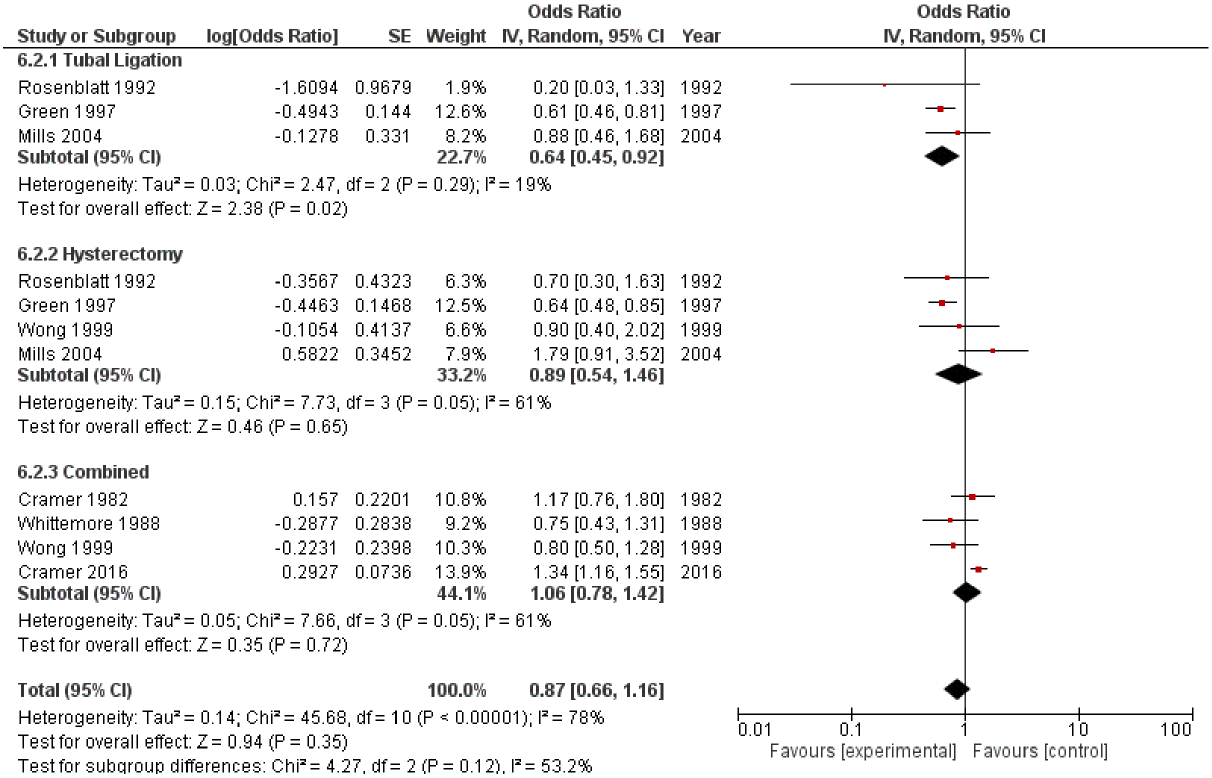

Perineal talc use and ovarian cancer – influence analysis

Table 9: Influence analysis of individual studies included in this meta-analysis

Examined Studies	aOR	95% CI	I^2 Statistic	P-Heterogeneity
Cramer et al. (1982) [2]	1.30	[1.22 to	24%	0.15
Hartge et al. (1983) [3]	1.29	[1.22 to	27%	0.10
Whittemore et al. (1988) [4]	1.28	[1.20 to	35%	0.04
Harlow & Weiss (1989) [6]	1.29	[1.21 to	35%	0.04
Harlow et al. (1992) [8]	1.28	[1.20 to	35%	0.04
Rosenblatt et al. (1992) [9]	1.28	[1.20 to	35%	0.04
Tzonou et al. (1993) [10]	1.28	[1.20 to	35%	0.04
Chang & Risch (1997) [12]	1.28	[1.20 to	35%	0.04
Cook et al. (1997) [13]	1.28	[1.20 to	33%	0.05
Green et al. (1997) [41]	1.28	[1.20 to	35%	0.04
Godard et al. (1998) [15]	1.28	[1.20 to	32%	0.06
Wong et al. (1999) [17]	1.30	[1.22 to	26%	0.12
Gertig et al. (2000) [20]	1.29	[1.21 to	32%	0.06
Ness et al. (2000) [18]	1.28	[1.20 to	34%	0.05
Langseth and Kjærheim	1.28	[1.20 to	35%	0.04
Mills et al. (2004) [19]	1.28	[1.20 to	35%	0.04
Gates et al. (2008) [43]	1.28	[1.19 to	35%	0.04
Merritt et al. (2008) [45]	1.29	[1.21 to	32%	0.06
Moorman et al. (2009) [46]	1.30	[1.22 to [illegible]	30%	0.08
Wu et al. (2009) [47]	1.28	[1.20 to	33%	0.05
Rosenblatt et al. (2011) [48]	1.28	[1.20 to	35%	0.04
Kurta et al. (2012) [49]	1.28	[1.19 to	34%	0.05
Houghton et al. (2014) [55]	1.30	[1.21 to	32%	0.06
Wu et al. (2015) [51]	1.27	[1.19 to	29%	0.09
Cramer et al. (2016) [52]	1.28	[1.20 to	35%	0.04
Gonzalez et al. (2016) [56]	1.29	[1.22 to	26%	0.11
Schildkraut et al. (2016)	1.28	[1.20 to	34%	0.05

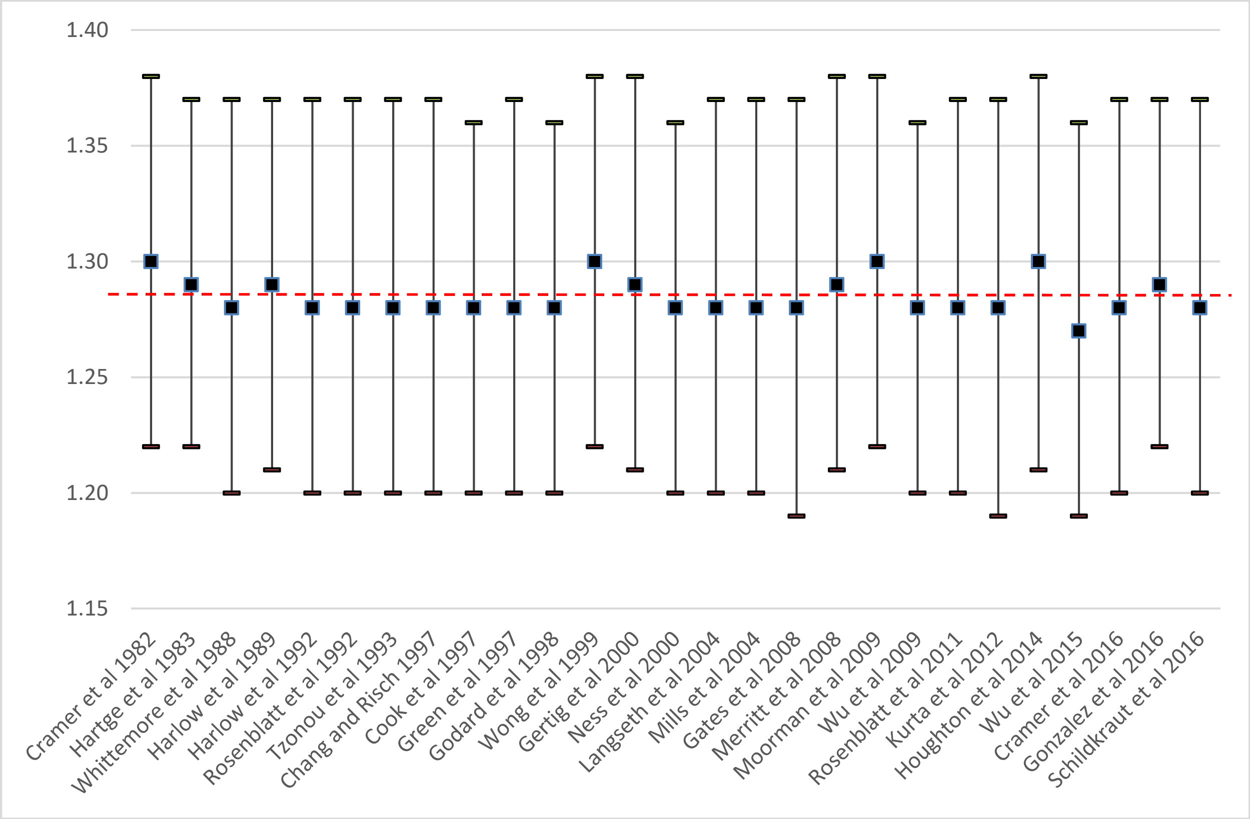

FIGURE 2: Influence analysis study.

The dashed line represents the overall pooled odds ratio

References

[1] IARC/International Agency for Research on Cancer, Carbon black, titanium dioxide, and talc, IARC Monogr Eval Carcinog Risks Hum 93 (2010) 1-413.

[2] D.W. Cramer, W.R. Welch, R.E. Scully, C.A. Wojciechowski, Ovarian cancer and talc: a case-control study, Cancer 50(2) (1982) 372-6.

[3] P. Hartge, R. Hoover, L.P. Lesher, L. McGowan, Talc and ovarian cancer, JAMA : the journal of the American Medical Association 250(14) (1983) 1844.

[4] A.S. Whittemore, M.L. Wu, R.S. Paffenbarger Jr, D.L. Sarles, J.B. Kampert, S. Grosser, D.L. Jung, S. Ballon, M. Hendrickson, Personal and environmental characteristics related to epithelial ovarian cancer. II. Exposures to talcum powder, tobacco, alcohol, and coffee, American Journal of Epidemiology 128(6) (1988) 1228-1240.

[5] M. Booth, V. Beral, P. Smith, Risk factors for ovarian cancer: a case-control study, Br J Cancer 60(4) (1989) 592-8.

[6] B.L. Harlow, N.S. Weiss, A case-control study of borderline ovarian tumors: the influence of perineal exposure to talc, American Journal of Epidemiology 130(2) (1989) 390-4.

[7] Y. Chen, P.C. Wu, J.H. Lang, W.J. Ge, P. Hartge, L.A. Brinton, Risk factors for epithelial ovarian cancer in Beijing, China, International journal of epidemiology 21(1) (1992) 23-9.

[8] B.L. Harlow, D.W. Cramer, D.A. Bell, W.R. Welch, Perineal exposure to talc and ovarian cancer risk, Obstet Gynecol 80(1) (1992) 19-26.

[9] K.A. Rosenblatt, M. Szklo, N.B. Rosenshein, Mineral fiber exposure and the development of ovarian cancer, Gynecologic Oncology 45(1) (1992) 20-25.

[10] A. Tzonou, A. Polychronopoulou, C.C. Hsieh, A. Rebelakos, A. Karakatsani, D. Trichopoulos, Hair dyes, analgesics, tranquilizers and perineal talc application as risk factors for ovarian cancer, International Journal of Cancer 55(3) (1993) 408-10.

[11] A. Shushan, O. Paltiel, J. Iscovich, U. Elchalal, T. Peretz, J.G. Schenker, Human menopausal gonadotropin and the risk of epithelial ovarian cancer, Fertility and sterility 65(1) (1996) 13-8.

[12] S. Chang, H.A. Risch, Perineal talc exposure and risk of ovarian carcinoma, Cancer 79(12) (1997) 2396-401.

[13] L.S. Cook, M.L. Kamb, N.S. Weiss, Perineal powder exposure and the risk of ovarian cancer.[Erratum appears in Am J Epidemiol 1998 Aug 15;148(4):410], American Journal of Epidemiology 145(5) (1997) 459-65.

[14] G.H. Eltabbakh, M.S. Piver, N. Natarajan, C.J. Mettlin, Epidemiologic differences between women with extraovarian primary peritoneal carcinoma and women with epithelial ovarian cancer, Obstet Gynecol 91(2) (1998) 254-9.

[15] B. Godard, W.D. Foulkes, D. Provencher, J.S. Brunet, P.N. Tonin, A.M. Mes-Masson, S.A. Narod, P. Ghadirian, Risk factors for familial and sporadic ovarian cancer

among French Canadians: a case-control study, Am J Obstet Gynecol 179(2) (1998) 403-10.

[16] D.W. Cramer, R.F. Liberman, L. Titus-Ernstoff, W.R. Welch, E.R. Greenberg, J.A. Baron, B.L. Harlow, Genital talc exposure and risk of ovarian cancer, International Journal of Cancer 81(3) (1999) 351-6.

[17] C. Wong, R.E. Hempling, M.S. Piver, N. Natarajan, C.J. Mettlin, Perineal talc exposure and subsequent epithelial ovarian cancer: a case-control study, Obstet Gynecol 93(3) (1999) 372-6.

[18] R.B. Ness, J.A. Grisso, C. Cottreau, J. Klapper, R. Vergona, J.E. Wheeler, M. Morgan, J.J. Schlesselman, Factors related to inflammation of the ovarian epithelium and risk of ovarian cancer, Epidemiology 11(2) (2000) 111-7.

[19] P.K. Mills, D.G. Riordan, R.D. Cress, H.A. Young, Perineal talc exposure and epithelial ovarian cancer risk in the Central Valley of California, International Journal of Cancer 112(3) (2004) 458-64.

[20] D.M. Gertig, D.J. Hunter, D.W. Cramer, G.A. Colditz, F.E. Speizer, W.C. Willett, S.E. Hankinson, Prospective study of talc use and ovarian cancer, J Natl Cancer Inst 92(3) (2000) 249-52.

[21] ACGIH (American Conference of Governmental Industrial Hygienists), Threshold Limit Values for Chemical Substances, USA, 2016.

[22] OSHA-Occupational Safety and Health Administration, Permissible Exposure Levels/PEL 2013. 29 CFR 1910.1000 OSHA Permissible Exposure Levels 6/2006 2013. https://igsvtu.lanuv.nrw.de/igs70/oberfl/mkmtexte/de/t1285.pdf. (Accessed 22 June 2017).

[23] GRF/German Research Foundation/ Deutsche Forschungsgemeinschaft (DFG), Talc (without asbestos fibres) (respirable fraction) [MAK Value Documentation, 2006], 2006. http://onlinelibrary.wiley.com/doi/10.1002/3527600418.mb1480796nfae0022/pdf. (Accessed 22 June 2017).

[24] Health Council of the Netherlands-The Hague, Talc. Evaluation of the carcinogenicity and genotoxicity Subcommittee on the Classification of Carcinogenic Substances of the Dutch Expert Committee on Occupational Safety, a Committee of the Health Council of the Netherlands, 2012. https://www.gezondheidsraad.nl/sites/default/files/Talc201211_0.pdf. (Accessed 22 June 2017).

[25] ANSES-French Agency for Food Environmental and Occupational Health & Safety, OPINION of the French Agency for Food, Environmental and Occupational Health & Safety on the assessment of the risks related to talc alone and talc contaminated by asbestiform and non-asbestiform fibres, 2012. https://www.anses.fr/en/system/files/AIR2009sa0332RaEN.pdf. (Accessed 22 June 2017).

[26] E. REACH-Registration, Authorisation and Restriction of Chemicals, Registered substances, Belgium,, Talc. Joint submission. First published: 19-Jan-2017 last

modified: 21-Apr-2017. Full submission., 2017. https://echa.europa.eu/registration-dossier/-/registered-dossier/18727/2/2. (Accessed 22 June 2017).

[27] Health Canada, Government of Canada. Canada Gazette. Food and Drugs Act Regulations. Amending the Food and Drug Regulations (1609 — Talc). Vol. 145, No. 26s. Registration: SOR/2011-279, 2011. http://www.gazette.gc.ca/rp-pr/p2/2011/2011-12-21/html/sor-dors279-eng.html. (Accessed 22 June 2017).

[28] Health Canada, Government of Canada. Canada Gazette. Food and Drugs Act. Food and Drug Regulations. Interim marketing authorization. , 2003. http://publications.gc.ca/gazette/archives/p1/2003/2003-08-16/pdf/g1-13733.pdf. (Accessed 22 June 2017).

[29] FDA (USA), Food Additive Status List, 2015. https://igsvtu.lanuv.nrw.de/igs70/oberfl/mkmtexte/de/t2481.pdf (Accessed 22 June 2017).

[30] FDA (USA), Color Additive Status List, 2009. https://igsvtu.lanuv.nrw.de/igs70/oberfl/mkmtexte/de/t2199.pdf. (Accessed 22 June 2017).

[31] FDA (USA), EAFUS Food Additive Database, 2015. https://igsvtu.lanuv.nrw.de/igs70/oberfl/mkmtexte/de/t2200.pdf. (Accessed 22 June 2017).

[32] Japan Food Chemical Research Foundation, List of Existing Food Additives. Effective from January 30, 2014, 2014. https://igsvtu.lanuv.nrw.de/igs70/oberfl/mkmtexte/de/t3558.pdf.

[33] Japan Food Chemical Research Foundation, Standards for Use, according to Use Categories. Effective from August 8, 2014, 2014. https://igsvtu.lanuv.nrw.de/igs70/oberfl/mkmtexte/de/t3556.pdf.

[34] FAO/WHO, Codex Alimentarius. International Food Standards. General Standards for Food additives. CODEX STAN 192-1995. Adopted in 1995. Revision 1997, 1999, 2001, 2003, 2004, 2005, 2006, 2007, 2008, 2009, 2010, 2011, 2012, 2013 & 2014, 2014. https://igsvtu.lanuv.nrw.de/igs70/oberfl/mkmtexte/de/t2119.pdf. (Accessed 22 June 2017).

[35] IOFI-International Organization of the Flavor Industry, Code of Practice. Substances in flavorings, 2012. https://igsvtu.lanuv.nrw.de/igs70/oberfl/mkmtexte/de/t3650.pdf. (Accessed 22 June 2017).

[36] Health Canada, Government of Canada. List of Ingredients that are Restricted for Use in Cosmetic Products 2015. https://www.canada.ca/en/health-canada/services/consumer-product-safety/cosmetics/cosmetic-ingredient-hotlist-prohibited-restricted-ingredients/hotlist.html#tbl2. (Accessed 22 June 2017).

[37] European Union, Regulation (EC) No 1223/2009 of the European Parliament and of the Council of 30 November 2009 on cosmetic products (recast) (Text with EEA

relevance), 2009. http://eur-lex.europa.eu/legal-content/EN/TXT/PDF/?uri=CELEX:32009R1223&qid=1498164622818&from=EN. (Accessed 22 June 2017).

[38] W. Berge, K. Mundt, H. Luu, P. Boffetta, Genital use of talc and risk of ovarian cancer: a meta-analysis, European journal of cancer prevention : the official journal of the European Cancer Prevention Organisation (ECP) (2017).

[39] D.W. Cramer, H. Xu, Epidemiologic evidence for uterine growth factors in the pathogenesis of ovarian cancer, Ann Epidemiol 5(4) (1995) 310-4.

[40] D. Purdie, A. Green, C. Bain, V. Siskind, B. Ward, N. Hacker, M. Quinn, G. Wright, P. Russell, B. Susil, Reproductive and other factors and risk of epithelial ovarian cancer: an Australian case-control study. Survey of Women's Health Study Group, International Journal of Cancer 62(6) (1995) 678-84.

[41] A. Green, D. Purdie, C. Bain, V. Siskind, P. Russell, M. Quinn, B. Ward, Tubal sterilisation, hysterectomy and decreased risk of ovarian cancer. Survey of Women's Health Study Group, International Journal of Cancer 71(6) (1997) 948-51.

[42] H. Langseth, K. Kjaerheim, Ovarian cancer and occupational exposure among pulp and paper employees in Norway, Scand J Work Environ Health 30(5) (2004) 356-61.

[43] M.A. Gates, S.S. Tworoger, K.L. Terry, L. Titus-Ernstoff, B. Rosner, I.d. Vivo, D.W. Cramer, S.E. Hankinson, Talc use, variants of the GSTM1, GSTT1, and NAT2 genes, and risk of epithelial ovarian cancer, Cancer Epidemiol Biomarkers Prev 17(9) (2008) 2436-2444.

[44] M.T. Goodman, G. Lurie, P.J. Thompson, K.E. McDuffie, M.E. Carney, Association of two common single-nucleotide polymorphisms in the CYP19A1 locus and ovarian cancer risk, Endocr.Relat Cancer 15(4) (2008) 1055-1060.

[45] M.A. Merritt, A.C. Green, C.M. Nagle, P.M. Webb, S. Australian Cancer, G. Australian Ovarian Cancer Study, Talcum powder, chronic pelvic inflammation and NSAIDs in relation to risk of epithelial ovarian cancer, International Journal of Cancer 122(1) (2008) 170-6.

[46] P.G. Moorman, R.T. Palmieri, L. Akushevich, A. Berchuck, J.M. Schildkraut, Ovarian cancer risk factors in African-American and white women, Am J Epidemiol 170(5) (2009) 598-606.

[47] A.H. Wu, C.L. Pearce, C.C. Tseng, C. Templeman, M.C. Pike, Markers of inflammation and risk of ovarian cancer in Los Angeles County, International Journal of Cancer 124(6) (2009) 1409-15.

[48] K.A. Rosenblatt, N.S. Weiss, K.L. Cushing-Haugen, K.G. Wicklund, M.A. Rossing, Genital powder exposure and the risk of epithelial ovarian cancer, Cancer Causes Control 22(5) (2011) 737-42.

[49] M.L. Kurta, K.B. Moysich, J.L. Weissfeld, A.O. Youk, C.H. Bunker, R.P. Edwards, F. Modugno, R.B. Ness, B. Diergaarde, Use of fertility drugs and risk of ovarian cancer: results from a U.S.-based case-control study, Cancer Epidemiol Biomarkers Prev 21(8) (2012) 1282-92.

[50] W.H. Lo-Ciganic, J.C. Zgibor, C.H. Bunker, K.B. Moysich, R.P. Edwards, R.B. Ness, Aspirin, nonaspirin nonsteroidal anti-inflammatory drugs, or acetaminophen and risk of ovarian cancer, Epidemiology 23(2) (2012) 311-319.

[51] A.H. Wu, C.L. Pearce, C.C. Tseng, M.C. Pike, African Americans and Hispanics Remain at Lower Risk of Ovarian Cancer Than Non-Hispanic Whites after Considering Nongenetic Risk Factors and Oophorectomy Rates, Cancer Epidemiol Biomarkers Prev 24(7) (2015) 1094-100.

[52] D.W. Cramer, A.F. Vitonis, K.L. Terry, W.R. Welch, L.J. Titus, The Association Between Talc Use and Ovarian Cancer: A Retrospective Case-Control Study in Two US States, Epidemiology 27(3) (2016) 334-46.

[53] J.M. Schildkraut, S.E. Abbott, A.J. Alberg, E.V. Bandera, J.S. Barnholtz-Sloan, M.L. Bondy, M.L. Cote, E. Funkhouser, L.C. Peres, E.S. Peters, A.G. Schwartz, P. Terry, S. Crankshaw, F. Camacho, F. Wang, P.G. Moorman, Association between Body Powder Use and Ovarian Cancer: The African American Cancer Epidemiology Study (AACES), Cancer Epidemiol Biomarkers Prev 25(10) (2016) 1411-1417.

[54] M.A. Gates, B.A. Rosner, J.L. Hecht, S.S. Tworoger, Risk factors for epithelial ovarian cancer by histologic subtype, Am J Epidemiol 171(1) (2010) 45-53.

[55] S.C. Houghton, K.W. Reeves, S.E. Hankinson, L. Crawford, D. Lane, J. Wactawski-Wende, C.A. Thomson, J.K. Ockene, S.R. Sturgeon, Perineal powder use and risk of ovarian cancer, J Natl Cancer Inst 106(9) (2014).

[56] N.L. Gonzalez, K.M. O'Brien, A.A. D'Aloisio, D.P. Sandler, C.R. Weinberg, Douching, Talc Use, and Risk of Ovarian Cancer, Epidemiology 27(6) (2016) 797-802.

[57] H. Langseth, S.E. Hankinson, J. Siemiatycki, E. Weiderpasse, Perineal use of talc and risk of ovarian cancer, Journal of Epidemiology and Community Health 62(4) (2008) 358-360.

[58] K.L. Terry, S. Karageorgi, Y.B. Shvetsov, M.A. Merritt, G. Lurie, P.J. Thompson, M.E. Carney, R.P. Weber, L. Akushevich, W.H. Lo-Ciganic, K. Cushing-Haugen, W. Sieh, K. Moysich, J.A. Doherty, C.M. Nagle, A. Berchuck, C.L. Pearce, M. Pike, R.B. Ness, P.M. Webb, S. Australian Cancer, G. Australian Ovarian Cancer Study, M.A. Rossing, J. Schildkraut, H. Risch, M.T. Goodman, C. Ovarian Cancer Association, Genital powder use and risk of ovarian cancer: a pooled analysis of 8,525 cases and 9,859 controls, Cancer Prevention Research 6(8) (2013) 811-21.

[59] R. Penninkilampi, G.D. Eslick, Perineal Talc Use and Ovarian Cancer: A Systematic Review and Meta-Analysis, Epidemiology 29(1) (2018) 41-49.

[60] M. Huncharek, J.F. Geschwind, B. Kupelnick, Perineal application of cosmetic talc and risk of invasive epithelial ovarian cancer: a meta-analysis of 11,933 subjects from sixteen observational studies, Anticancer Res 23(2C) (2003) 1955-60.

[61] G. Wells, B. Shea, D. O'Connell, J. Peterson, V. Welch, M. Losos, P. Tugwell, The Newcastle-Ottawa Scale (NOS) for assessing the quality of nonrandomised studies in meta-analyses, 2008. http://www.ohri.ca/programs/clinical_epidemiology/oxford.asp. (Accessed May 8 2017).

[62] J. Higgins, S. Green, Cochrane Handbook for Systematic Reviews of Interventions, 2011. www.cochrane-handbook.org.

[63] Evidence Partners, Distiller SR, Ottawa, ON, Canada.

[64] D. Moher, K.F. Schulz, D.G. Altman, The CONSORT statement: revised recommendations for improving the quality of reports of parallel-group randomised trials, Clinical oral investigations 7(1) (2003) 2-7.

[65] H.J. Klimisch, M. Andreae, U. Tillmann, A systematic approach for evaluating the quality of experimental toxicological and ecotoxicological data, Regulatory toxicology and pharmacology : RTP 25(1) (1997) 1-5.

[66] K. Schneider, M. Schwarz, I. Burkholder, A. Kopp-Schneider, L. Edler, A. Kinsner-Ovaskainen, T. Hartung, S. Hoffmann, "ToxRTool", a new tool to assess the reliability of toxicological data, Toxicology letters 189(2) (2009) 138-44.

[67] NTP/National Toxicology Program, NTP Toxicology and Carcinogenesis Studies of Talc (CAS No. 14807-96-6)(Non-Asbestiform) in F344/N Rats and B6C3F1 Mice (Inhalation Studies), Natl Toxicol Program Tech Rep Ser, 1993, pp. 1-287.

[68] G.A. Boorman, J.C. Seely, The lack of an ovarian effect of lifetime talc exposure in F344/N rats and B6C3F1 mice, Regulatory Toxicology & Pharmacology 21(2) (1995) 242-3.

[69] J.A. Pickrell, M.B. Snipes, J.M. Benson, R.L. Hanson, R.K. Jones, R.L. Carpenter, J.J. Thompson, C.H. Hobbs, S.C. Brown, Talc deposition and effects after 20 days of repeated inhalation exposure of rats and mice to talc, Environ Res 49(2) (1989) 233-45.

[70] J.C. Wagner, G. Berry, T.J. Cooke, R.J. Hill, F.D. Pooley, J.W. Skidmore, Animal experiments with talc, in: W.H. Walton, B. McGovern (Eds.), Inhaled Particles IV, Part 2, Pergamon Press, Oxford, UK, 1977, pp. 647–654.

[71] A.P. Wehner, G.M. Zwicker, W.C. Cannon, Inhalation of talc baby powder by hamsters, Food Cosmet Toxicol 15(2) (1977) 121-9.

[72] A. Woywodt, W. Schneider, U. Goebel, F.C. Luft, Hypercalcemia due to talc granulomatosis, Chest 117(4) (2000) 1195-6.

[73] F. Stenback, J. Rowlands, Role of talc and benzo(a)pyrene in respiratory tumor formation. An experimental study, Scandinavian journal of respiratory diseases 59(3) (1978) 130-40.

[74] F. Bischoff, G. Bryson, Talc at Rodent Intrathoracic, Intraperitoneal, and Subcutaneous Sitse, Proceedings of The American Association for Cancer Research, American Association for Cancer Research Public Ledger Bldg, Suite 816, 150 S. Independence Mall W., Philadelphia, PA 19106, 1976, pp. 1-1.

[75] J. Jagatic, M.E. Rubnitz, M.C. Godwin, R.W. Weiskopf, Tissue response to intraperitoneal asbestos with preliminary report of acute toxicity of heart-treated asbestos in mice, Environ Res 1(3) (1967) 217-30.

[76] M. Ozesmi, T.E. Patiroglu, G. Hillerdal, C. Ozesmi, Peritoneal mesothelioma and malignant lymphoma in mice caused by fibrous zeolite, Br J Ind Med 42(11) (1985) 746-9.

[77] F. Pott, R. Dolgner, K.H. Friedrichs, F. Huth, [The oncogenic effect of fibrous dust. Animal experiments and their relationship with human carcinogenesis], Annales d'anatomie pathologique 21(2) (1976) 237-46.

[78] F. Pott, K.H. Friedrichs, F. Huth, [Results of animal experiments concerning the carcinogenic effect of fibrous dusts and their interpretation with regard to the carcinogenesis in humans (author's transl)], Zentralblatt fur Bakteriologie, Parasitenkunde, Infektionskrankheiten und Hygiene. Erste Abteilung Originale. Reihe B: Hygiene, praventive Medizin 162(5-6) (1976) 467-505.

[79] F. Pott, F. Huth, K.H. Friedrichs, Tumorigenic effect of fibrous dusts in experimental animals, Environmental health perspectives 9 (1974) 313-5.

[80] J.A. Styles, J. Wilson, Comparison between in vitro toxicity of polymer and mineral dusts and their fibrogenicity, Ann Occup Hyg 16(3) (1973) 241-50.

[81] S. Neukomm, M. De Trey, Etude de certains azureurs optiques du point de vue de leur pouvoir cancérigène et co-cancérigène, Pharmacology 4(5) (1961) 298-306.

[82] W. Gibel, K. Lohs, K.H. Horn, G.P. Wildner, F. Hoffmann, [Experimental study on cancerogenic activity of asbestos filters (author's transl)], Archiv fur Geschwulstforschung 46(6) (1976) 437-42.

[83] T.C. Hamilton, H. Fox, C.H. Buckley, W.J. Henderson, K. Griffiths, Effects of talc on the rat ovary, British journal of experimental pathology 65(1) (1984) 101-6.

[84] B. Celik, O. Aydin, A. Cilingir, A. Basoglu, A comparison of the effectiveness of talc, polidocanol and ethanol as pleural sclerosing agents in rabbits, The Thoracic and cardiovascular surgeon 57(2) (2009) 102-6.

[85] E.H. Genofre, F.S. Vargas, L. Antonangelo, L.R. Teixeira, M.A. Vaz, E. Marchi, V.L. Capelozzi, Ultrastructural acute features of active remodeling after chemical pleurodesis induced by silver nitrate or talc, Lung 183(3) (2005) 197-207.

[86] E.H. Genofre, F.S. Vargas, M.M. Acencio, L. Antonangelo, L.R. Teixeira, E. Marchi, Talc pleurodesis: evidence of systemic inflammatory response to small size talc particles, Respiratory medicine 103(1) (2009) 91-7.

[87] Y. Iwasaki, S. Takamori, M. Mitsuoka, M. Kashihara, T. Nishi, D. Murakami, R. Matsumoto, H. Mifune, Y. Tajiri, Y. Akagi, Experimental validation of talc pleurodesis for carcinomatous pleuritis in an animal model, General thoracic and cardiovascular surgery 64(7) (2016) 409-13.

[88] E. Marchi, F.S. Vargas, M.M. Acencio, L. Antonangelo, E.H. Genofre, L.R. Teixeira, Evidence that mesothelial cells regulate the acute inflammatory response in talc pleurodesis, The European respiratory journal 28(5) (2006) 929-32.

[89] Q. Miller, C. Meschter, T. Neumaster, J. Pratt, M. Moulton, D. Downey, J. Harre, Comparison of pleurodesis by erythromycin, talc, doxycycline, and diazepam in a rabbit model, Journal of surgical education 64(1) (2007) 41-5.

[90] V.F. Rossi, F.S. Vargas, E. Marchi, M.M. Acencio, E.H. Genofre, V.L. Capelozzi, L. Antonangelo, Acute inflammatory response secondary to intrapleural administration of two types of talc, The European respiratory journal 35(2) (2010) 396-401.

[91] C. Puel, J. Mardon, S. Kati-Coulibaly, M.J. Davicco, P. Lebecque, C. Obled, E. Rock, M.N. Horcajada, A. Agalias, L.A. Skaltsounis, V. Coxam, Black Lucques olives prevented bone loss caused by ovariectomy and talc granulomatosis in rats, The British journal of nutrition 97(5) (2007) 1012-20.

[92] E. Orsal, Z. Halici, Y. Bayir, E. Cadirci, H. Bilen, I. Ferah, A. Aydin, S. Ozkanlar, A.K. Ayan, B. Seven, S. Ozaltin, The role of carnitine on ovariectomy and inflammation-induced osteoporosis in rats, Experimental biology and medicine (Maywood, N.J.) 238(12) (2013) 1406-12.

[93] B. Polat, Z. Halici, E. Cadirci, A. Albayrak, E. Karakus, Y. Bayir, H. Bilen, A. Sahin, T.N. Yuksel, The effect of alpha-lipoic acid in ovariectomy and inflammation-mediated osteoporosis on the skeletal status of rat bone, Eur J Pharmacol 718(1-3) (2013) 469-74.

[94] L.R. Teixeira, F.S. Vargas, M.M. Acencio, S.C. Ribeiro, R.K. Sales, L. Antonangelo, E. Marchi, Blockage of vascular endothelial growth factor (VEGF) reduces experimental pleurodesis, Lung cancer (Amsterdam, Netherlands) 74(3) (2011) 392-5.

[95] N. Keskin, Y.A. Teksen, E.G. Ongun, Y. Ozay, H. Saygili, Does long-term talc exposure have a carcinogenic effect on the female genital system of rats? An experimental pilot study, Arch Gynecol Obstet 280(6) (2009) 925-31.

[96] O. Yumrutas, M. Kara, R. Atilgan, S.B. Kavak, I. Bozgeyik, E. Sapmaz, Application of talcum powder, trichloroacetic acid and silver nitrate in female rats for non-surgical sterilization: evaluation of the apoptotic pathway mRNA and miRNA genes, International journal of experimental pathology 96(2) (2015) 111-5.

[97] M.J. Akhtar, S. Kumar, R.C. Murthy, M. Ashquin, M.I. Khan, G. Patil, I. Ahmad, The primary role of iron-mediated lipid peroxidation in the differential cytotoxicity caused by two varieties of talc nanoparticles on A549 cells and lipid peroxidation inhibitory effect exerted by ascorbic acid, Toxicol In Vitro 24(4) (2010) 1139-47.

[98] M.J. Akhtar, M. Ahamed, M.A. Khan, S.A. Alrokayan, I. Ahmad, S. Kumar, Cytotoxicity and apoptosis induction by nanoscale talc particles from two different geographical regions in human lung epithelial cells, Environ Toxicol 29(4) (2014) 394-406.

[99] A.R. Buz'Zard, B.H.S. Lau, Pycnogenol® reduces talc-induced neoplastic transformation in human ovarian cell cultures, Phytotherapy Research 21(6) (2007) 579-586.

[100] M. Chamberlain, R.C. Brown, The cytotoxic effects of asbestos and other mineral dust in tissue culture cell lines, British journal of experimental pathology 59(2) (1978) 183-9.

[101] R. Davies, J.W. Skidmore, D.M. Griffiths, C.B. Moncrieff, Cytotoxicity of talc for macrophages in vitro, Food and chemical toxicology : an international journal published for the British Industrial Biological Research Association 21(2) (1983) 201-7.

[102] A.J. Ghio, J.M. Soukup, L.A. Dailey, J.H. Richards, J.L. Turi, E.N. Pavlisko, V.L. Roggli, Disruption of iron homeostasis in mesothelial cells after talc pleurodesis, Am J Respir Cell Mol Biol 46(1) (2012) 80-86.

[103] W.J. Henderson, G. Blundell, R. Richards, P.M. Hext, B.E. Volcani, K. Griffiths, Ingestion of talc particles by cultured lung fibroblasts, Environ Res 9(2) (1975) 173-8.

[104] A.L. Lichtman, D.J. Mc, et al., Talc granuloma, Surgery, gynecology & obstetrics 83 (1946) 531-46.

[105] P. Lee, L. Sun, C.K. Lim, S.E. Aw, H.G. Colt, Selective apoptosis of lung cancer cells with talc, The European respiratory journal 35(2) (2010) 450-2.

[106] N. Nasreen, D.L. Hartman, K.A. Mohammed, V.B. Antony, Talc-induced expression of C-C and C-X-C chemokines and intercellular adhesion molecule-1 in mesothelial cells, Am J Respir Crit Care Med 158(3) (1998) 971-8.

[107] N. Nasreen, K.A. Mohammed, P.A. Dowling, M.J. Ward, G. Galffy, V.B. Antony, Talc induces apoptosis in human malignant mesothelioma cells in vitro, Am J Respir Crit Care Med 161(2 Pt 1) (2000) 595-600.

[108] A. Shukla, M.B. MacPherson, J. Hillegass, M.E. Ramos-Nino, V. Alexeeva, P.M. Vacek, J.P. Bond, H.I. Pass, C. Steele, B.T. Mossman, Alterations in gene expression in human mesothelial cells correlate with mineral pathogenicity, Am J Respir Cell Mol Biol 41(1) (2009) 114-23.

[109] S. Endo-Capron, A. Renier, X. Janson, L. Kheuang, M.C. Jaurand, In vitro response of rat pleural mesothelial cells to talc samples in genotoxicity assays (sister chromatid exchanges and DNA repair), Toxicol In Vitro 7(1) (1993) 7-14.

[110] IARC/International Agency for Research on Cancer, Silica and Some Silicates, IARC Monogr Eval Carcinog Risks Hum 42 (1987) 185-224.

[111] R.L. Hanson, J.M. Benson, T.R. Henderson, R.L. Carpenter, J.A. Pickrell, S.C. Brown, Method for determining the lung burden of talc in rats and mice after inhalation exposure to talc aerosols, J Appl Toxicol 5(5) (1985) 283-7.

[112] R.K. Wolff, G.M. Kanapilly, R.H. Gray, R.O. McClellan, Deposition and retention of inhaled aggregate 67Ga2O3 particles in beagle dogs, Fischer-344 rats, and CD-1 mice, Am Ind Hyg Assoc J 45(6) (1984) 377-81.

[113] A.P. Wehner, C.L. Wilkerson, W.C. Cannon, R.L. Buschbom, T.M. Tanner, Pulmonary deposition, translocation and clearance of inhaled neutron-activated talc in hamsters, Food Cosmet Toxicol 15(3) (1977) 213-24.

[114] J.C. Phillips, P.J. Young, K. Hardy, S.D. Gangolli, Studies on the absorption and disposition of 3H-labelled talc in the rat, mouse, guinea-pig and rabbit, Food Cosmet.Toxicol 16(2) (1978) 161-163.

[115] A.P. Wehner, T.M. Tanner, R.L. Buschbom, Absorption of ingested talc by hamsters, Food Cosmet Toxicol 15(5) (1977) 453-55.

[116] A. Fraticelli, A. Robaglia-Schlupp, H. Riera, S. Monjanel-Mouterde, P. Cau, P. Astoul, Distribution of calibrated talc after intrapleural administration: an experimental study in rats, Chest 122(5) (2002) 1737-41.

[117] J. Ferrer, J.F. Montes, M.A. Villarino, R.W. Light, J. Garcia-Valero, Influence of particle size on extrapleural talc dissemination after talc slurry pleurodesis, Chest 122(3) (2002) 1018-27.

[118] E.C. Werebe, R. Pazetti, J.R. Milanez de Campos, P.P. Fernandez, V.L. Capelozzi, F.B. Jatene, F.S. Vargas, Systemic distribution of talc after intrapleural administration in rats, Chest 115(1) (1999) 190-3.

[119] W.J. Henderson, T.C. Hamilton, M.S. Baylis, C.G. Pierrepoint, K. Griffiths, The demonstration of the migration of talc from the vagina and posterior uterus to the ovary in the rat, Environ Res 40(2) (1986) 247-50.

[120] A.P. Wehner, R.E. Weller, E.A. Lepel, On talc translocation from the vagina to the oviducts and beyond, Food and chemical toxicology : an international journal published for the British Industrial Biological Research Association 24(4) (1986) 329-38.

[121] R. DerSimonian, N. Laird, Meta-analysis in clinical trials, Controlled clinical trials 7(3) (1986) 177-88.

[122] W. Viechtbauer, M.W. Cheung, Outlier and influence diagnostics for meta-analysis, Research synthesis methods 1(2) (2010) 112-25.

[123] Review Manager (RevMan), [Computer program] Version 5.3. Copenhagen: The Nordic Cochrane Centre, The Cochrane Collaboration, 2014.

[124] D.W. Cramer, L. Titus-Ernstoff, J.R. McKolanis, W.R. Welch, A.F. Vitonis, R.S. Berkowitz, O.J. Finn, Conditions associated with antibodies against the tumor-associated antigen MUC1 and their relationship to risk for ovarian cancer, Cancer Epidemiol Biomarkers Prev 14(5) (2005) 1125-31.

[125] A.F. Vitonis, L. Titus-Ernstoff, D.W. Cramer, A.F. Vitonis, L. Titus-Ernstoff, D.W. Cramer, Assessing ovarian cancer risk when considering elective oophorectomy at the time of hysterectomy, Obstet Gynecol 117(5) (2011) 1042-1050.

[126] N. Urban, S. Hawley, H. Janes, B.Y. Karlan, C.D. Berg, C.W. Drescher, J.E. Manson, M.R. Palomares, M.B. Daly, J. Wactawski-Wende, M.J. O'Sullivan, J. Thorpe, R.D. Robinson, D. Lane, C.I. Li, G.L. Anderson, Identifying post-menopausal women at elevated risk for epithelial ovarian cancer, Gynecologic Oncology 139(2) (2015) 253-60.

[127] M. Kurta, B. Diergaarde, The impact of fertility drug use, infertility, and lifetime ovulation on ovarian cancer risk, Cancer Prevention Research. Conference: AACR International Conference on Frontiers in Cancer Prevention Research 4(10 SUPPL. 1) (2011).
